# Access to *P*-stereogenic compounds *via* desymmetrizing enantioselective bromination†‡

**DOI:** 10.1039/d0sc07008d

**Published:** 2021-02-12

**Authors:** Qiu-Hong Huang, Qian-Yi Zhou, Chen Yang, Li Chen, Jin-Pei Cheng, Xin Li

**Affiliations:** State Key Laboratory of Elemento-Organic Chemistry, College of Chemistry, Nankai University Tianjin 300071 China xin_li@nankai.edu.cn; Center of Basic Molecular Science (CBMS), Department of Chemistry, Tsinghua University Beijing 100084 China

## Abstract

A novel and efficient desymmetrizing asymmetric *ortho*-selective mono-bromination of bisphenol phosphine oxides under chiral squaramide catalysis was reported. Using this asymmetric *ortho*-bromination strategy, a wide range of chiral bisphenol phosphine oxides and bisphenol phosphinates were obtained with good to excellent yields (up to 92%) and enantioselectivities (up to 98.5 : 1.5 e.r.). The reaction could be scaled up, and the synthetic utility of the desired *P*-stereogenic compounds was proved by transformations and application in an asymmetric reaction.

## Abstract 2

A highly efficient desymmetrizing asymmetric bromination of bisphenol phosphine oxides was developed, providing a wide range of chiral bisphenol phosphine oxides and bisphenol phosphinates with high yields and enantioselectivities.

## Graphical abstract

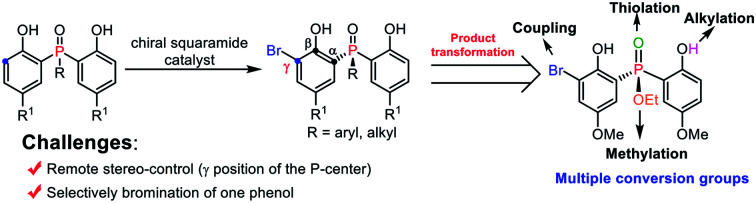

*P*-Stereogenic compounds are a class of privileged structures, which have been widely present in natural products, drugs and biologically active molecules (Fig. 1a).^1–4^ In addition, they are also important chiral materials for the development of chiral catalysts and ligands (Fig. 1b), because the chirality of the phosphorus atom is closer to the catalytic center which can cause remarkable stereo-induction.^5,6^ Thus, the development of efficient methods for the synthesis of *P*-stereogenic compounds with novel structures and functional groups is very meaningful.^5*a*^ Conventional syntheses of *P*-stereogenic compounds mainly depended on the resolution of diastereomeric mixtures and chiral-auxiliary-based approaches, in which stoichiometric amounts of chiral reagents are usually needed.^7^ By comparison, asymmetric catalytic strategies, including asymmetric desymmetric reactions of dialkynyl, dialkenyl, diaryl and bisphenol phosphine oxides,^8–14^ (dynamic) kinetic resolution of tertiary phosphine oxides,^15^ and asymmetric reactions of secondary phosphine oxides,^16^ can effectively solve the above-mentioned problems and have been considered as the most direct and efficient synthesis methods for constructing *P*-chiral phosphine oxides (Fig. 1c). Among them, organocatalytic asymmetric desymmetrization methods have been sporadic, in which the reaction sites were mainly limited to the hydroxyl group of bisphenol phosphine oxides that hindered their further transformation.^8–11^ It is worth mentioning that asymmetric desymmetrization methods, especially organocatalytic desymmetrization reactions, due to their unique advantages of mild reaction conditions and wide substrate scope, have become an important strategy for asymmetric synthesis. Accordingly, the development of efficient organocatalytic desymmetrization strategy for the synthesis of important functionalized *P*-stereogenic compounds which contain multiple conversion groups is very meaningful and highly desirable.

**Fig. 1:**
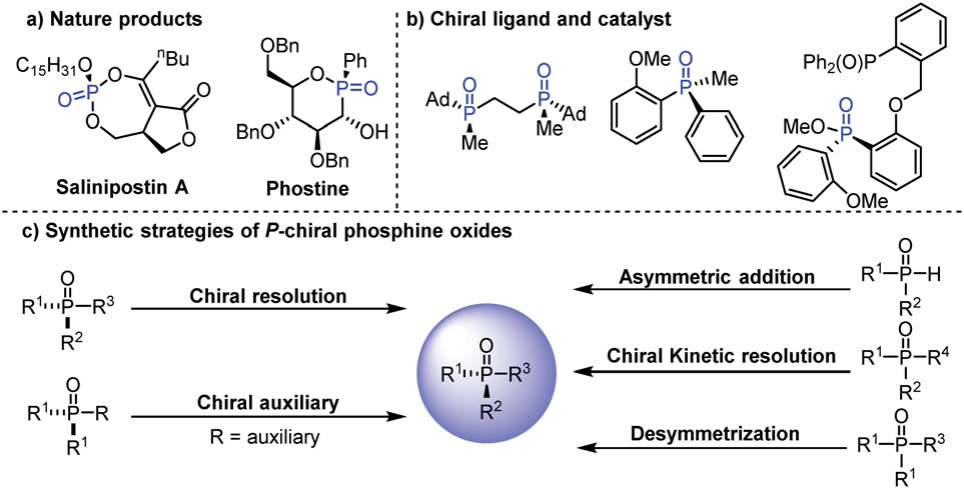
(a) Examples of natural products containing *P*-stereogenic centers. (b) *P*-Stereogenic compound type ligand and catalyst. (c) Typical *P*-stereogenic compounds' synthetic strategies.

On the other hand, asymmetric bromination has been demonstrated to be one of the most attractive approaches for chiral compound syntheses.^17^ Since the pioneering work on peptide catalyzed asymmetric bromination for the construction of biaryl atropisomers,^18*a*^ the reports on constructing axially biaryl atropisomers,^18^ C–N axially chiral compounds,^19^ atropisomeric benzamides,^20^ axially chiral isoquinoline *N*-oxides,^21^ and axially chiral *N*-aryl quinoids^22^ by electrophilic aromatic bromination have been well developed (Scheme 1a). In comparison, the desymmetrization of phenol through asymmetric bromination to construct central chirality remains a daunting task. Miller discovered a series of tailor made peptide catalyzed enantioselective desymmetrizations of diarylmethylamide through *ortho*-bromination (Scheme 1b).^23^ Recently, Yeung realized amino-urea catalyzed desymmetrizing asymmetric *ortho*-selective mono-bromination of phenol derivatives to fix a new class of potent privileged bisphenol catalyst cores with excellent yields and enantioselectivities (Scheme 1b).^24^ Despite this elegant work, there is no report on the synthesis of *P*-centered chiral compounds using the desymmetrizing asymmetric bromination strategy.

**Scheme 1:**
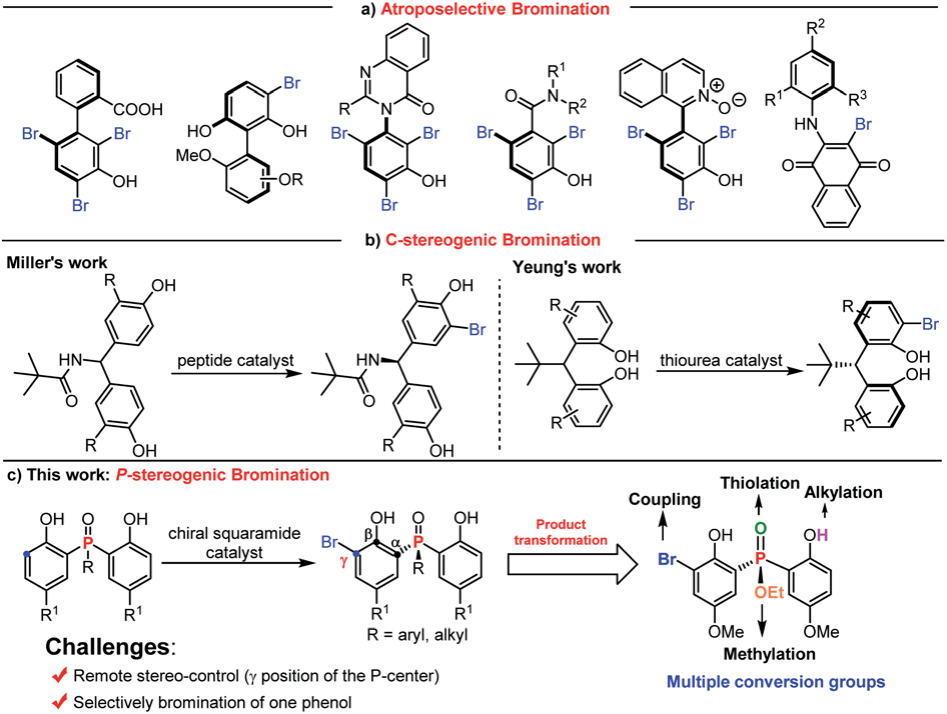
(a) Constructing axially chiral compounds by asymmetric bromination. (b) Known synthesis of central chiral compounds *via* asymmetric bromination. (c) This work: access to *P*-stereogenic compounds *via* desymmetrizing enantioselective bromination.

Taking into account the above-mentioned consideration, we speculated that bisphenol phosphine oxides and bisphenol phosphinates are potential substrate candidates for desymmetrizing asymmetric bromination to construct *P*-stereogenic centers. The advantages of using bisphenol phosphine oxides and bisphenol phosphinates as substrates are shown in two aspects. First, the *ortho*-position of electron rich phenol is easy to take place electrophilic bromination reaction. Second, the corresponding bromination product structure contains abundant synthetic conversion groups, including bromine, hydroxyl group, alkoxy group and phosphoryl group. To achieve this goal, two challenges need to be overcome: (i) finding a suitable chiral catalyst for the desymmetrization process to induce enantiomeric control is troublesome, due to the remote distance between the prochiral phosphorus center and the enantiotopic site; (ii) selectively brominating one phenol to inhibit the formation of an achiral by-product is difficult. Herein, we report a chiral squaramide catalyzed asymmetric *ortho*-bromination strategy to construct a wide range of chiral bisphenol phosphine oxides and bisphenol phosphinates with good to excellent yields and enantioselectivities (Scheme 1c). It is worth mentioning that the obtained *P*-stereogenic compounds can be further transformed at multiple sites.

Our initial investigation was carried out with bis(2-hydroxyphenyl)phosphine oxide **1a** and *N*-bromosuccinimide (NBS) **2a** as the model substrates, 10 mol% chiral amino-thiourea **4a** as the catalyst, and toluene as the solvent, which were stirred at −78 °C for 12 h. As a result, the reaction gave the desired desymmetrization product **3a** in 65% yield with 56 : 44 e.r. (Table 1, entry 1). Then, thiourea **4b** was tested, in which a little better result was obtained (Table 1, entry 2). To our delight, using the chiral squaramides **4c–4f** as the catalysts, the enantiomeric ratios of the desymmetrization products had been significantly improved (Table 1, entries 3–6). Especially, when chiral squaramide catalyst **4c** was applied to this reaction, the enantiomeric ratio of **3a** was increased to 95 : 5 (Table 1, entry 3). To further improve the yield and enantioselectivity, we next optimized the reaction conditions by varying reaction media and additives. As shown in Table 1, the reaction was affected by the solvent dramatically. Product **3a** was obtained with low yield and enantioselectivity in DCM (Table 1, entry 7). Also, when Et_2_O was used as the solvent, the yield and e.r. value of product **3a** were all decreased (Table 1, entry 8). As a result, the initial used toluene was the optimal solvent. We also inspected the effect of different bromine sources, and found that the initially used NBS was the optimal one (Table 1, entries 3, 11 and 12). Fortunately, by adjusting the amount of bisphenol phosphine oxides to 1.5 equiv., the yield and the enantiomeric ratio of **3a** were increased to 80% and 96.5 : 3.5, respectively (Table 1, entries 3, 13 and 14). Further increasing the amount of bisphenol phosphine oxides to 2.0 equiv. resulted in a reduced enantioselectivity (Table 1, entry 15).

**Table 1:** Optimization of the reaction conditions^a^

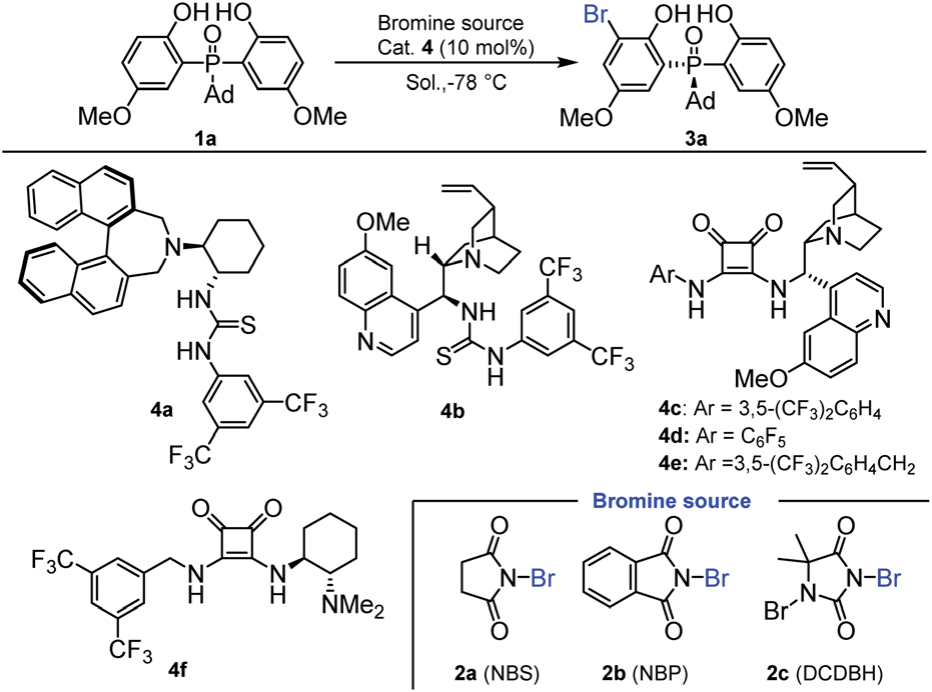					
Entry	Cat.	Bromine source	Solvent	Yield^b^ (%)	e.r.^c^
1	**4a**	**2a**	Toluene	65	56 : 44
2	**4b**	**2a**	Toluene	49	68 : 32
3	**4c**	**2a**	Toluene	61	95 : 5
4	**4d**	**2a**	Toluene	41	75 : 25
5	**4e**	**2a**	Toluene	53	93 : 6
6	**4f**	**2a**	Toluene	39	61 : 39
7	**4c**	**2a**	DCM	47	89 : 11
8	**4c**	**2a**	Et_2_O	39	67 : 33
9^d^	**4c**	**2a**	Toluene	69	94 : 6
10^e^	**4c**	**2a**	Toluene	61	93 : 7
11	**4c**	**2b**	Toluene	63	94 : 6
12	**4c**	**2c**	Toluene	65	87 : 13
13^f^	**4c**	**2a**	Toluene	75	95 : 5
14^g^	**4c**	**2a**	Toluene	80	96.5 : 3.5
15^h^	**4c**	**2a**	Toluene	79	95 : 5

a Reaction conditions: a mixture of **1a** (0.05 mmol), **2a** (0.05 mmol) and cat. **4** (10 mol%) in the solvent (0.5 mL) was stirred at −78 °C for 12 h.

b Isolated yield.

c Determined by HPLC analysis.

d 3 Å MS (10.0 mg) was used as the additive.

e 4 Å MS (10.0 mg) was used as the additive.

f **1a** : **2a** = 1.2 : 1.

g **1a** : **2a** = 1.5 : 1.

h **1a** : **2a** = 2.0 : 1.

Under the optimized reaction conditions, the scope of the desymmetrizing asymmetric *ortho*-selective mono-bromination of phosphine oxides was examined. Firstly, the variation of the *P*-center substituted group was investigated. As shown in Table 2, a variety of *P*-aryl, *P*-alkyl substituted phosphine oxides and phosphinates (**3a–3f**) were well amenable to this reaction and the corresponding *ortho*-brominated products were obtained in good yield (up to 87%) with high enantiomeric ratios (up to 98.5 : 1.5 e.r.). Moreover, regardless of whether the R was a bulky group or a smaller one, the enantiomeric ratios of the products were maintained at excellent levels. Especially, when the *P*-center substituted group was ethoxyl (**1e**), the corresponding bromination product **3e** was obtained in 80% yield with 98.5 : 1.5 e.r. When a *P*-methyl substituted phosphine oxide was used as the substrate, a moderate yield and enantiomeric ratio were obtained for **3g**.

**Table 2:** The scope of bisphenol phosphine oxides with different substituents on the *P*-atom^a^^,^^b^^,^^c^

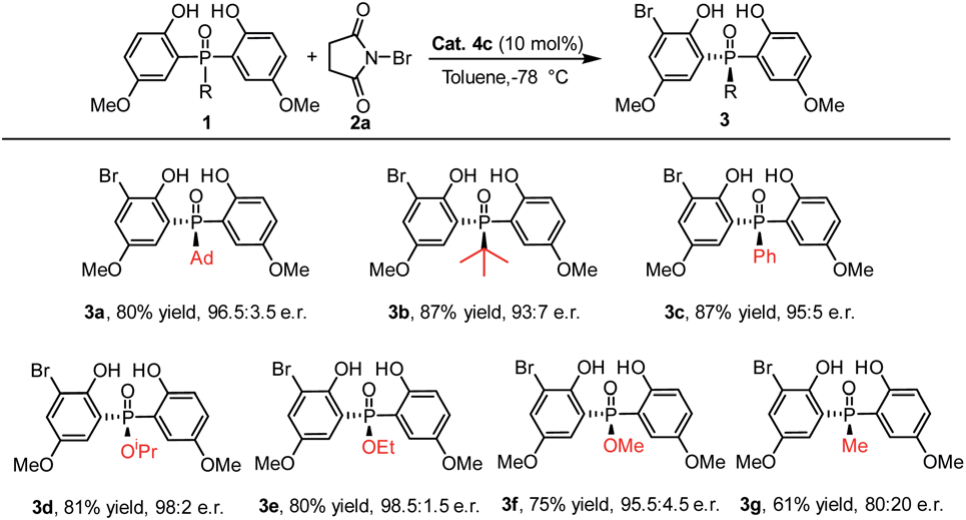

a Reaction conditions: a mixture of **1a** (0.15 mmol), **2a** (0.1 mmol) and **4c** (10 mol%) in toluene (1.0 mL) was stirred at −78 °C for 12 h.

b Isolated yield.

c Determined by HPLC analysis.

Next, using the ethoxyl substituted phosphinate as the template, a diversity of phosphinates with a 5-position substituent on the phenyl ring were examined (Table 3). To our delight, a range of phosphinates with different alkyl substituent on the phenyl ring was suitable for the currently studied reaction and the desired products **3h–3l** were obtained with very good enantioselectivities (90.5 : 9.5–97.5 : 2.5 e.r.). Furthermore, substrates with aryl and alkoxy groups at the 5-position of the phenol moiety were also tolerated well under the reaction conditions, and gave the products **3m–3q** with good to excellent yields (81–92%) and enantioselectivities (95 : 5–98.5 : 1.5 e.r.). Moreover, when a disubstituted phenol phosphinate substrate was used, the desired bromination product **3r** was also delivered with a good yield and e.r. value.

**Table 3:** The scope of bisphenol phosphinates^a^^,^^b^^,^^c^

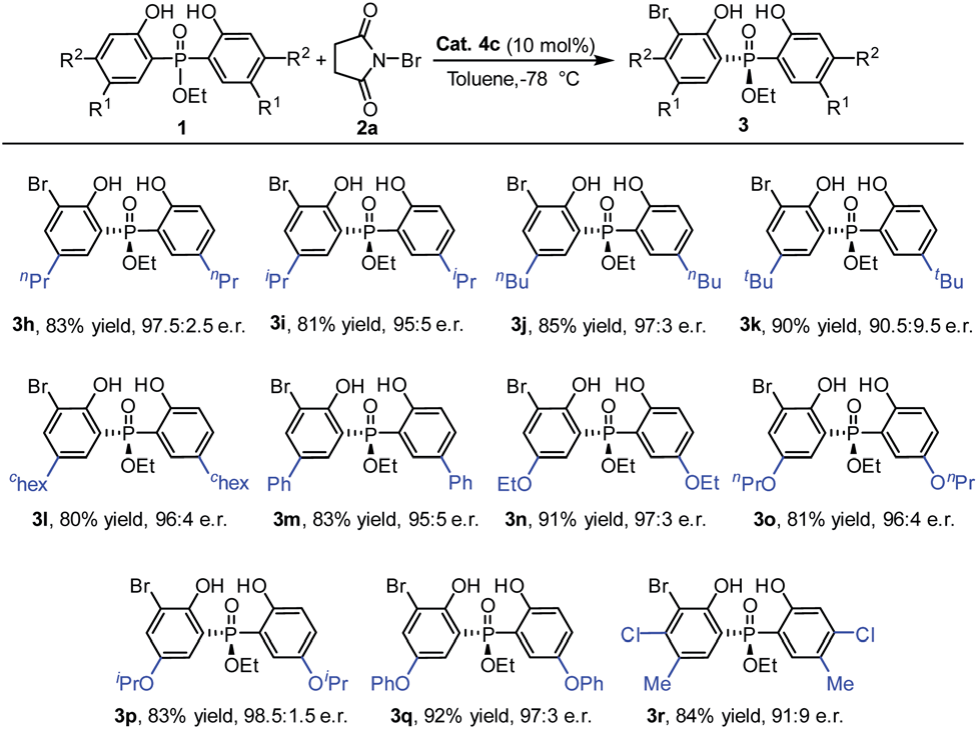

a Reaction conditions: a mixture of **1a** (0.15 mmol), **2a** (0.1 mmol) and **4c** (10 mol%) in toluene (1.0 mL) was stirred at −78 °C for 12 h.

b Isolated yield.

c Determined by HPLC analysis.

Then, we turned our attention to inspect the scope of *ortho*-bromination of *P*-adamantyl substituted phosphine oxides. As exhibited in Table 4, 5-methyl, 5-ethyl and 4,5-dimethyl aryl substituted phosphine oxides could be transformed into the corresponding products (**3s**, **3t** and **3u**) with excellent yields (81–89%) and enantioselectivities (95 : 5–96 : 4 e.r.). Upon increasing the size of the 5-position substituent on the phenyl ring of phosphine oxides, the enantioselectivities of the products **3v–3y** had a little decreasing tendency (81 : 19–93 : 7 e.r.). The absolute configuration of **3v** was determined by X-ray diffraction analysis and those of other products were assigned by analogy.^25^

**Table 4:** The scope of adamantyl substituted bisphenol phosphine oxides^a^^,^^b^^,^^c^

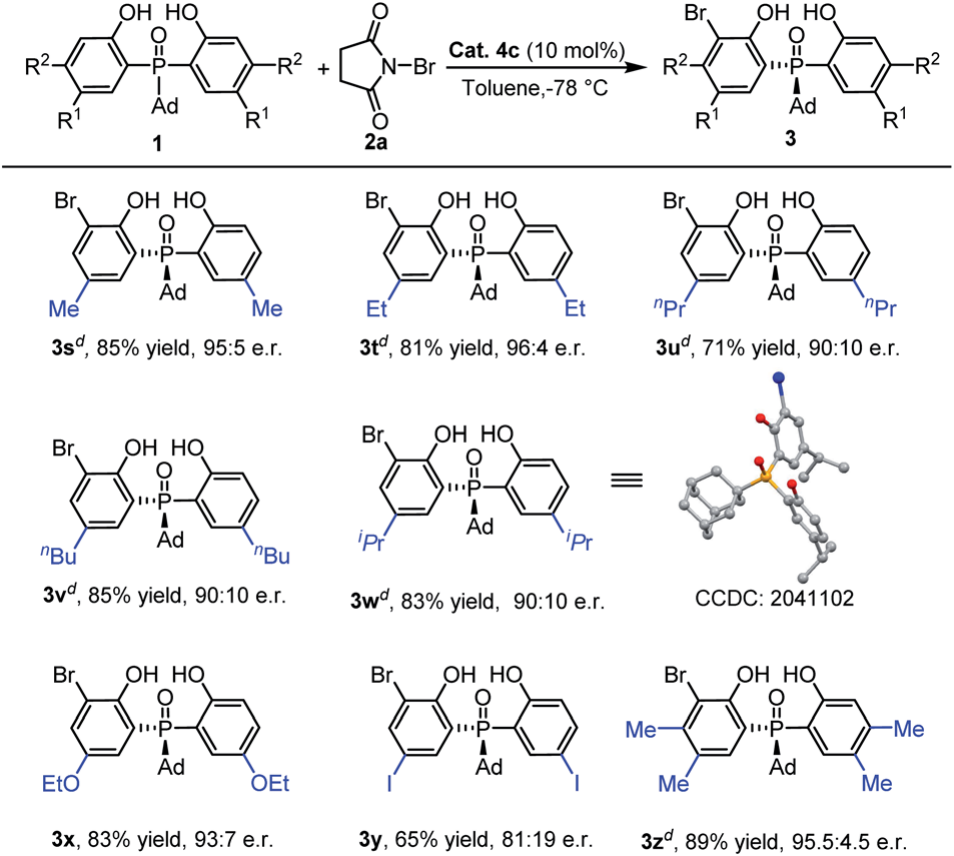

a Reaction conditions: a mixture of **1a** (0.15 mmol), **2a** (0.1 mmol) and **4c** (10 mol%) in toluene (1.0 mL) was stirred at −78 °C for 12 h.

b Isolated yield.

c Determined by HPLC analysis.^24^

d **1a** : **2a** = 1.2 : 1.

To demonstrate the utility of this desymmetrizing asymmetric *ortho*-selective mono-bromination, the reaction was scaled up to 1.0 mmol, and the corresponding product **3a** was obtained in 80% yield with 96.5 : 3.5 e.r. (98.5 : 1.5 e.r. after single recrystallization) (Scheme 2a). The encouraging results implied that this strategy had the potential for large-scale production. Additionally, the transformations of products **3a** and **3e** were also investigated (Scheme 2b). In the presence of Pd(OAc)_2_ and bulky electron-rich ligand *S*-Phos, **3a** could react with phenylboronic acid effectively, in which the desired cross-coupling product **5** was generated in high yield with maintained enantioselectivity. In the presence of Lawesson's reagent, **3a** could be transformed into thiophosphine oxide **6** with a high yield and e.r. value. Furthermore, **3e** could react with methyl lithium to afford the DiPAMP analogue **3g** in 85% yield with 98.5 : 1.5 e.r. And **3e** could also be converted to chiral bidentate Lewis base **7** by a straightforward alkylation reaction. It was encouraging to find that **7** could be used as a catalyst for the asymmetric reaction between *trans*-chalcone and furfural, in which the desired product **8** was furnished with moderate stereoselectivity (Scheme 2c).^26^

**Scheme 2:**
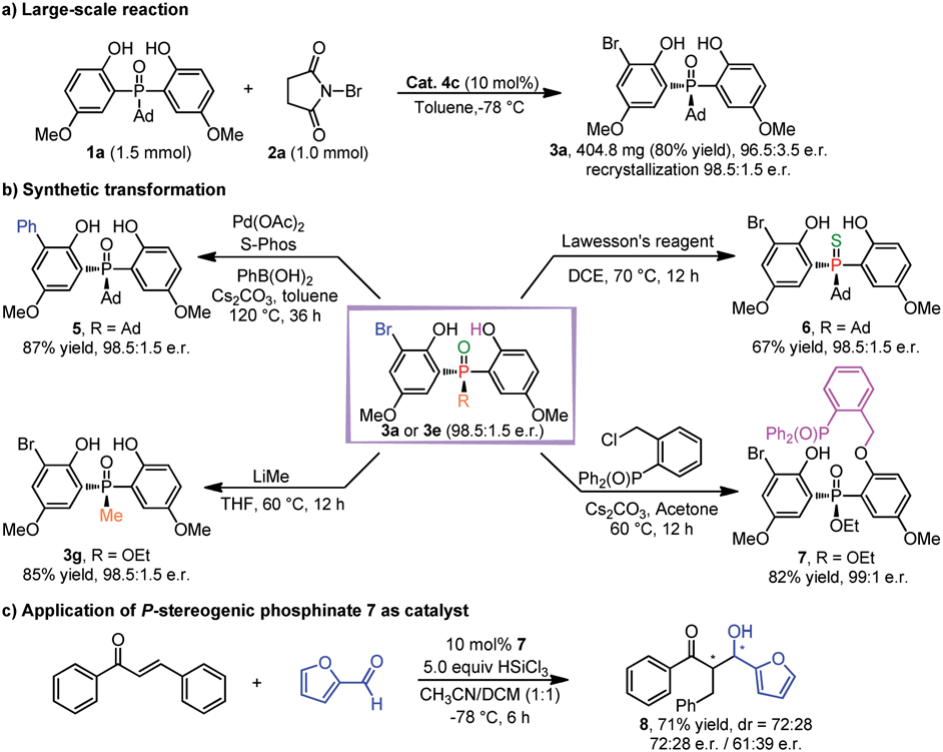
(a) Large-scale reaction. (b) Synthetic transformations. (c) Application of the transformed product.

Since the mono-bromination product **3a** could undergo further bromination to form the dibromo adduct, we wondered whether this second bromination is a kinetic resolution process. As shown in Scheme 3a, a racemic sample of **3a** was subjected to the catalytic conditions ((±)-**3a** and **2a** in a 2 : 1 molar ratio). Upon complete consumption of **2a** (with the formation of a dibromo product in 49% yield), the mono-bromination product **3a** was recovered in 51% yield with 99 : 1 e.r. This result indicated that the second bromination was indeed a kinetic resolution process and had a positive contribution to the enantioselectivity. Considering the excellent enantiomeric ratio of recovered **3a**, we further investigated the reaction of *rac*-**9** with **2a** under kinetic resolution conditions (Scheme 3b). To our delight, the unreacted raw material **9** can be obtained in 51% yield with 99.5 : 0.5 e.r., and chiral dihalogenated product **10** can also be generated in 49% yield with 90 : 10 e.r.

**Scheme 3:**
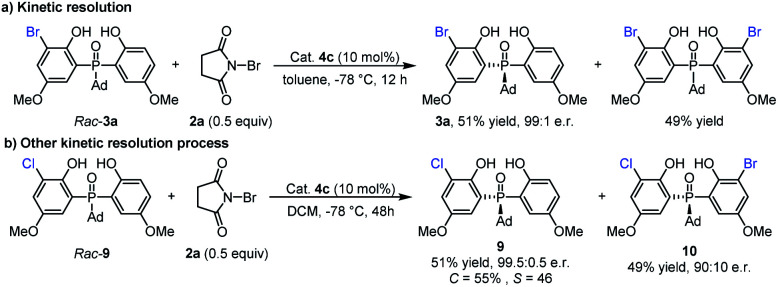
Kinetic resolution process.

To investigate the mechanism, we performed some control experiments. First, a mono-methyl protected phosphine oxide substrate was prepared and subjected to *ortho*-bromination under the optimal conditions. As shown in Scheme 4a, the corresponding product **11** was obtained with 72.5 : 27.5 e.r. When the same reaction conditions were applied to the dimethyl protected phosphine oxide substrate, no reaction occurred (Scheme 4b). These results indicated that the phenol moieties of the substrate were essential for the bromination reaction. In fact, hydrogen bonds formed between the two phenolic hydroxyl groups and P=O could be observed in the single crystal structure of the product **3w**.^25^ Furthermore, when thiophosphine oxide, which had a weak hydrogen bond acceptor P=S group, was prepared and tested in the reaction, the corresponding product **6** was obtained with a lower yield and enantioselectivity than that of **3a** (Scheme 4c). This result suggested that the intramolecular hydrogen bonds of the substrate might be beneficial for both the reactivity and the enantioselectivity.^27^ In light of the control experiments and previous studies,^24^ two possible mechanisms were proposed (see the ESI‡).

**Scheme 4:**
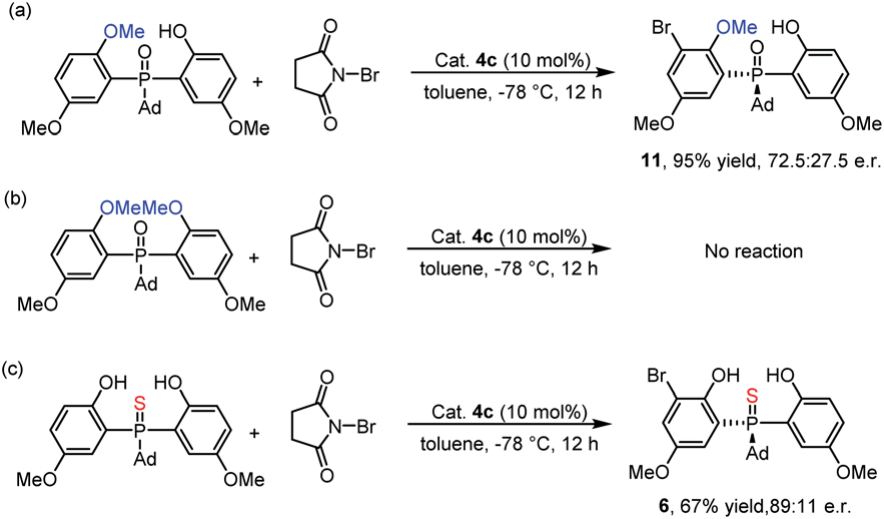
Control experiments: (a) mono-methyl protected phosphine oxide substrate was evaluated; (b) dimethyl protected phosphine oxide substrate was examined; (c) thiophosphine oxide substrate was investigated.

In summary, a novel and efficient desymmetrizing asymmetric *ortho*-selective mono-bromination of bisphenol phosphine oxides under chiral squaramide catalysis was reported. Using this asymmetric *ortho*-bromination strategy, a wide range of chiral bisphenol phosphine oxides and bisphenol phosphinates were obtained with good to excellent yields and enantioselectivities. The reaction could be scaled up, and the synthetic utility of the desired *P*-stereogenic compounds was proved by transformations and application in an asymmetric reaction. Ongoing studies focus on the further mechanistic investigations and the potential applications of these chiral *P*-stereogenic compounds in other asymmetric transformations.

## Conflicts of interest

There are no conflicts to declare.

## Supplementary Material

SC-012-D0SC07008D-s001

SC-012-D0SC07008D-s002
