# Supplementary material for: Access to P-stereogenic compounds via desymmetrizing enantioselective bromination
Source: Chem Sci. 2021 Feb 12;12(12):4582–7. doi: 10.1039/d0sc07008d (PMC8179578; doi:10.1039/d0sc07008d)
Supplement: SC-012-D0SC07008D-s001 [file SC-012-D0SC07008D-s001.pdf]

Electronic Supplementary Material (ESI) for Chemical Science.
This journal is © The Royal Society of Chemistry 2021

Electronic Supplementary Information

Access to *P*-Stereogenic Compounds via Desymmetrizing Enantioselective Bromination

Qiu-Hong Huang, Qian-Yi Zhou, Chen Yang, Li Chen, Jin-Pei Cheng and Xin Li*

Abstract: A novel and efficient desymmetrizing asymmetric *ortho*-selective *mono*-bromination of bisphenol phosphine oxides under chiral squaramide catalysis was reported. Using this asymmetric *ortho*-bromination strategy, a wide range of chiral bisphenol phosphine oxides and bisphenol phosphinates were obtained with good to excellent yields (up to 92%) and enantioselectivities (up to 98.5:1.5 e.r.). The reaction could be scaled up, and the synthetic utility of the desired *P*-stereogenic compounds was proved by transformations and application in the asymmetric reaction.

DOI:

Contents

1. General procedure for the synthesis of the substrates........3

2. General procedure for this reaction........3

3. Large-scale reaction and further transformations........4

4. Kinetic resolution........5

5. Control experiment........6

6. Crystal structure data of 3w........8

7. DFT calculation for nucleophilicity of phosphine oxides........9

8. Proposed possible mechanism........13

9. Characterization of products........14

10. NMR and HPLC spectra........28

NMR spectra........28

HPLC spectra........78

11. References........111

General information

Commercially available materials purchased was used as received. ^{1}H NMR were recorded on a Bruker Avance (400 MHz) spectrometer, and reported as δ in units of parts per million (ppm) relative to tetramethylsilane (δ 0.00), and splitting patterns are designated as singlet (s), doublet (d), triplet (t), quartet (q), dd (doublet of doublets), m (multiplets). ^{13}C NMR were reported on a Bruker Avance (101 MHz) spectrometer, and reported as δ in units of parts per million (ppm) relative to the signal of chloroform-d (δ 77.16 triplet). ^{31}P NMR were reported on a Bruker Avance (162 MHz) spectrometer. Mass spectra were obtained using electrospray ionization (ESI) mass spectrometer.

1. General procedure for the synthesis of the substrates [1-2]

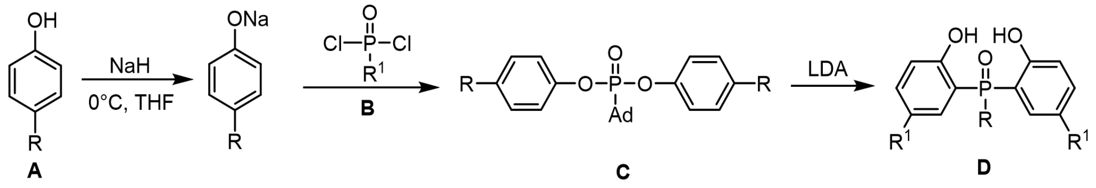

To a dry round bottomed flask equipped with a magnetic stir bar, added phenols **A** (1 equiv) in THF, then NaH (1.2 equiv) was added with nitrogen. The reaction was stirring at 0 °C for 30 minutes. When the reaction completed, **B** (0.5 equiv) was added to the mixture at 0 °C for 1h with nitrogen, and then 24 h at room temperature. Extracted with $CHCl_3$ and the organic phase was dried over $MgSO_4$.The resulting crude residue was purified *via* column chromatography on silica gel to afford the desired products **C**.

To a dry round bottomed flask equipped with a magnetic stir bar, added LDA (4 equiv) at -78 °C, **C** (1 equiv) dissolved in pure and dry THF was added in 60 min at -78 °C. The resulting reaction mixture was stirred at -78 °C for another 60 min, then it was allowed to warm up to rt and it was stirred at rt for 12 h. After the reaction was completed, quenched with saturated aqueous NH_4Cl solution, then extracted with $CHCl_3$. The organic phase was separated and the combined organic phase was dried over $MgSO_4$, filtered and the solvent was removed. The crude product was first purified by chromatography on silica gel to afford the products **D**.

2. General procedure for this reaction

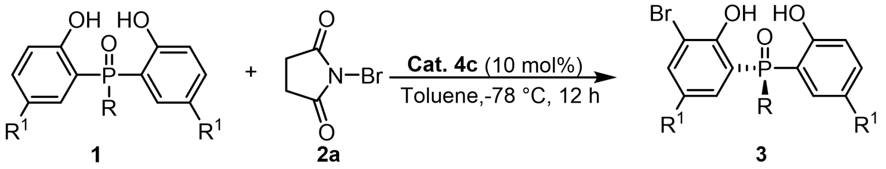

To a solution of toluene (1.0 mL) were added phosphine oxide **1** (0.15 mmol), NBS **2a** (0.1 mmol) and catalyst **4c** (0.01 mmol). The reaction mixture was stirred at -78 ^{o}C for 12 h. The solvent was evaporated to give the crude product, which was directly purified by silica gel chromatography to provide the desired product **3**.

3. Large-scale reaction and further transformations

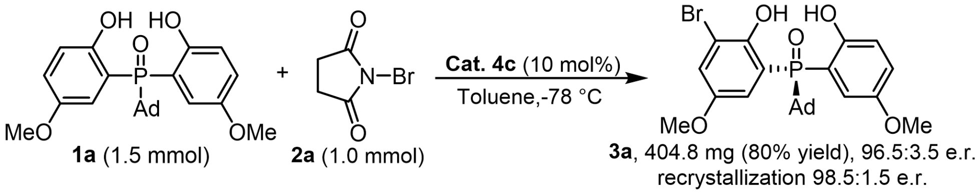

To a solution of toluene (5.0 mL) were added phosphine oxide **1a** (640.0 mg, 1.5 mmol), NBS **2a** (178.0 mg, 1.0 mmol) and catalyst **4c** (63.0 mg, 0.1 mmol). The reaction mixture was stirred at -78 ^{o}C for 12 h. The solvent was evaporated to give the crude product, which was directly purified by silica gel chromatography to provide the desired product **3a** as a white solid (404.8 mg, 80% yield, 96.5:3.5 e.r. and 98.5:1.5 e.r. after one recrystallization).

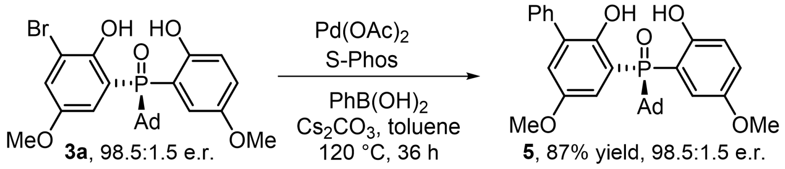

To an oven-dried 10 mL Schlenk flask equipped with a stir bar and Graham con-denser was added $Pd(OAc)_2$ (5 mmol%) and S-Phos (10 mmol%). The flask was evacuated and back-filled with nitrogen. Then dry toluene (1 mL) was added and the solution was stirred at room temperature for 5 min. To the solution were added phosphine oxide **3a** (0.1 mmol), boronic acid (0.15 mmol), and Cs_2CO_3 (0.3 mmol) successively under nitrogen atmosphere. The mixed solution was heated to 120 ºC and stirred for 36 h, after which the resulting mixture was allowed to cool to room temperature and purified by silica gel chromatography to afford chiral phosphine oxides **5** (87% yield, 98.5:1.5 e.r.).

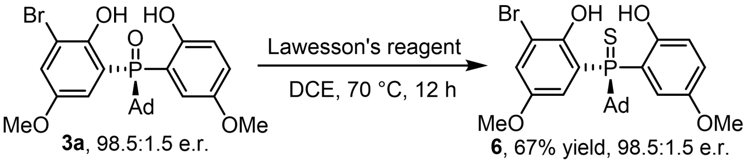

3a, 98.5:1.5 e.r. **6**, 67% yield, 98.5:1.5 e.r.

To a 10 ml RBF equipped with a magnetic stir bar, was added phosphinate **3a** (0.1 mmol), Lawesson's reagent (0.5 mmol) and dry 1, 2-dichloroethane (2 mL). The flask was placed in 70 °C oil bath stirred for 12 h. After the reaction completed, the mixture was being evaporated. Then the residue was purified by column chromatography on silica gel to afford **6** (67% yield, 98.5:1.5 e.r.).

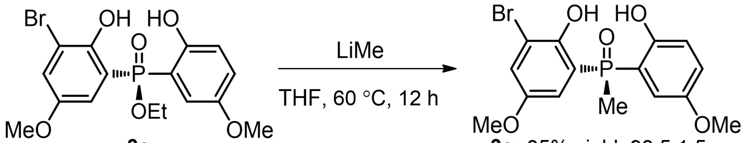

3e **3g**, 85% yield, 98.5:1.5 e.r.

To a dry Schlenk tube equipped with a magnetic stir bar, was added phosphinate **3e** (0.1mmol). The tube was closed with a septum, evacuated, and refilled with nitrogen. Freshly distilled THF (1 mL) was added and the reaction mixture was then stirred at 0 °C for 5 minutes, followed by methyl lithium solution (0.5 mmol) dropwise. The reaction placed in 60 °C oil bath with water-jacketed condenser and stirred for 12 hours. Upon the reaction completed, the mixture was quenched with sat. NH_4Cl (5 mL), extracted by EA (10 mL*3), dried with $MgSO_4$. The organic solvent was concentrated under reduced pressure, and the resulting crude residue was purified *via* column chromatography on silica gel to afford the desired product **3g** (85% yield, 98.5:1.5 e.r.).

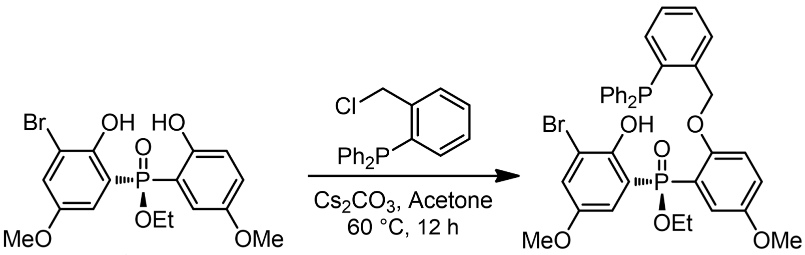

3e **7**, 82% yield, 99:1 e.r.

To a 10 ml RBF equipped with a magnetic stir bar, was added phosphinate **3e** (0.1mmol), (2-(chloromethyl)phenyl)diphenylphosphane (0.1 mmol), Cs_2CO_3 (0.2 mmol) and acetone (1 mL). The flask was placed in 60 °C oil bath with water-jacketed condenser and stirred for 6 hours. After the reaction completed, the mixture was filtered. Then the filtrate was evaporated and the crude mixture was purified via column chromatography on silica gel to afford **7** (82% yield, 99:1 e.r.).

4. Kinetic resolution

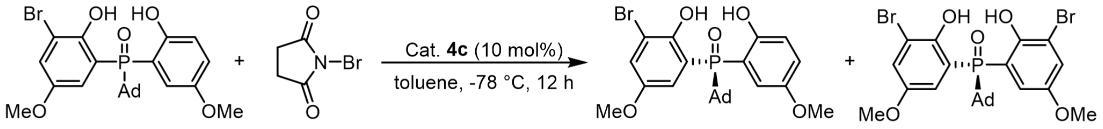

Rac-**3a** **2a** (0.5 equiv) **3a**, 51% yield, 99:1 e.r. 49% yield

To a solution of toluene (1.0 mL) were added phosphine oxide *rac*-**3a** (0.1 mmol), **2a** (0.05 mmol) and catalyst **4c** (0.01 mmol). The reaction mixture was stirred at -78 °C for 12 h. The solvent was evaporated to give the crude product, which was directly purified by silica gel chromatography to afford the dibrominated product (49% yield) and recover the unreacted **3a** (46% yield, 99:1 e.r.).

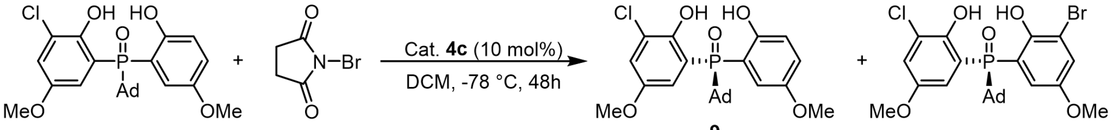

Rac-**9** **2a** (0.5 equiv) **9** 51% yield, 99.5:0.5 e.r. *C* = 55% , *S* = 46 **10** 49% yield, 90:10 e.r.

To a solution of DCM (1.0 mL) were added phosphine oxide *rac*-**9** (0.1 mmol), **2a** (0.05 mmol) and catalyst **4c** (0.01 mmol). The reaction mixture was stirred at -78 °C for 12 h. The solvent was evaporated to give the crude product, which was directly purified by silica gel chromatography to afford the unreacted raw material **9** (51% yield, 99.5:0.5 e.r.) and chiral dihalogenated product **10** (49% yield, 90:10 e.r.).

5. Control experiment

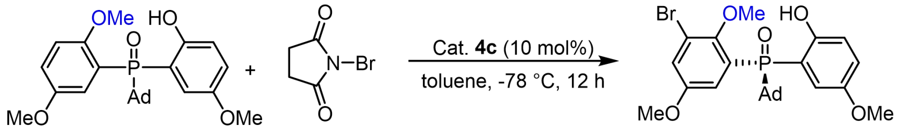

11, 95% yield, 72.5:27.5 e.r.

To a solution of toluene (1.0 mL) were added mono-*O*-methylated substrate (0.15 mmol), **2a** (0.1 mmol) and catalyst **4c** (0.01 mmol). The reaction mixture was stirred at -78 °C for 12 h. The solvent was evaporated to give the crude product, which was directly purified by silica gel chromatography to afford the product **11** (95% yield, 72.5:27.5 e.r.)

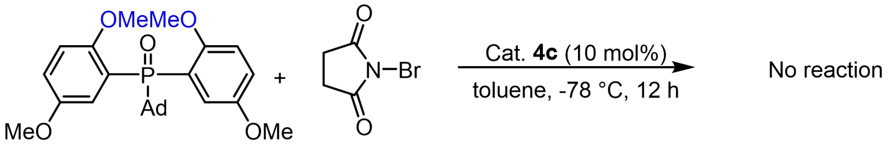

To a solution of toluene (1.0 mL) were added di-*O*-methylated substrate (0.15 mmol), **2a** (0.01 mmol) and catalyst **4c** (0.01 mmol). The reaction mixture was stirred at -78 °C for 12 h and no reaction was observed by TLC.

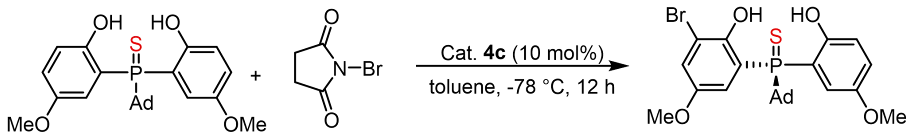

6, 67% yield,89:11 e.r.

To a solution of toluene (1.0 mL) were added mono-*O*-methylated substrate (0.15 mmol), **2a** (0.1 mmol) and catalyst **4c** (0.01 mmol). The reaction mixture was stirred at -78 °C for 12 h. The solvent was evaporated to give the crude product, which was directly purified by silica gel chromatography to afford the product **6** (67% yield, 89:11 e.r.)

6. Crystal structure data of 3w

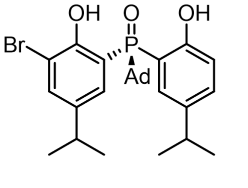

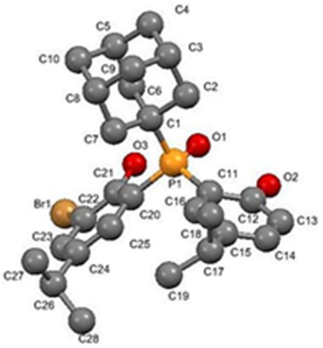

Table 1 Crystal data and structure refinement for 3w: 2041102.

Identification code	**3w**: 2041102
Empirical formula	$C_{28}H_{36}BrO_3P$
Formula weight	531.45
Temperature/K	294.15
Crystal system	orthorhombic
Space group	$P2_12_12_1$
a/Å	9.15691(7)
b/Å	16.71291(19)
c/Å	17.53844(17)
α /°	90
β /°	90
γ /°	90
Volume/Å^3	2684.06(5)
Z	4
ρ_{calc}g/cm^3	1.315
μ /mm^{-1}	2.851
F(000)	1112.0
Crystal size/mm^3	0.18 × 0.16 × 0.14
Radiation	CuK$^{\alpha}$ (λ = 1.54184)
2$^{\Theta}$ range for data collection/°	7.306 to 158.54
Index ranges	-11 ≤ h ≤ 11, -21 ≤ k ≤ 20, -17 ≤ l ≤ 22
Reflections collected	19439
Independent reflections	5564 [Rint = 0.0356, Rsigma = 0.0220]
Data/restraints/parameters	5564/12/315
Goodness-of-fit on F^2	1.039
Final R indexes [I>=2σ (I)]	R1 = 0.0381, wR2 = 0.1076
Final R indexes [all data]	R1 = 0.0398, wR2 = 0.1094
Largest diff. peak/hole / e Å^{-3}	0.46/-0.19
Flack parameter	-0.031(12)

7. DFT calculations for nucleophilicity of phosphine oxides

We carried out the computational calculations to study the nucleophilicity of the phosphine oxides (**1**-**2**) and thiophosphine oxide (**3**). The condensed local nucleophilicity index within the framework of conceptual density funcitional theory (CDFT)[3] was calculated to evaluate nucleophilicity of C7 sites (colored in red) of **1**-**3**[4]. Geometries were optimized in solution phase at the B3LYP-D3/6-311G(d,p)-SMD(toluene) level with Guassian16[5]. The optimized geometries were employed for the *N*, *N*+1, and *N*-1 electron states, where *N* refers to the number of electrons of a target molecule. The condensed local nucleophilicity index was calculated with Multiwfn[6] package. We could find that the nucleophilicity of phosphine oxide **2** which had intramolecular hydrogen bonds was stronger than phosphine oxide **1**. At the same time, the nucleophilicity of phosphine oxide **2** was still stronger than thiophosphine oxide **3** which had weak hydrogen bond acceptor P=S group. These calculation results indicated that the intramolecular hydrogen bonds of the substrate was indispensable for the enantioselective *ortho*-bromination, which was consistent with the observation of the control experiment.

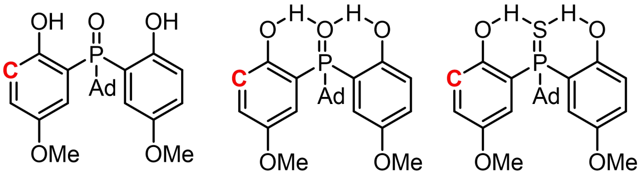

1 **2** **3**

Compounds	1	2	3
Condensed local nucleophilicity index (e*eV)	0.13928	0.15781	0.13003

Cartesian Coordinates

1

C	1.58118200	2.92311000	-1.13521300
C	1.13111000	4.12551100	-0.59242200
C	3.54098300	-2.64124400	0.33191400
C	2.30550700	-2.98448900	-0.21062000
C	1.18586500	1.72177100	-0.54512500
C	0.29031400	4.10312600	0.51867300
C	3.65016700	-1.48885500	1.10952000
C	1.19922800	-2.16561900	0.03286200
C	2.54865100	-0.67719700	1.35340600
C	0.34718800	1.68623800	0.56728600
C	-0.11143700	2.90377900	1.10227900
C	1.29255600	-1.00892700	0.80168300
H	1.41980800	5.07798800	-1.01483800

H	4.41894400	-3.24949500	0.16498000
H	1.56325500	0.80326200	-0.97541500
H	-0.06084000	5.04128500	0.93844200
H	0.26378900	-2.47339600	-0.40480800
H	3.54641200	0.55776900	2.41918000
H	-1.17208000	3.78655900	2.41767700
O	2.64092000	0.45295300	2.10718900
O	-0.95544900	2.88045400	2.17143200
O	-0.43866000	-0.01958300	2.72081700
P	-0.14824800	0.04843100	1.24988000
H	4.61238400	-1.22080800	1.53542700
C	-4.55501200	-1.05911000	0.33164100
H	-5.50294900	-1.29805000	-0.16353100
H	-4.74457700	-1.05031900	1.41028500
C	-4.05187800	0.31877200	-0.12682600
H	-4.79026600	1.08588600	0.12788600
C	-3.49742200	-2.12163700	-0.00610400
H	-3.84353700	-3.10627300	0.32374500
C	-3.24957700	-2.14137500	-1.52489600
H	-4.17191700	-2.40954300	-2.05188500
H	-2.50367400	-2.90489000	-1.77362000
C	-3.82322200	0.30195400	-1.64710700
H	-3.48998200	1.28850400	-1.98804200
H	-4.76118500	0.07837300	-2.16756300
C	-2.76302600	-0.75730600	-1.98963000
H	-2.58974500	-0.77107700	-3.07036000
C	-1.43987700	-0.40874600	-1.28091300
H	-0.67027900	-1.12148600	-1.58118400
H	-1.10002000	0.57936600	-1.60199400
C	-2.73012000	0.65267800	0.58627900
H	-2.87836200	0.69517200	1.66699700
H	-2.38638500	1.63954900	0.26781700
C	-2.18477800	-1.78794100	0.72461100
H	-2.34748700	-1.75193400	1.80436100
H	-1.45765500	-2.58100000	0.54161000
C	-1.65104800	-0.41387500	0.24898700
O	2.06808700	-4.08379300	-0.98533500
O	2.40281400	2.81367400	-2.22161600
C	3.16130500	-4.95052800	-1.25746600
H	3.57484700	-5.37829200	-0.33713600
H	2.76039200	-5.75303100	-1.87524000
H	3.95584300	-4.43524000	-1.80925200
C	2.82362900	4.01304900	-2.85629100
H	3.45537700	3.70580600	-3.68884700

H	1.97250800	4.58459200	-3.24396300
H	3.40766500	4.64577400	-2.17799200

2

C	1.26295000	3.37591300	-0.68784500
C	0.73108200	4.37200500	0.13685900
C	3.46792500	-2.81380500	0.47953500
C	2.95779200	-2.10492700	-0.61281900
C	1.00521900	2.04124800	-0.38831500
C	-0.03119500	4.02364400	1.24428700
C	2.87621900	-2.68192600	1.72748500
C	1.85814000	-1.27526200	-0.42316600
C	1.77080900	-1.85143100	1.93694200
C	0.22581700	1.68043300	0.71460700
C	-0.29579700	2.68937100	1.56028600
C	1.25306900	-1.12996300	0.83169500
H	0.91242900	5.41839300	-0.06681900
H	4.32130800	-3.46907100	0.37062700
H	1.44006000	1.29339700	-1.03587400
H	-0.43317600	4.78926800	1.89677700
H	1.48746900	-0.74748500	-1.28879200
H	0.49274300	-1.18818800	3.19242300
H	-1.01949300	1.47790900	2.84904100
O	1.26904000	-1.80030400	3.18688700
O	-1.04972500	2.44473700	2.65535600
O	-0.62040200	-0.14251600	2.54752700
P	-0.18401900	-0.05182800	1.07369100
H	3.26424000	-3.22795200	2.57878700
C	-4.34462800	-1.60328500	-0.11256000
H	-5.23970800	-1.91821000	-0.65967600
H	-4.55464000	-1.73258300	0.95452700
C	-4.03698300	-0.12651700	-0.41318200
H	-4.88498300	0.49674200	-0.11410500
C	-3.14439200	-2.47361500	-0.52263300
H	-3.35846800	-3.52477500	-0.30742000
C	-2.86881100	-2.29588500	-2.02563500
H	-3.73797200	-2.62141200	-2.60709600
H	-2.02314000	-2.92281800	-2.32899800
C	-3.76357800	0.05092900	-1.91567300
H	-3.55857700	1.10385600	-2.13792700
H	-4.64688300	-0.23671200	-2.49589400
C	-2.56220800	-0.81765900	-2.32196700
H	-2.35558900	-0.68928700	-3.38873700
C	-1.31536700	-0.38658200	-1.52502800

H	-0.46951300	-1.00254400	-1.83663400
H	-1.07246700	0.65624500	-1.74654100
C	-2.79806300	0.31196500	0.38685800
H	-2.98805700	0.21198400	1.45760800
H	-2.58072100	1.36550400	0.18674300
C	-1.90212400	-2.04735700	0.28104400
H	-2.08117800	-2.18165300	1.35126000
H	-1.04679700	-2.67324700	0.00941300
C	-1.58323100	-0.56325000	-0.01574100
O	3.45511000	-2.15916700	-1.88379700
O	2.03411700	3.60552500	-1.79136600
C	4.59925200	-2.97124300	-2.11537500
H	4.39480500	-4.02668200	-1.90296300
H	4.83481600	-2.85997400	-3.17291700
H	5.45645900	-2.63822200	-1.51957600
C	2.33945600	4.95505700	-2.11850200
H	2.96606700	4.91199200	-3.00834300
H	1.43445200	5.53013600	-2.34526900
H	2.89357800	5.44944500	-1.31279400

3

C	1.81998300	2.75380400	-1.27419500
C	1.35743700	3.99744700	-0.83373800
C	3.45295100	-2.69413600	0.43610700
C	2.29275700	-2.91471400	-0.30852500
C	1.35581700	1.60039200	-0.64877100
C	0.45584600	4.06722500	0.22066000
C	3.47605900	-1.68588300	1.39260700
C	1.18243900	-2.09936200	-0.09637500
C	2.36955300	-0.87240800	1.62446500
C	0.43258500	1.66183400	0.40023700
C	-0.01969200	2.91933200	0.85532000
C	1.20314200	-1.07047100	0.84703000
H	1.69943400	4.91495700	-1.29256300
H	4.33493500	-3.30259100	0.29150900
H	1.73048200	0.64712400	-0.99727000
H	0.10623600	5.02696300	0.58109500
H	0.31187900	-2.29316700	-0.70057500
H	1.61431000	0.31754500	2.92956700
H	-0.96689000	2.28090500	2.40326100
O	2.49626900	0.09745700	2.56272000
O	-0.91328500	3.09577000	1.85854100
P	-0.18497200	0.08653500	1.08111300
H	4.36310100	-1.51605700	1.99031100

C	-4.58460600	-1.03755600	0.07279300
H	-5.51630700	-1.27135800	-0.45332000
H	-4.80144700	-1.06542000	1.14577300
C	-4.08936400	0.36079000	-0.33001300
H	-4.84077800	1.11092200	-0.06662200
C	-3.50730400	-2.07781500	-0.27305100
H	-3.84883200	-3.07610300	0.01601100
C	-3.21907000	-2.04359600	-1.78405900
H	-4.12337900	-2.30537300	-2.34336900
H	-2.45672600	-2.78846500	-2.03847200
C	-3.82220900	0.39710600	-1.84270700
H	-3.49329300	1.39765900	-2.14337500
H	-4.74354900	0.17875300	-2.39306600
C	-2.74134500	-0.63824300	-2.18906300
H	-2.53293200	-0.61318400	-3.26280800
C	-1.44239000	-0.29401100	-1.43460100
H	-0.65399500	-0.98080600	-1.74274600
H	-1.11340500	0.71167400	-1.70585700
C	-2.79267300	0.68716700	0.43318700
H	-2.98674600	0.68186000	1.50724600
H	-2.44962500	1.68908600	0.16493400
C	-2.21654100	-1.75863600	0.50383000
H	-2.41058500	-1.76947300	1.57927600
H	-1.46973300	-2.52900400	0.30538700
C	-1.70174500	-0.36177000	0.08893500
O	2.14454700	-3.88065800	-1.26046800
O	2.71038700	2.56683600	-2.29126900
C	3.25631600	-4.72780800	-1.52690300
H	3.54135400	-5.30757700	-0.64209800
H	2.93002600	-5.40975600	-2.31083200
H	4.12031200	-4.15652600	-1.88410300
C	3.22776900	3.72047600	-2.94230400
H	3.91495500	3.35081000	-3.70215600
H	2.43413800	4.29989300	-3.42758300
H	3.77614000	4.36420600	-2.24555400
S	-0.60450700	0.23401600	3.05749400

8. Proposed possible mechanism

In light of the conducted control experiments, the crystal structure analysis of the product[7] and previous studies,[8] the two possible mechanisms were proposed. In **path a**, the squaramide catalyst first activates NBS to initiate the reaction. And the Br cation is transferred to the tertiary amine nitrogen atom of the catalyst,

whereas the succinimide anion is bonded to the squaramide moiety through hydrogen bonds. In **path b**, the squaramide catalyst activates NBS and bisphenol phosphine oxides through hydrogen bonds. Thus, four putative transition states were proposed to account for the observed enantioselectivity. In the **TS1** (or **TS3**) leading to the major enantiomer product, the admantyl (Ad) group is orienting to the opposite direction of the 3,5-bis(trifluoromethyl)phenyl moiety. However, the Ad group in the **TS2** (or **TS4**), leading to the minor enantiomer product, approaches to the 3,5-bis(trifluoromethyl)phenyl moiety. The repulsive steric effect between the bulky Ad and the catalyst should make the **TS2** (or **TS4**) less stable than **TS1** (or **TS3**) in favor of the formation of major enantiomer. In addition, **TS1** (or **TS3**) may also stabilized by C-H···F interactions between hydrogens of phenol and trifluoromethyl of catalyst, which are missing in **TS2** (or **TS4**). The C-H···F hydrogen bonds might be another key factor to the excellent enantio-control.

path a

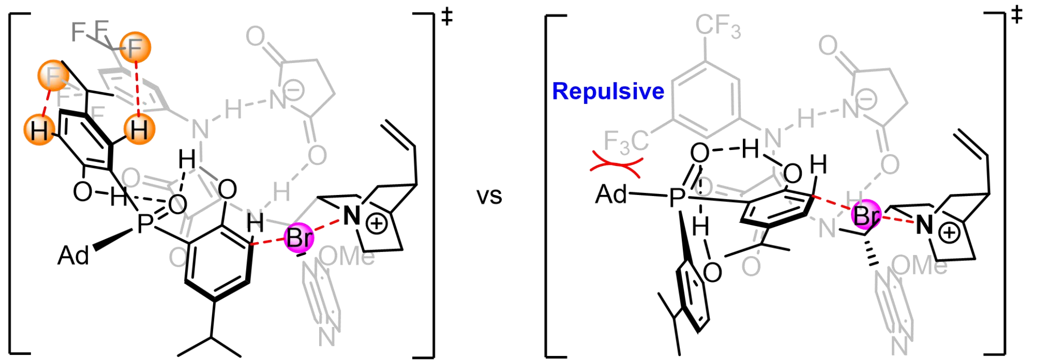

TS1 **TS2**

path b

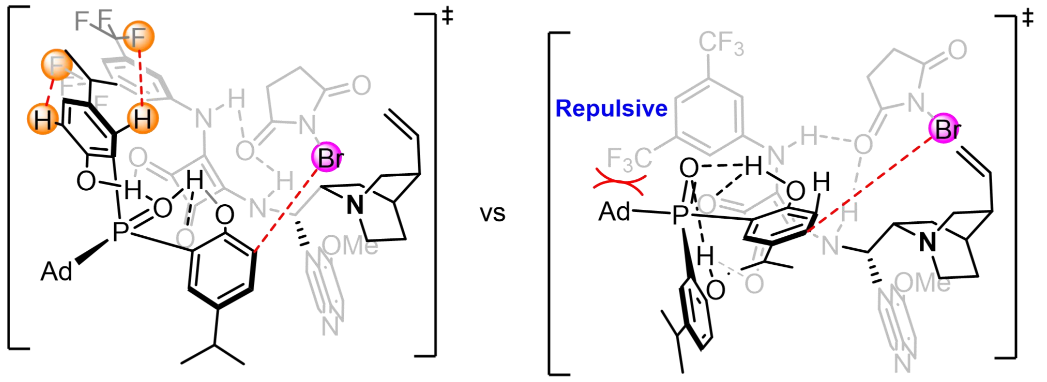

TS3 **TS4**

9. Characterization of products

(*R*)-((1*s*,3*R*,5*R*,7*S*)-Adamantan-1-yl)(3-bromo-2-hydroxy-5-methoxyphenyl)(2-hydroxy-5-methoxy

phenyl) phosphine oxide (3a)

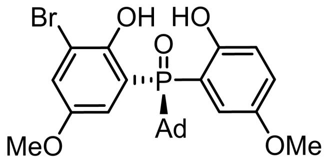

White solid, 40.6 mg, 80% yield. ^{1}H NMR (400 MHz, DMSO-d_6) δ 12.45 (s, 1H), 10.63 (s, 1H), 7.69 (dd, *J* = 13.3, 3.0 Hz, 1H), 7.44-7.37 (m, 2H), 7.08 (dd, *J* = 8.9, 3.2 Hz, 1H), 6.92 (dd, *J* = 8.8, 6.2 Hz, 1H), 3.71 (d, *J* = 4.6 Hz, 6H), 2.02-1.98 (m, 6H), 1.85-1.82 (m, 3H), 1.65 (q, *J* = 12.2 Hz, 6H). ^{13}C NMR (101 MHz, DMSO-d_6) δ 153.8 (d, *J* = 4.0 Hz), 152.7 (d, *J* = 12.1 Hz), 152.3 (d, *J* = 4.4 Hz), 151.4 (d, *J* = 15.7 Hz), 123.1, 121.1, 118.5 (d, *J* = 6.2 Hz), 118.3 (d, *J* = 9.2 Hz), 117.3 (d, *J* = 11.9 Hz), 115.4 (d, *J* = 87.1 Hz), 112.2 (d, *J* = 86.1 Hz), 111.5 (d, *J* = 11.9 Hz), 56.1 (d, *J* = 23.2 Hz), 39.3, 38.6, 36.4, 35.3, 27.5 (d, *J* = 10.9 Hz). ^{31}P NMR (162 MHz, DMSO-d_6) δ 53.0. HRMS (ESI) calculated for $[C_{24}H_{28}BrO_5P\text{-}H]^-$: 507.0765, found: 507.0758. $[\alpha]_D^{20}$= - 9.1 (c = 0.5, $CHCl_3$). HPLC separation (Chiralpak AD-H, *i*-PrOH / hexane = 1 / 5, 1.0 mL/min, 210 nm; tr (minor) = 8.6 min, tr (major) = 9.7 min, 96.5:3.5 e.r.).

(*R*)-(3-Bromo-2-hydroxy-5-methoxyphenyl)(*tert*-butyl)(2-hydroxy-5-methoxyphenyl)phosphine oxide (3b)

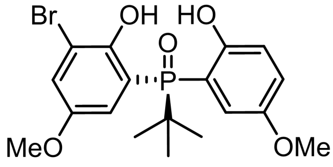

White solid, 37.4 mg, 87% yield. ^{1}H NMR (400 MHz, Chloroform-*d*) δ 11.34 (s, 1H), 10.47 (s, 1H), 7.30 (d, *J* = 2.9 Hz, 1H), 7.04 (dd, *J* = 9.1, 3.0 Hz, 1H), 6.97- 6.88 (m, 3H), 3.75 (d, *J* = 2.2 Hz, 6H), 1.34 (d, *J* = 16.3 Hz, 9H). ^{13}C NMR (101 MHz, Chloroform-*d*) δ 158.1 (d, *J* = 1.5 Hz), 154.0, 151.9 (d, *J* = 14.3 Hz), 151.7 (d, *J* = 15.9 Hz), 123.3 (d, *J* = 2.5 Hz), 120.8, 120.2 (d, *J* = 8.7 Hz), 115.7 (d, *J* = 10.4 Hz), 115.2 (d, *J* = 10.6 Hz), 113.1 (d, *J* = 11.4 Hz), 110.9 (d, *J* = 86.9 Hz), 108.7 (d, *J* = 91.0 Hz), 56.1, 55.9, 36.8 (d, *J* = 68.2 Hz), 24.3. ^{31}P NMR (162 MHz, Chloroform-*d*) δ 61.7. HRMS (ESI) calculated for $[C_{18}H_{22}BrO_5P\text{-}H]^-$: 429.0295, found: 429.0289. $[\alpha]_D^{20}$= - 13.8 (c = 0.5, $CHCl_3$). HPLC separation (Chiralpak IC, 4.6 x 250mm; *i*-PrOH / hexane = 1 / 3, 1.0 mL/min, 210 nm; tr (major) = 9.4 min, tr (minor) = 15.6 min, 93:7 e.r.).

(*R*)-(3-bromo-2-hydroxy-5-methoxyphenyl)(2-hydroxy-5-methoxyphenyl)(phenyl)phosphine oxide (3c)

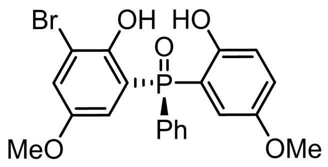

Colorless oil, 39.2 mg, 87% yield. 1H NMR (400 MHz, Chloroform-d) δ 10.17 (s, 1H), 9.73 (s, 1H), 7.67-7.62 (m, 3H), 7.54-7.49 (m, 2H), 7.32 (d, *J* = 2.9 Hz, 1H), 7.07 (dd, *J* = 9.0, 2.9 Hz, 1H), 6.97 (dd, *J* = 9.0, 5.7 Hz, 1H), 6.61 (dd, *J* = 14.3, 3.0 Hz, 1H), 6.54 (dd, *J* = 14.5, 3.0 Hz, 1H), 3.68 (d, *J* = 5.8 Hz, 6H). ^{13}C NMR (101 MHz, Chloroform-*d*) δ 153.0, 153.0, 152.4, 152.2, 133.5 (d, *J* = 2.6 Hz), 132.0, 131.9, 129.1, 129.0, 123.7 (d, *J* = 1.1 Hz), 121.4, 121.4, 121.4, 120.0 (d, *J* = 9.0 Hz), 116.4 (d, *J* = 47.4 Hz),

116.4 (d, *J* = 70.3 Hz), 56.1, 55.9. ^{31}P NMR (162 MHz, Chloroform-*d*) δ 46.8. HRMS (ESI) calculated for $[C_{20}H_{18}BrO_5P\text{-}H]^-$: 450.0055, found: 450.0054. $[\alpha]_D^{20}$ = -30.3 (c = 0.5, $CHCl_3$). HPLC separation (Chiralpak AD-H, 4.6 x 250mm; *i*-PrOH / hexane = 1 / 3, 1.0 mL/min, 210 nm; tr (major) = 8.6, tr (minor) = 9.2 min, 95:5 e.r.).

Isopropyl (*S*)-(3-bromo-2-hydroxy-5-methoxyphenyl)(2-hydroxy-5-methoxyphenyl)phosphinate (3d)

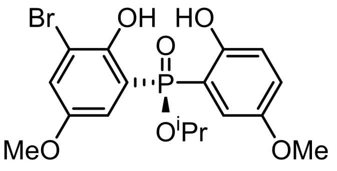

Colorless oil, 35.0 mg, 81% yield. ^{1}H NMR (400 MHz, Chloroform-*d*) δ 9.86 (s, 1H), 9.40 (s, 1H), 7.28 (d, *J* = 3.1 Hz, 1H), 7.03 (dd, *J* = 9.1, 3.0 Hz, 1H), 6.96-6.87 (m, 3H), 4.70 (dq, *J* = 12.6, 6.3 Hz, 1H), 3.74 (s, 6H), 1.40 (d, *J* = 6.2 Hz, 6H). ^{13}C NMR (101 MHz, Chloroform-*d*) δ 155.9 (d, *J* = 5.4 Hz), 152.6 (d, *J* = 2.6 Hz), 152.4 (d, *J* = 4.3 Hz), 151.7 (d, *J* = 5.6 Hz), 124.3 (d, *J* = 2.3 Hz), 122.3 (d, *J* = 2.5 Hz), 119.5 (d, *J* = 11.8 Hz), 115.8 (d, *J* = 9.0 Hz), 115.0 (d, *J* = 9.2 Hz), 113.8 (d, *J* = 136.3 Hz), 112.4 (d, *J* = 16.1 Hz), 111.3 (d, *J* = 140.4 Hz), 73.2 (d, *J* = 6.7 Hz), 56.1, 55.9, 24.2 (d, *J* = 4.3 Hz). ^{31}P NMR (162 MHz, Chloroform-*d*) δ 39.8. HRMS (ESI) calculated for $[C_{17}H_{20}BrO_6P\text{-}H]^-$: 431.0088, found: 431.0084. $[\alpha]_D^{20}$ = -33.3 (c = 0.5, $CHCl_3$). HPLC separation (Chiralpak AD-H, 4.6 x 250mm; *i*-PrOH / hexane = 1 / 3, 1.0 mL/min, 210 nm; tr (minor) = 6.9, tr (major) = 8.3 min, 98:2 e.r.).

Ethyl (*S*)-(3-bromo-2-hydroxy-5-methoxyphenyl)(2-hydroxy-5-methoxyphenyl)phosphinate (3e)

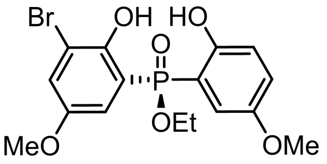

Colorless oil, 33.4 mg, 80% yield. ^{1}H NMR (400 MHz, Chloroform-*d*) δ 9.68 (s, 1H), 9.33 (s, 1H), 7.28 (d, *J* = 3.0 Hz, 1H), 7.05-7.02(m, 1H), 6.96 (dd, *J* = 14.3, 3.0 Hz, 1H), 6.93-6.88 (m, 2H), 4.22-4.14 (m, 2H), 3.75 (d, *J* = 2.0 Hz, 6H), 1.41 (t, *J* = 7.1 Hz, 3H). ^{13}C NMR (101 MHz, Chloroform-*d*) δ 156.0 (d, *J* = 5.0 Hz), 152.7, 152.6, 151.8 (d, *J* = 5.1 Hz), 124.4 (d, *J* = 1.8 Hz), 122.4 (d, *J* = 2.2 Hz), 119.5 (d, *J* = 11.2 Hz), 115.7 (d, *J* = 9.3 Hz), 114.9 (d, *J* = 8.9 Hz), 113.4 (d, *J* = 135.9 Hz), 112.5 (d, *J* = 16.1 Hz), 110.8 (d, *J* = 140.5 Hz), 63.0 (d, *J* = 6.2 Hz), 56.1, 55.9, 16.4 (d, *J* = 7.2 Hz). ^{31}P NMR (162 MHz, Chloroform-*d*) δ 40.8. HRMS (ESI) calculated for $[C_{16}H_{18}BrO_6P\text{-}H]^-$: 416.9931, found: 416.9929. $[\alpha]_D^{20}$= - 35.6 (c = 0.5, $CHCl_3$). HPLC separation (Chiralpak AD-H, 4.6 x 250mm; *i*-PrOH / hexane = 1 / 5, 1.0 mL/min, 210 nm; tr (minor) = 7.7 min, tr (major) = 8.7 min, 98.5:1.5 e.r.).

Methyl (*S*)-(3-bromo-2-hydroxy-5-methoxyphenyl)(2-hydroxy-5-methoxyphenyl)phosphinate (3f)

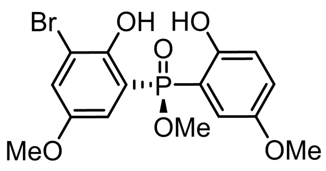

Colorless oil, 30.0 mg, 75% yield. ^{1}H NMR (400 MHz, Chloroform-*d*) δ 9.52 (s, 1H), 9.28 (s, 1H), 7.29 (d, *J* = 3.1 Hz, 1H), 7.04 (dd, *J* = 9.0, 3.1 Hz, 1H), 6.97 (dd, *J* = 14.3, 3.0 Hz, 1H), 6.92-6.88 (m, 2H), 3.83 (d, *J* = 11.9 Hz, 3H), 3.74 (d, *J* = 1.3 Hz, 6H). ^{13}C NMR (101 MHz, Chloroform-*d*)

δ 156.1 (d, J = 5.0 Hz), 152.8 (d, J = 1.2 Hz), 152.6, 151.8 (d, J = 5.7 Hz), 124.6 (d, J = 2.5 Hz), 122.6 (d, J = 2.5 Hz), 119.5 (d, J = 11.8 Hz), 115.6 (d, J = 8.7 Hz), 114.8 (d, J = 9.2 Hz), 113.1 (d, J = 104.1 Hz), 112.3 (d, J = 16.7 Hz), 110.3 (d, J = 140.6 Hz), 56.0, 55.9, 52.5 (d, J = 6.2 Hz). ^{31}P NMR (162 MHz, Chloroform-*d*) δ 42.4. HRMS (ESI) calculated for $[C_{15}H_{16}BrO_6P\text{-}H]^-$: 402.9775, found: 402.9776. $[\alpha]_D^{20}$= - 35.4 (c = 0.5, $CHCl_3$). HPLC separation (Chiralpak IC, 4.6 x 250mm; *i*-PrOH / hexane = 2 / 3, 1.0 mL/min, 210 nm; tr (minor) = 13.9 min, tr (major) = 15.3 min, 95.5:4.5 e.r.).

(*R*)-(3-bromo-2-hydroxy-5-methoxyphenyl)(2-hydroxy-5-methoxyphenyl)(methyl)phosphine oxide (3g)

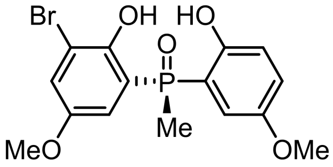

Colorless oil, 33.0 mg, 85% yield. ^{1}H NMR (400 MHz, Chloroform-*d*) δ 9.64 (s, 1H), 9.49 (s, 1H), 7.00 (dd, J = 9.1, 3.0 Hz, 1H), 6.91-6.85 (m, 3H), 6.72 (dd, J = 14.2, 3.1 Hz, 1H), 3.72 (d, J = 3.4 Hz, 6H), 2.21 (s, 3H). ^{13}C NMR (101 MHz, Chloroform-*d*) δ^{13}C NMR (101 MHz, Chloroform-*d*) δ 153.0, 152.4, 152.2, 133.5 (d, J = 2.6 Hz), 132.0, 131.9, 129.1 (d, J = 13.1 Hz), 123.7 (d, J = 1.1 Hz), 121.4, 120.0 (d, J = 9.0 Hz), 116.6 (d, J = 11.1 Hz), 116.1 (d, J = 11.7 Hz), 56.1, 55.9, 29.7. ^{31}P NMR (162 MHz, Chloroform-*d*) δ 40.5. HRMS (ESI) calculated for $[C_{15}H_{16}BrO_5P\text{-}H]^-$: 386.9825, found: 386.9827. $[\alpha]_D^{20}$= - 20.6 (c = 0.5, $CHCl_3$). HPLC separation (Chiralpak AD-H, 4.6 x 250mm; *i*-PrOH / hexane = 1/ 5, 1.0 mL/min, 210 nm; tr (major) = 11.4 min, tr (minor) = 14.3 min, 98.5:1.5 e.r.).

Ethyl (*S*)-(3-bromo-2-hydroxy-5-propylphenyl)(2-hydroxy-5-propylphenyl)phosphinate (3h)

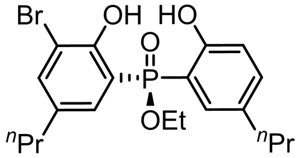

Colorless oil, 36.7 mg, 83% yield. ^{1}H NMR (400 MHz, Chloroform-*d*) δ 10.25 (s, 1H), 9.66 (s, 1H), 7.51 (d, J = 2.1 Hz, 1H), 7.24-7.16 (m, 3H), 6.88 (dd, J = 8.5, 6.3 Hz, 1H), 4.16 (p, J = 7.1 Hz, 2H), 2.49 (q, J = 7.3 Hz, 4H), 1.57 (q, J = 7.4 Hz, 4H), 1.41 (t, J = 7.0 Hz, 3H), 0.90 (t, J = 7.3 Hz, 6H). ^{13}C NMR (101 MHz, Chloroform-*d*) δ 160.1 (d, J = 6.1 Hz), 155.9 (d, J = 6.2 Hz), 138.5 (d, J = 2.2 Hz), 136.0 (d, J = 2.1 Hz), 135.3 (d, J = 13.6 Hz), 134.1 (d, J = 12.4 Hz), 130.6 (d, J = 8.5 Hz), 130.3 (d, J = 8.4 Hz), 118.3 (d, J = 10.0 Hz), 113.0 (d, J = 136.7 Hz), 112.0 (d, J = 14.5 Hz), 110.4 (d, J = 140.5 Hz), 62.8 (d, J = 6.2 Hz), 36.9, 36.6, 29.7, 24.6, 24.4, 16.4 (d, J = 6.3 Hz), 13.5 (d, J = 5.0 Hz). ^{31}P NMR (162 MHz, Chloroform-*d*) δ 42.9. HRMS (ESI) calculated for $[C_{20}H_{26}BrO_4P\text{-}H]^-$: 441.0659, found: 441.0656. $[\alpha]_D^{20}$ = - 36.4 (c = 0.5, $CHCl_3$). HPLC separation (Chiralpak AD-H, 4.6 x 250mm; *i*-PrOH / hexane = 1 / 4, 1.0 mL/min, 210 nm; tr (minor) = 9.0 min, tr (major) = 11.2 min, 97.5:2.5 e.r.).

Ethyl (*S*)-(3-bromo-2-hydroxy-5-isopropylphenyl)(2-hydroxy-5-isopropylphenyl)phosphinate (3i)

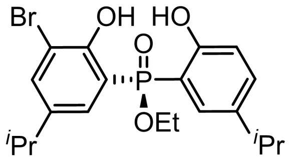

Colorless oil, 35.8 mg, 81% yield. ^{1}H NMR (400 MHz, Chloroform-*d*) δ 10.27 (s,

1H), 9.67 (s, 1H), 7.54 (d, *J* = 2.2 Hz, 1H), 7.29 (dd, *J* = 8.6, 2.3 Hz, 1H), 7.24 (d, *J* = 2.3 Hz, 1H), 7.20 (d, *J* = 2.2 Hz, 1H), 6.87 (dd, *J* = 8.6, 6.3 Hz, 1H), 4.14 (q, *J* = 7.7, 7.1 Hz, 2H), 2.85-2.77 (m, 2H), 1.39 (t, *J* = 7.1 Hz, 3H), 1.18 (dd, *J* = 6.9, 1.6 Hz, 12H). ^{13}C NMR (101 MHz, Chloroform-*d*) δ 160.2 (d, *J* = 5.6 Hz), 156.0 (d, *J* = 6.4 Hz), 141.5 (d, *J* = 13.0 Hz), 140.2 (d, *J* = 11.9 Hz), 136.8, 134.2, 128.5 (d, *J* = 8.5 Hz), 128.3 (d, *J* = 7.7 Hz), 118.4 (d, *J* = 10.9 Hz), 113.0 (d, *J* = 135.1 Hz), 112.1 (d, *J* = 13.8 Hz), 110.3 (d, *J* = 139.5 Hz), 62.8 (d, *J* = 5.6 Hz), 33.1 (d, *J* = 9.7 Hz), 24.2, 24.0, 23.8 (d, *J* = 12.3 Hz), 16.4 (d, *J* = 6.2 Hz). ^{31}P NMR (162 MHz, Chloroform-*d*) δ 43.2. HRMS (ESI) calculated for $[C_{20}H_{26}BrO_4P\text{-}H]^-$: 441.0659, found: 441.0656. $[\alpha]_D^{20}$ = -34.8 (c = 0.5, $CHCl_3$). HPLC separation (Chiralpak IC, 4.6 x 250mm; *i*-PrOH / hexane = 2 / 3, 1.0 mL/min, 210 nm; tr (minor) = 11.2 min, tr (major) = 18.3 min, 95:5 e.r.).

Ethyl (*S*)-(3-bromo-5-butyl-2-hydroxyphenyl)(5-butyl-2-hydroxyphenyl)phosphinate (3j)

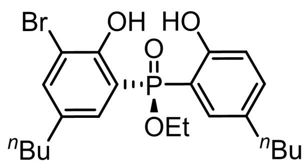

Colorless oil, 40.0 mg, 85% yield. ^{1}H NMR (400 MHz, Chloroform-*d*) δ 10.26 (s, 1H), 9.68 (s, 1H), 7.53 (d, *J* = 2.1 Hz, 1H), 7.29-7.28 (m, 1H), 7.19 (dd, *J* = 13.4, 2.2 Hz, 2H), 6.89 (dd, *J* = 8.5, 6.2 Hz, 1H), 4.22-4.14 (m, 2H), 2.53 (q, *J* = 7.2 Hz, 4H), 1.54 (p, *J* = 7.4 Hz, 4H), 1.43 (t, *J* = 7.0 Hz, 3H), 1.35-1.27 (m, 4H), 0.92 (t, *J* = 7.3 Hz, 6H). ^{13}C NMR (101 MHz, Chloroform-*d*) δ 160.1 (d, *J* = 5.5 Hz), 155.9 (d, *J* = 6.2 Hz), 138.5 (d, *J* = 2.1 Hz), 135.9 (d, *J* = 2.5 Hz), 135.5 (d, *J* = 13.1 Hz), 134.3 (d, *J* = 12.4 Hz), 130.5 (d, *J* = 8.0 Hz), 130.2 (d, *J* = 7.5 Hz), 118.3 (d, *J* = 10.5 Hz), 113.0 (d, *J* = 135.7 Hz), 112.0 (d, *J* = 13.7 Hz), 110.3 (d, *J* = 139.4 Hz), 62.8 (d, *J* = 6.2 Hz), 34.6, 34.3, 33.7, 33.5, 22.1, 22.1, 16.4 (d, *J* = 6.3 Hz), 13.9, 13.9. ^{31}P NMR (162 MHz, Chloroform-*d*) δ 43.0. HRMS (ESI) calculated for $[C_{22}H_{30}BrO_4P\text{-}H]^-$: 469.0972, found: 469.0969. $[\alpha]_D^{20}$ = - 29.6 (c = 0.5, $CHCl_3$). HPLC separation (Chiralpak AD, 4.6 x 250mm; *i*-PrOH / hexane = 1 / 9, 1.0 mL/min, 210 nm; tr (minor) = 14.4 min, tr (major) = 17.4 min, 97:3 e.r.).

Ethyl (*S*)-(3-bromo-5-(tert-butyl)-2-hydroxyphenyl)(5-(*tert*-butyl)-2-hydroxyphenyl)phosphinate (3k)

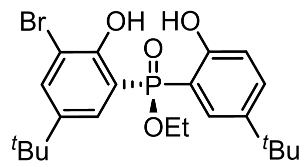

Colorless oil, 42.3 mg, 90% yield. ^{1}H NMR (400 MHz, Chloroform-*d*) δ 10.43 (s, 1H), 9.74 (s, 1H), 7.71 (d, *J* = 2.4 Hz, 1H), 7.49 (dd, *J* = 8.8, 2.5 Hz, 1H), 7.39 (dd, *J* = 13.7, 2.3 Hz, 2H), 6.91 (dd, *J* = 8.8, 6.4 Hz, 1H), 4.17 (p, *J* = 7.2 Hz, 2H), 1.41 (t, *J* = 7.0 Hz, 3H), 1.27 (d, *J* = 2.9 Hz, 18H). ^{13}C NMR (101 MHz, Chloroform-*d*) δ 160.0 (d, *J* = 5.5 Hz), 155.9 (d, *J* = 6.5 Hz), 143.9 (d, *J* = 12.2 Hz), 142.6 (d, *J* = 11.9 Hz), 136.0, 133.2, 127.3 (d, *J* = 8.6 Hz), 127.1 (d, *J* = 7.7 Hz), 118.2 (d, *J* = 10.4 Hz), 112.6 (d, *J* = 95.3 Hz), 111.9 (d, *J* = 25.9 Hz), 109.7 (d, *J* = 139.6 Hz), 62.8 (d, *J* = 5.7 Hz), 34.2 (d, *J* = 11.9 Hz), 31.3, 31.2, 29.7, 16.4 (d, *J* = 6.5 Hz). ^{31}P NMR (162 MHz, Chloroform-*d*) δ 43.8. HRMS (ESI) calculated for $[C_{22}H_{30}BrO_4P\text{-}H]^-$:

469.0972, found: 469.0966. $[\alpha]_D^{20}$ = -22.0 (c = 0.5, $CHCl_3$). HPLC separation (Chiralpak IC, 4.6 x 250mm; *i*-PrOH / hexane = 1 / 5, 1.0 mL/min, 210 nm; tr (minor) = 14.0 min, tr (major) = 23.6 min, 90.5:9.5 e.r.).

Ethyl (*S*)-(3-bromo-5-cyclohexyl-2-hydroxyphenyl)(5-cyclohexyl-2-hydroxyphenyl)phosphinate (3l)

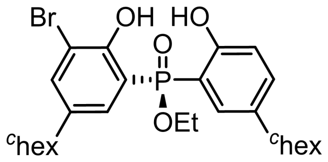

Colorless oil, 41.8 mg, 80% yield. ^{1}H NMR (400 MHz, Chloroform-*d*) δ 10.28 (s, 1H), 9.67 (s, 1H), 7.54 (d, *J* = 2.1 Hz, 1H), 7.30-7.29 (m, 1H), 7.24 (d, *J* = 12.8 Hz, 2H), 6.88 (dd, *J* = 8.6, 6.3 Hz, 1H), 4.22-4.13 (m, 2H), 2.43-2.42 (m, 2H), 1.79 (dd, *J* = 30.0, 11.1 Hz, 10H), 1.41 (t, *J* = 7.0 Hz, 3H), 1.37-1.29 (m, 6H), 1.23-1.19 (m, 1H), 0.94-0.86 (m, 1H). ^{13}C NMR (101 MHz, Chloroform-*d*) δ 160.2 (d, *J* = 6.0 Hz), 156.0 (d, *J* = 6.5 Hz), 140.7 (d, *J* = 12.9 Hz), 139.5 (d, *J* = 11.9 Hz), 137.1, 134.5, 128.9 (d, *J* = 8.4 Hz), 128.7 (d, *J* = 7.7 Hz), 118.2 (d, *J* = 9.9 Hz), 112.9 (d, *J* = 135.8 Hz), 112.0 (d, *J* = 14.0 Hz), 110.3 (d, *J* = 139.6 Hz), 62.8 (d, *J* = 6.4 Hz), 43.4, 43.2, 34.7 (d, *J* = 10.7 Hz), 34.4 (d, *J* = 14.9 Hz), 26.8, 26.7, 26.0, 25.9, 16.4 (d, *J* = 6.4 Hz). ^{31}P NMR (162 MHz, Chloroform-*d*) δ 43.2. HRMS (ESI) calculated for $[C_{26}H_{34}BrO_4P\text{-}H]^-$: 521.1285, found: 521.1286. $[\alpha]_D^{20}$ = -18.4 (c = 0.5, $CHCl_3$). HPLC separation (Chiralpak AD-H, 4.6 x 250mm; *i*-PrOH / hexane = 1 / 9, 1.0 mL/min, 210 nm; tr (minor) = 15.2 min, tr (major) = 18.3 min, 96:4 e.r.).

Ethyl (*S*)-(5-bromo-4-hydroxy-[1,1'-biphenyl]-3-yl)(4-hydroxy-[1,1'-biphenyl]-3-yl)phosphinate (3m)

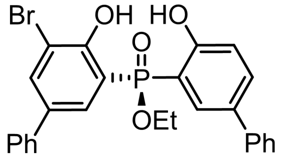

Colorless oil, 42.3 mg, 83% yield. ^{1}H NMR (400 MHz, Chloroform-*d*) δ 10.23 (s, 1H), 9.89 (s, 1H), 7.94 (d, *J* = 2.2 Hz, 1H), 7.72-7.67 (m, 3H), 7.50-7.41 (m, 8H), 7.38-7.33 (m, 2H), 7.07 (dd, *J* = 8.9, 6.2 Hz, 1H), 4.29-4.22 (m, 2H), 1.45 (t, *J* = 7.1 Hz, 3H). ^{13}C NMR (101 MHz, Chloroform-*d*) δ 161.5 (d, *J* = 5.7 Hz), 157.1 (d, *J* = 6.1 Hz), 139.7, 138.5, 137.1 (d, *J* = 2.5 Hz), 134.7, 134.5 (d, *J* = 2.8 Hz), 133.4 (d, *J* = 12.7 Hz), 129.8 (d, *J* = 8.4 Hz), 129.3 (d, *J* = 8.0 Hz), 129.1, 129.0, 127.8, 127.4, 126.7, 126.7, 119.0 (d, *J* = 10.2 Hz), 113.8 (d, *J* = 136.4 Hz), 112.8 (d, *J* = 13.7 Hz), 111.3 (d, *J* = 140.0 Hz), 63.1 (d, *J* = 6.2 Hz), 16.5 (d, *J* = 6.5 Hz). ^{31}P NMR (162 MHz, Chloroform-*d*) δ 41.7. HRMS (ESI) calculated for $[C_{26}H_{22}BrO_4P\text{-}H]^-$: 509.0346, found: 509.0346. $[\alpha]_D^{20}$ = -42.8 (c = 0.5, $CHCl_3$). HPLC separation (Chiralpak AD-H, 4.6 x 250mm; 25% *i*-PrOH / hexane = 1 / 4, 1.0 mL/min, 210 nm; tr (minor) = 7.5 min, tr (major) = 8.6 min, 95:5 e.r.).

Ethyl (*S*)-(3-bromo-5-ethoxy-2-hydroxyphenyl)(5-ethoxy-2-hydroxyphenyl)phosphinate (3n)

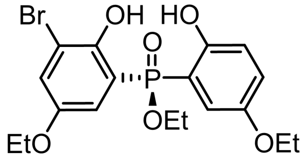

Colorless oil, 40.6 mg, 91% yield. ^{1}H NMR (400 MHz, Chloroform-*d*) δ 9.70 (s, 1H), 9.34 (s, 1H), 7.28 (d, *J* = 3.0 Hz, 1H), 7.03 (dd, *J* = 9.0, 3.1 Hz, 1H), 6.97-6.87 (m, 3H), 4.17 (p, *J* = 7.3 Hz, 2H), 3.94 (qd, *J* = 7.0, 2.8 Hz, 4H), 1.42-1.36 (m, 9H). ^{13}C NMR (101 MHz, Chloroform-*d*) δ 155.9 (d, *J* = 5.4 Hz),

152.0 (d, J = 1.6 Hz), 151.9, 151.7 (d, J = 5.9 Hz), 124.9 (d, J = 2.4 Hz), 122.9 (d, J = 2.8 Hz), 119.4 (d, J = 11.8 Hz), 116.5 (d, J = 8.7 Hz), 115.8 (d, J = 9.2 Hz), 114.0, 112.4 (d, J = 16.1 Hz), 110.7 (d, J = 139.9 Hz), 64.6, 64.3, 62.9 (d, J = 6.2 Hz), 29.7, 16.4 (d, J = 6.8 Hz), 14.8. ^{31}P NMR (162 MHz, Chloroform-*d*) δ 41.1. HRMS (ESI) calculated for $[C_{18}H_{22}BrO_6P\text{-}H]^-$: 445.0244, found: 445.0242. $[\alpha]_D^{20}$ = - 28.4 (c = 0.5, $CHCl_3$). HPLC separation (Chiralpak AD, 4.6 x 250mm; *i*-PrOH / hexane = 1 / 4, 1.0 mL/min, 210 nm; tr (minor) = 9.6 min, tr (major) = 11.2 min, 97:3 e.r.).

Ethyl (*S*)-(3-bromo-2-hydroxy-5-propoxyphenyl)(2-hydroxy-5-propoxyphenyl)phosphinate (3o)

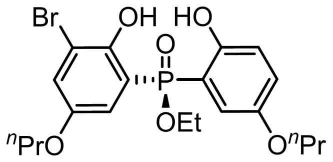

Colorless oil, 38.4 mg, 81% yield. ^{1}H NMR (400 MHz, DMSO-d_6) δ 11.09 (s, 1H), 10.16 (s, 1H), 7.38 (d, J = 3.0 Hz, 1H), 7.22 (dd, J = 14.3, 3.2 Hz, 1H), 7.10-7.03 (m, 2H), 6.81 (dd, J = 8.9, 7.2 Hz, 1H), 4.12-3.98 (m, 2H), 3.89-3.81 (m, 4H), 1.68 (dq, J = 18.9, 6.9 Hz, 4H), 1.31 (t, J = 7.0 Hz, 3H), 0.95 (dt, J = 15.8, 7.4 Hz, 6H). ^{13}C NMR (101 MHz, DMSO-d_6) δ 154.1 (d, J = 4.4 Hz), 152.3 (d, J = 6.0 Hz), 151.7 (d, J = 1.7 Hz), 151.5, 124.4 (d, J = 2.1 Hz), 122.4 (d, J = 1.9 Hz), 118.0 (d, J = 10.7 Hz), 117.8, 117.0 (d, J = 9.9 Hz), 116.2 (d, J = 42.9 Hz), 114.8 (d, J = 32.2 Hz), 111.2 (d, J = 16.1 Hz), 70.3, 70.1, 62.1 (d, J = 5.9 Hz), 22.6, 22.4, 16.7 (d, J = 6.3 Hz), 10.9, 10.8. ^{31}P NMR (162 MHz, DMSO-d_6) δ 35.3. HRMS (ESI) calculated for $[C_{20}H_{26}BrO_6P\text{-}H]^-$: 473.0557, found: 473.0557. $[\alpha]_D^{20}$ = - 22.4 (c = 0.5, $CHCl_3$). HPLC separation (Chiralpak AD-H, 4.6 x 250mm; *i*-PrOH / hexane = 1 / 4, 1.0 mL/min, 210 nm; tr (minor) = 6.2 min, tr (major) = 7.3 min, 96:4 e.r.).

Ethyl (*S*)-(3-bromo-2-hydroxy-5-isopropoxyphenyl)(2-hydroxy-5-isopropoxyphenyl)phosphinate (3p)

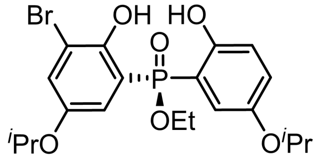

Colorless oil, 39.3 mg, 83% yield. ^{1}H NMR (400 MHz, Chloroform-*d*) δ 9.75 (s, 1H), 9.37 (s, 1H), 7.28 (d, J = 2.9 Hz, 1H), 7.03 (dd, J = 9.0, 3.0 Hz, 1H), 6.96-6.86 (m, 3H), 4.37 (dtd, J = 11.9, 5.9, 1.5 Hz, 2H), 4.21-4.13 (m, 2H), 1.41 (t, J = 7.1 Hz, 3H), 1.28 (d, J = 6.0 Hz, 12H). ^{13}C NMR (101 MHz, Chloroform-*d*) δ 156.1 (d, J = 5.5 Hz), 151.9 (d, J = 6.2 Hz), 150.8 (d, J = 8.7 Hz), 150.7 (d, J = 7.5 Hz), 126.9, 124.8, 119.4 (d, J = 12.0 Hz), 118.3 (d, J = 7.8 Hz), 118.1 (d, J = 8.7 Hz), 113.3 (d, J = 136.2 Hz), 112.4 (d, J = 16.1 Hz), 110.8 (d, J = 139.6 Hz), 71.7, 71.4, 62.9 (d, J = 6.4 Hz), 29.7, 22.0 (d, J = 9.5 Hz), 16.4 (d, J = 6.5 Hz). ^{31}P NMR (162 MHz, Chloroform-*d*) δ 41.1. HRMS (ESI) calculated for $[C_{20}H_{26}BrO_6P\text{-}H]^-$: 473.0557, found: 473.0558. $[\alpha]_D^{20}$ = - 27.2 (c = 0.5, $CHCl_3$). HPLC separation (Chiralpak AD, 4.6 x 250mm; *i*-PrOH / hexane = 1 / 4, 1.0 mL/min, 210 nm; tr (minor) = 6.4 min, tr (major) = 7.1 min, 98.5:1.5 e.r.).

Ethyl (*S*)-(3-bromo-2-hydroxy-5-phenoxyphenyl)(2-hydroxy-5-phenoxyphenyl)phosphinate (3q)

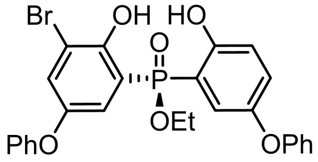

Colorless oil, 49.9 mg, 92% yield. ^{1}H NMR (400 MHz, Chloroform-*d*) δ 9.94 (s, 1H), 9.60 (s, 1H), 7.35 (d, *J* = 2.9 Hz, 1H), 7.24-7.21 (m, 4H), 7.11-6.99 (m, 5H), 6.92-6.84 (m, 5H), 4.11 (q, *J* = 7.4 Hz, 2H), 1.31 (t, *J* = 7.1 Hz, 3H). ^{13}C NMR (101 MHz, Chloroform-*d*) δ 158.2 (d, *J* = 5.0 Hz), 157.8, 157.2, 154.1 (d, *J* = 5.6 Hz), 149.5 (d, *J* = 18.3 Hz), 149.2 (d, *J* = 16.3 Hz), 130.0, 129.9, 129.8, 127.7, 123.7, 123.1, 121.6 (d, *J* = 8.0 Hz), 121.1 (d, *J* = 7.9 Hz), 119.9 (d, *J* = 11.7 Hz), 118.0, 117.6, 113.6 (d, *J* = 136.8 Hz), 112.8 (d, *J* = 15.3 Hz), 111.5 (d, *J* = 140.7 Hz), 63.2 (d, *J* = 6.4 Hz), 16.3 (d, *J* = 6.5 Hz). ^{31}P NMR (162 MHz, Chloroform-*d*) δ 39.8. HRMS (ESI) calculated for $[C_{26}H_{22}BrO_6P\text{-}H]^-$: 541.0244, found: 541.0248. $[\alpha]_D^{20}$ = -21.4 (c = 0.5, $CHCl_3$). HPLC separation (Chiralpak AD-H, 4.6 x 250mm; *i*-PrOH / hexane = 1 / 3, 1.0 mL/min, 210 nm; tr (major) = 13.1 min, tr (minor) = 18.1 min, 97:3 e.r.).

Ethyl (*S*)-(3-bromo-4-chloro-2-hydroxy-5-methylphenyl)(4-chloro-2-hydroxy-5-methylphenyl) phosphinate (3r)

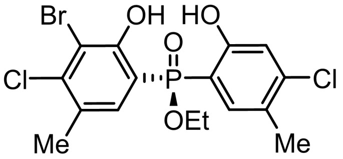

Colorless oil, 38.1 mg, 84% yield. ^{1}H NMR (400 MHz, Chloroform-*d*) δ 11.28 (s, 1H), 10.05 (s, 1H), 7.62 (s, 1H), 7.31 (d, *J* = 8.5 Hz, 1H), 6.84 (dd, *J* = 8.6, 5.4 Hz, 1H), 4.33-4.25 (m, 2H), 2.22 (s, 6H), 1.49 (t, *J* = 7.1 Hz, 3H). ^{13}C NMR (101 MHz, Chloroform-*d*) δ 161.1, 157.6 (d, *J* = 4.4 Hz), 139.7, 137.2, 135.9 (d, *J* = 5.3 Hz), 135.2 (d, *J* = 4.9 Hz), 129.2 (d, *J* = 8.9 Hz), 128.4 (d, *J* = 8.7 Hz), 116.8 (d, *J* = 10.8 Hz), 112.3 (d, *J* = 140.6 Hz), 110.7 (d, *J* = 147.1 Hz), 110.4 (d, *J* = 13.8 Hz), 64.1 (d, *J* = 5.5 Hz), 29.7, 19.4 (d, *J* = 12.4 Hz), 16.1 (d, *J* = 6.5 Hz). ^{31}P NMR (162 MHz, Chloroform-*d*) δ 41.0. HRMS (ESI) calculated for $[C_{16}H_{16}BrCl_2O4P\text{-}H]^-$: 452.9253, found: 452.9255. $[\alpha]_D^{20}$ = - 27.2 (c = 0.5, $CHCl_3$). HPLC separation (Chiralpak IC, 4.6 x 250mm; *i*-PrOH / hexane = 1 / 5, 1.0 mL/min, 210 nm; tr (minor) = 13.2 min, tr (major) = 21.0min, 91:9 e.r.).

(*R*)-((1*s*,3*R*,5*R*,7*S*)-Adamantan-1-yl)(3-bromo-2-hydroxy-5-methylphenyl)(2-hydroxy-5-methylphenyl) phosphine oxide (3s)

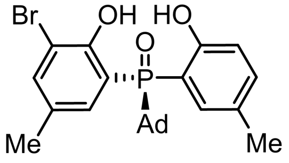

White solid, 40.5 mg, 85% yield. ^{1}H NMR (400 MHz, Chloroform-*d*) δ 11.79 (s, 1H), 10.87 (s, 1H), 7.52 (s, 1H), 7.23 (d, *J* = 8.6 Hz, 1H), 7.19-7.13 (m, 2H), 6.85 (dd, *J* = 8.5, 4.7 Hz, 1H), 2.31-2.30 (m, 6H), 2.06 (s, 3H), 1.99-1.97 (m, 6H), 1.72 (q, *J* = 12.4 Hz, 6H). ^{13}C NMR (101 MHz, Chloroform-*d*) δ 162.3, 158.1 (d, *J* = 2.2 Hz), 138.4, 135.6, 130.1 (d, *J* = 8.8 Hz), 129.8 (d, *J* = 8.7 Hz), 129.1 (d, *J* = 12.1 Hz), 128.1 (d, *J* = 11.9 Hz), 119.2 (d, *J* = 8.3 Hz), 112.8 (d, *J* = 10.5 Hz), 110.1 (d, *J* = 86.6 Hz), 107.7 (d, *J* = 90.5 Hz),

39.7 (d, J = 69.8 Hz), 36.2, 34.3, 27.3, 27.2, 20.7 (d, J = 30.1 Hz). ^{31}P NMR (162 MHz, Chloroform-*d*) δ 57.0. HRMS (ESI) calculated for $[C_{24}H_{28}BrO_3P-H]^-$: 475.0866, found: 475.0866. $[\alpha]_D^{20}$= - 10.7 (c = 0.5, $CHCl_3$). HPLC separation (Chiralpak AD-H, 4.6 x 250mm; *i*-PrOH / hexane = 1 / 15, 1.0 mL/min, 210 nm; tr (minor) = 7.8 min, tr (major) = 10.2 min, 95:5 e.r.).

(*R*)-((1*s*,3*R*,5*R*,7*S*)-Adamantan-1-yl)(3-bromo-5-ethyl-2-hydroxyphenyl)(5-ethyl-2-hydroxyphenyl) phosphine oxide (3t)

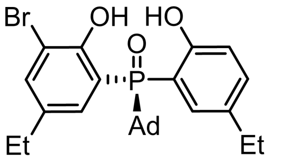

White solid, 40.8 mg, 81% yield. ^{1}H NMR (400 MHz, Chloroform-*d*) δ 11.82 (s, 1H), 10.88 (s, 1H), 7.56 (s, 1H), 7.28-7.27 (m, 1H), 7.25-7.19 (m, 2H), 6.88 (dd, J = 8.5, 4.7 Hz, 1H), 2.61 (dt, J = 13.3, 7.3 Hz, 4H), 2.07 (s, 3H), 2.01-1.98 (m, 6H), 1.73 (q, J = 12.2 Hz, 6H), 1.22 (t, J = 7.6 Hz, 6H). ^{13}C NMR (101 MHz, Chloroform-*d*) δ 162.4 (d, J = 1.9 Hz), 158.2 (d, J = 2.6 Hz), 137.3 (d, J = 2.5 Hz), 135.5 (d, J = 11.4 Hz), 134.5 (d, J = 2.5 Hz), 134.4, 129.0 (d, J = 9.2 Hz), 128.7 (d, J = 8.9 Hz), 119.2 (d, J = 8.0 Hz), 112.8 (d, J = 9.9 Hz), 110.1 (d, J = 86.9 Hz), 107.7 (d, J = 90.8 Hz), 39.6 (d, J = 69.4 Hz), 36.2 (d, J = 1.5 Hz), 34.4 (d, J = 1.8 Hz), 28.0, 27.8, 27.3, 27.2, 15.6 (d, J = 20.5 Hz). ^{31}P NMR (162 MHz, Chloroform-*d*) δ 57.1. HRMS (ESI) calculated for $[C_{26}H_{32}BrO_3P-H]^-$: 503.1179, found: 503.1185. $[\alpha]_D^{20}$= - 22.3 (c = 0.5, $CHCl_3$). HPLC separation (Chiralpak IC, 4.6 x 250mm; *i*-PrOH / hexane = 1 / 5, 1.0 mL/min, 210 nm; tr (major) = 9.1 min, tr (minor) = 13.0 min, 96:4 e.r.).

(*R*)-((1*s*,3*R*,5*R*,7*S*)-Adamantan-1-yl)(3-bromo-2-hydroxy-5-propylphenyl)(2-hydroxy-5-propylphenyl) phosphine oxide (3u)

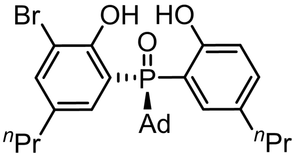

White solid, 37.8 mg, 71% yield. ^{1}H NMR (400 MHz, Chloroform-*d*) δ 11.86 (s, 1H), 10.92 (s, 1H), 7.57 (d, J = 2.0 Hz, 1H), 7.31-7.27 (m, 1H), 7.25-7.21 (m, 2H), 6.91 (dd, J = 8.5, 4.7 Hz, 1H), 2.57 (q, J = 7.3 Hz, 4H), 2.10 (s, 3H), 2.04 (d, J = 6.0 Hz, 6H), 1.77 (q, J = 12.2 Hz, 6H), 1.67 – 1.62 (m, 4H), 0.97 (t, J = 7.3 Hz, 6H). ^{13}C NMR (101 MHz, Chloroform-*d*) δ 162.5, 158.3 (d, J = 2.2 Hz), 137.8, 135.0, 133.9 (d, J = 11.9 Hz), 132.9 (d, J = 10.9 Hz), 129.8 (d, J = 8.8 Hz), 129.4 (d, J = 9.4 Hz), 119.2 (d, J = 7.7 Hz), 112.8 (d, J = 9.8 Hz), 110.0 (d, J = 87.1 Hz), 107.6 (d, J = 91.4 Hz), 39.7 (d, J = 68.8 Hz), 37.1, 36.8, 36.2, 34.4, 27.3, 27.2, 24.5 (d, J = 14.2 Hz), 13.6, 13.5. ^{31}P NMR (162 MHz, Chloroform-*d*) δ 57.0. HRMS (ESI) calculated for $[C_{28}H_{36}BrO_3P-H]^-$: 531.1492, found: 531.1491. $[\alpha]_D^{20}$= - 11.8 (c = 0.5, $CHCl_3$). HPLC separation (Chiralpak IC, 4.6 x 250mm; *i*-PrOH / hexane = 1 / 5, 1.0 mL/min, 210 nm; tr (major) = 8.1 min, tr (minor) = 12.0 min, 90:10 e.r.).

(*R*)-((1*s*,3*R*,5*R*,7*S*)-Adamantan-1-yl)(3-bromo-5-butyl-2-hydroxyphenyl)(5-butyl-2-hydroxyphenyl) phosphine oxide (3v)

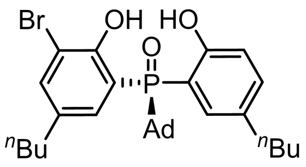

White solid, 47.6 mg, 85% yield. ^{1}H NMR (400 MHz, Chloroform-*d*) δ 11.82 (s, 1H), 10.87 (s, 1H), 7.53 (s, 1H), 7.25-7.16 (m, 3H), 6.87 (dd, *J* = 8.5, 4.7 Hz, 1H), 2.55 (q, *J* = 7.4 Hz, 4H), 2.06 (s, 3H), 1.98 (s, 6H), 1.72 (q, *J* = 12.5 Hz, 6H), 1.56 (p, *J* = 7.5 Hz, 4H), 1.33 (dq, *J* = 14.7, 7.3 Hz, 4H), 0.92 (t, *J* = 7.3 Hz, 6H). ^{13}C NMR (101 MHz, Chloroform-*d*) δ 162.4 (d, *J* = 1.8 Hz), 158.2 (d, *J* = 2.8 Hz), 137.8 (d, *J* = 2.5 Hz), 134.9 (d, *J* = 2.5 Hz), 134.1 (d, *J* = 11.7 Hz), 133.0 (d, *J* = 11.2 Hz), 129.7 (d, *J* = 9.1 Hz), 129.3 (d, *J* = 9.0 Hz), 119.2 (d, *J* = 8.0 Hz), 112.8 (d, *J* = 10.0 Hz), 110.0 (d, *J* = 87.0 Hz), 107.6 (d, *J* = 90.8 Hz), 39.7 (d, *J* = 69.1 Hz), 36.2 (d, *J* = 1.8 Hz), 34.7, 34.4, 34.4 (d, *J* = 1.8 Hz), 33.6, 33.4, 29.7, 27.2 (d, *J* = 10.6 Hz), 22.05 (d, *J* = 2.5 Hz), 13.94, 13.89. ^{31}P NMR (162 MHz, Chloroform-*d*) δ 57.0. HRMS (ESI) calculated for $[C_{30}H_{40}BrO_3P-H]^-$: 559.1805, found: 559.1808. $[\alpha]_D^{20}$= - 9.0 (c = 0.5, $CHCl_3$). HPLC separation (Chiralpak IC, 4.6 x 250mm; *i*-PrOH / hexane = 1 / 15, 1.0 mL/min, 210 nm; tr (major) = 12.1 min, tr (minor) = 16.5 min, 90:10 e.r.).

(*R*)-((1*s*,3*R*,5*R*,7*S*)-Adamantan-1-yl)(3-bromo-2-hydroxy-5-isopropylphenyl)(2-hydroxy-5-isopropyl phenyl)phosphine oxide (3w)

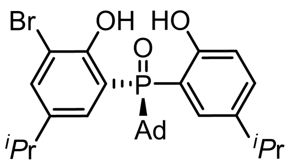

White solid, 44.2 mg, 83% yield. ^{1}H NMR (400 MHz, Chloroform-*d*) δ 11.77 (s, 1H), 10.82 (s, 1H), 7.55 (d, *J* = 2.1 Hz, 1H), 7.28 (d, *J* = 2.1 Hz, 1H), 7.23 (ddd, *J* = 7.0, 4.1, 2.1 Hz, 2H), 6.85 (dd, *J* = 8.4, 4.7 Hz, 1H), 2.83 (dq, *J* = 14.1, 7.1 Hz, 2H), 2.03 (s, 3H), 1.96 (s, 6H), 1.69 (q, *J* = 12.7 Hz, 6H), 1.21 (d, *J* = 6.9 Hz, 12H). ^{13}C NMR (101 MHz, Chloroform-*d*) δ 162.5 (d, *J* = 1.5 Hz), 158.3 (d, *J* = 2.6 Hz), 140.1 (d, *J* = 11.0 Hz), 139.1(d, *J* = 10.8 Hz), 136.1, 133.2, 127.5 (d, *J* = 9.5 Hz), 127.2 (d, *J* = 8.9 Hz), 119.1 (d, *J* = 7.7 Hz), 112.8 (d, *J* = 9.9 Hz), 110.1 (d, *J* = 87.2 Hz), 107.7 (d, *J* = 90.7 Hz), 39.6 (d, *J* = 69.7 Hz), 36.2, 34.4, 33.1 (d, *J* = 12.0 Hz), 29.7, 27.3 (d, *J* = 10.9 Hz), 24.1, 23.9. ^{31}P NMR (162 MHz, Chloroform-*d*) δ 57.2. HRMS (ESI) calculated for $[C_{28}H_{36}BrO_3P-H]^-$: 531.1492, found: 531.1494. $[\alpha]_D^{20}$= - 17.3 (c = 0.5, $CHCl_3$). HPLC separation (Chiralpak IC, 4.6 x 250mm; *i*-PrOH / hexane = 1 / 15, 1.0 mL/min, 210 nm; tr (major) = 9.6 min, tr (minor) = 11.7 min, 90:10 e.r.).

(*R*)-((1*s*,3*R*,5*R*,7*S*)-Adamantan-1-yl)(3-bromo-5-ethoxy-2-hydroxyphenyl)(5-ethoxy-2-hydroxyphenyl) phosphine oxide (3x)

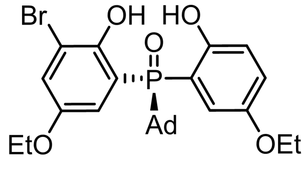

White solid, 44.5 mg, 83% yield. ^{1}H NMR (400 MHz, Chloroform-*d*) δ 11.42 (s, 1H), 10.56 (s, 1H), 7.29 (d, *J* = 2.9 Hz, 1H), 7.02 (dd, *J* = 9.1, 2.9 Hz, 1H),

6.95-6.85 (m, 3H), 3.99-3.94 (m, 4H), 2.05 (s, 3H), 1.99 (s, 6H), 1.71 (q, *J* = 12.3 Hz, 6H), 1.39 (td, *J* = 7.0, 2.7 Hz, 6H). ^{13}C NMR (101 MHz, Chloroform-*d*) δ 158.3 (d, *J* = 1.8 Hz), 154.1 (d, *J* = 2.4 Hz), 151.1 (d, *J* = 12.6 Hz), 150.9, 123.9 (d, *J* = 2.1 Hz), 121.4 (d, *J* = 2.5 Hz), 120.0 (d, *J* = 8.7 Hz), 116.4 (d, *J* = 10.0 Hz), 116.0 (d, *J* = 10.3 Hz), 113.0 (d, *J* = 11.8 Hz), 110.2 (d, *J* = 86.5 Hz), 107.9 (d, *J* = 90.8 Hz), 64.7, 64.4, 39.7 (d, *J* = 69.6 Hz), 36.1 (d, *J* = 1.8 Hz), 34.4 (d, *J* = 1.9 Hz), 27.2 (d, *J* = 10.6 Hz), 14.9, 14.8. ^{31}P NMR (162 MHz, Chloroform-*d*) δ 56.4. HRMS (ESI) calculated for $[C_{26}H_{32}BrO_5P\text{-}H]^-$: 535.1078, found: 535.1077. $[\alpha]_D^{20}$ = -11.5 (c = 0.5, $CHCl_3$). HPLC separation (Chiralpak IC, 4.6 x 250mm; *i*-PrOH / hexane = 1 / 5, 1.0 mL/min, 210 nm; tr (major) = 11.1 min, tr (minor) = 15.2 min, 93:7 e.r.).

(*R*)-((1*s*,3*R*,5*R*,7*S*)-adamantan-1-yl)(3-bromo-2-hydroxy-5-iodophenyl)(2-hydroxy-5-iodophenyl) phosphine oxide (3y)

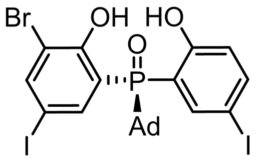

White solid, 45.4 mg, 65% yield. ^{1}H NMR (400 MHz, Chloroform-*d*) δ 11.32 (s, 1H), 10.58 (s, 1H), 7.16 (d, *J* = 2.9 Hz, 1H), 7.06 (dd, *J* = 9.1, 3.0 Hz, 1H), 6.98-6.87 (m, 3H), 2.07 (s, 3H), 2.03 (d, *J* = 4.8 Hz, 6H), 1.74 (q, *J* = 12.6 Hz, 6H). ^{13}C NMR (101 MHz, Chloroform-*d*) δ 158.5, 153.7, 152.0 (d, *J* = 14.3 Hz), 151.6 (d, *J* = 15.7 Hz), 124.0 (d, *J* = 12.3 Hz), 120.7, 120.3, 120.2, 115.5 (d, *J* = 10.2 Hz), 115.1 (d, *J* = 10.0 Hz), 110.6 (d, *J* = 87.3 Hz), 108.2 (d, *J* = 90.8 Hz), 39.9 (d, *J* = 69.6 Hz), 36.3 (d, *J* = 1.8 Hz), 34.6 (d, *J* = 2.0 Hz), 27.4 (d, *J* = 10.6 Hz). ^{31}P NMR (162 MHz, Chloroform-*d*) δ 54.5. HRMS (ESI) calculated for $[C_{22}H_{22}BrI_2O_3P\text{-}H]^-$: 698.8486, found: 698.8497. $[\alpha]_D^{20}$= - 13.6 (c = 0.5, $CHCl_3$). HPLC separation (Chiralpak IC, 4.6 x 250mm; *i*-PrOH / hexane = 1 / 5, 1.0 mL/min, 210 nm; tr (major) = 7.4 min, tr (minor) = 8.2 min, 81:19 e.r.).

(*R*)-((1*s*,3*R*,5*R*,7*S*)-Adamantan-1-yl)(3-bromo-2-hydroxy-4,5-dimethylphenyl)(2-hydroxy-4,5-dimethyl phenyl)phosphine oxide (3z)

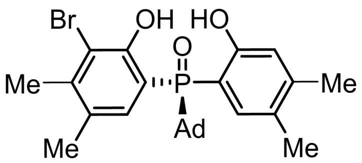

White solid, 44.9 mg, 89% yield. ^{1}H NMR (400 MHz, Chloroform-*d*) δ 11.83 (s, 1H), 10.82 (s, 1H), 7.14 (dd, *J* = 10.8, 7.4 Hz, 2H), 6.73 (d, *J* = 4.6 Hz, 1H), 2.40 (s, 3H), 2.30 (s, 3H), 2.22 (s, 6H), 2.04 (s, 3H), 1.99-1.98 (m, 6H), 1.76-1.67 (m, 6H). ^{13}C NMR (101 MHz, Chloroform-*d*) δ 162.5, 158.4 (d, *J* = 2.1 Hz), 144.3 (d, *J* = 2.4 Hz), 143.4 (d, *J* = 2.2 Hz), 130.5 (d, *J* = 8.9 Hz), 129.6 (d, *J* = 8.8 Hz), 127.9 (d, *J* = 12.4 Hz), 127.3 (d, *J* = 11.0 Hz), 120.0 (d, *J* = 7.7 Hz), 116.2 (d, *J* = 10.6 Hz), 107.1 (d, *J* = 89.5 Hz), 105.2 (d, *J* = 93.6 Hz), 39.6 (d, *J* = 69.9 Hz), 36.2, 34.3, 27.3 (d, *J* = 10.8 Hz), 21.0, 20.5, 20.2, 19.3. ^{31}P NMR (162 MHz, Chloroform-*d*) δ 56.6. HRMS (ESI) calculated for $[C_{26}H_{32}BrO_3P\text{-}H]^-$: 503.1179, found: 503.1185. $[\alpha]_D^{20}$= - 16.6 (c = 0.5, $CHCl_3$). HPLC separation (Chiralpak IC, 4.6 x 250mm; *i*-PrOH / hexane = 1 /

5, 1.0 mL/min, 210 nm; tr (minor) = 8.1 min, tr (major) = 8.9 min, 95.5:4.5 e.r.).

(*R*)-((1s,3R,5R,7S)-adamantan-1-yl)(2-hydroxy-5-methoxy-[1,1'-biphenyl]-3-yl)(2-hydroxy-5-methoxy phenyl)phosphine oxide (5)

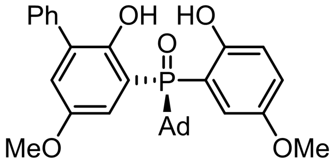

Colorless oil, 43.8 mg, 87% yield. ^{1}H NMR (400 MHz, Chloroform-d) δ 11.02 (s, 1H), 10.74 (s, 1H), 7.56 (d, *J* = 7.1 Hz, 2H), 7.42 (t, *J* = 7.5 Hz, 2H), 7.34 (t, *J* = 7.3 Hz, 1H), 7.09-6.89 (m, 5H), 3.80 (d, *J* = 3.7 Hz, 6H), 2.06 (d, *J* = 5.4 Hz, 9H), 1.73 (t, *J* = 9.3 Hz, 6H). ^{13}C NMR (101 MHz, Chloroform-*d*) δ 158.4, 155.3, 151.8 (d, *J* = 14.1 Hz), 151.5 (d, *J* = 15.4 Hz), 137.7 (d, *J* = 30.4 Hz), 132.5 (d, *J* = 9.1 Hz), 129.3 (d, *J* = 42.3 Hz), 128.2 (d, *J* = 9.9 Hz), 127.5, 125.3, 120.8 (d, *J* = 73.5 Hz), 120.0 (d, *J* = 8.8 Hz), 115.5 (d, *J* = 10.1 Hz), 115.0 (d, *J* = 10.4 Hz), 109.4 (d, *J* = 59.2 Hz), 108.6 (d, *J* = 60.5 Hz), 56.0, 39.8 (d, *J* = 69.7 Hz), 36.2, 34.6, 27.3 (d, *J* = 10.6 Hz), 21.5. ^{31}P NMR (162 MHz, Chloroform-*d*) δ 56.6. HRMS (ESI) calculated for $[C_{30}H_{33}O_5P\text{-}H]^-$: 503.1993, found: 503.1189. $[\alpha]_D^{20}$= - 18.6 (c = 0.5, $CHCl_3$). HPLC separation (Chiralpak IC, 4.6 x 250mm; *i*-PrOH / hexane = 2 / 3, 1.0 mL/min, 210 nm; tr (minor) = 9.0 min, tr (major) = 10.4 min, 98.5:1.5 e.r.).

(*R*)-((1s,3R,5R,7S)-adamantan-1-yl)(3-bromo-2-hydroxy-5-methoxyphenyl)(2-hydroxy-5-methoxyphenyl) phosphine sulfide (6)

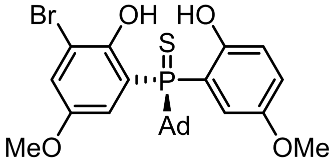

Colorless oil, 35.1 mg, 67% yield. ^{1}H NMR (400 MHz, Chloroform-*d*) δ 8.45 -8.33 (m, 1H), 7.94-7.88 (m, 1H), 7.48-7.44 (m, 1H), 7.31 (d, *J* = 8.1 Hz, 1H), 7.13 (dt, *J* = 8.6, 4.1 Hz, 1H), 3.88 (d, *J* = 9.0 Hz, 6H), 2.00 (s, 6H), 1.82-1.71 (m, 3H), 1.67-1.59 (m, 6H). ^{13}C NMR (101 MHz, Chloroform-*d*) δ 158.4, 154.2, 151.8 (d, *J* = 14.3 Hz), 151.6 (d, *J* = 15.7 Hz), 123.1 (d, *J* = 2.4 Hz), 120.6 (d, *J* = 2.5 Hz), 120.1 (d, *J* = 8.7 Hz), 115.8 (d, *J* = 10.4 Hz), 115.3 (d, *J* = 10.5 Hz), 113.1 (d, *J* = 11.3 Hz), 109.8, 108.0 (d, *J* = 90.8 Hz), 56.1, 56.0, 39.8 (d, *J* = 69.6 Hz), 36.1 (d, *J* = 1.7 Hz), 34.4 (d, *J* = 2.0 Hz), 27.2 (d, *J* = 10.6 Hz). ^{31}P NMR (162 MHz, Chloroform-*d*) δ 56.3. HRMS (ESI) calculated for $[C_{24}H_{28}BrO_4PS\text{-}H]^-$: 523.0536, found: 523.0538. $[\alpha]_D^{20}$= - 28.6 (c = 0.5, $CHCl_3$). HPLC separation (Chiralpak IC, 4.6 x 250mm; *i*-PrOH / hexane = 1 / 3, 1.0 mL/min, 210 nm; tr (minor) = 9.9 min, tr (major) = 11.7 min, 98.5:1.5 e.r.).

Ethyl (*R*)-(3-bromo-2-hydroxy-5-methoxyphenyl)(2-((2-(diphenylphosphanyl)benzyl)oxy)-5-methoxy phenyl)phosphinate (7)

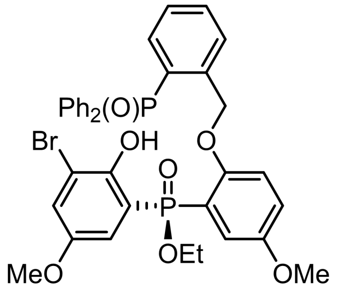

Colorless oil, 56.7 mg, 82% yield. ^{1}H NMR (400 MHz, Chloroform-*d*) δ 11.15 (s, 1H), 7.57-7.44 (m, 1H), 7.40-7.31 (m, 2H), 7.25 (s, 4H), 7.18-

7.13 (m, 7H), 7.06 (t, *J* = 6.8 Hz, 1H), 6.83-6.66 (m, 3H), 6.58 (dt, *J* = 16.3, 8.1 Hz, 1H), 5.16-5.02 (m, 2H), 4.17 (dt, *J* = 10.3, 7.2 Hz, 1H), 3.97 (dt, *J* = 10.0, 7.4 Hz, 1H), 3.68 (s, 3H), 3.44 (s, 3H), 1.29 (t, *J* = 7.0 Hz, 3H). ^{13}C NMR (101 MHz, Chloroform-*d*) δ 154.2 (d, *J* = 3.8 Hz), 153.5 (d, *J* = 5.8 Hz), 153.4 (d, *J* = 15.2 Hz), 152.0 (d, *J* = 17.4 Hz), 140.2 (d, *J* = 22.1 Hz), 135.5 (d, *J* = 5.0 Hz), 135.5 (d, *J* = 4.8 Hz), 134.0 (d, *J* = 20.6 Hz), 132.7, 129.3, 129.1 (d, *J* = 4.1 Hz), 128.8 (d, *J* = 7.2 Hz), 127.8, 126.8, 124.4, 120.5 (d, *J* = 2.0 Hz), 118.4, 118.0, 114.9 (d, *J* = 10.9 Hz), 114.4 (d, *J* = 12.8 Hz), 112.9, 111.7 (d, *J* = 16.4 Hz), 68.1, 61.8 (d, *J* = 5.8 Hz), 55.9, 29.7, 16.4 (d, *J* = 6.7 Hz). ^{31}P NMR (162 MHz, Chloroform-*d*) δ 35.8, -16.6. HRMS (ESI) calculated for $[C_{35}H_{33}BrO_6P_2-H]^-$: 691.0843, found: 691.0845. $[\alpha]_D^{20}$= - 40.6 (c = 0.5, $CHCl_3$). HPLC separation (Chiralpak AD-H, 4.6 x 250mm; *i*-PrOH / hexane = 1 / 5, 1.0 mL/min, 210 nm; tr (major) = 12.7 min, tr (minor) = 19.7 min, 99:1 e.r.).

(2*R*,3*R*)-2-benzyl-3-(furan-2-yl)-3-hydroxy-1-phenylpropan-1-one (8).

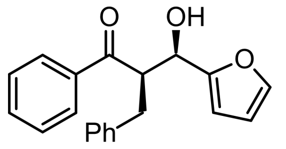

71% yield. The NMR data is in accord with literature.[9] HPLC separation (Chiralpak AS-H, 4.6 x 250mm; *i*-PrOH / hexane = 1 / 49, 1.0 mL/min, 210 nm; tr (anti, major) = 36.0 min, tr (syn, major) = 45.9 min, tr (syn, minor) = 49.0 min, tr (anti, major) = 77.0 min, 52:17:11:20 e.r.)

(*R*)-((3S,5S,7S)-adamantan-1-yl)(3-chloro-2-hydroxy-5-methoxyphenyl)(2-hydroxy-5-methoxyphenyl) phosphine oxide (9)

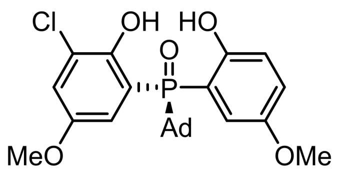

White solid, 23.7 mg, 51% yield. ^{1}H NMR (400 MHz, Chloroform-*d*) δ 11.30 (s, 1H), 10.56 (s, 1H), 7.14 (d, *J* = 2.9 Hz, 1H), 7.04 (dd, *J* = 9.1, 3.0 Hz, 1H), 6.95- 6.88 (m, 3H), 3.76 (d, *J* = 3.3 Hz, 6H), 2.05- 2.00 (m, 9H), 1.72 (q, *J* = 12.6 Hz, 6H). ^{13}C NMR (101 MHz, Chloroform-*d*) δ 158.3, 153.5, 151.8 (d, *J* = 14.3 Hz), 151.4 (d, *J* = 15.7 Hz), 123.8 (d, *J* = 12.3 Hz), 120.5, 120.1, 120.0, 115.3 (d, *J* = 10.2 Hz), 114.9 (d, *J* = 10.0 Hz), 110.4 (d, *J* = 87.2 Hz), 108.0 (d, *J* = 90.8 Hz), 56.1, 56.0, 39.8 (d, *J* = 69.6 Hz), 36.1 (d, *J* = 1.8 Hz), 34.4 (d, *J* = 2.0 Hz), 27.2 (d, *J* = 10.6 Hz). ^{31}P NMR (162 MHz, Chloroform-*d*) δ 56.5. HRMS (ESI) calculated for $[C_{24}H_{28}ClO_5P-H]^-$: 463.1261, found: 463.1262. $[\alpha]_D^{20}$= - 31.6 (c = 0.5, $CHCl_3$). HPLC separation (Chiralpak IC, 4.6 x 250mm; *i*-PrOH / hexane = 1 / 3, 1.0 mL/min, 210 nm; tr (minor) = 8.6 min, tr (major) = 9.5 min, 99.5:0.5 e.r.).

(*S*)-((1*s*,3*R*,5*S*,7*S*)-adamantan-1-yl)(3-bromo-2-hydroxy-5-methoxyphenyl)(3-chloro-2-hydroxy-5-methoxyphenyl)phosphine oxide (10)

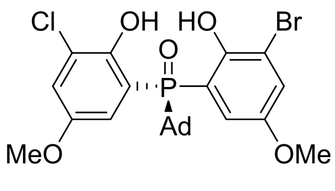

White solid, 26.5 mg, 49% yield. ^{1}H NMR (400 MHz, Chloroform-*d*) δ

11.42 (s, 1H), 10.68 (s, 1H), 7.16 (dd, *J* = 9.1, 3.0 Hz, 1H), 7.07-7.00 (m, 3H), 3.88 (d, *J* = 3.3 Hz, 6H), 2.17-2.13 (m, 9H), 1.84 (q, *J* = 12.6 Hz, 6H). ^{13}C NMR (101 MHz, Chloroform-*d*) δ 158.4, 153.6 (d, *J* = 1.9 Hz), 151.9 (d, *J* = 14.3 Hz), 151.5 (d, *J* = 15.8 Hz), 123.9 (d, *J* = 12.3 Hz), 120.7, 120.2, 120.1, 115.4 (d, *J* = 10.1 Hz), 115.0 (d, *J* = 10.0 Hz), 110.5 (d, *J* = 87.3 Hz), 108.1 (d, *J* = 90.8 Hz), 56.1 (d, *J* = 12.4 Hz), 39.9 (d, *J* = 69.7 Hz), 36.2 (d, *J* = 1.8 Hz), 34.5 (d, *J* = 2.0 Hz), 27.4, 27.3. ^{31}P NMR (162 MHz, Chloroform-*d*) δ 56.7. HRMS (ESI) calculated for $[C_{24}H_{27}BrClO_5P\text{-}H]^-$: 541.0375, found: 541.0377. $[\alpha]_D^{20}$= - 21.6 (c = 0.5, $CHCl_3$). HPLC separation (Chiralpak IC, 4.6 x 250mm; *i*-PrOH / hexane = 1 / 9, 1.0 mL/min, 210 nm; tr (minor) = 7.7 min, tr (major) = 9.8 min, 90:10 e.r.).

(*S*)-((1*s*,3*R*,5*S*,7*S*)-adamantan-1-yl)(3-bromo-2-hydroxy-5-methoxyphenyl)(2,5-dimethoxyphenyl) phosphine oxide (11)

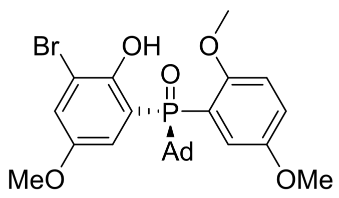

White solid, 49.5 mg, 95% yield. ^{1}H NMR (400 MHz, Chloroform-*d*) δ 12.48 (s, 1H), 7.70 (dd, *J* = 13.4, 3.0 Hz, 1H), 7.30 (dd, *J* = 13.3, 2.9 Hz, 1H), 7.23 (d, *J* = 2.7 Hz, 1H), 7.04 (d, *J* = 3.0 Hz, 1H), 6.90 (dd, *J* = 8.8, 6.3 Hz, 1H), 3.85 (s, 3H), 3.81 (s, 3H), 3.73 (s, 3H), 2.02 (s, 6H), 1.93 (d, *J* = 11.5 Hz, 3H), 1.69 (d, *J* = 13.9 Hz, 6H). ^{13}C NMR (101 MHz, Chloroform-*d*) δ 154.5 (d, *J* = 3.1 Hz), 154.1 (d, *J* = 12.4 Hz), 153.1 (d, *J* = 4.7 Hz), 151.0 (d, *J* = 16.2 Hz), 122.7 (d, *J* = 2.4 Hz), 120.6 (d, *J* = 2.6 Hz), 119.5 (d, *J* = 5.7 Hz), 118.7 (d, *J* = 86.4 Hz), 116.6 (d, *J* = 11.8 Hz), 112.4 (d, *J* = 8.7 Hz), 112.0 (d, *J* = 3.0 Hz), 111.5 (d, *J* = 73.4 Hz), 56.0 (d, *J* = 4.9 Hz), 55.3, 39.3 (d, *J* = 70.8 Hz), 36.4 (d, *J* = 1.8 Hz), 35.4 (d, *J* = 1.8 Hz), 27.8, 27.7. ^{31}P NMR (162 MHz, Chloroform-*d*) δ 51.8. HRMS (ESI) calculated for $[C_{25}H_{30}BrO_5P\text{-}H]^-$: 521.0921, found: 521.0931. $[\alpha]_D^{20}$= - 10.6 (c = 0.5, $CHCl_3$). HPLC separation (Chiralpak IC, 4.6 x 250mm; *i*-PrOH / hexane = 1 / 1, 1.0 mL/min, 210 nm; tr (minor) = 13.2 min, tr (major) = 17.8 min, 73.5:26.5 e.r.).

10. NMR and HPLC spectra

NMR spectra

^{1}H NMR/^{13}C NMR/^{31}P NMR of product 3a

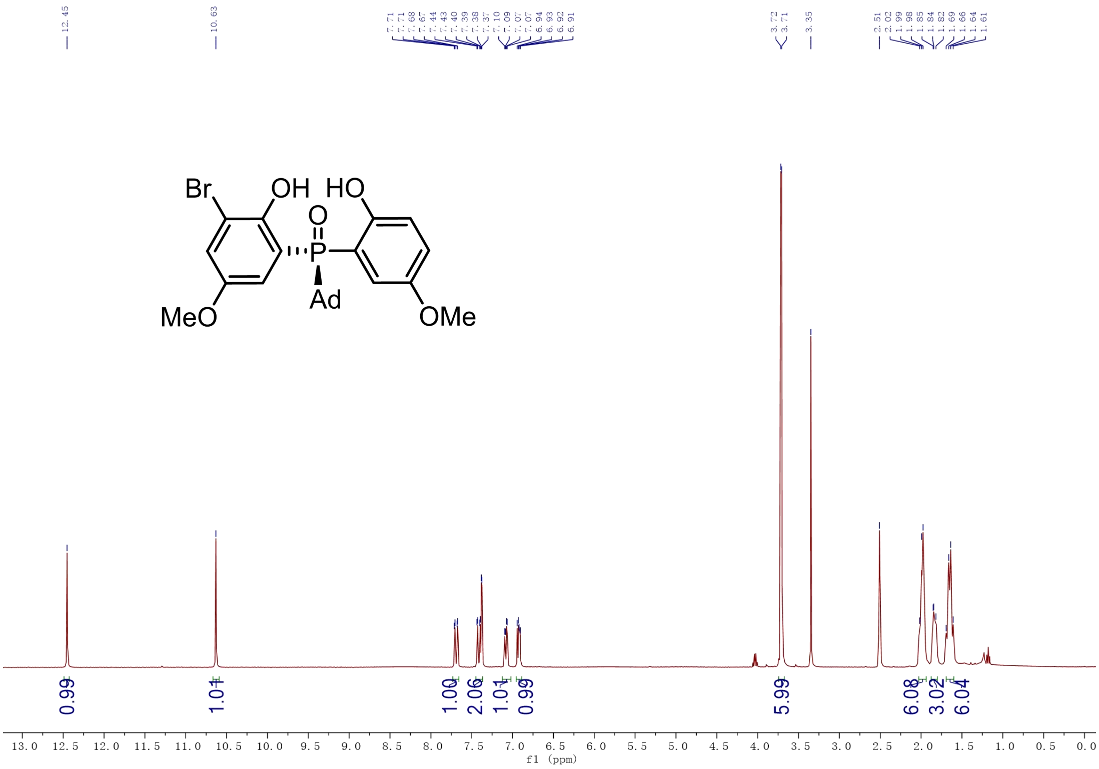

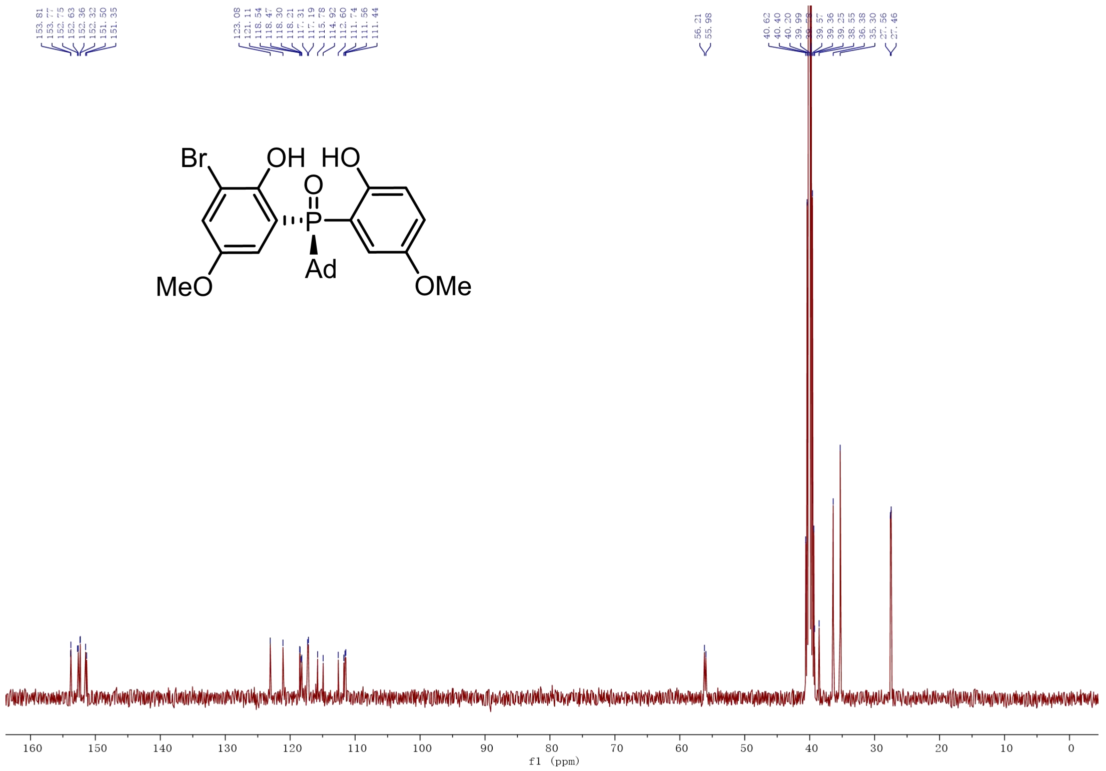
153.81
153.77
152.75
152.63
152.36
152.32
151.50
151.35
123.08
121.11
118.54
118.47
118.30
118.21
117.31
117.19
115.78
114.92
112.60
111.74
111.56
111.44
56.21
55.98
40.62
40.40
40.20
39.99
Br
OH
HO
O
P
Ad
MeO
OMe
f1 (ppm)

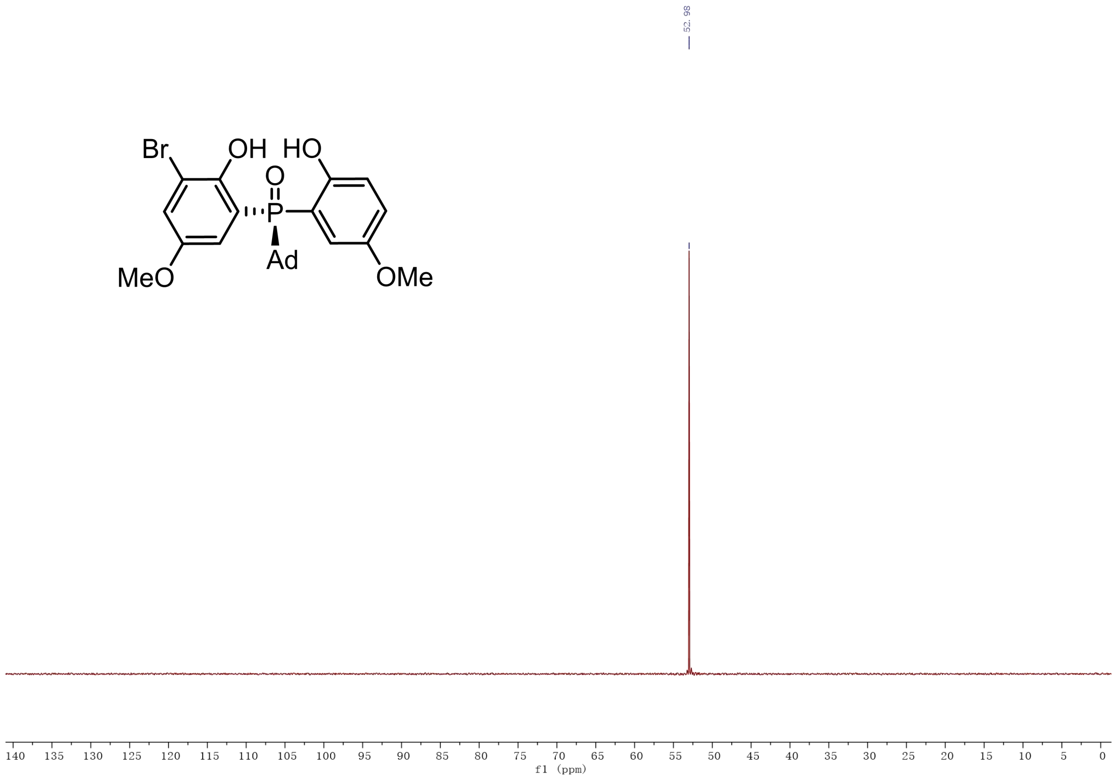
52.98
Br
OH
HO
O
P
Ad
MeO
OMe
f1 (ppm)

^{1}H NMR/^{13}C NMR/^{31}P NMR of product 3b

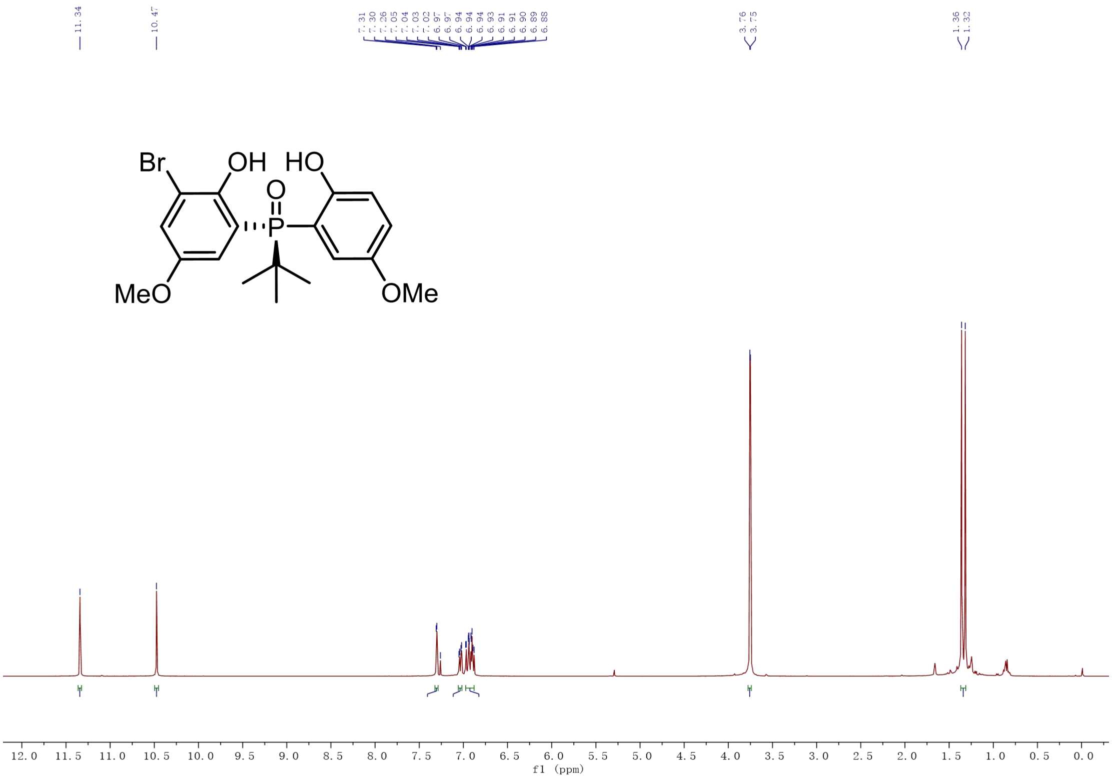

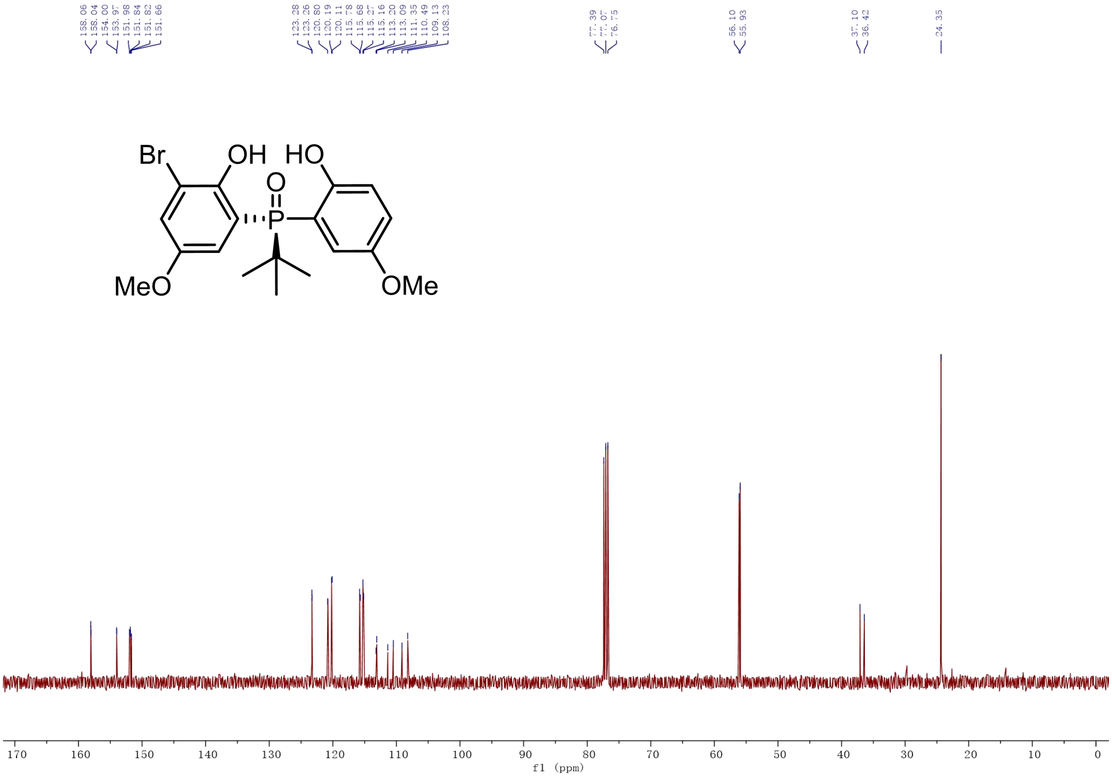

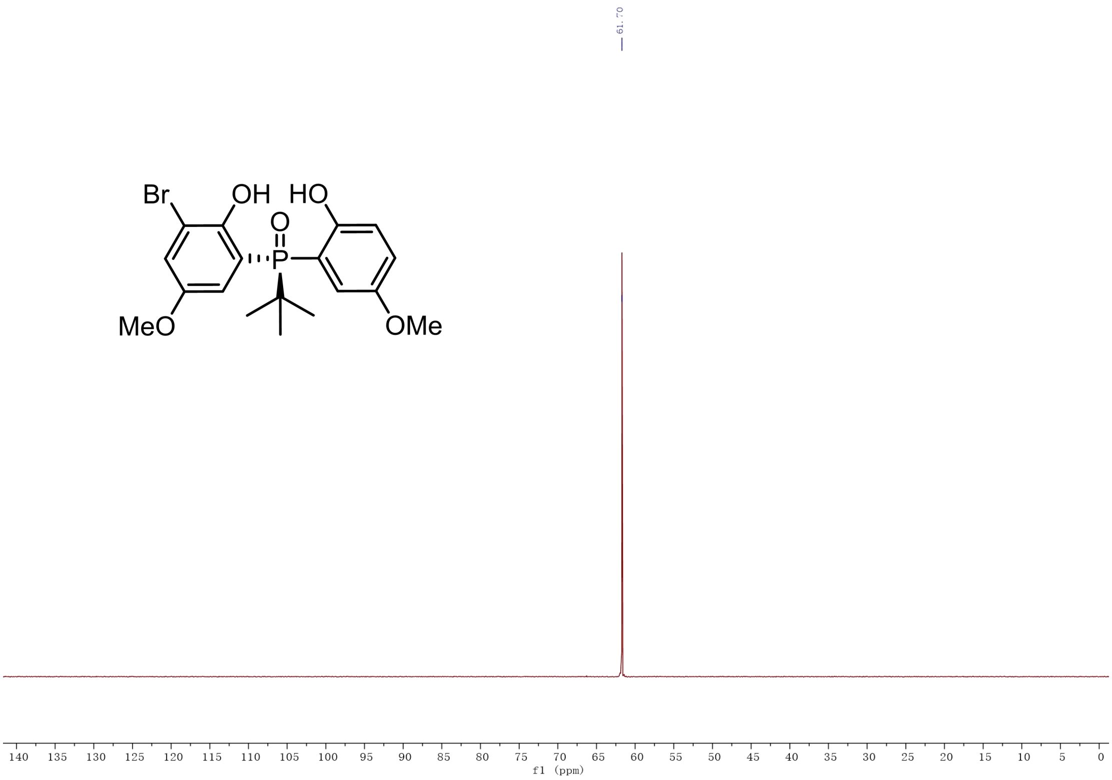

^{1}H NMR/^{13}C NMR/^{31}P NMR of product 3c

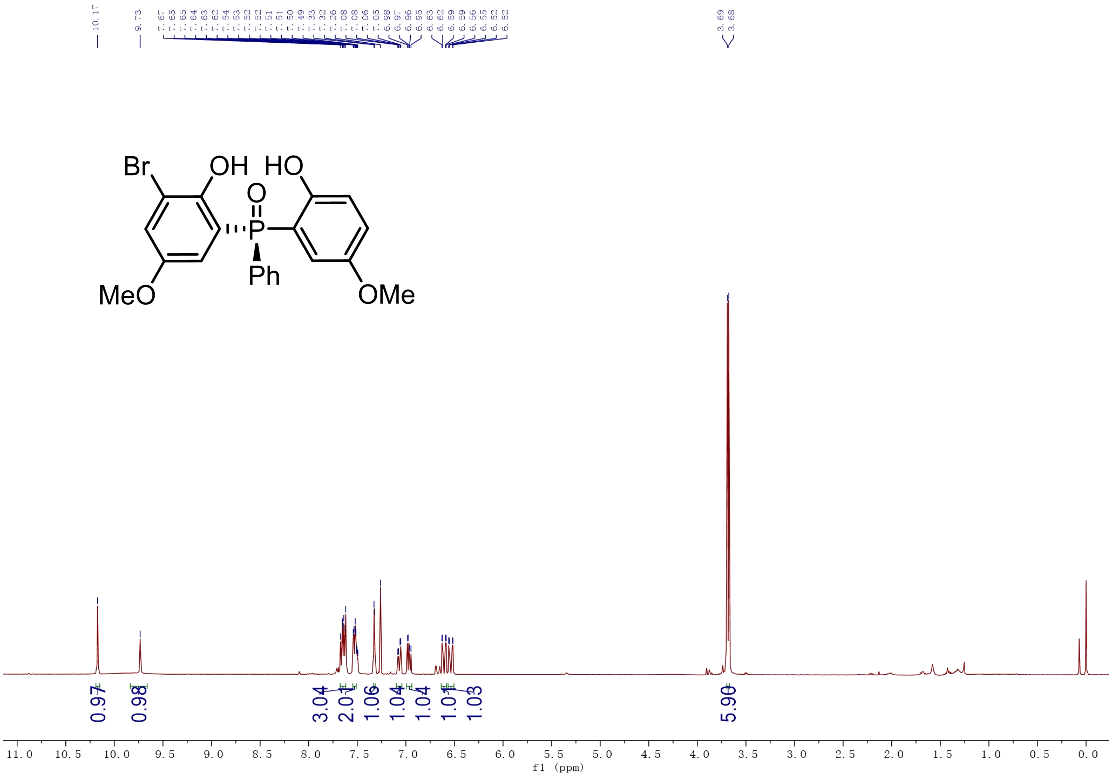

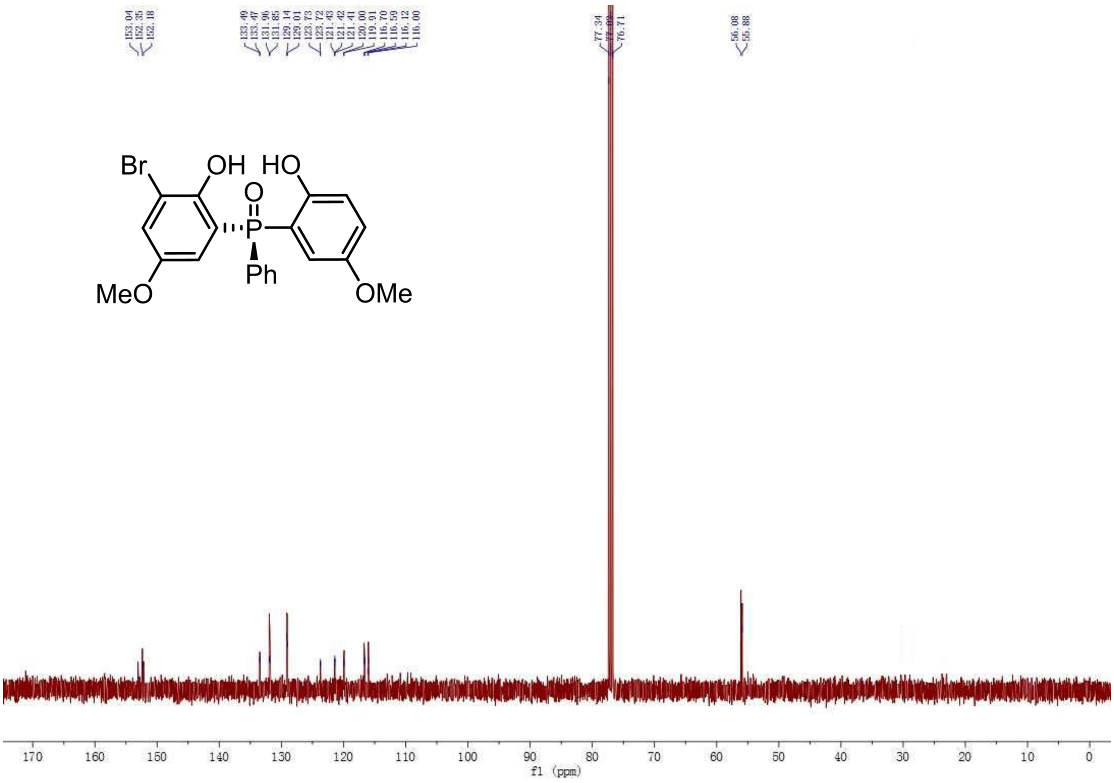

153.04
152.35
152.18
133.49
133.47
131.96
131.85
129.14
129.01
123.73
123.72
121.43
121.42
121.41
120.00
119.91
116.70
116.59
116.12
116.00
77.34
76.71
56.08
55.88
Br
OH
HO
O
P
Ph
MeO
OMe
170
160
150
140
130
120
110
100
90
80
70
60
50
40
30
20
10
0
f1 (ppm)

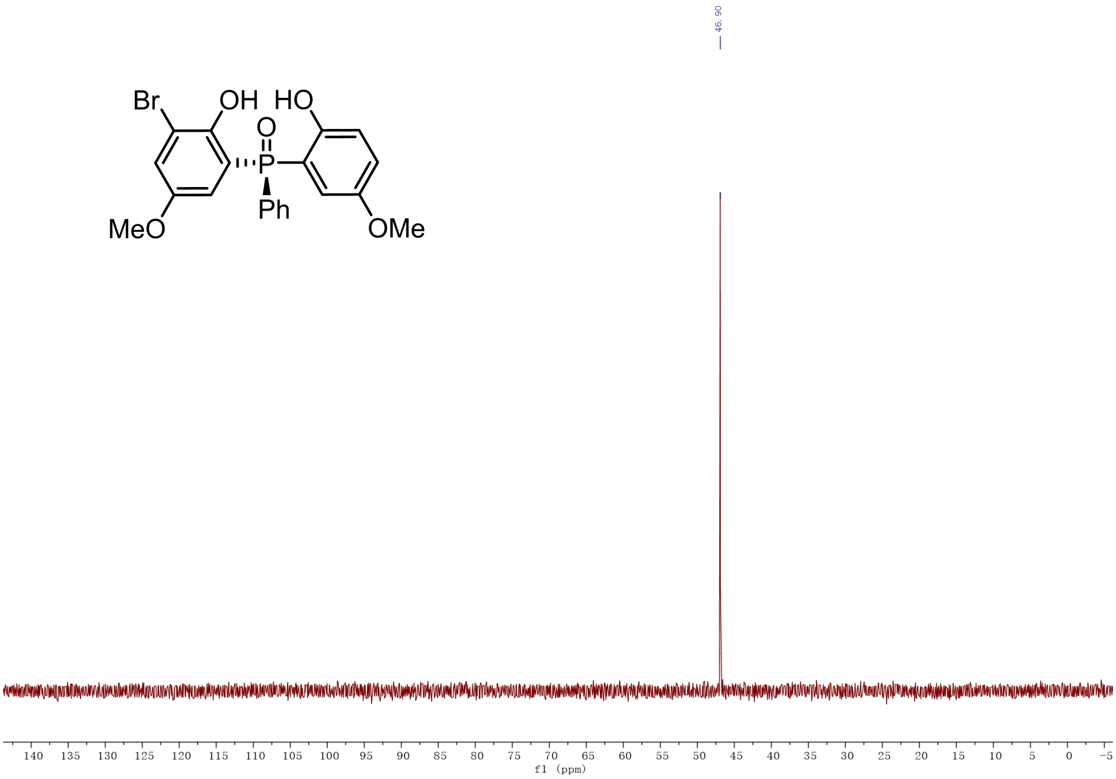

46.90
Br
OH
HO
O
P
Ph
MeO
OMe
140
135
130
125
120
115
110
105
100
95
90
85
80
75
70
65
60
55
50
45
40
35
30
25
20
15
10
5
0
-5
f1 (ppm)

^1H NMR/^{13}C NMR/^{31}P NMR of product 3d

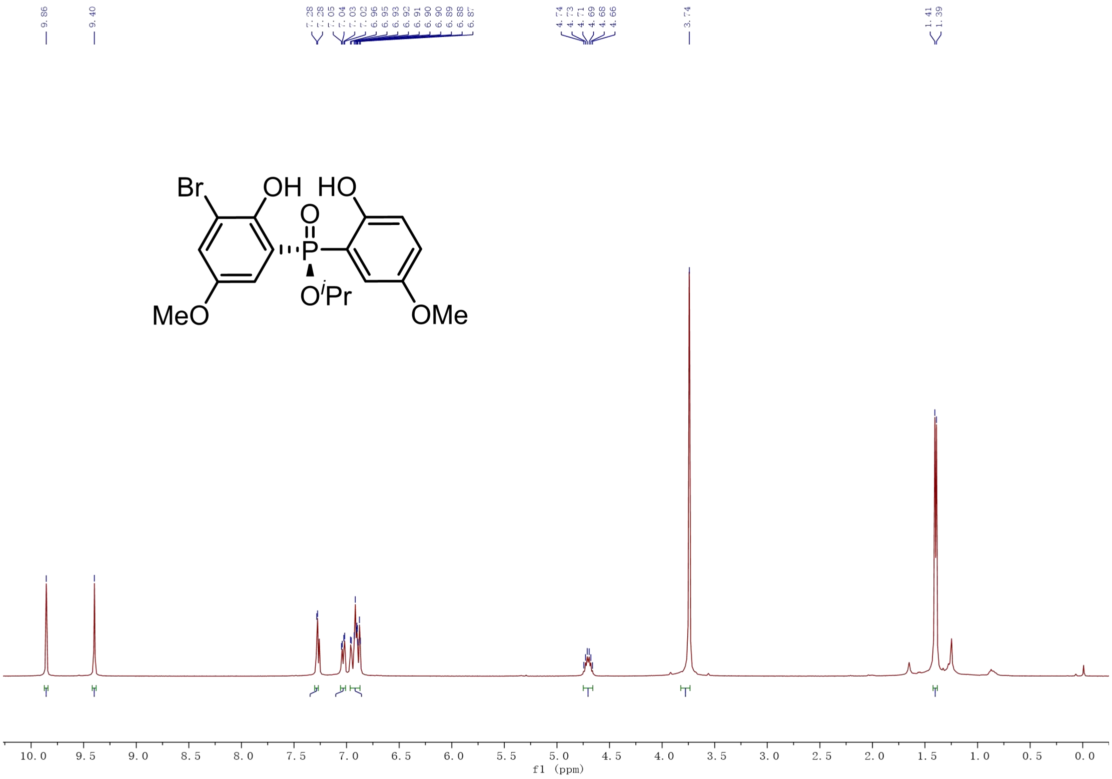

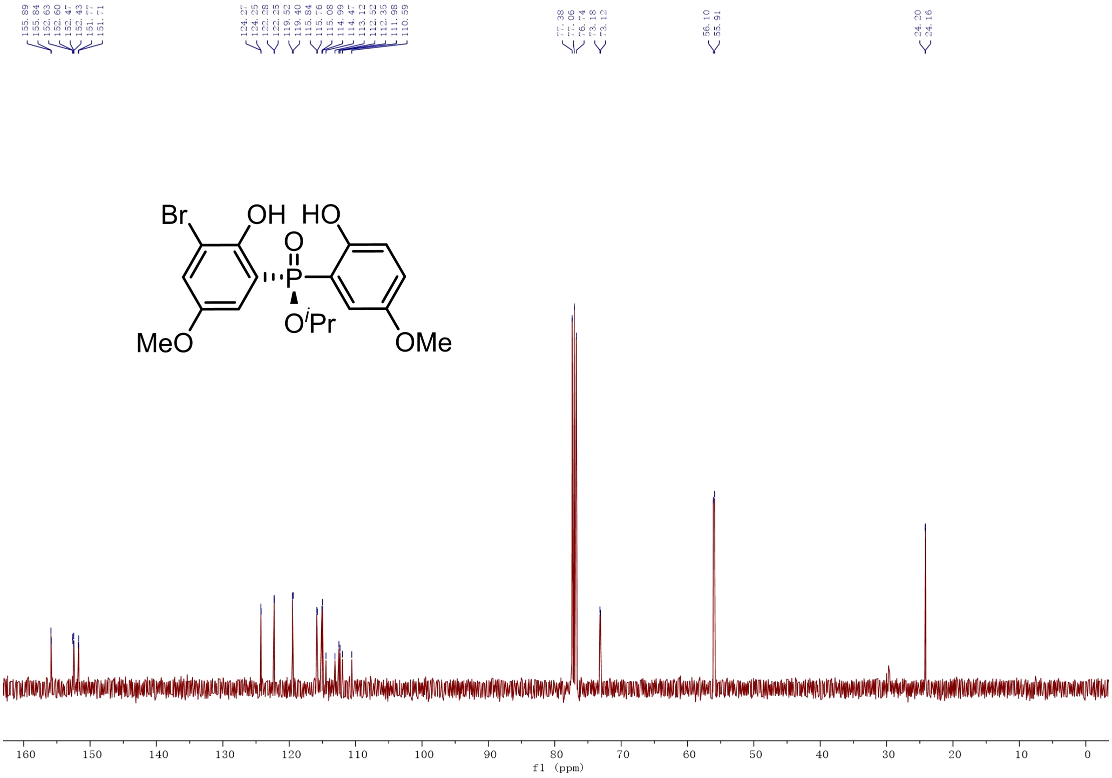

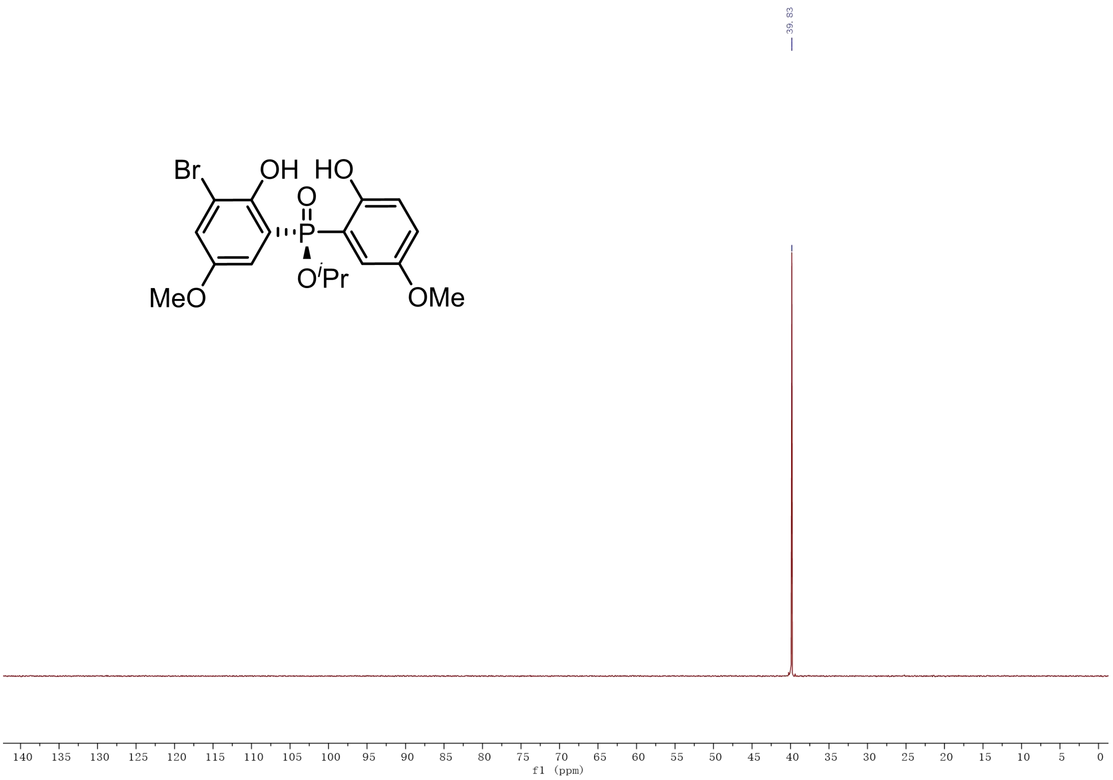

^{1}H NMR/^{13}C NMR/^{31}P NMR of product 3e

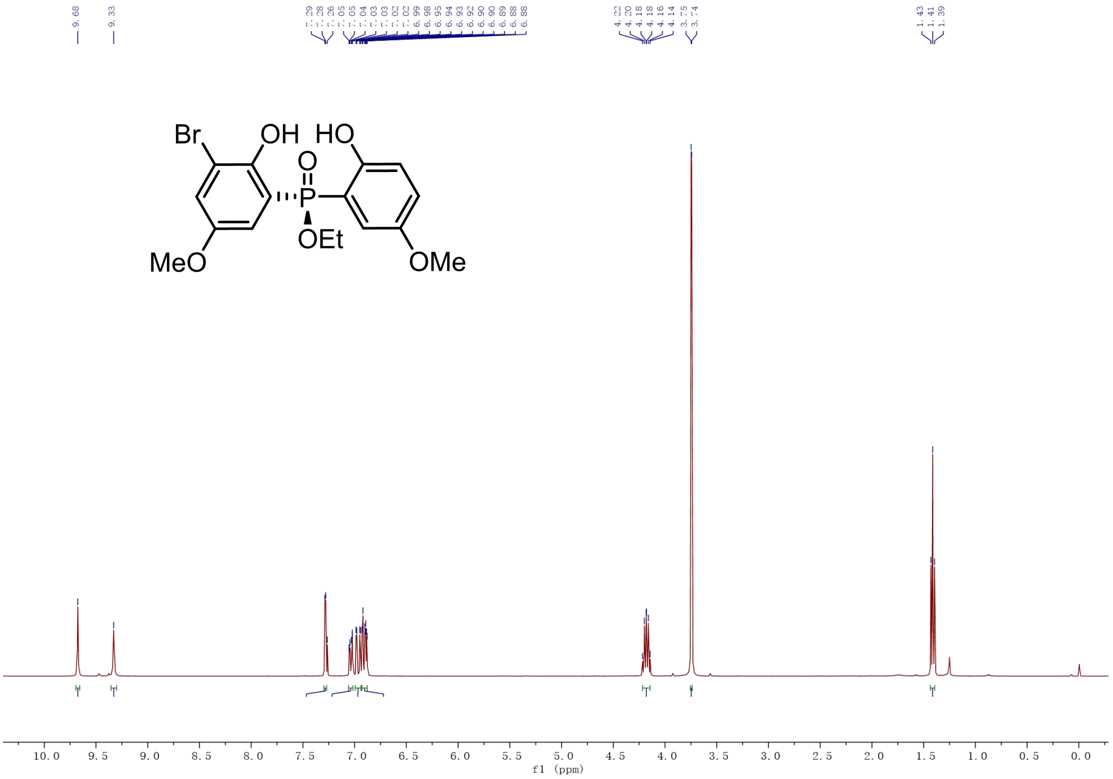

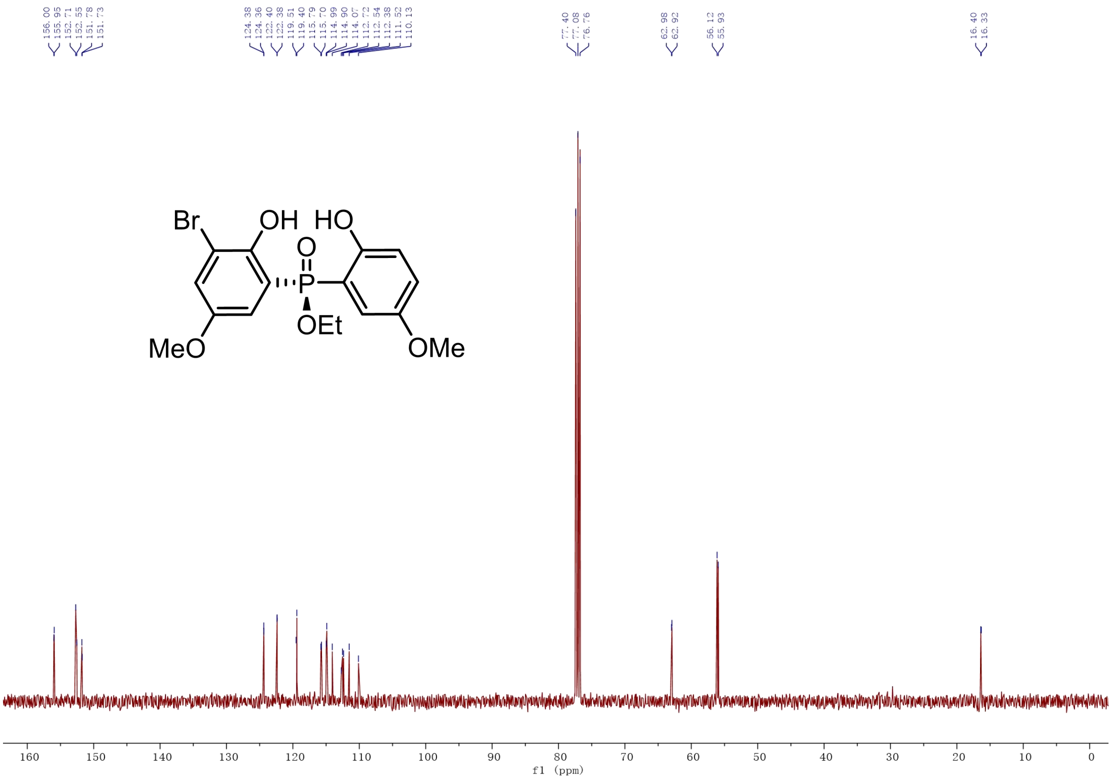

156.00
155.95
152.71
152.55
151.78
151.73
124.38
124.36
122.40
122.38
119.51
119.40
115.79
115.70
114.99
114.90
114.07
112.72
112.54
112.38
111.52
110.13
77.40
77.08
76.76
62.98
62.92
56.12
55.93
16.40
16.33
Br
OH
HO
O
P
OEt
MeO
OMe
f1 (ppm)

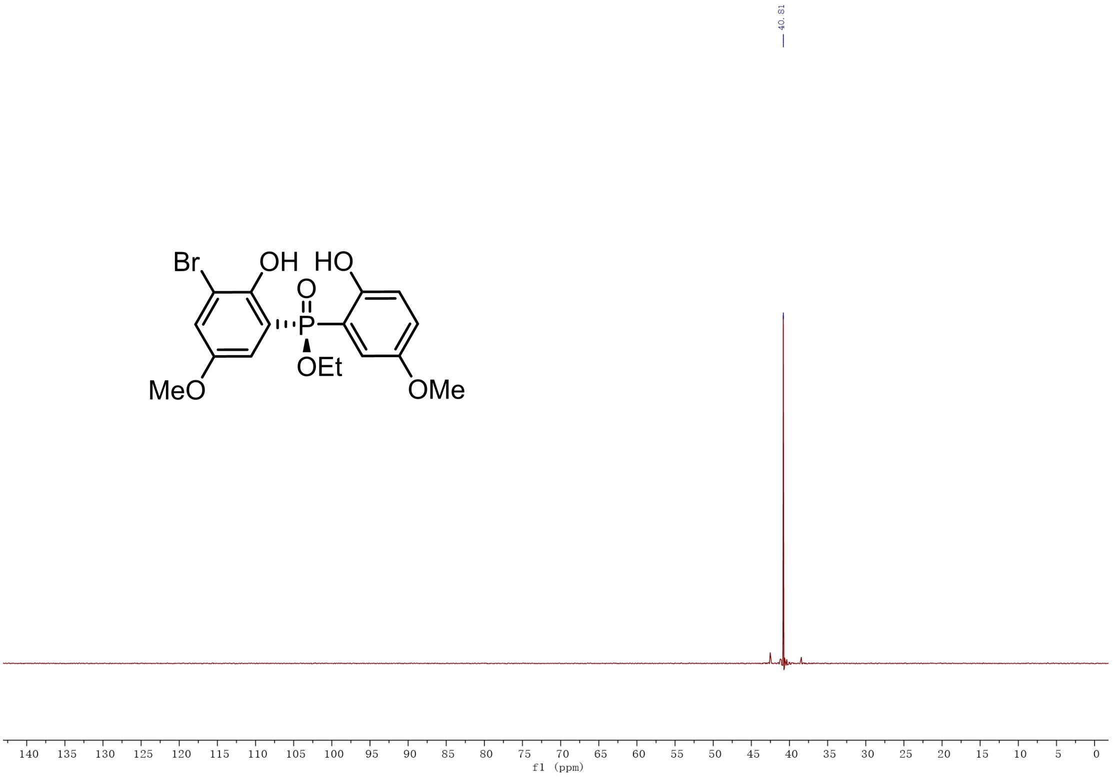

40.81
Br
OH
HO
O
P
OEt
MeO
OMe
f1 (ppm)

^{1}H NMR/^{13}C NMR/^{31}P NMR of product 3f

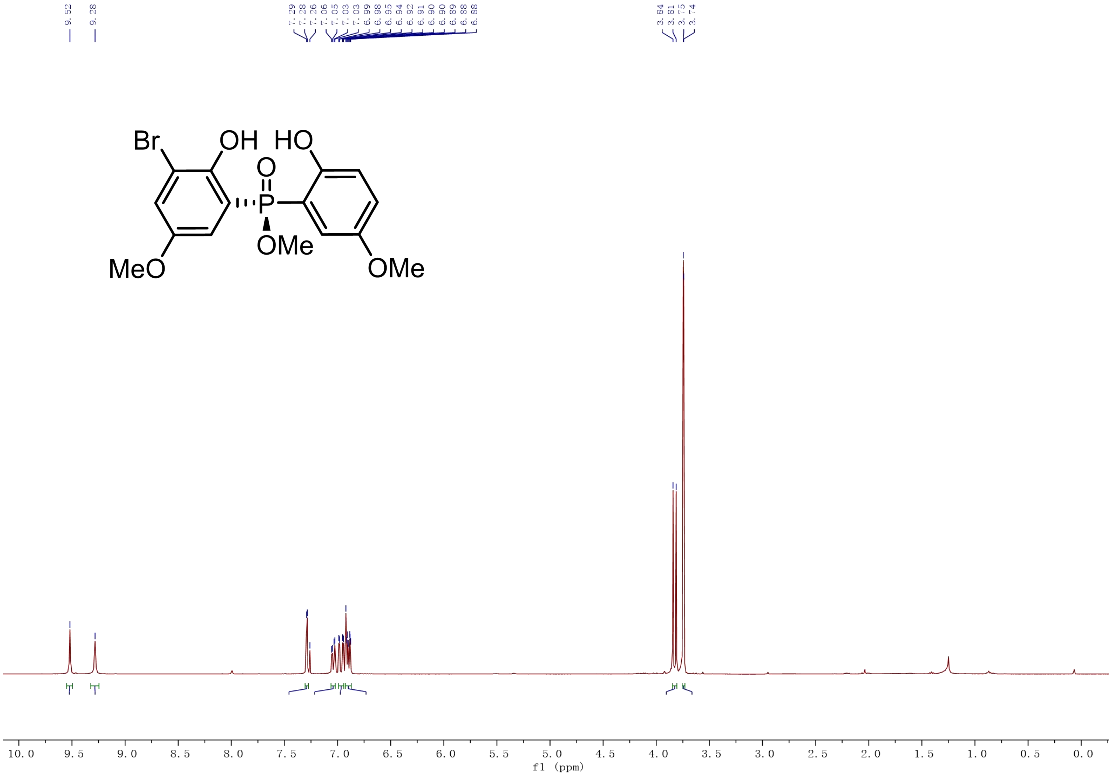

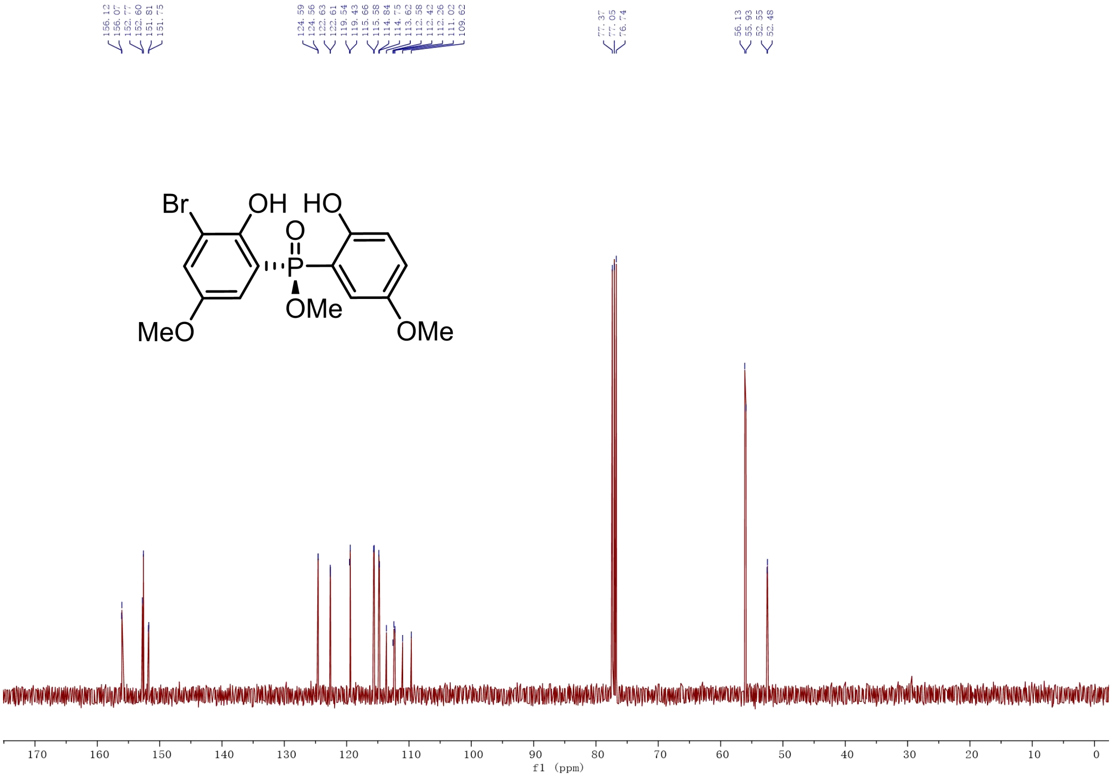

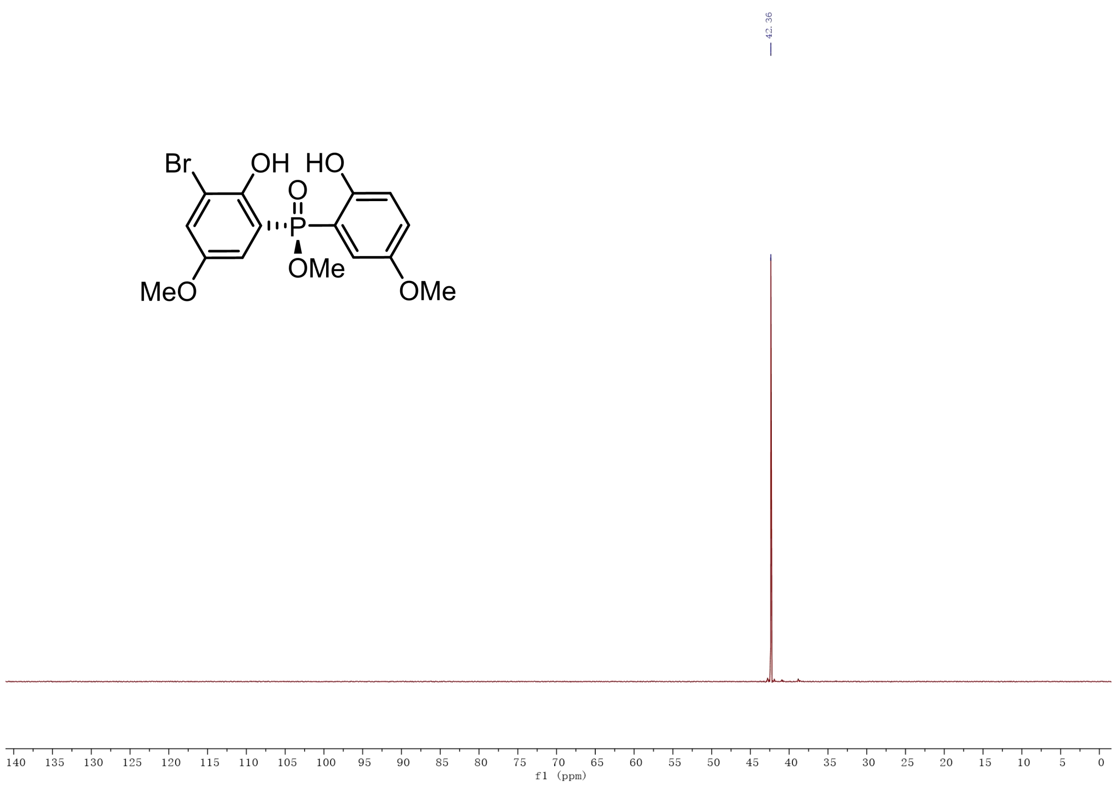

^1H NMR/^{13}C NMR/^{31}P NMR of product 3g

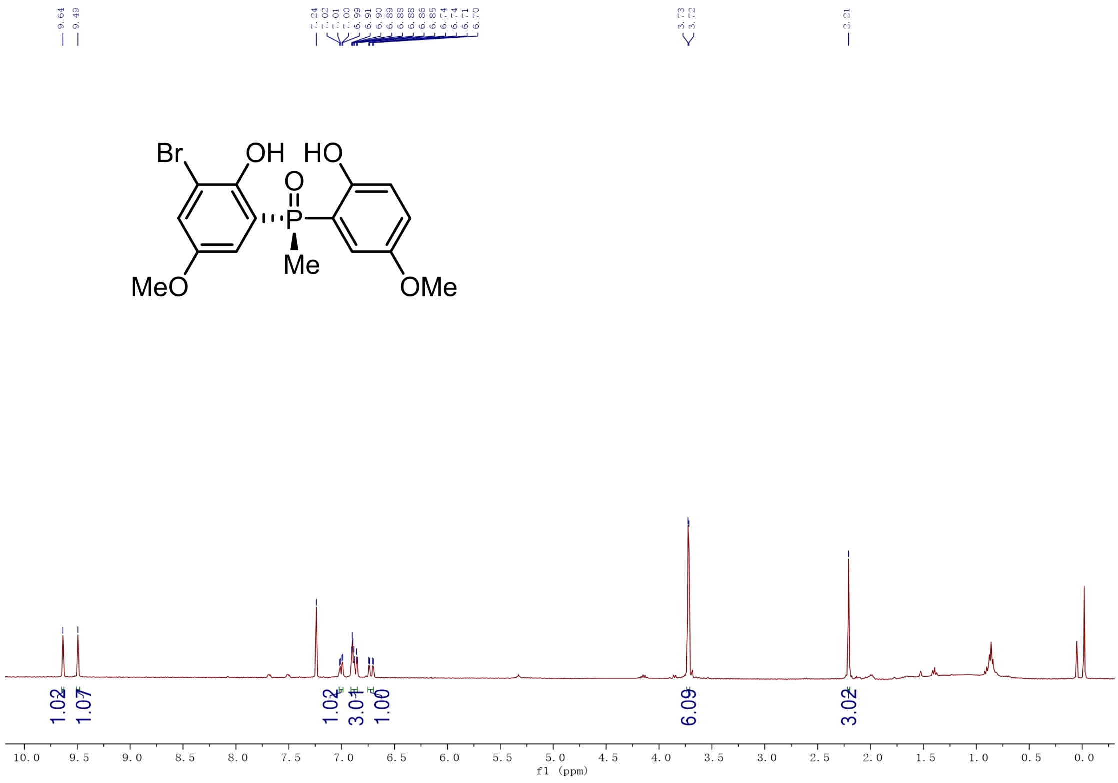

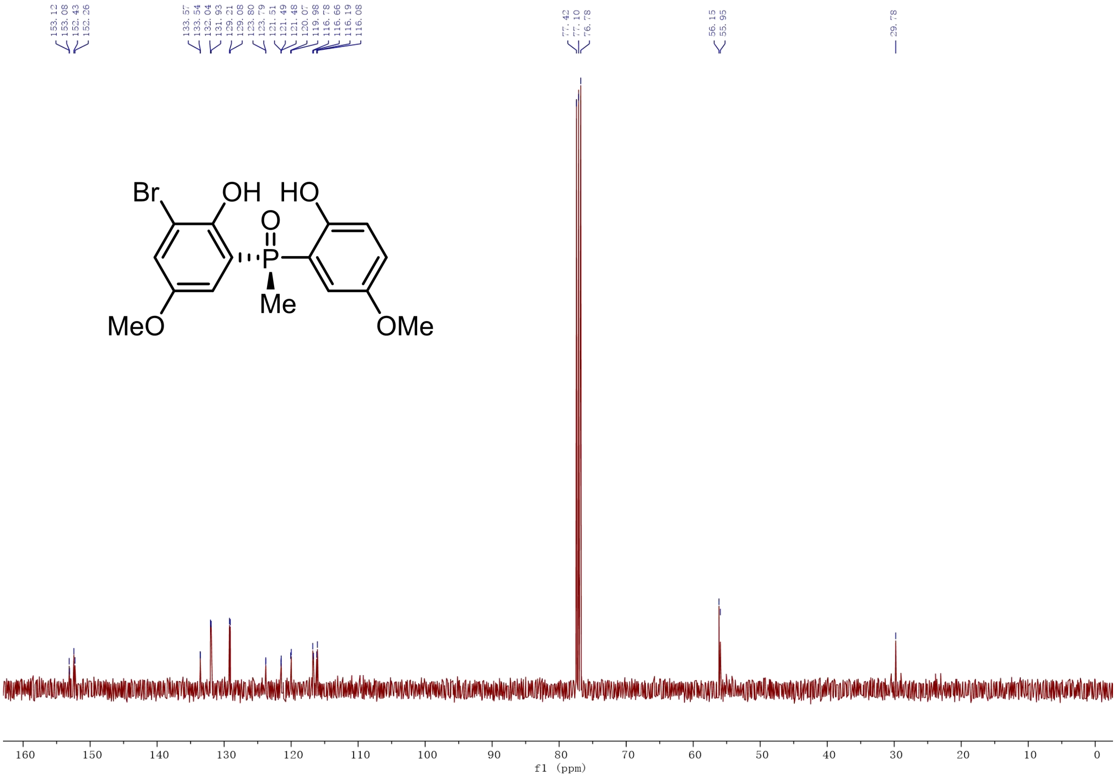
Br
OH
HO
O
P
Me
MeO
OMe
f1 (ppm)

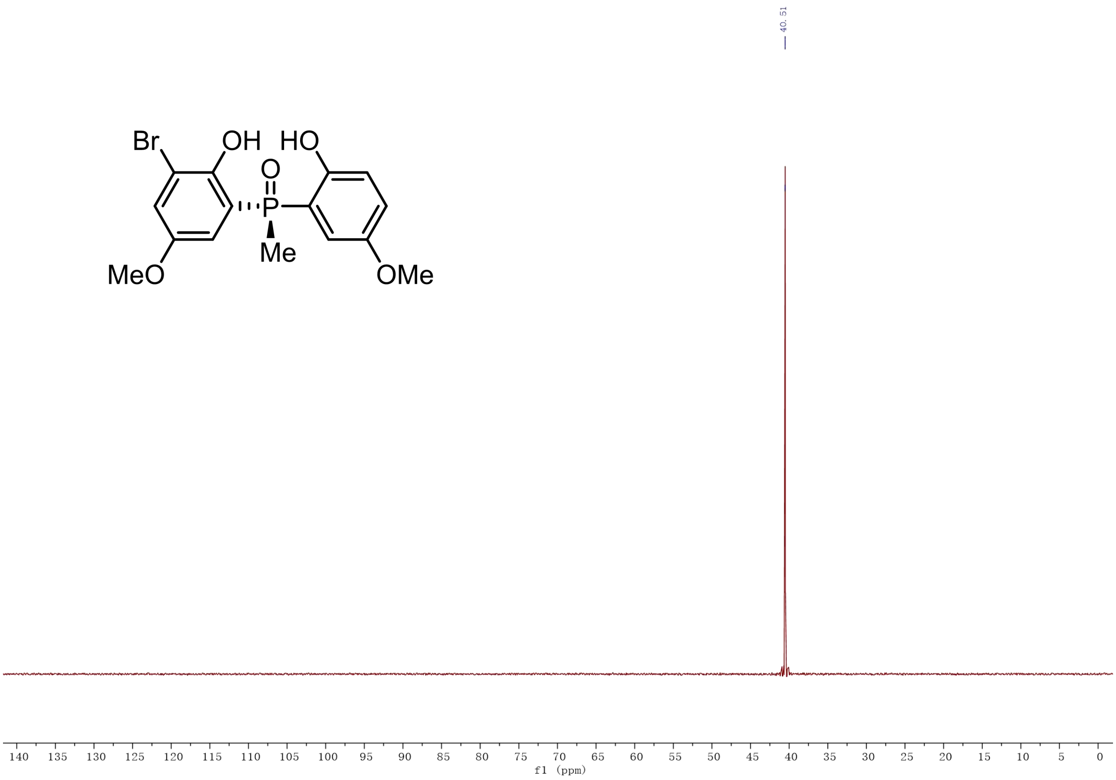
Br
OH
HO
O
P
Me
MeO
OMe
f1 (ppm)

^1H NMR/^{13}C NMR/^{31}P NMR of product 3h

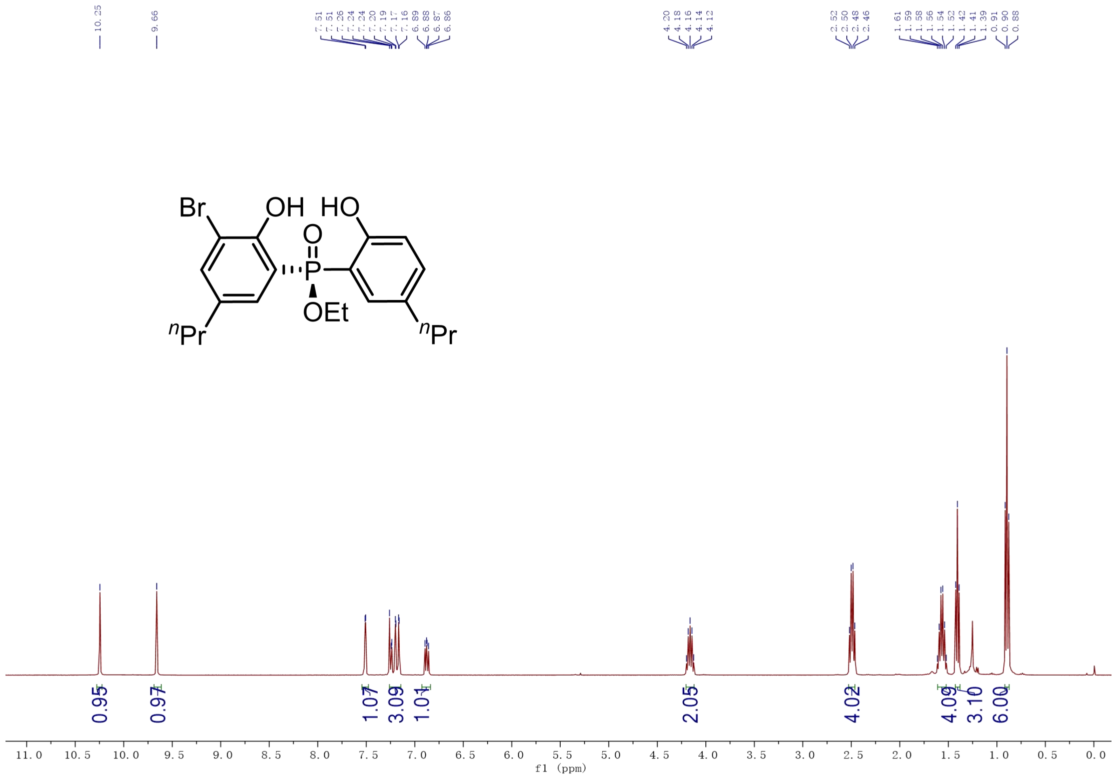

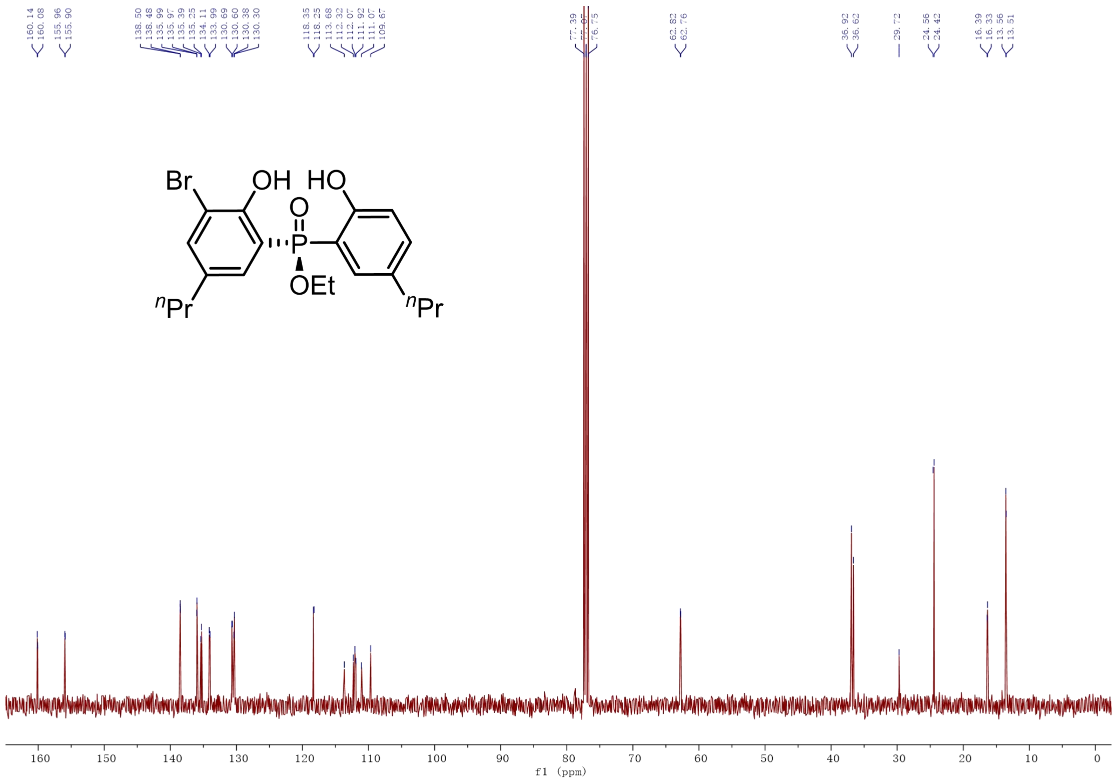

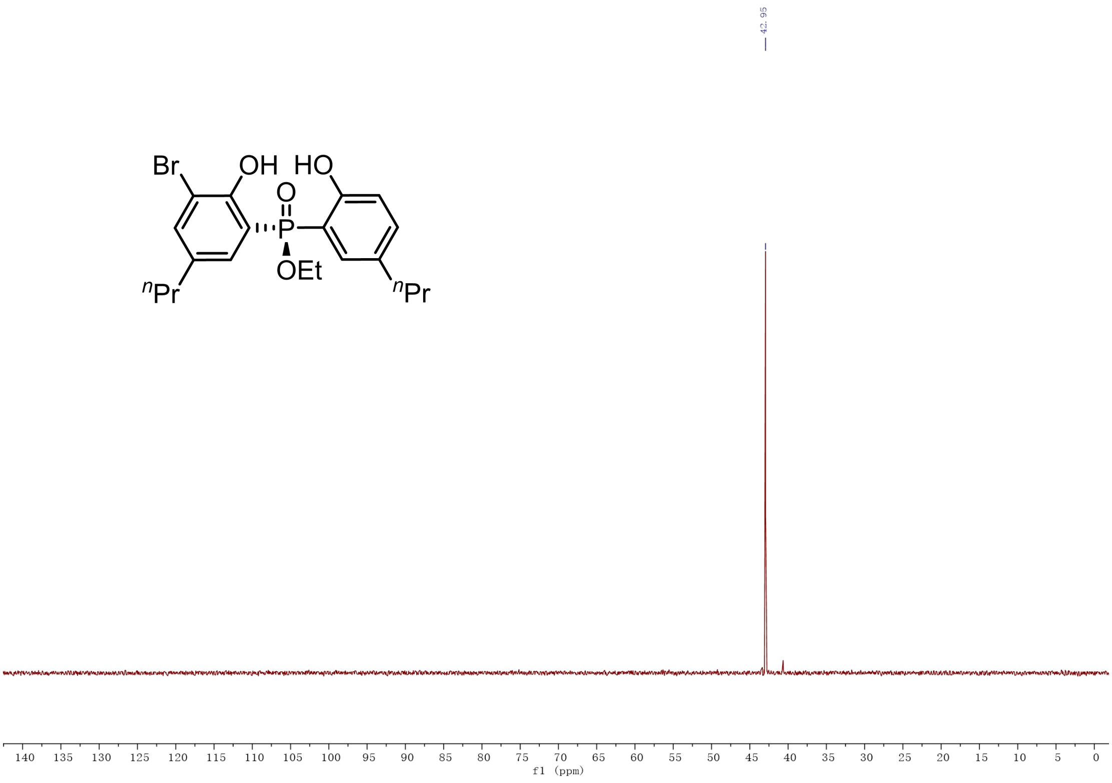

^{1}H NMR/^{13}C NMR/^{31}P NMR of product 3i

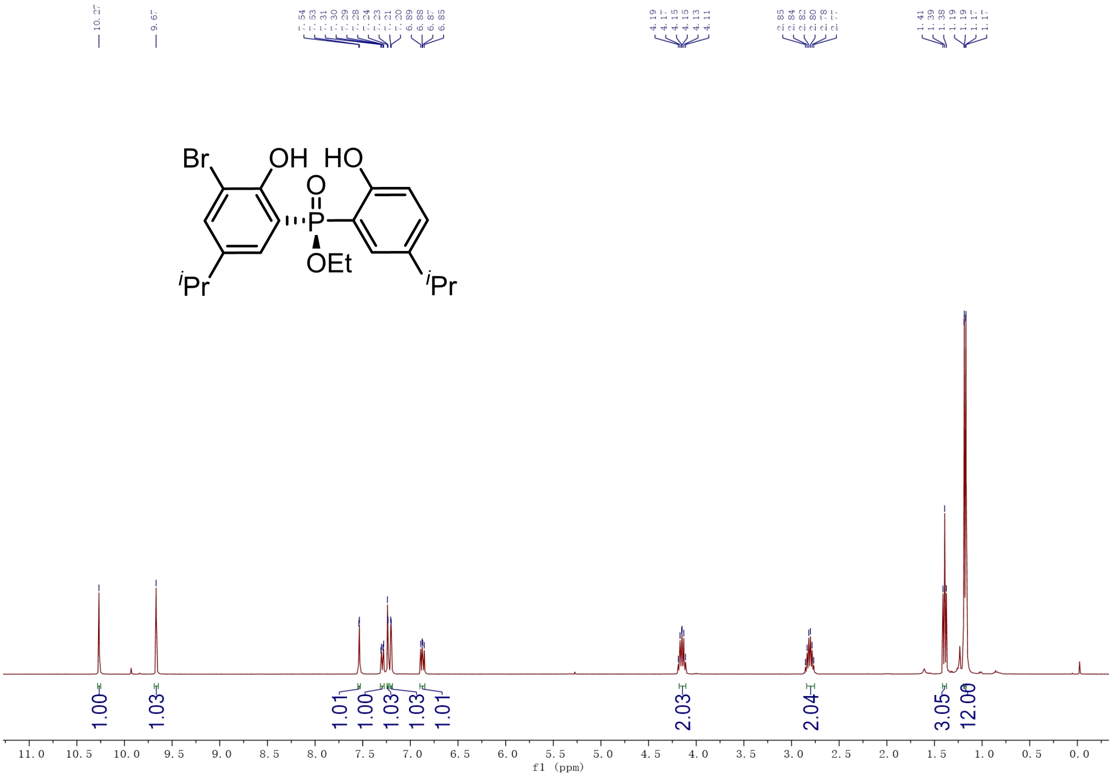

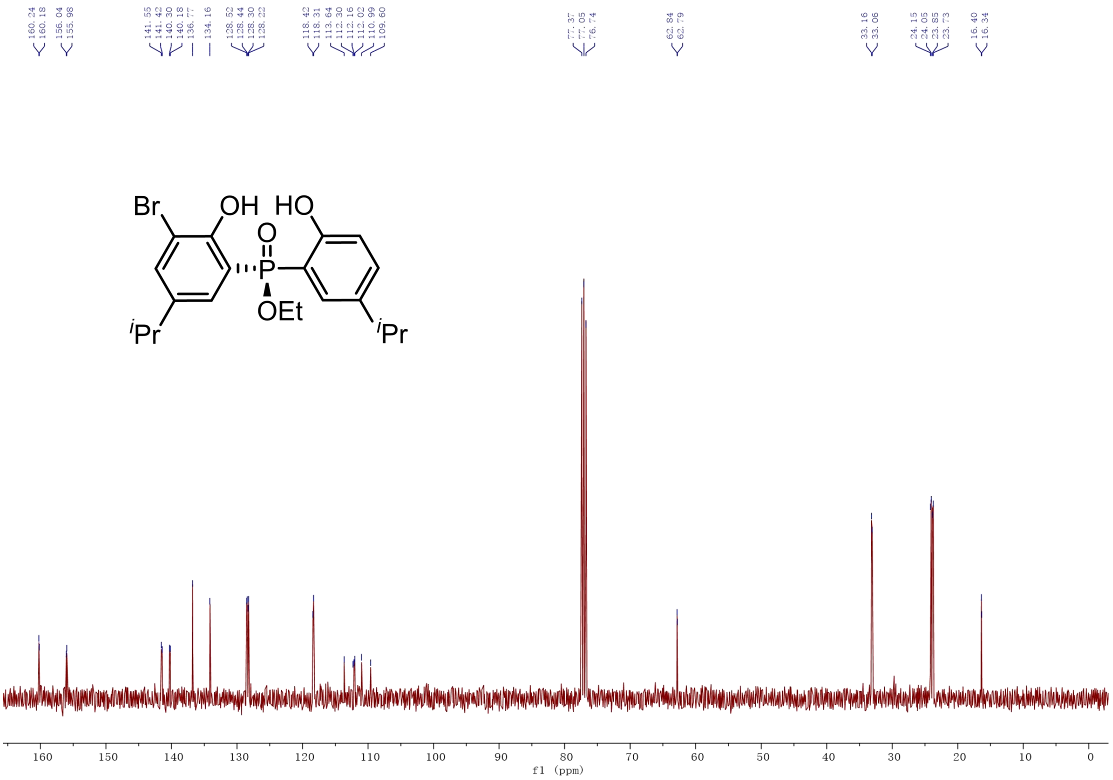
160.24
160.18
156.04
155.98
141.55
141.42
140.30
140.18
136.77
134.16
128.52
128.44
128.30
128.22
118.42
118.31
113.64
112.30
112.16
112.02
110.99
109.60
77.37
77.05
76.74
62.84
62.79
33.16
33.06
24.15
24.05
23.85
23.73
16.40
16.34
Br
OH
HO
O
P
OEt
iPr
iPr
f1 (ppm)

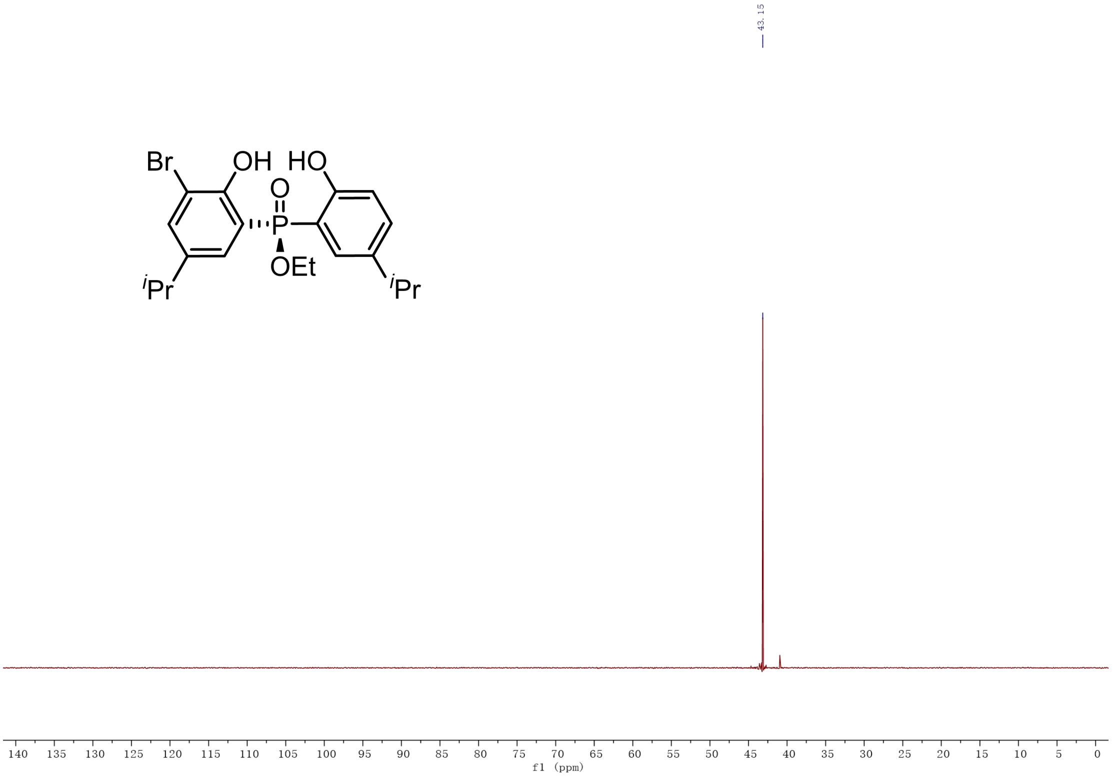
43.15
Br
OH
HO
O
P
OEt
iPr
iPr
f1 (ppm)

^{1}H NMR/^{13}C NMR/^{31}P NMR of product 3j

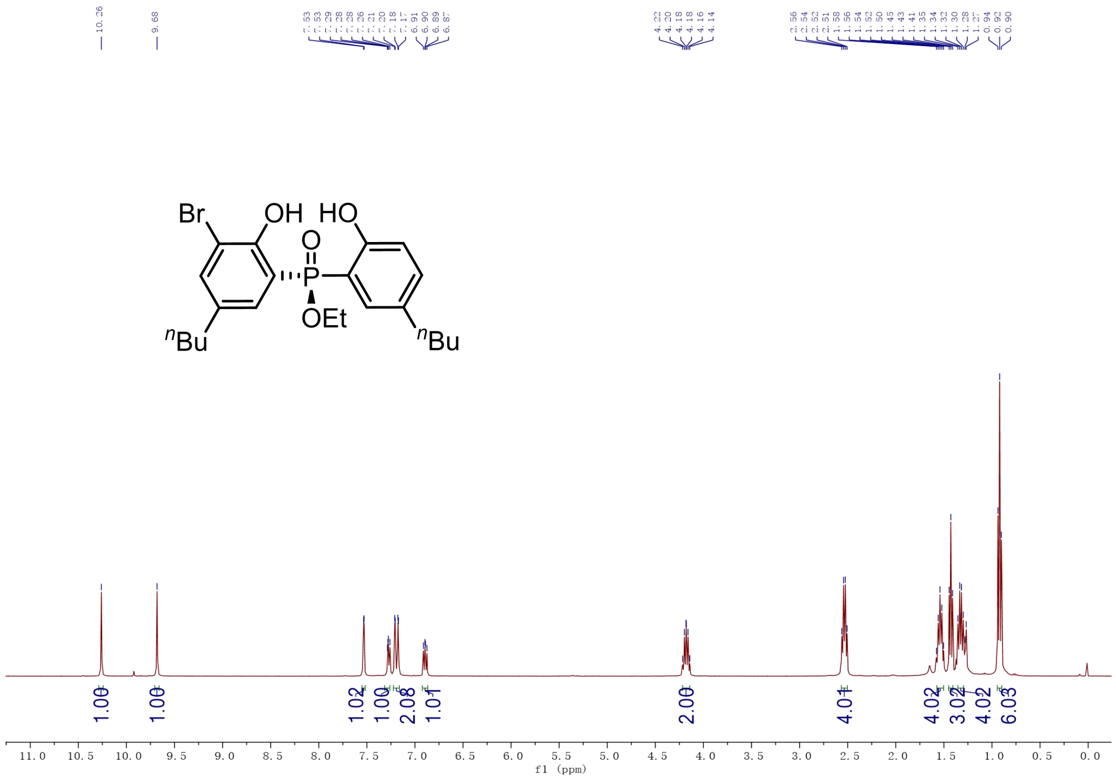

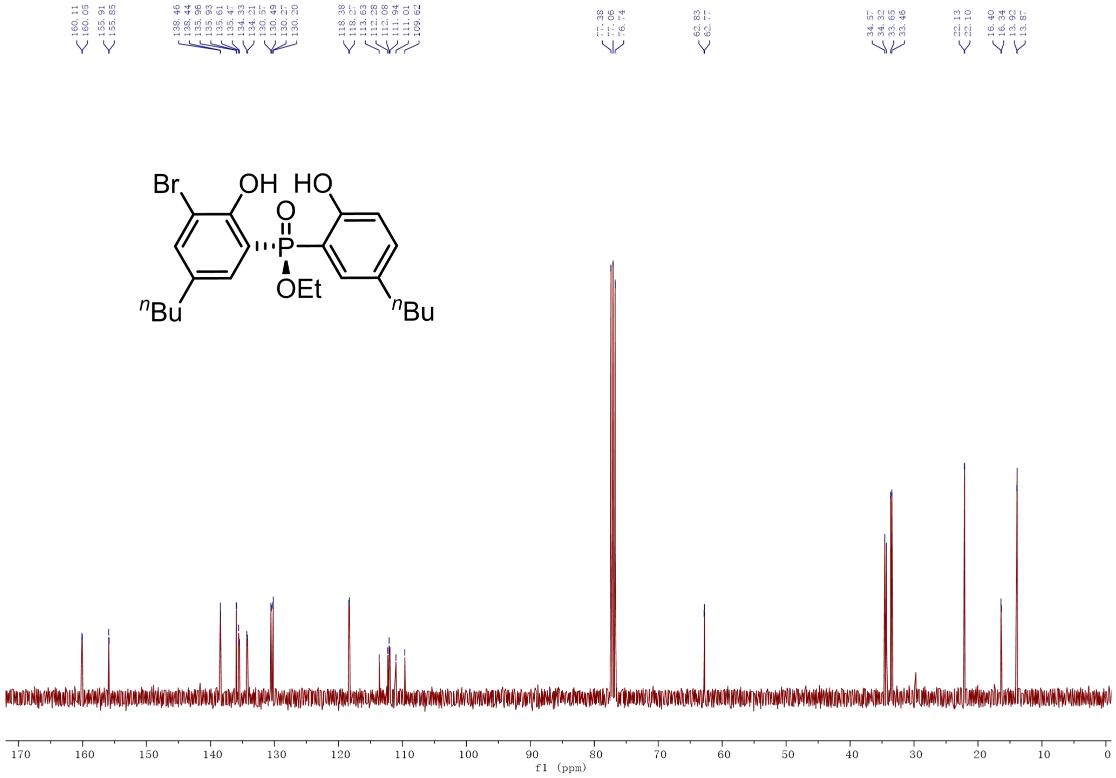

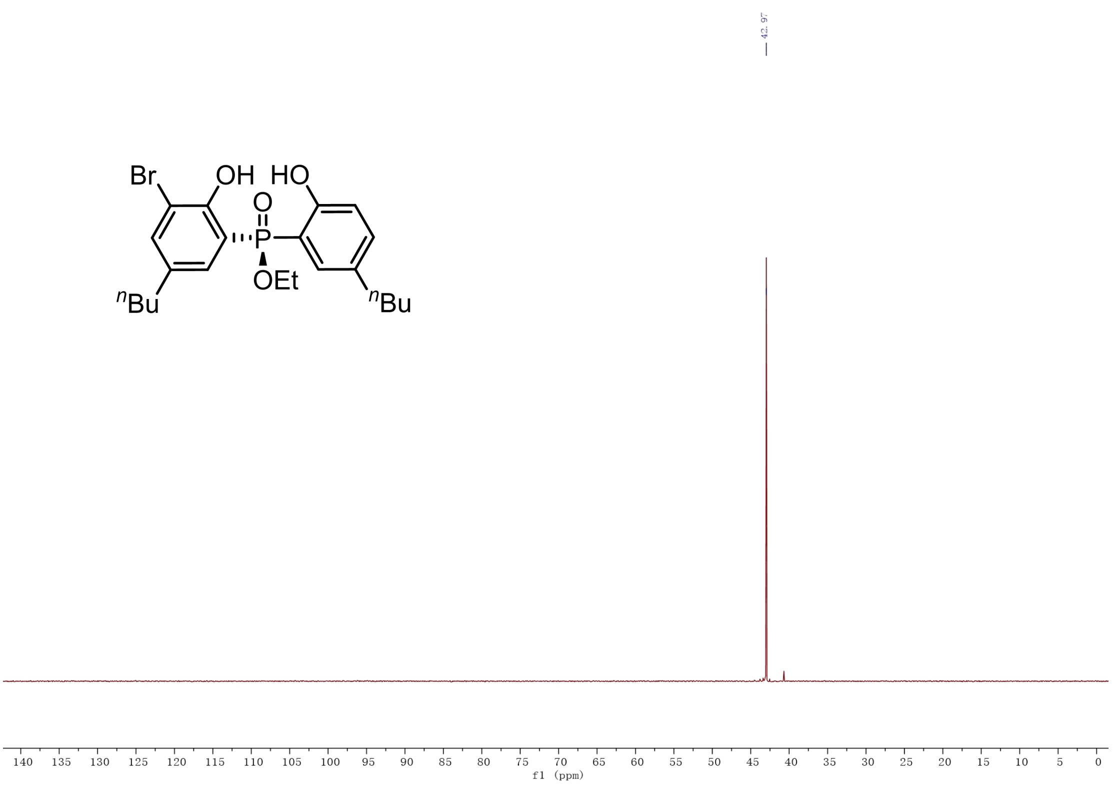

^{1}H NMR/^{13}C NMR/^{31}P NMR of product 3k

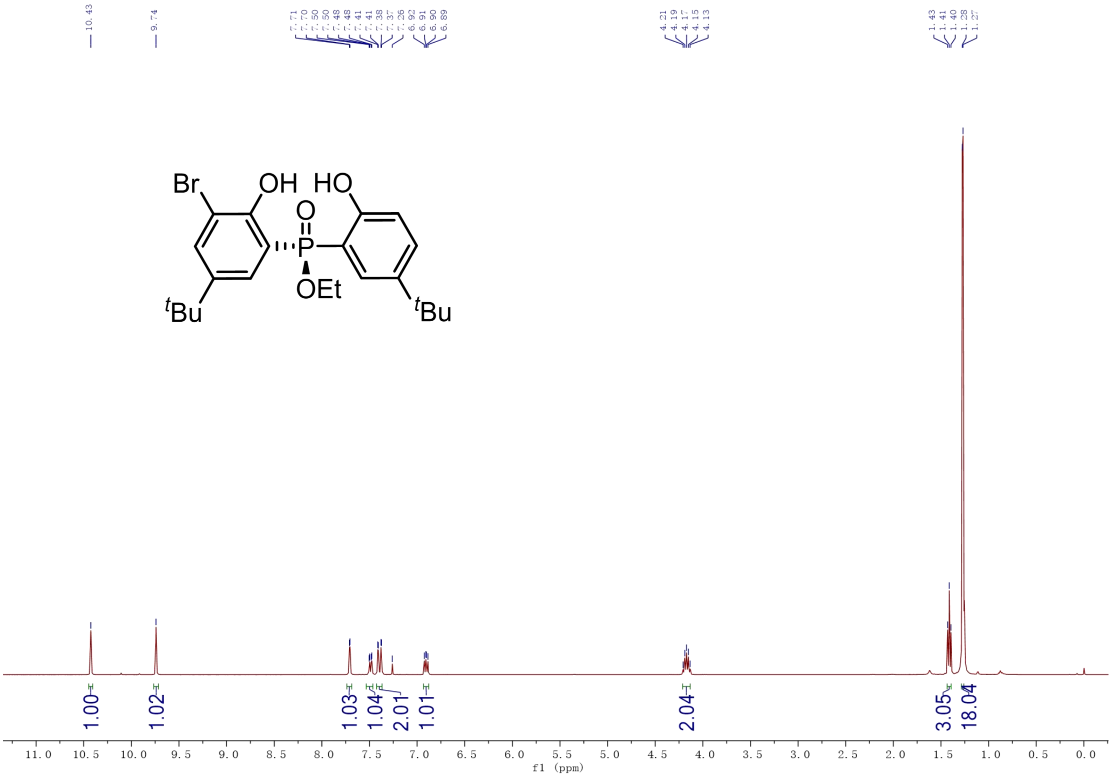

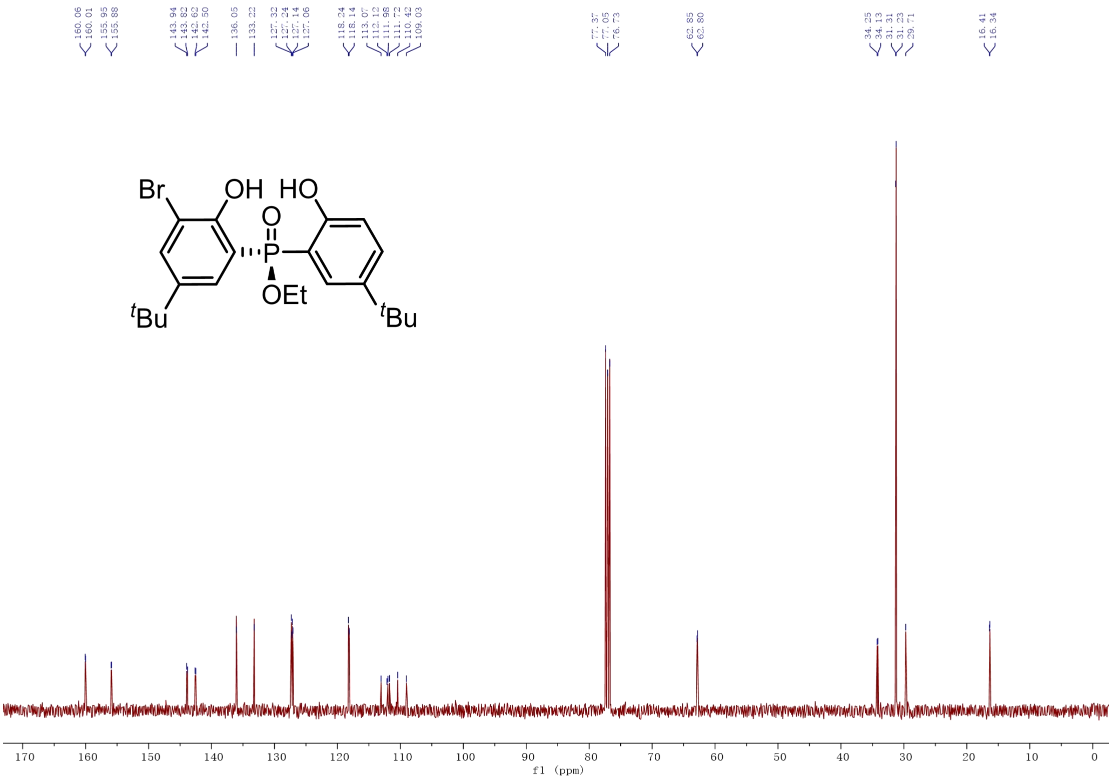
160.06
160.01
155.95
155.88
143.94
143.82
142.62
142.50
136.05
133.22
127.32
127.24
127.14
127.06
118.24
118.14
113.07
112.12
111.98
111.72
110.42
109.03
77.37
77.05
76.73
62.85
62.80
34.25
34.13
31.31
31.23
29.71
16.41
16.34
Br
OH
HO
O
P
OEt
tBu
tBu
f1 (ppm)

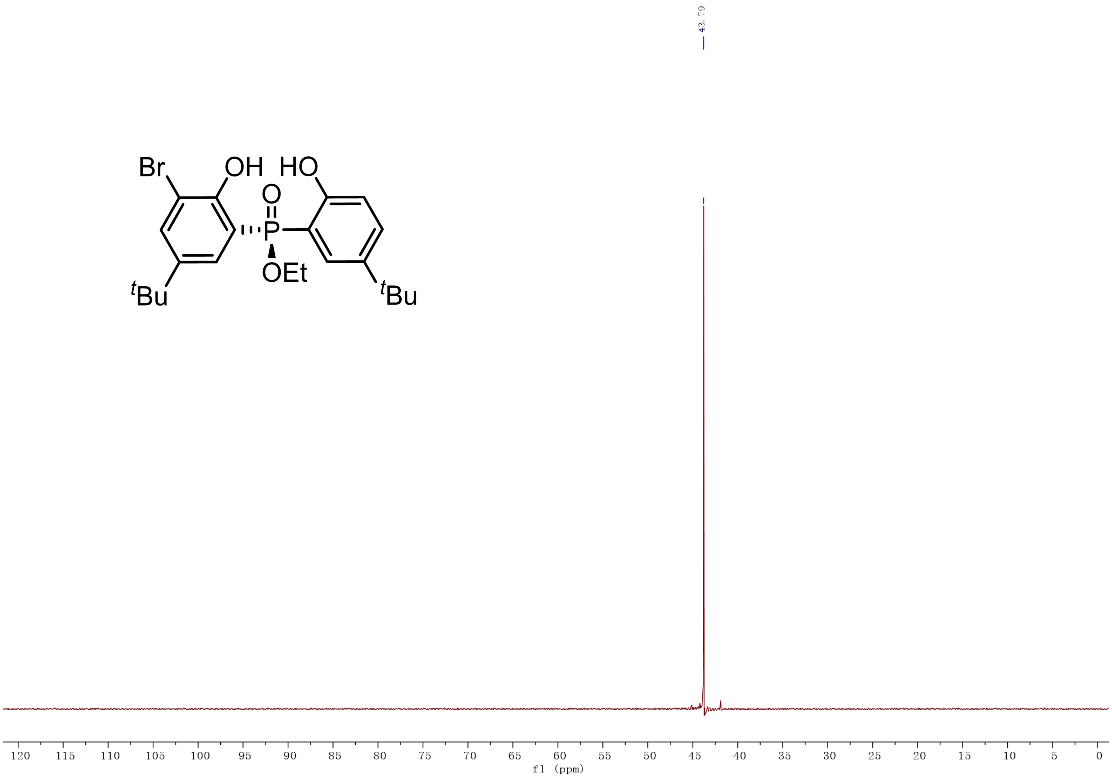
43.79
Br
OH
HO
O
P
OEt
tBu
tBu
f1 (ppm)

^{1}H NMR/^{13}C NMR/^{31}P NMR of product 3l

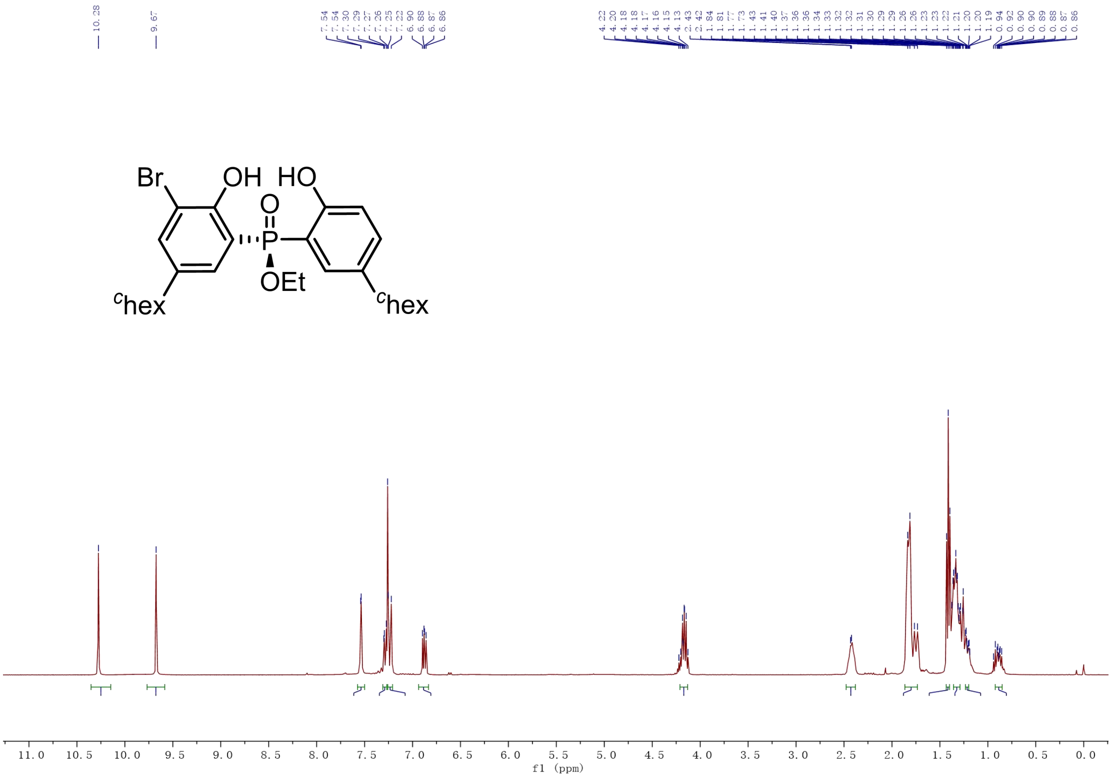

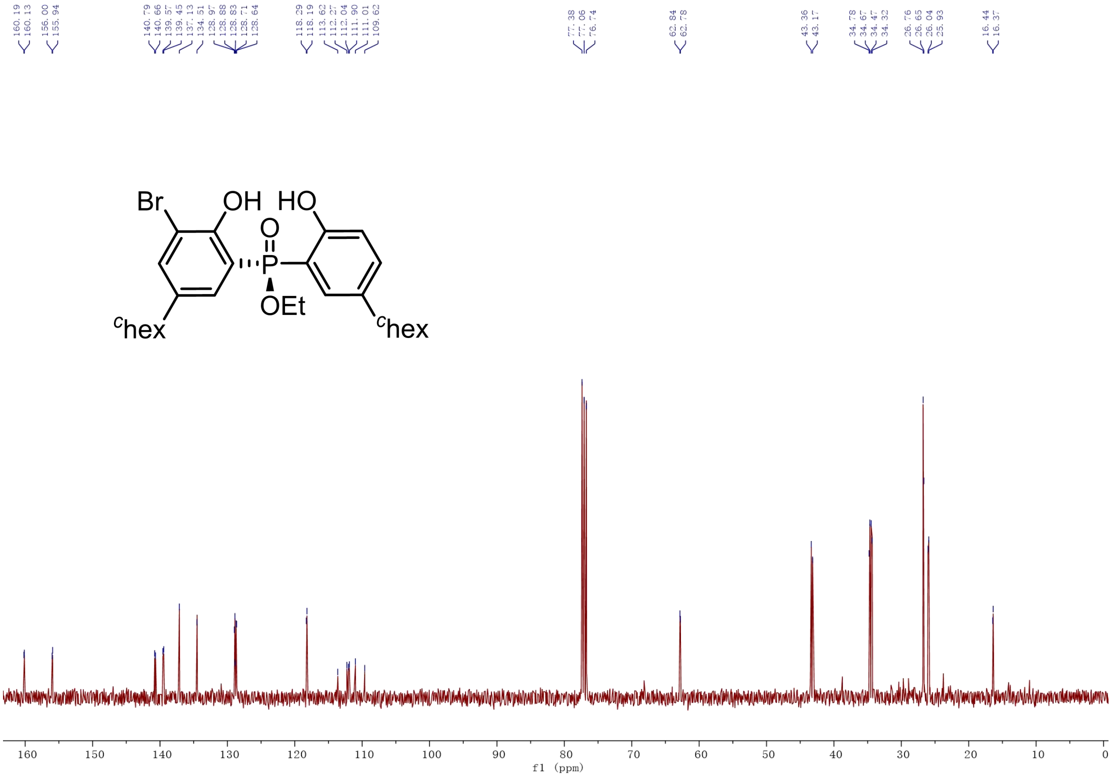

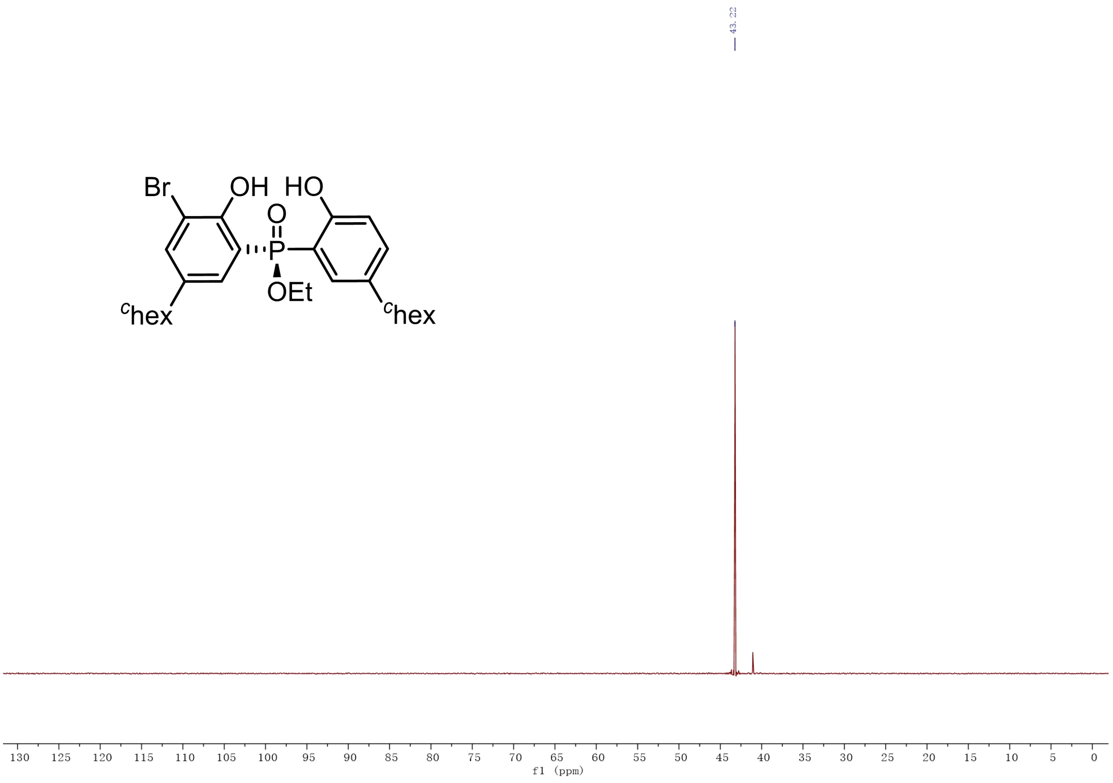

^{1}H NMR/^{13}C NMR/^{31}P NMR of product 3m

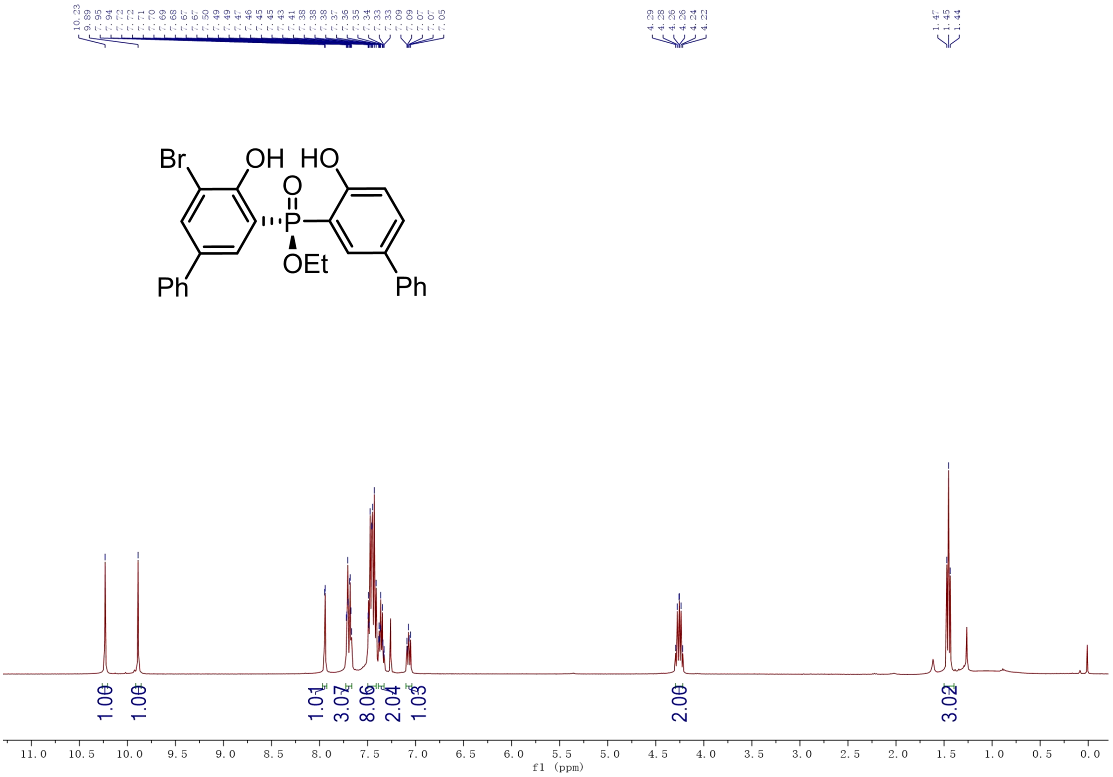

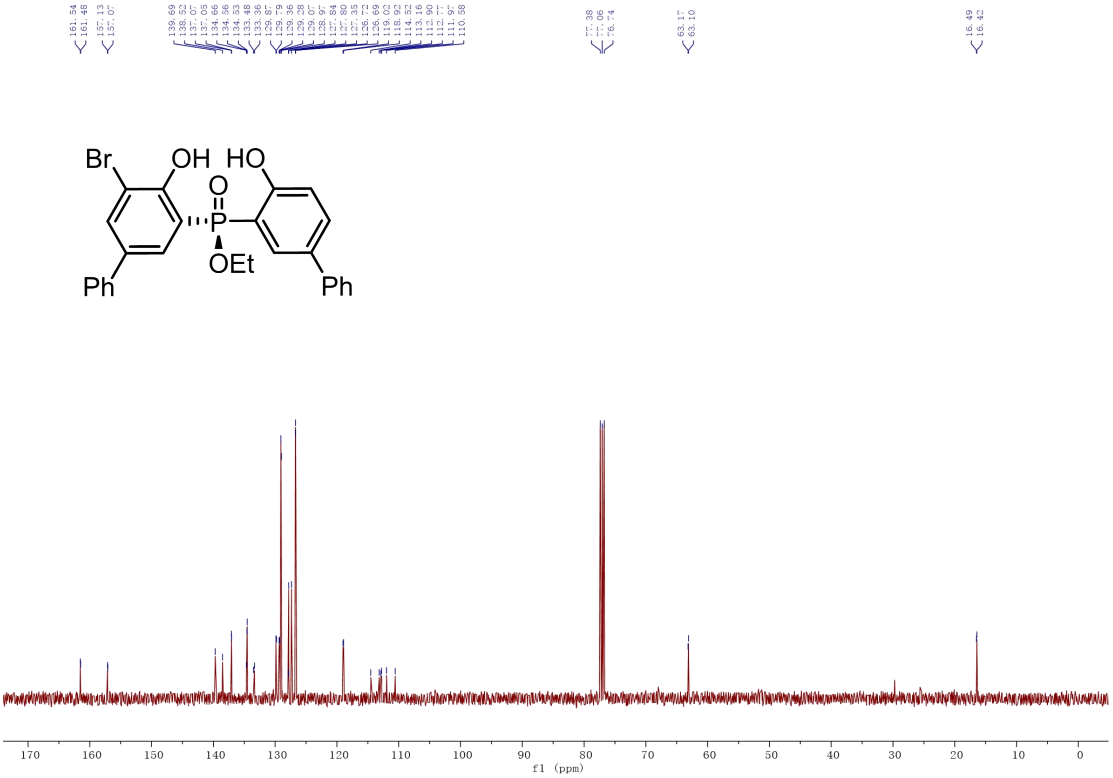
161.54
161.48
157.13
157.07
139.69
138.52
137.07
137.05
134.66
134.56
134.53
133.48
133.36
129.87
129.79
129.36
129.28
129.07
128.97
127.84
127.80
127.35
126.72
126.69
119.02
118.92
114.52
113.16
112.90
112.77
111.97
110.58
77.38
77.06
76.74
63.17
63.10
16.49
16.42
Br
OH
HO
O
P
OEt
Ph
Ph
f1 (ppm)

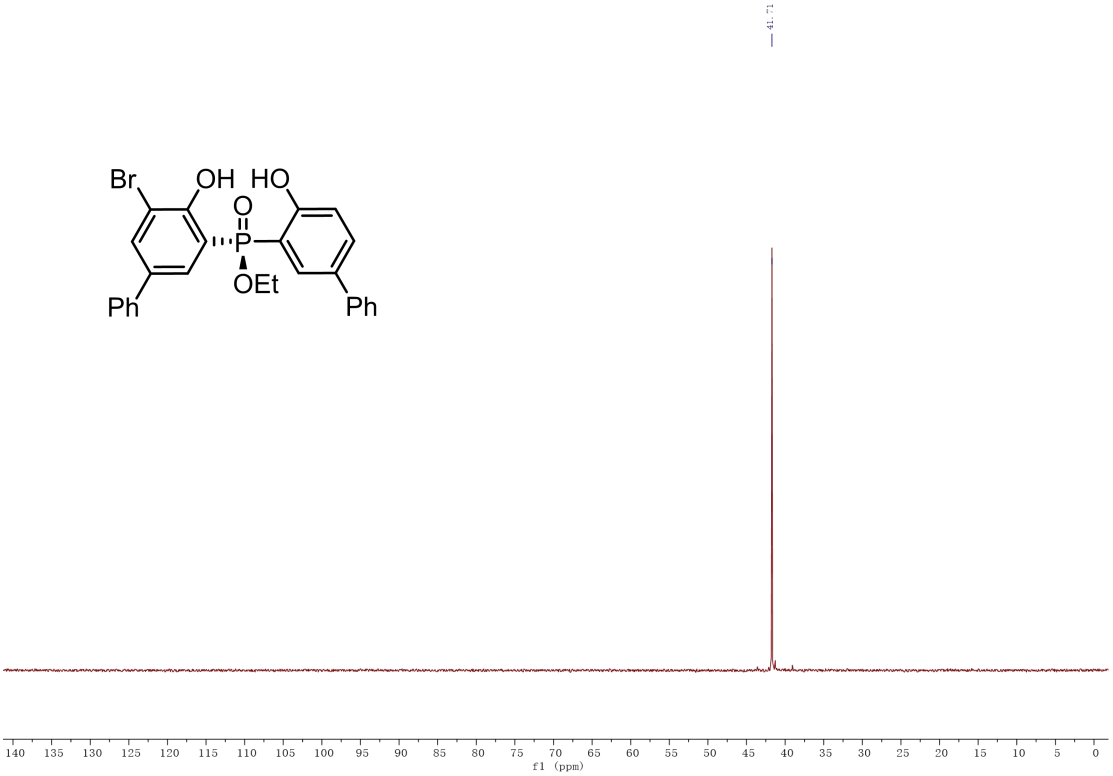
41.71
Br
OH
HO
O
P
OEt
Ph
Ph
f1 (ppm)

^{1}H NMR/^{13}C NMR/^{31}P NMR of product 3n

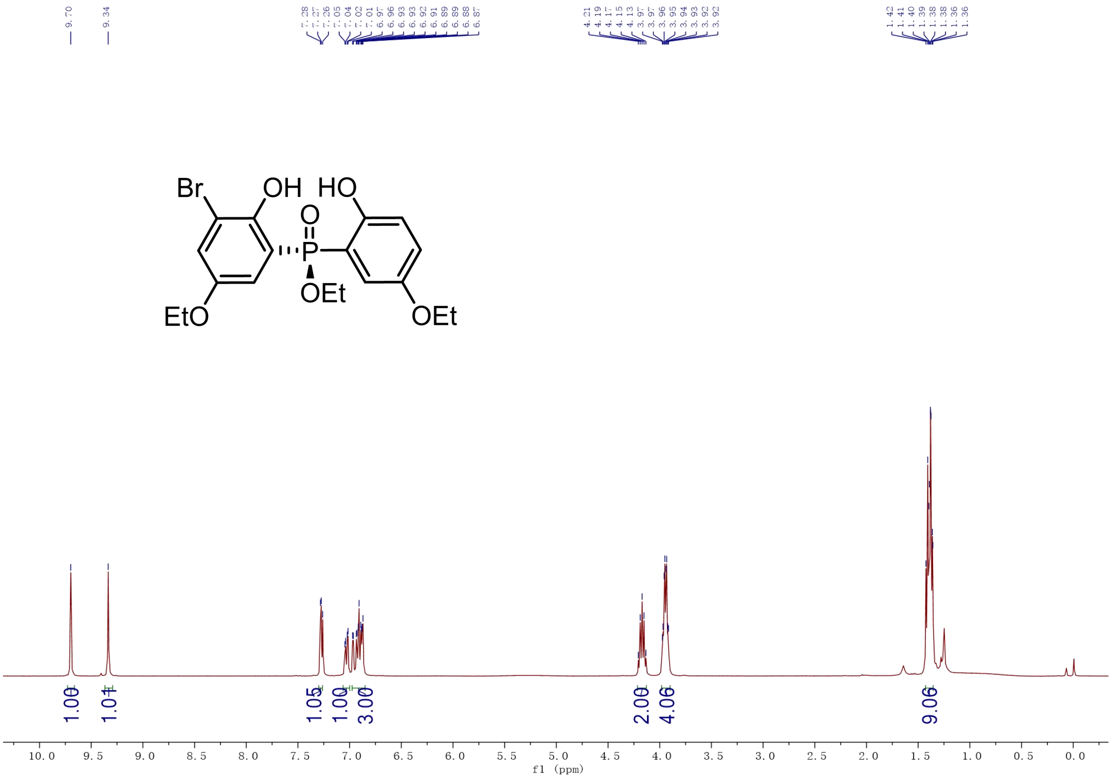

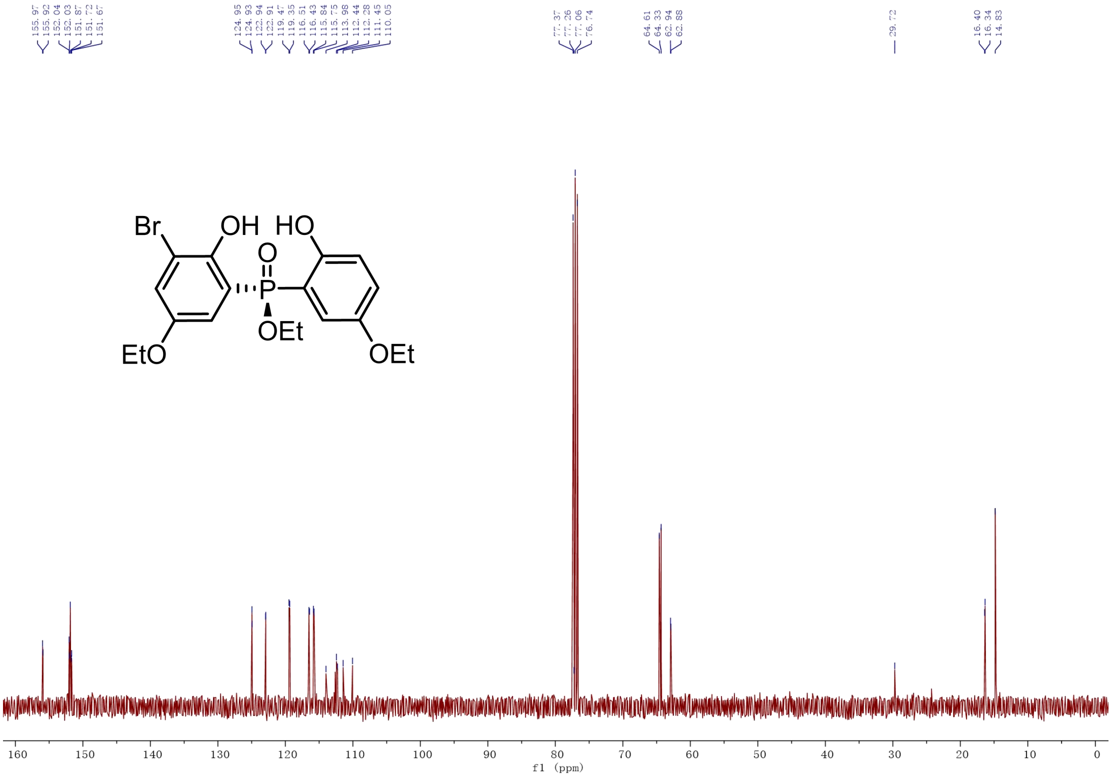

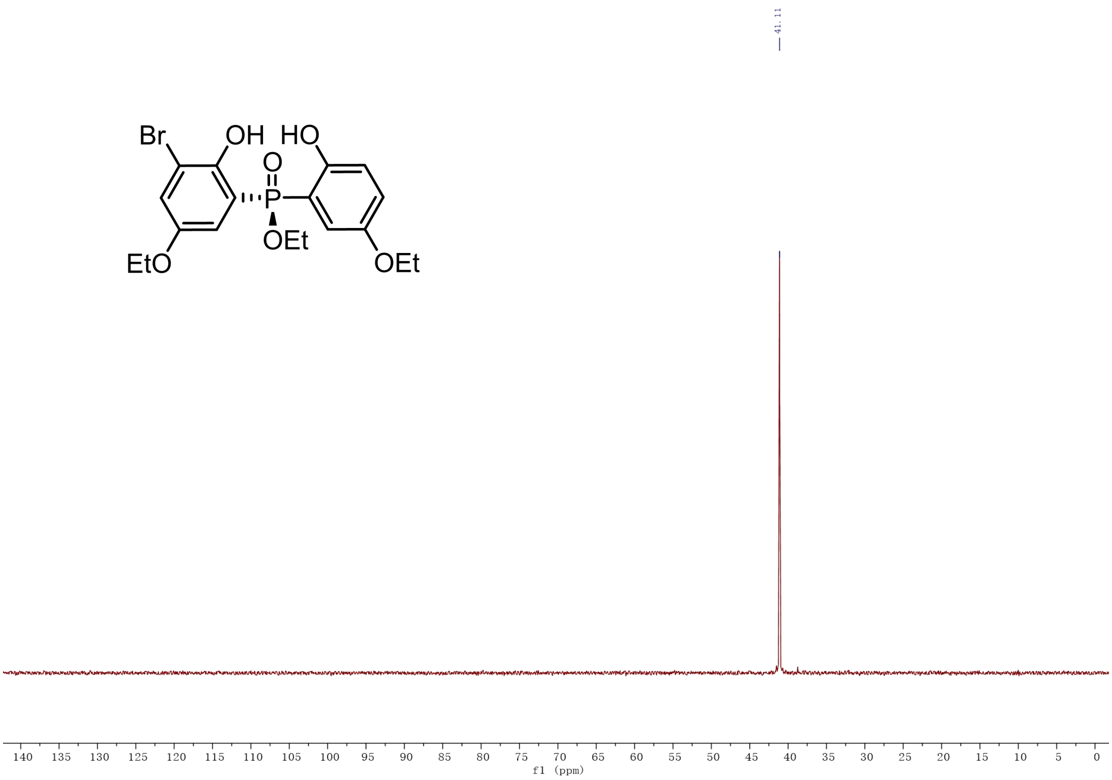

^{1}H NMR/^{13}C NMR/^{31}P NMR of product 3o

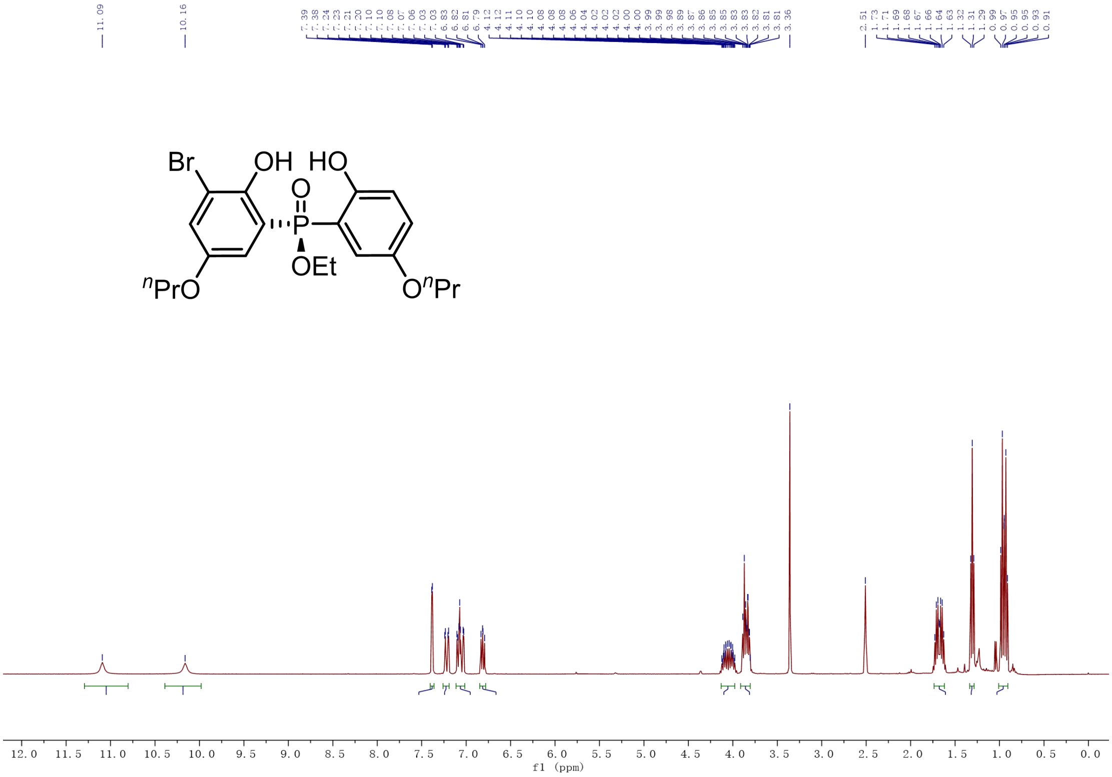

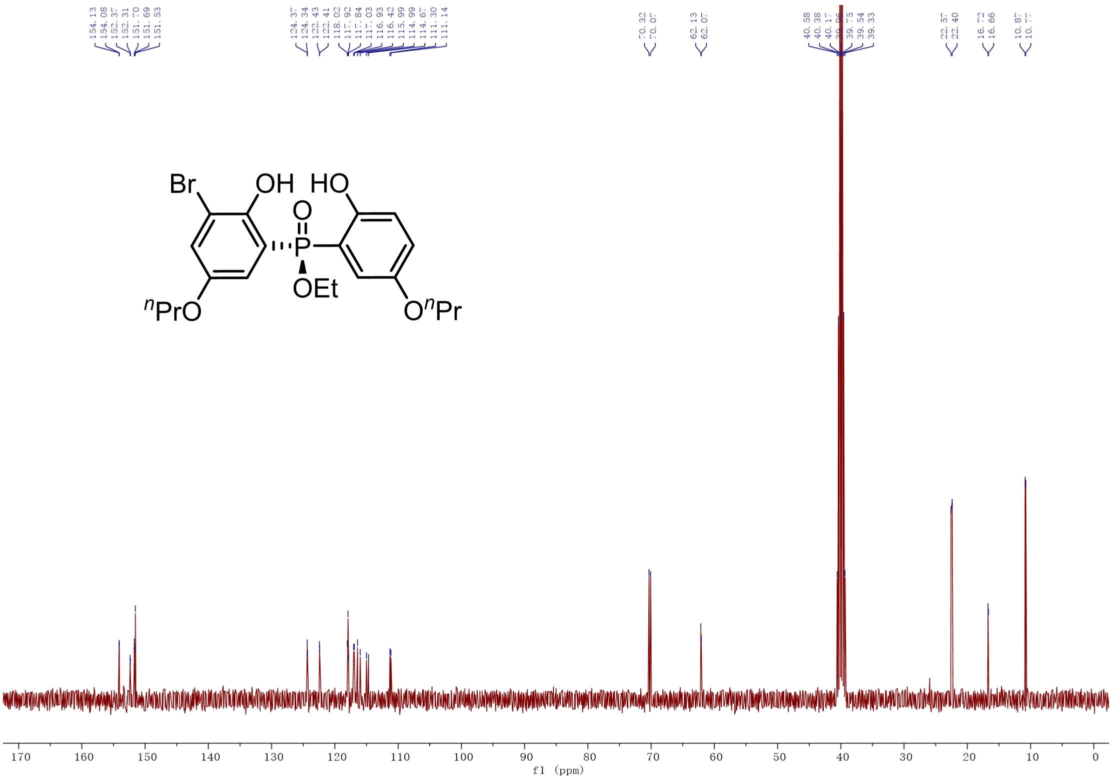
154.13
154.08
152.37
152.31
151.70
151.69
151.53
124.37
124.34
122.43
122.41
118.02
117.92
117.84
117.03
116.93
116.42
115.99
114.99
114.67
111.30
111.14
70.32
70.07
62.13
62.07
40.58
40.38
40.17
39.96
39.75
39.54
39.33
22.57
22.40
16.72
16.66
10.87
10.77
Br
OH
HO
O
P
OEt
nPrO
OnPr
f1 (ppm)

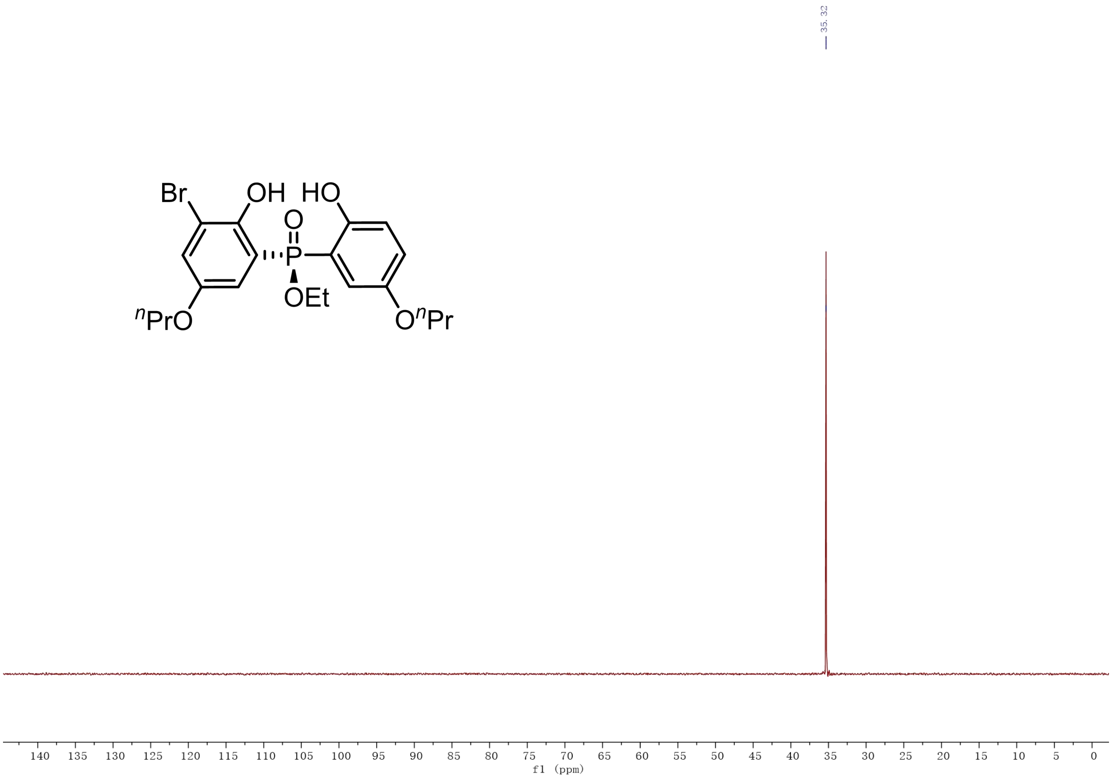
35.32
Br
OH
HO
O
P
OEt
nPrO
OnPr
f1 (ppm)

^{1}H NMR/^{13}C NMR/^{31}P NMR of product 3p

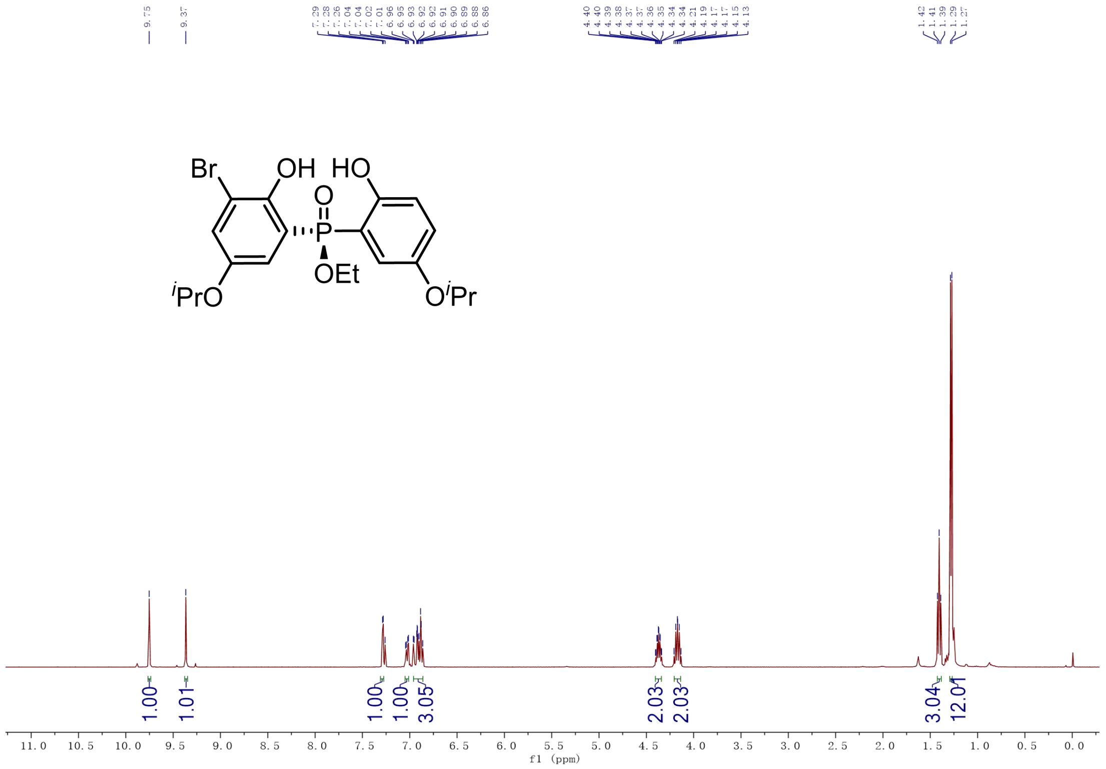

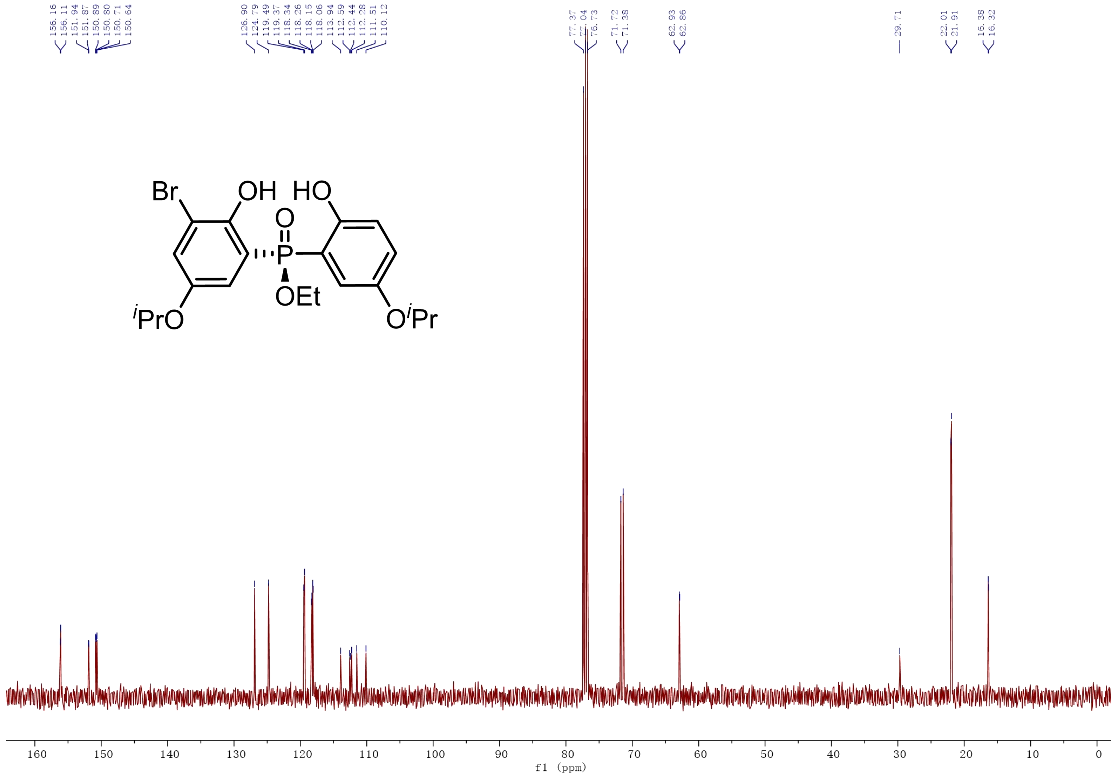

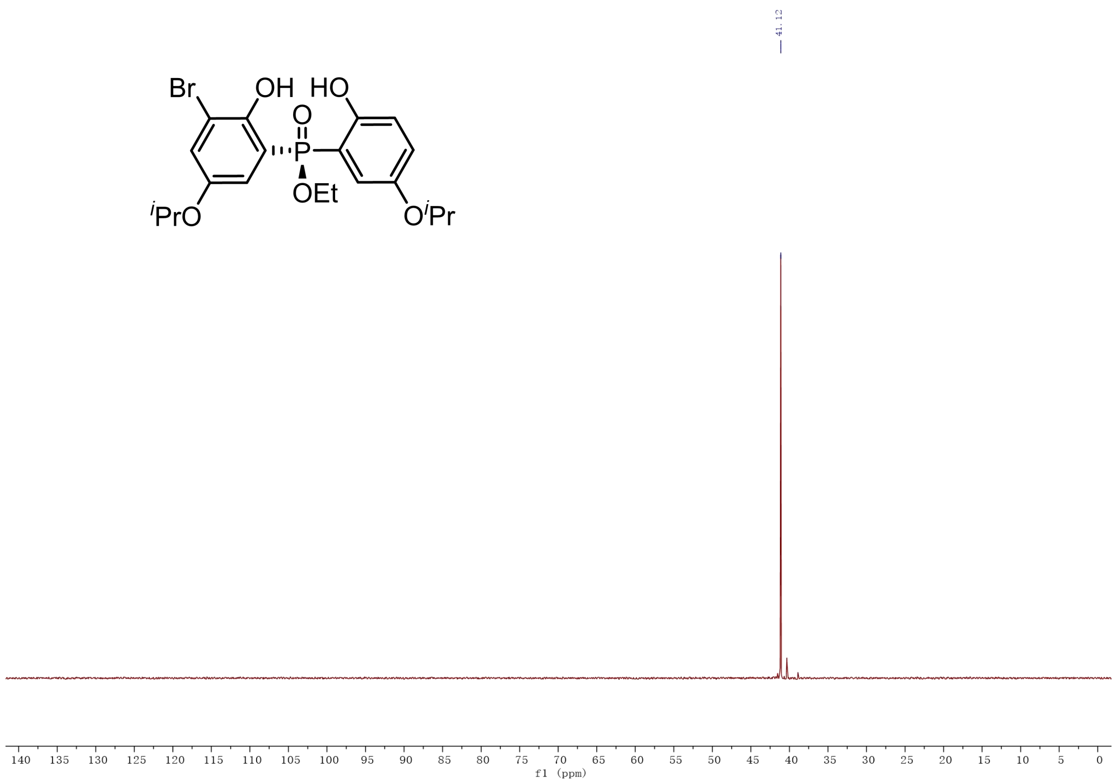

41.12
Br
OH
HO
O
P
OEt
iPrO
OiPr
140 135 130 125 120 115 110 105 100 95 90 85 80 75 70 65 60 55 50 45 40 35 30 25 20 15 10 5 0
f1 (ppm)

^{1}H NMR/^{13}C NMR/^{31}P NMR of product 3q

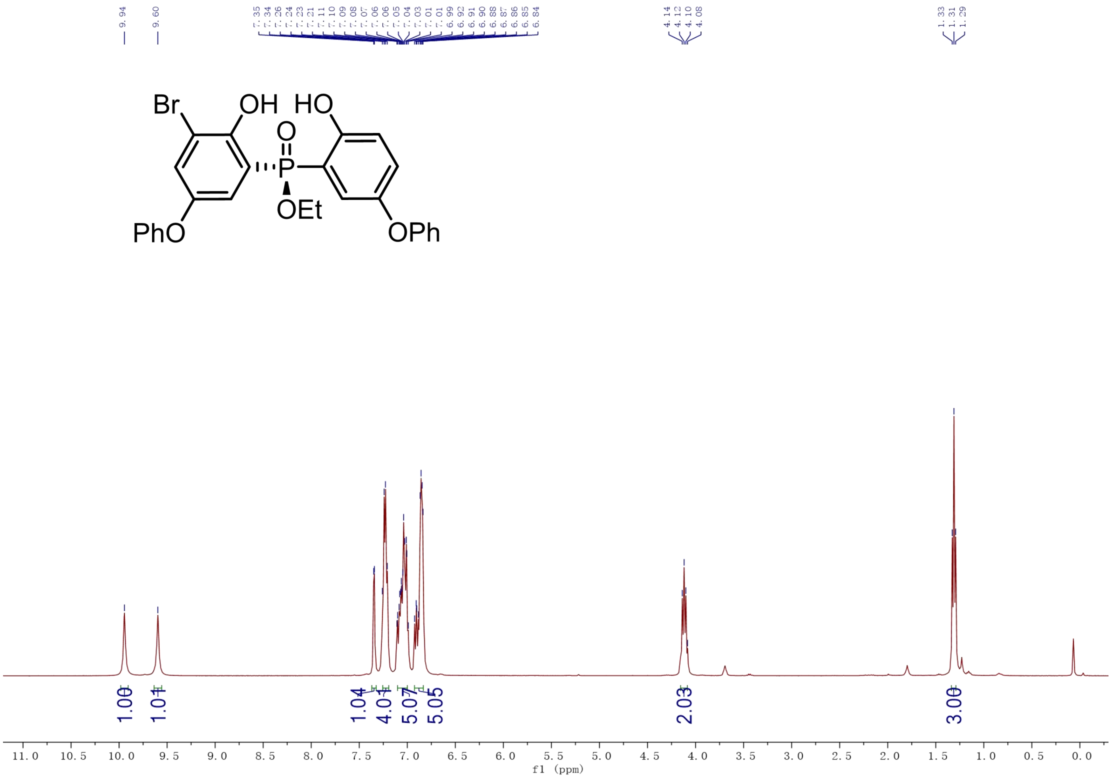

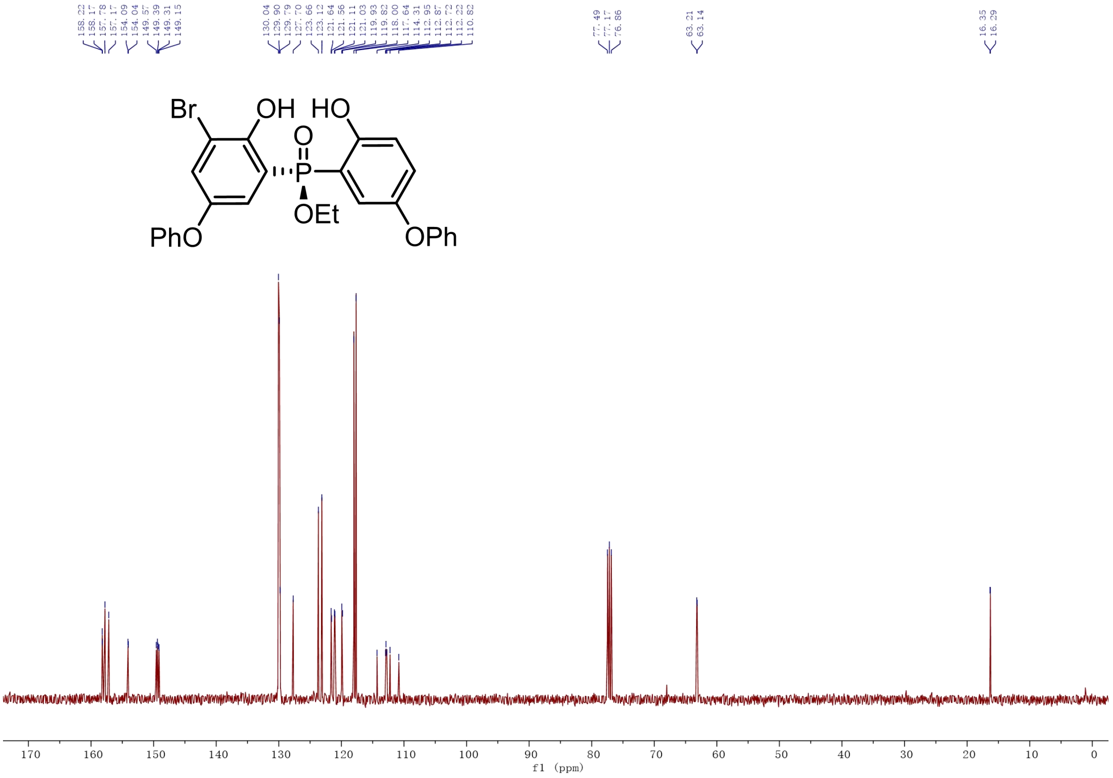

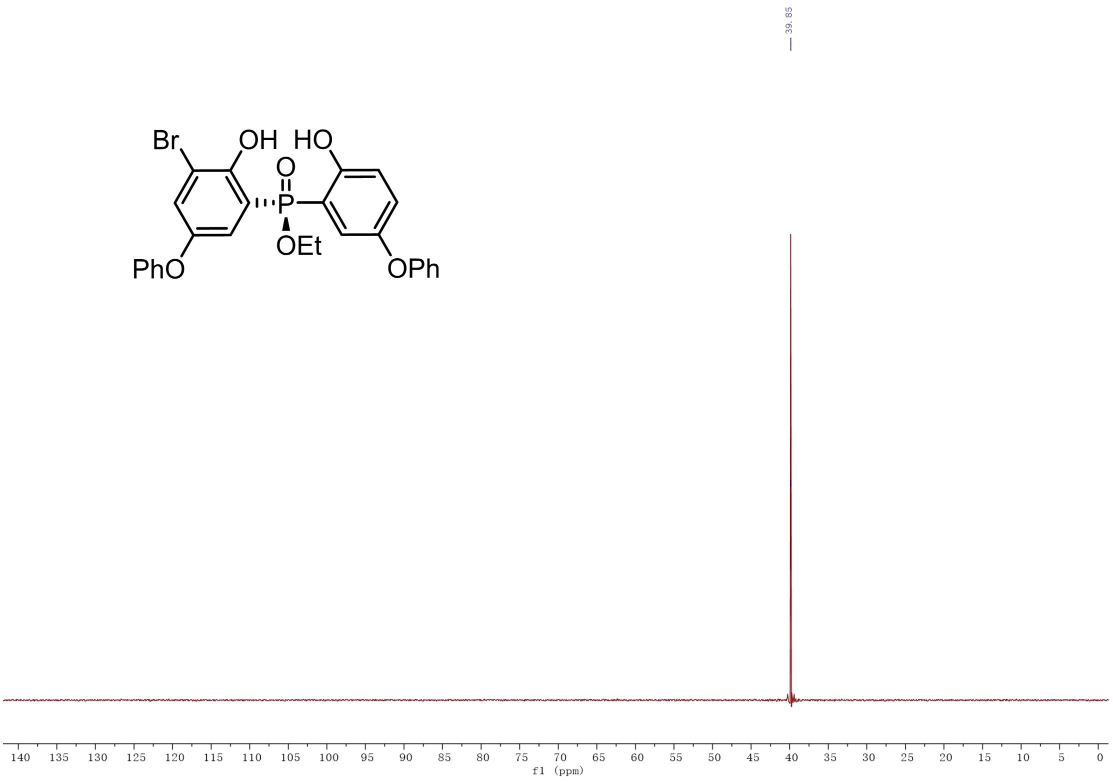

^{1}H NMR/^{13}C NMR/^{31}P NMR of product 3r

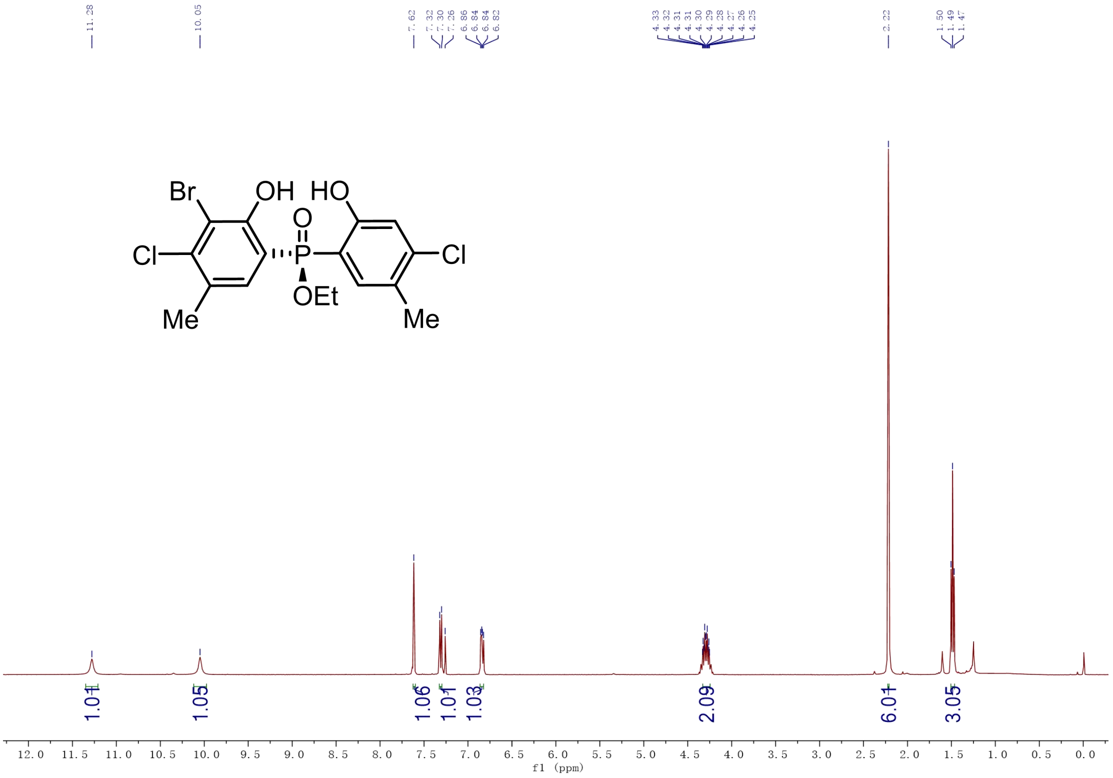

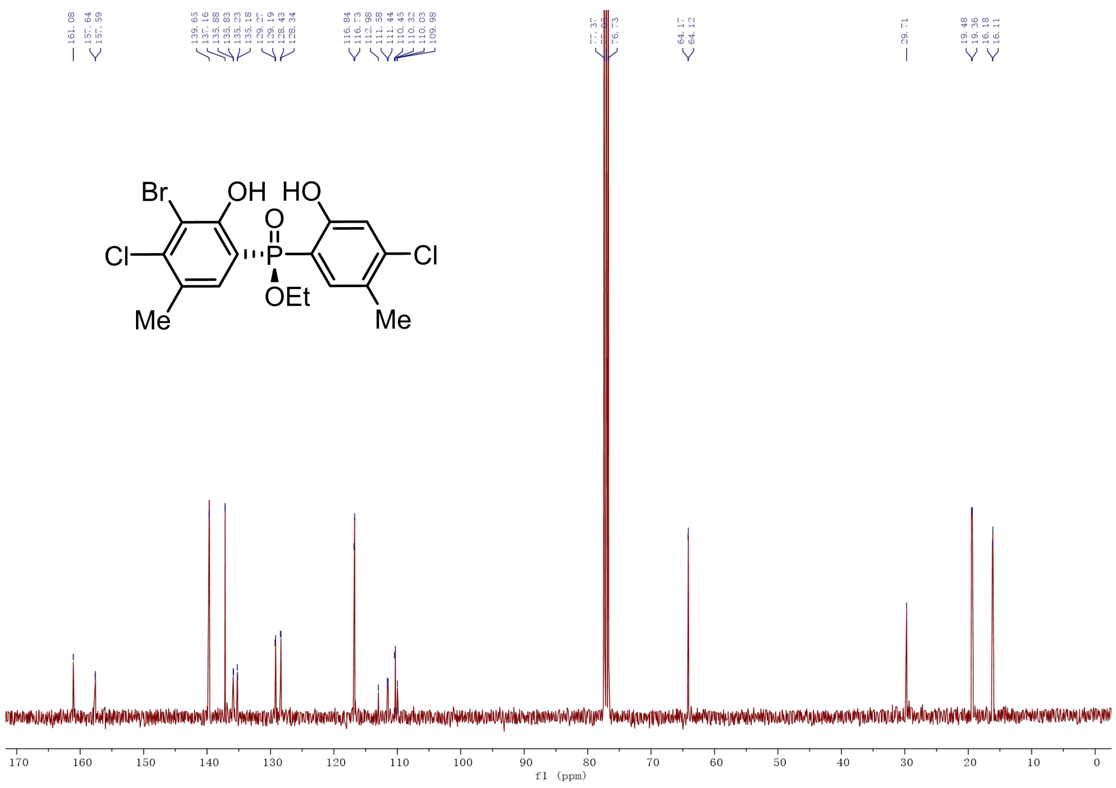

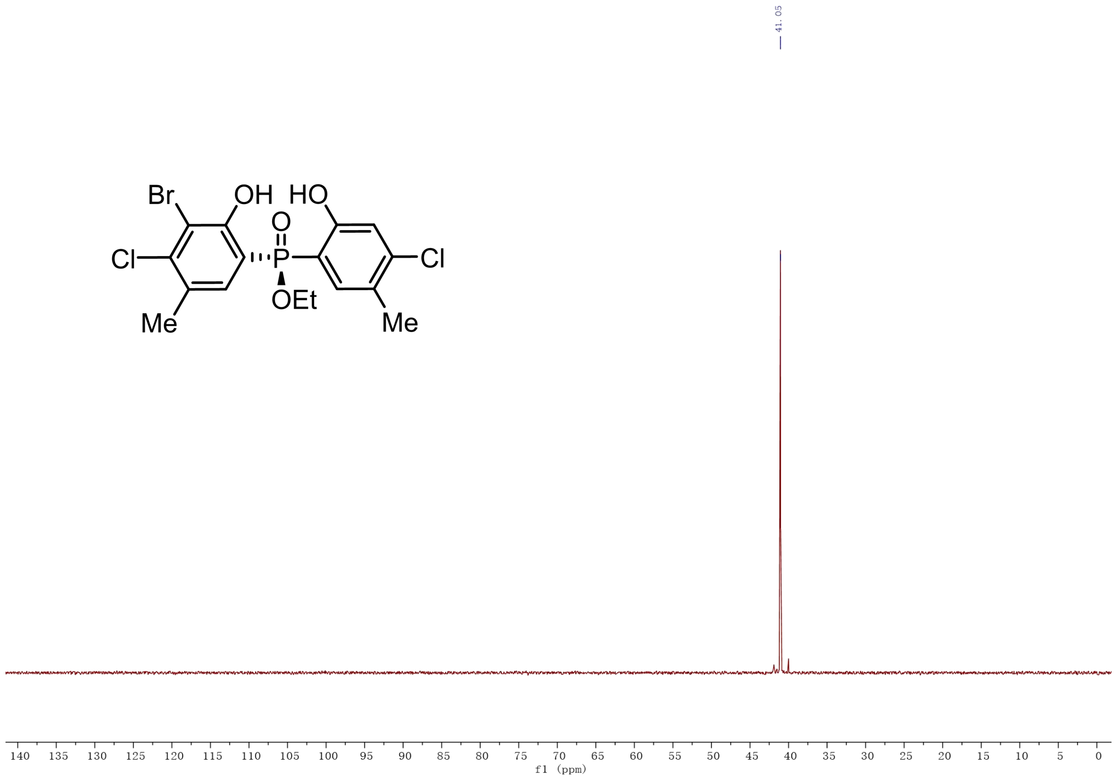

^{1}H NMR/^{13}C NMR/^{31}P NMR of product 3s

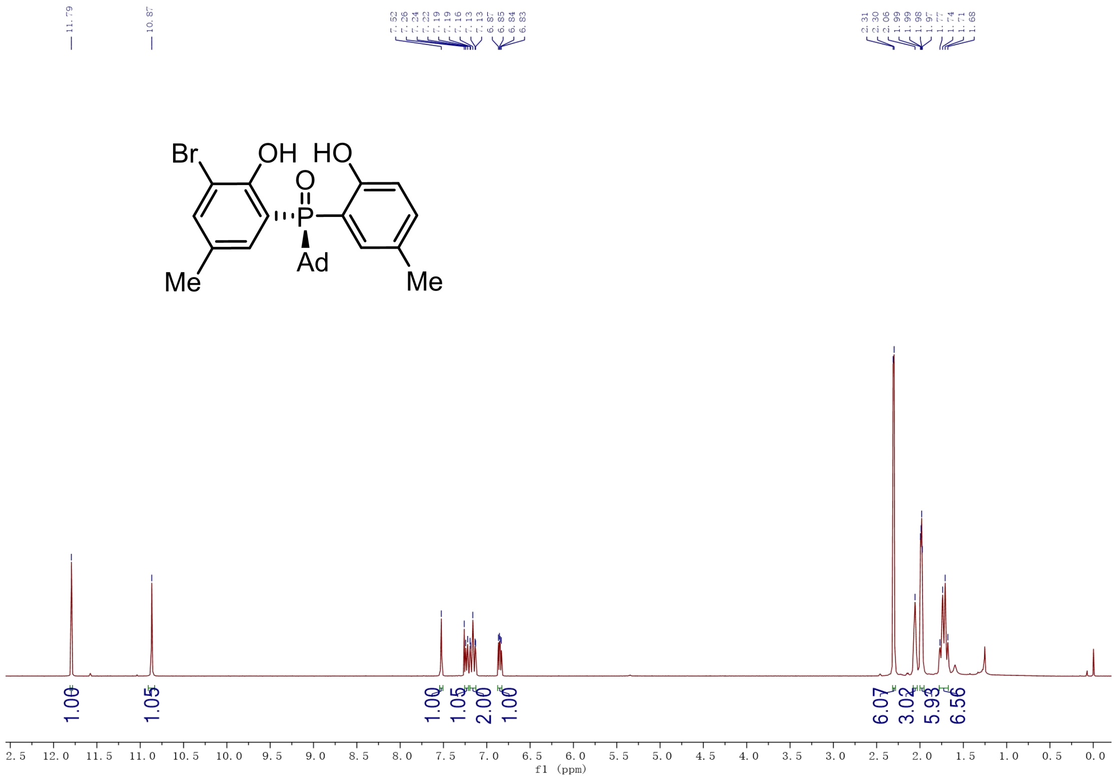

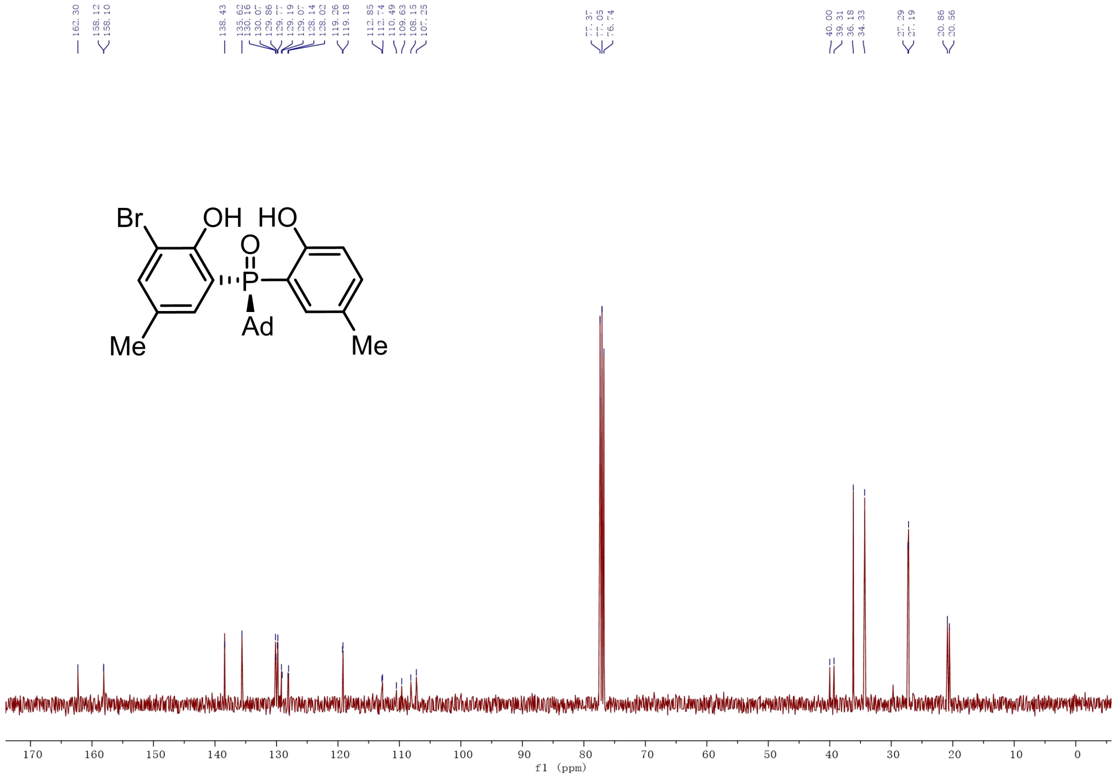

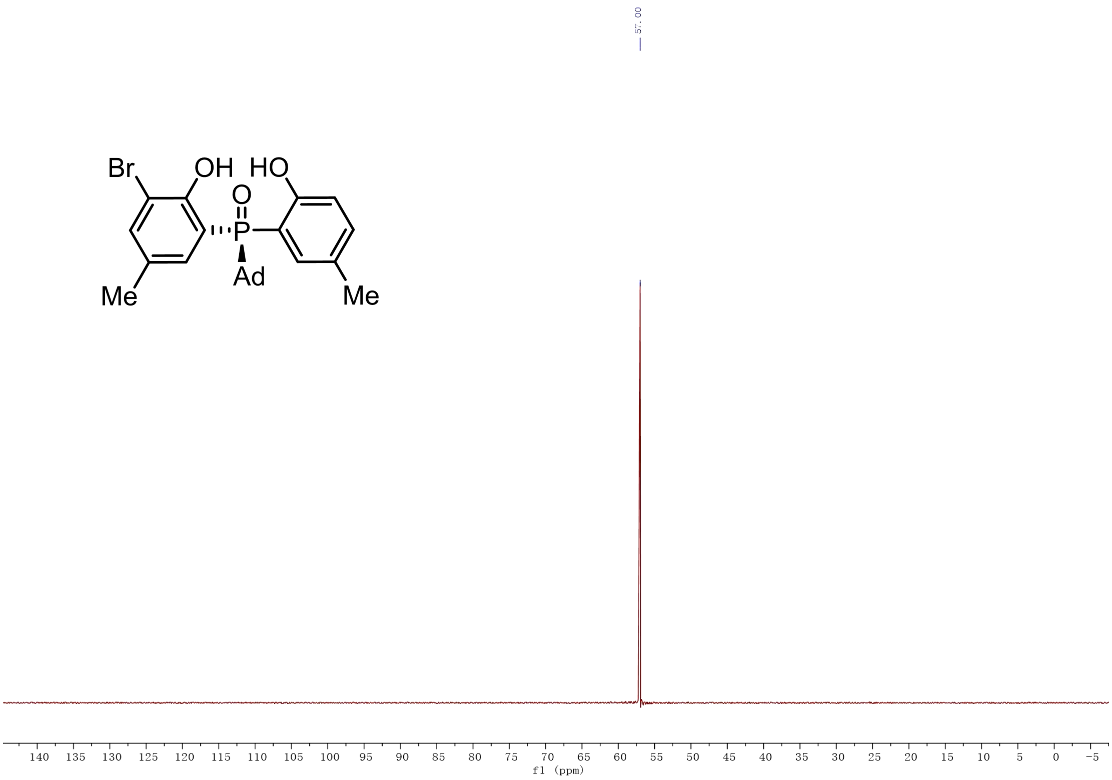

^{1}H NMR/^{13}C NMR/^{31}P NMR of product 3t

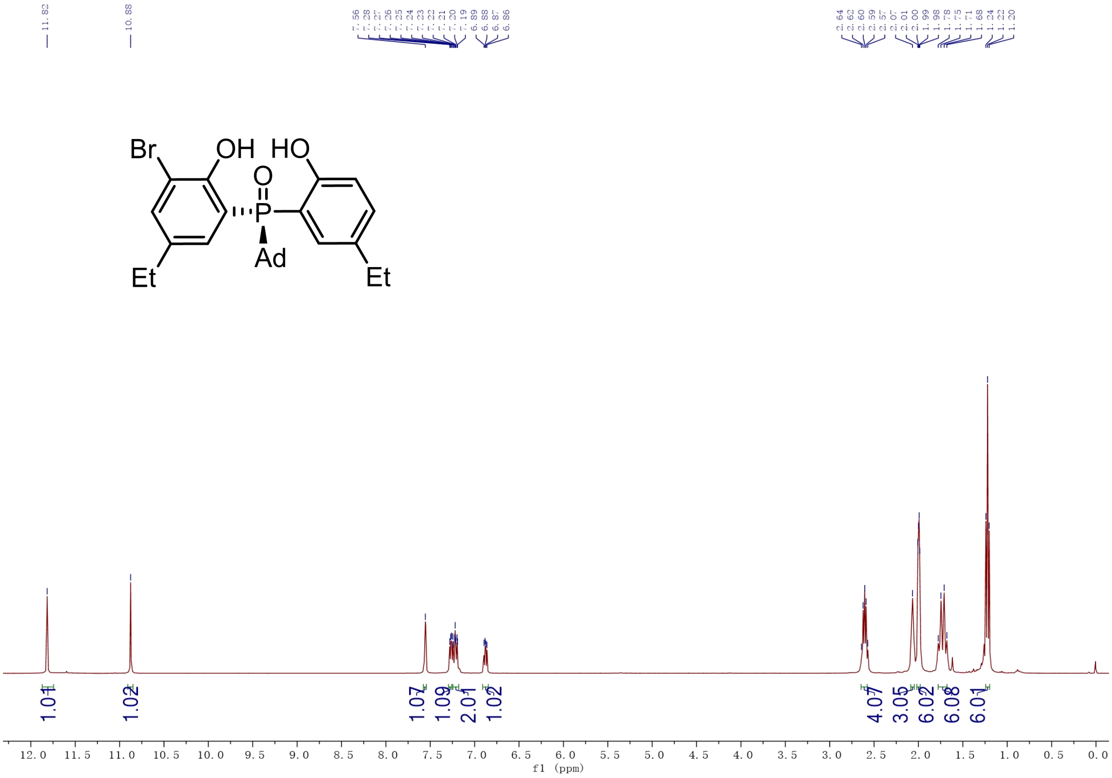

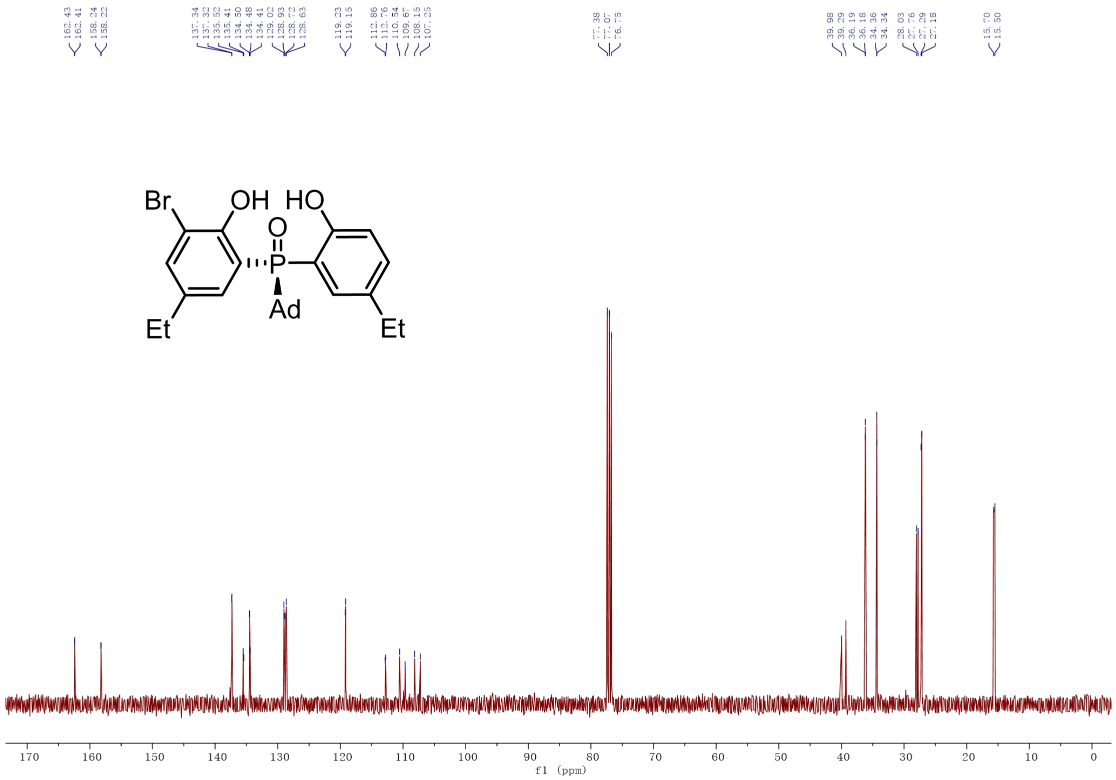
162.43
162.41
158.24
158.22
137.34
137.32
135.52
135.41
134.50
134.48
134.41
129.02
128.93
128.72
128.63
119.23
119.15
112.86
112.76
110.54
109.67
108.15
107.25
77.38
77.07
76.75
39.98
39.29
36.19
36.18
34.36
34.34
28.03
27.76
27.29
27.18
15.70
15.50
Br
OH
HO
O
P
Ad
Et
Et
f1 (ppm)

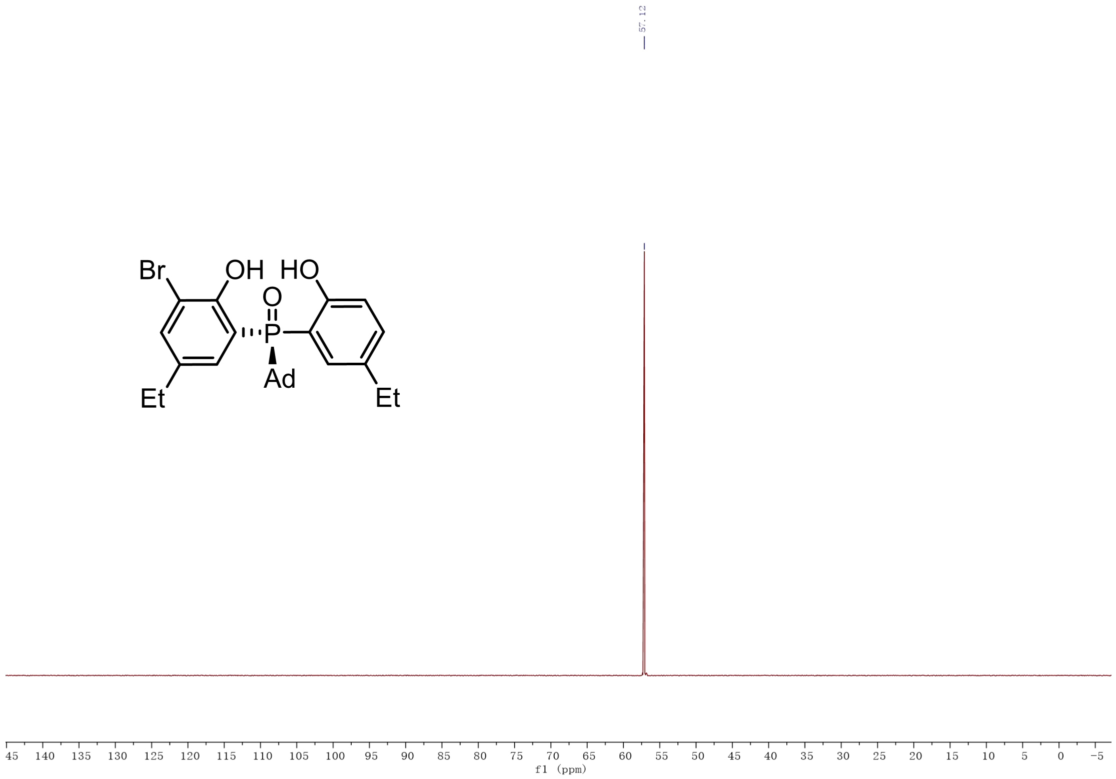
57.12
Br
OH
HO
O
P
Ad
Et
Et
f1 (ppm)

^{1}H NMR/^{13}C NMR/^{31}P NMR of product 3u

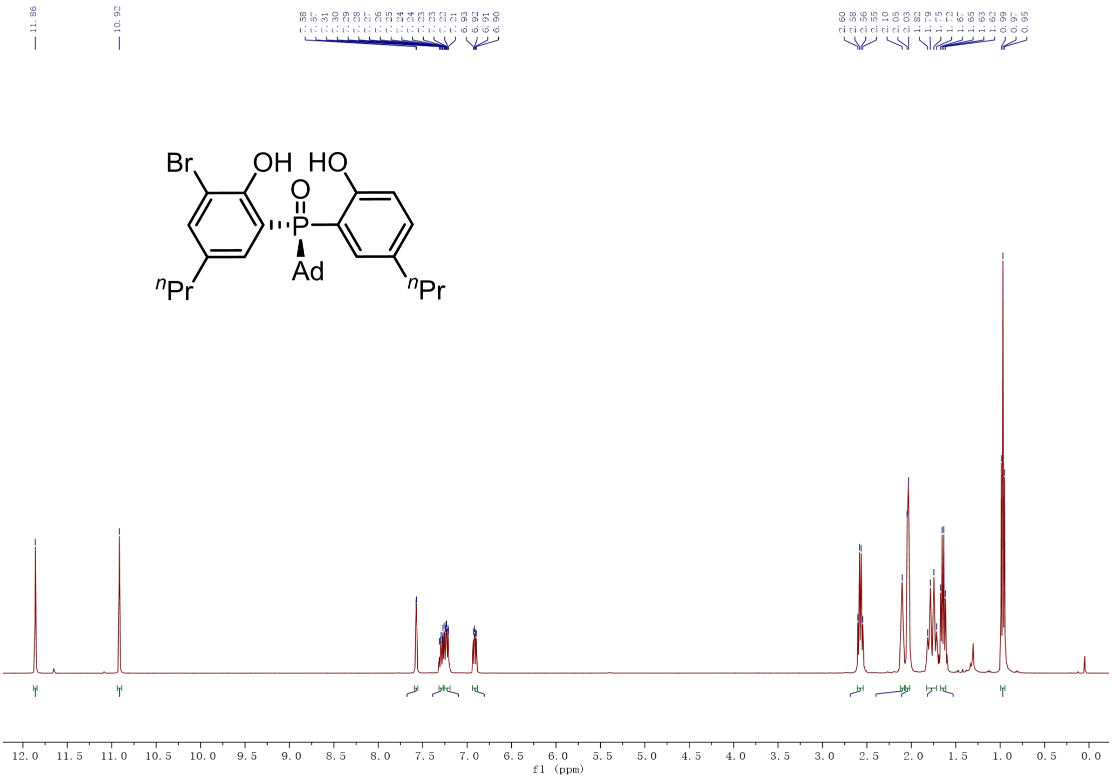

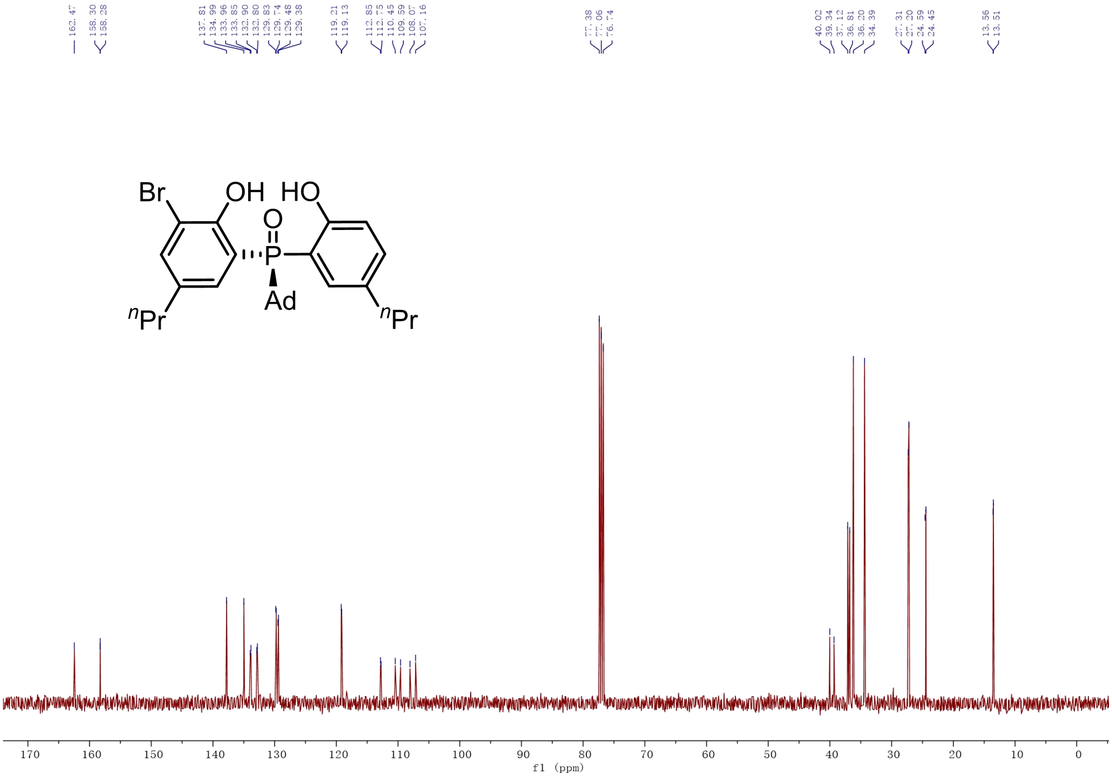

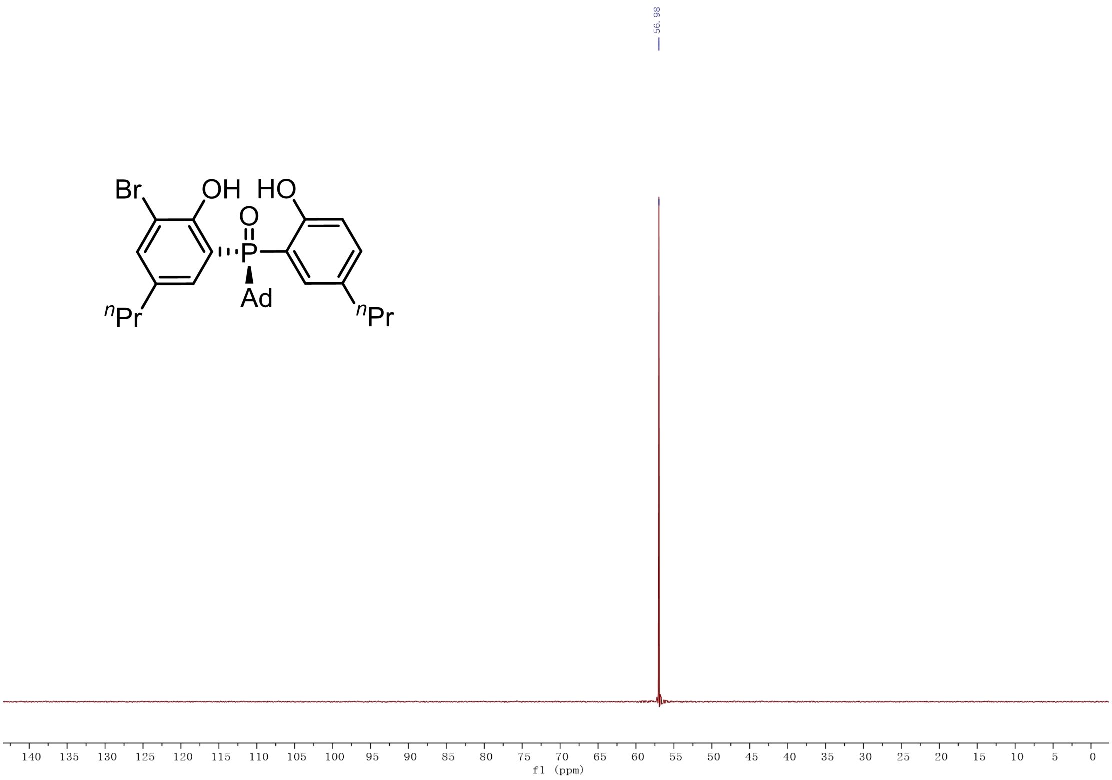

^{1}H NMR/^{13}C NMR/^{31}P NMR of product 3v

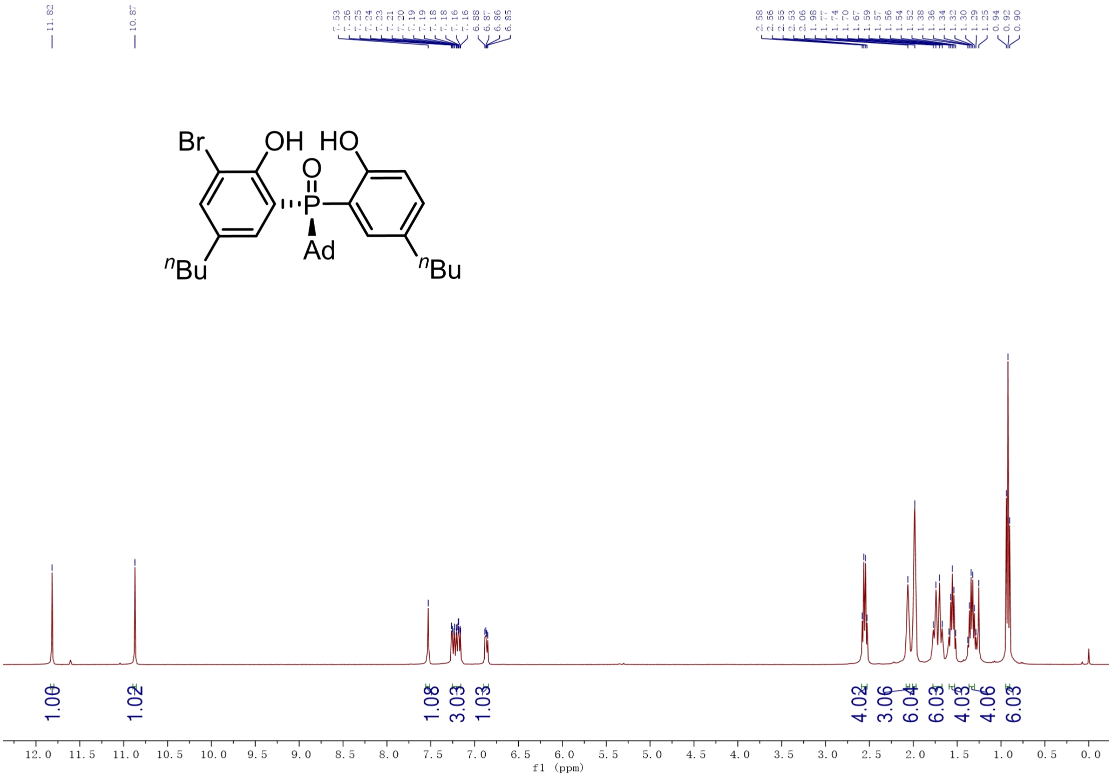

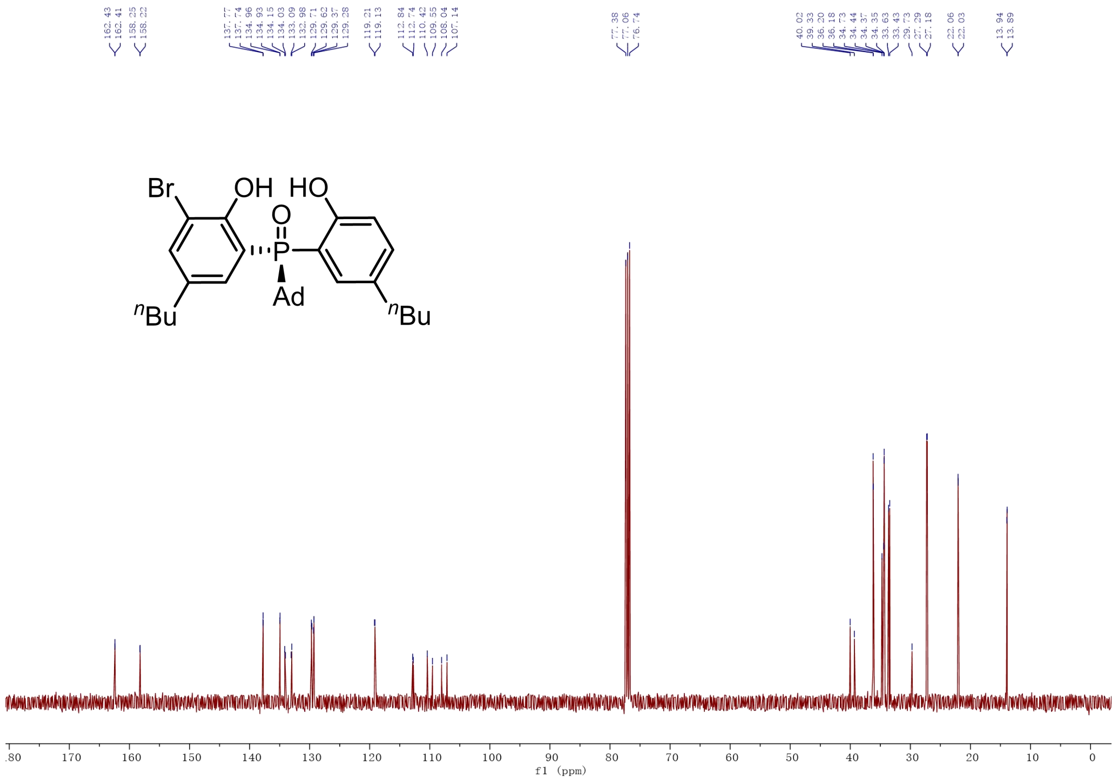
162.43
162.41
158.25
158.22
137.77
137.74
134.96
134.93
134.15
134.03
133.09
132.98
129.71
129.62
129.37
129.28
119.21
119.13
112.84
112.74
110.42
109.55
108.04
107.14
77.38
77.06
76.74
40.02
39.33
36.20
36.18
34.73
34.44
34.37
34.35
33.63
33.43
29.73
27.29
27.18
22.06
22.03
13.94
13.89
Br
OH
HO
O
P
Ad
nBu
nBu
f1 (ppm)

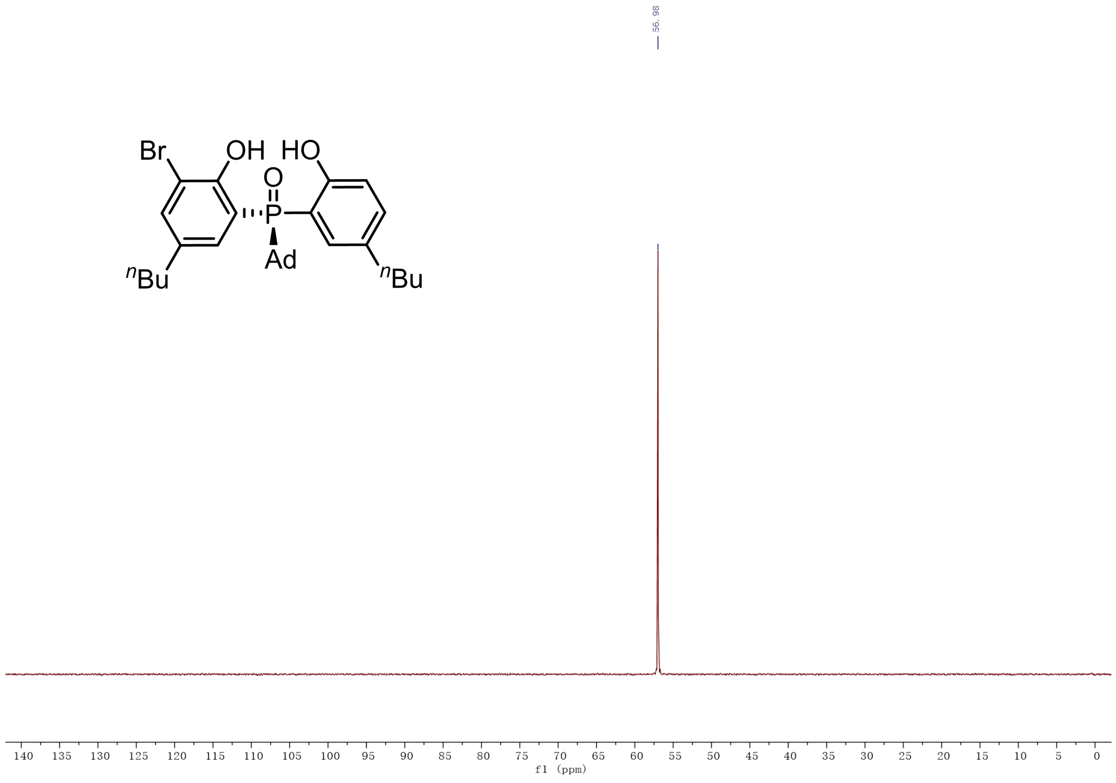
56.98
Br
OH
HO
O
P
Ad
nBu
nBu
f1 (ppm)

^{1}H NMR/^{13}C NMR/^{31}P NMR of product 3w

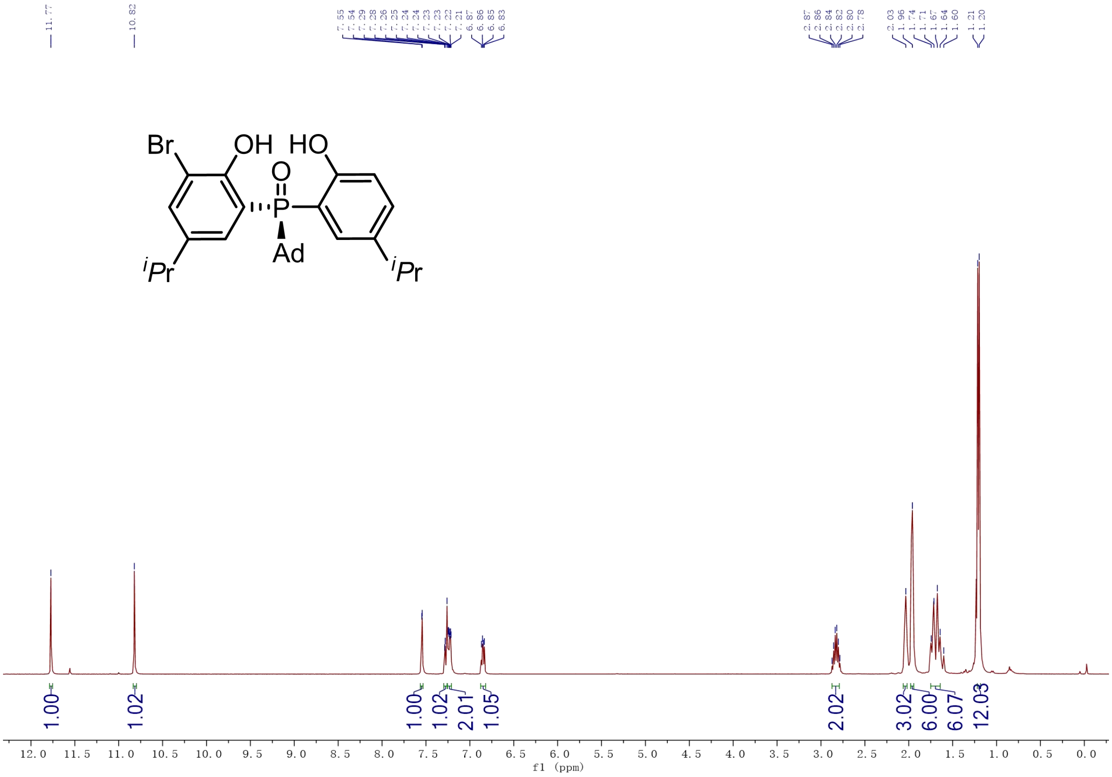

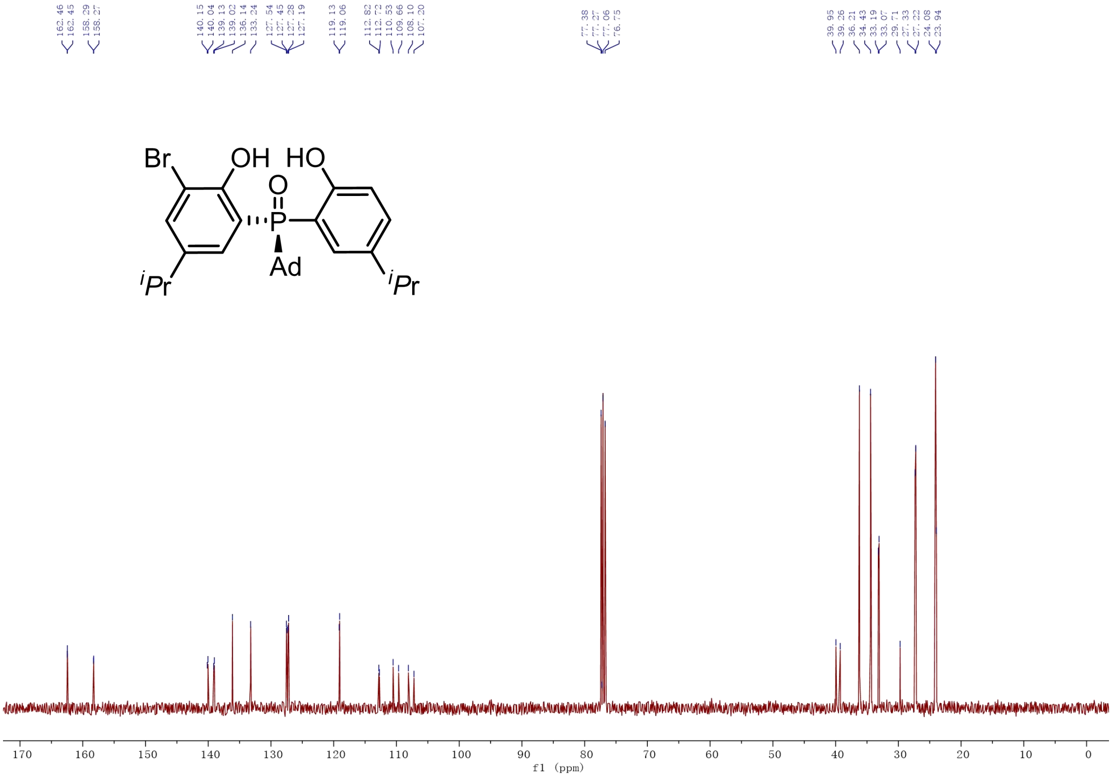

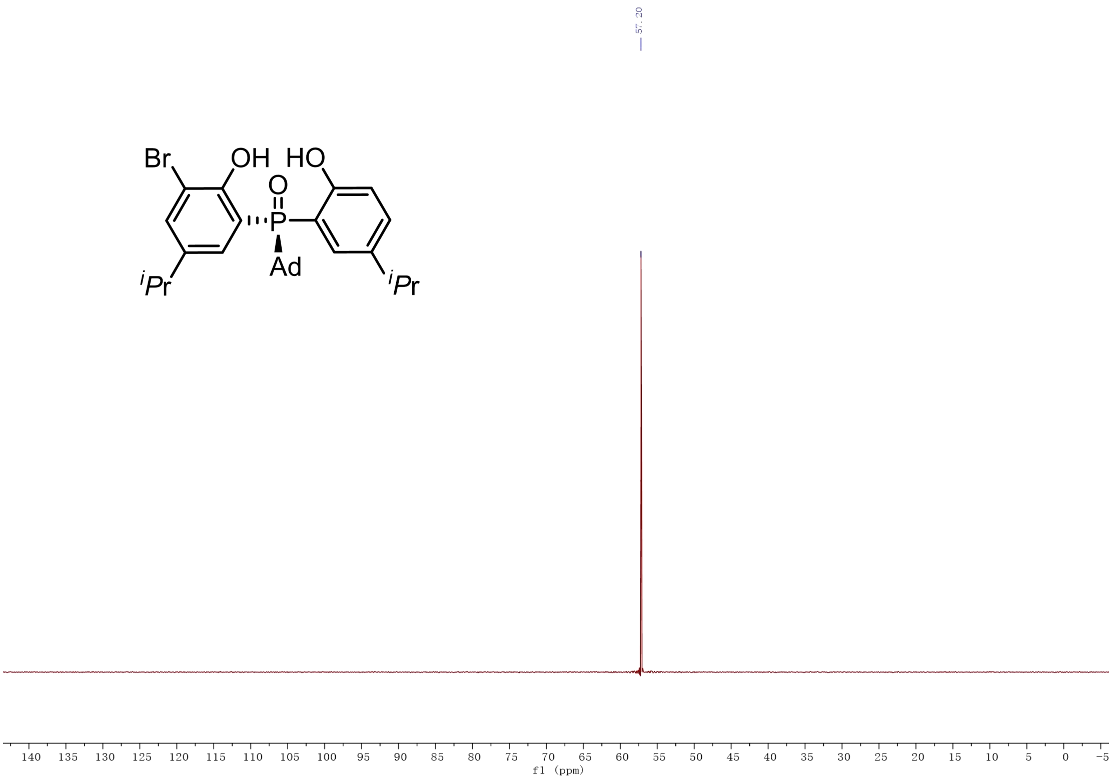

^{1}H NMR/^{13}C NMR/^{31}P NMR of product 3x

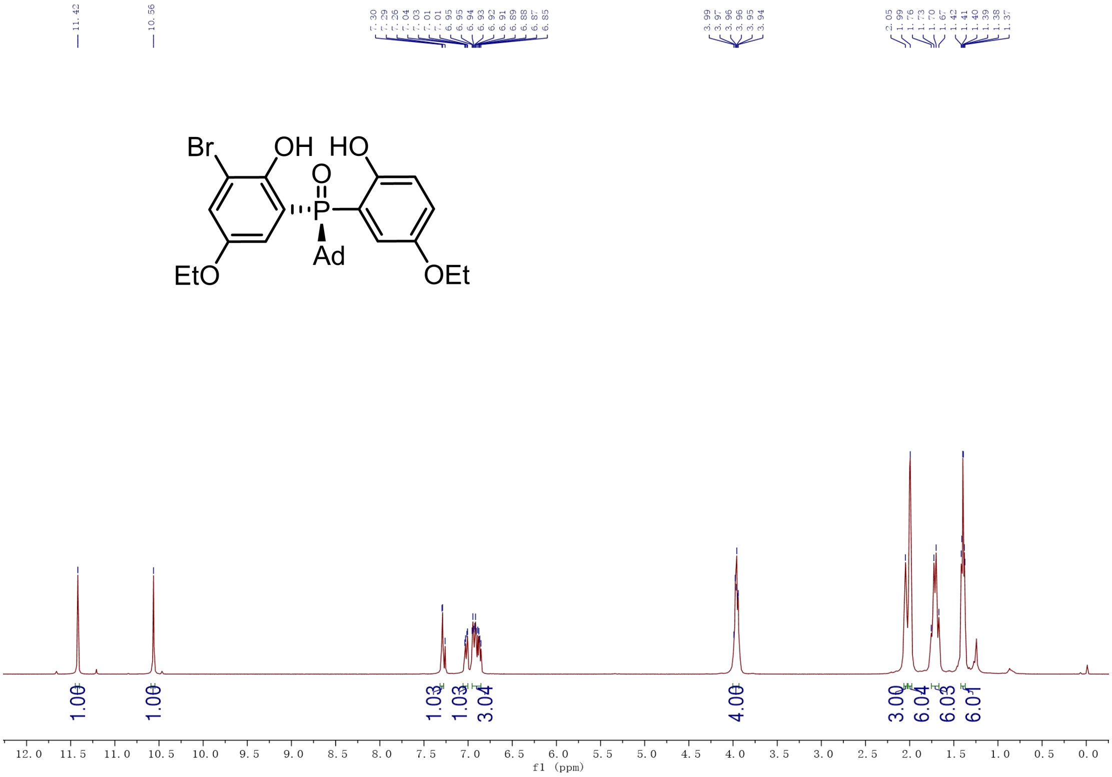

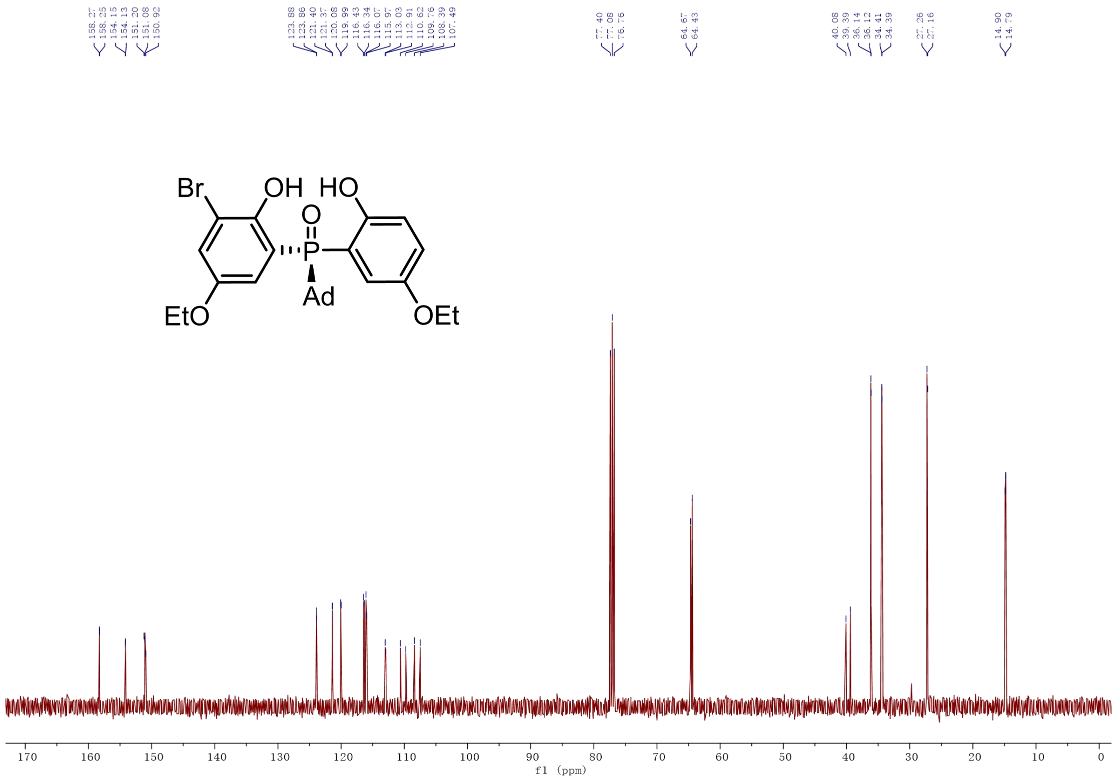
158.27
158.25
154.15
154.13
151.20
151.08
150.92
123.88
123.86
121.40
121.37
120.08
119.99
116.43
116.34
116.07
115.97
113.03
112.91
110.62
109.76
108.39
107.49
77.40
77.08
76.76
64.67
64.43
40.08
39.39
36.14
36.12
34.41
34.39
27.26
27.16
14.90
14.79
Br
OH
HO
O
P
Ad
EtO
OEt
f1 (ppm)

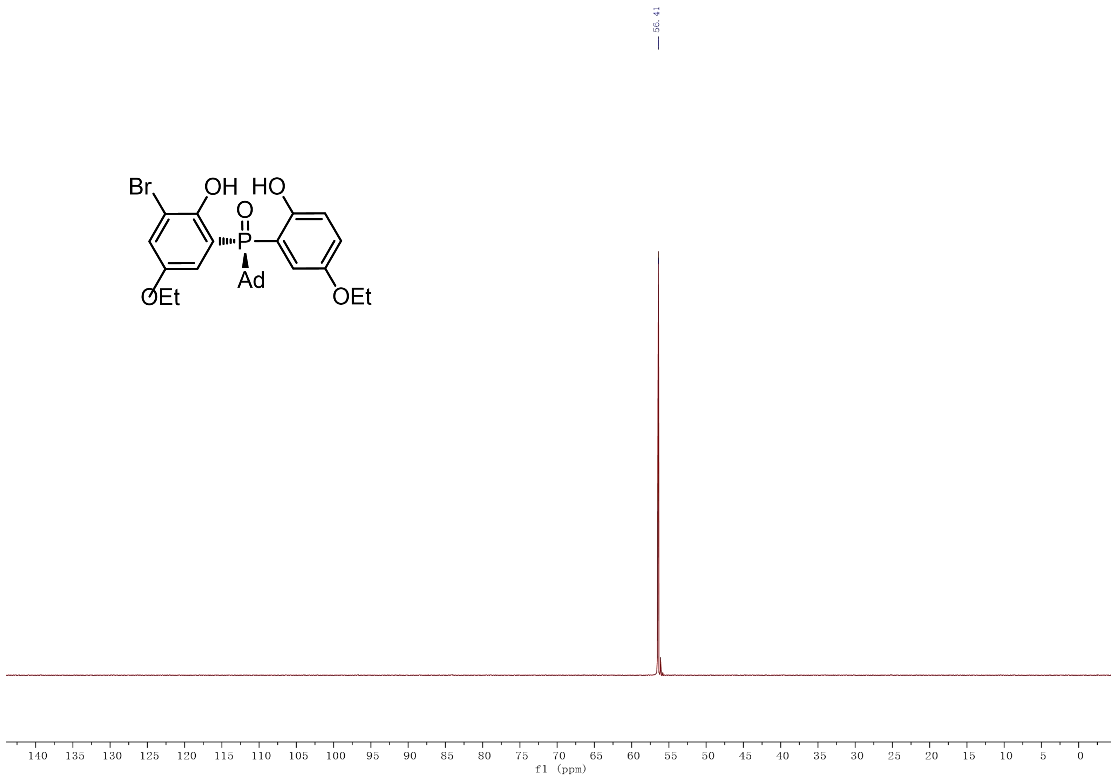
56.41
Br
OH
HO
O
P
Ad
OEt
OEt
f1 (ppm)

^{1}H NMR/^{13}C NMR/^{31}P NMR of product 3y

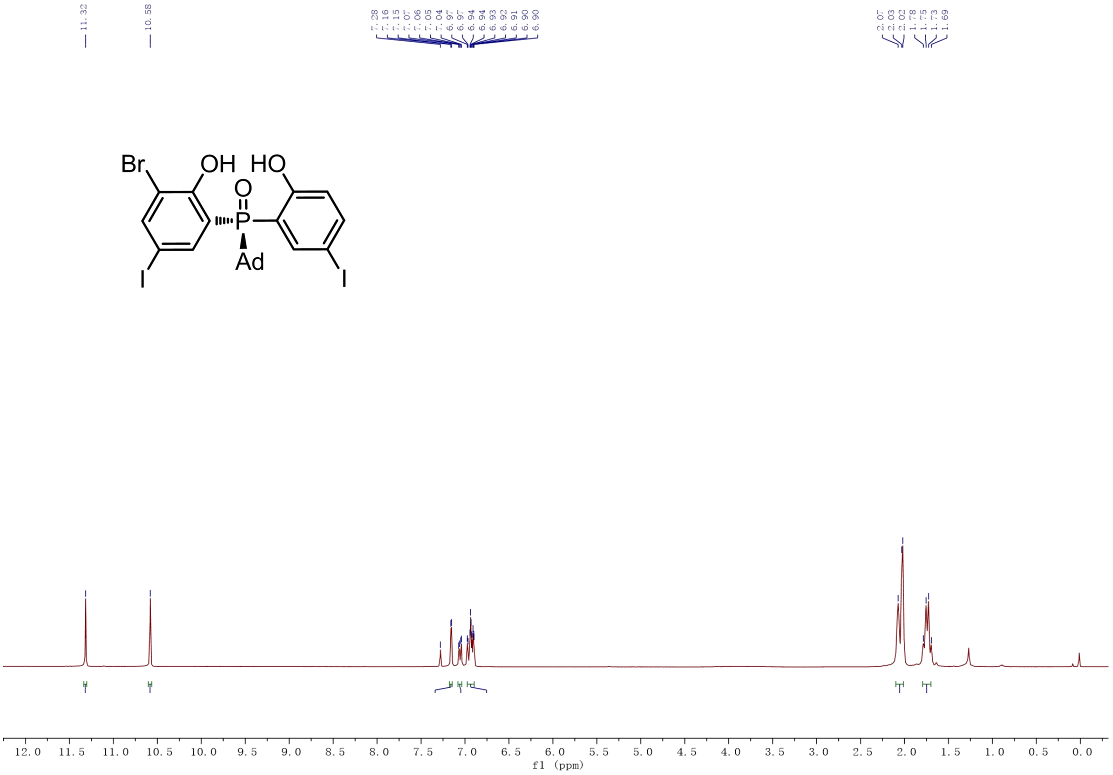

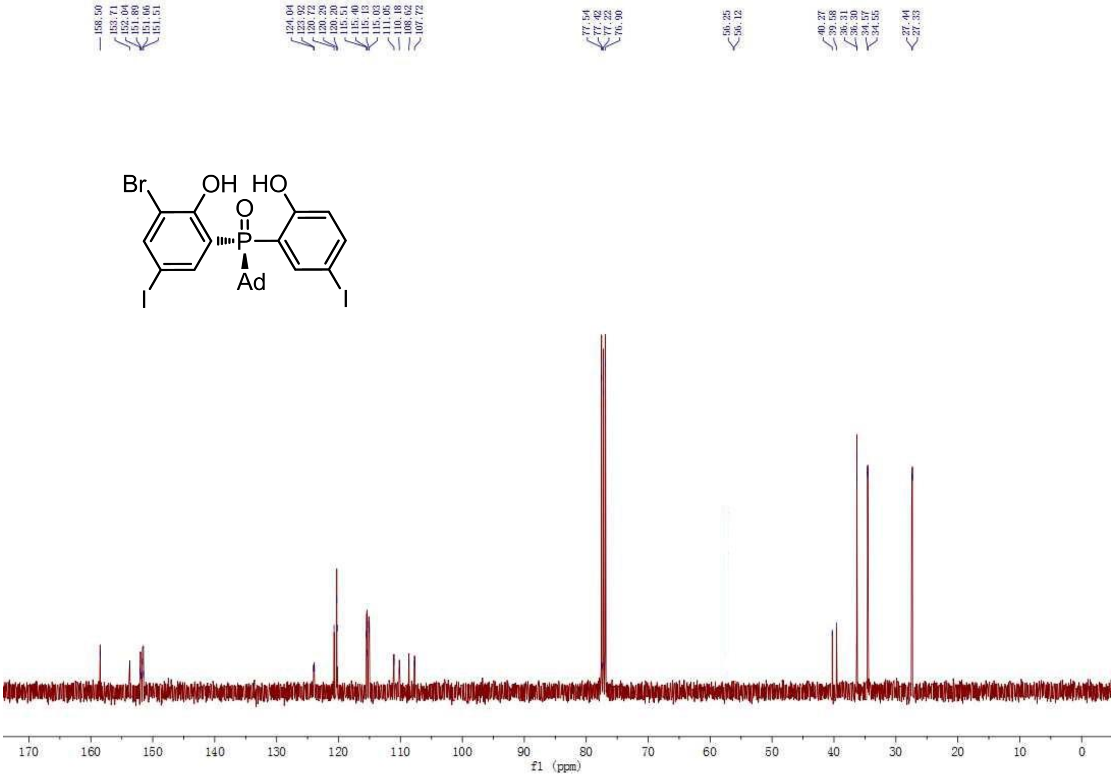

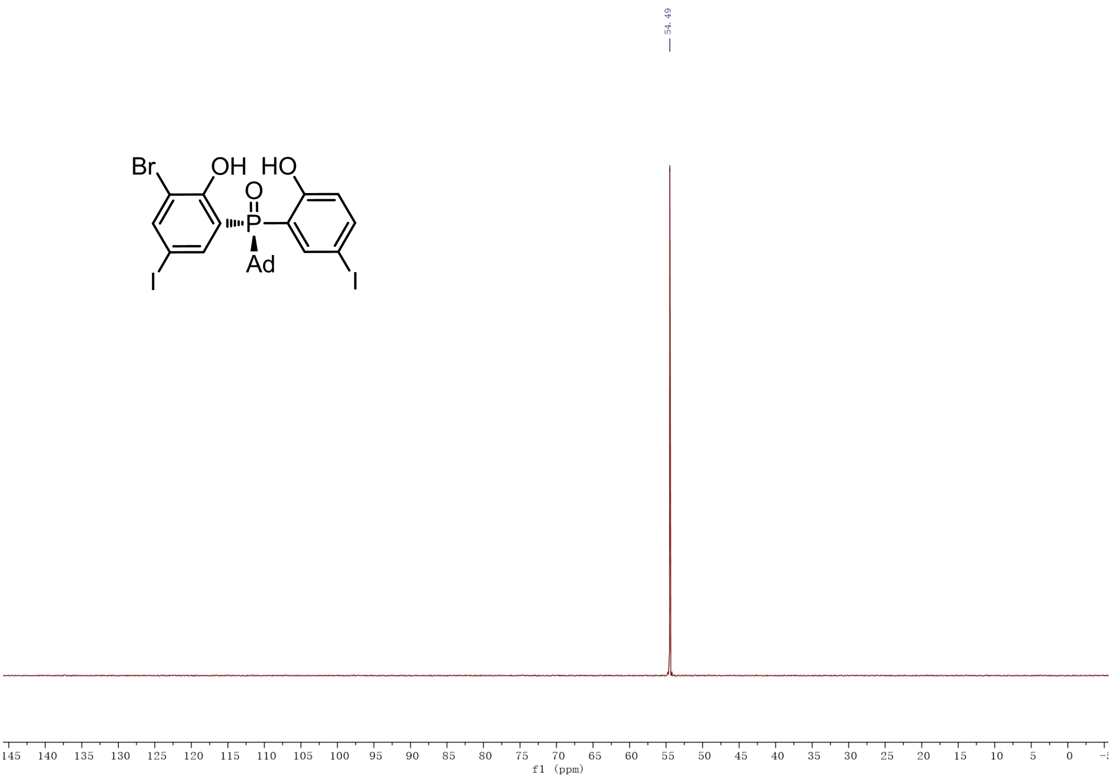

^{1}H NMR/^{13}C NMR/^{31}P NMR of product 3z

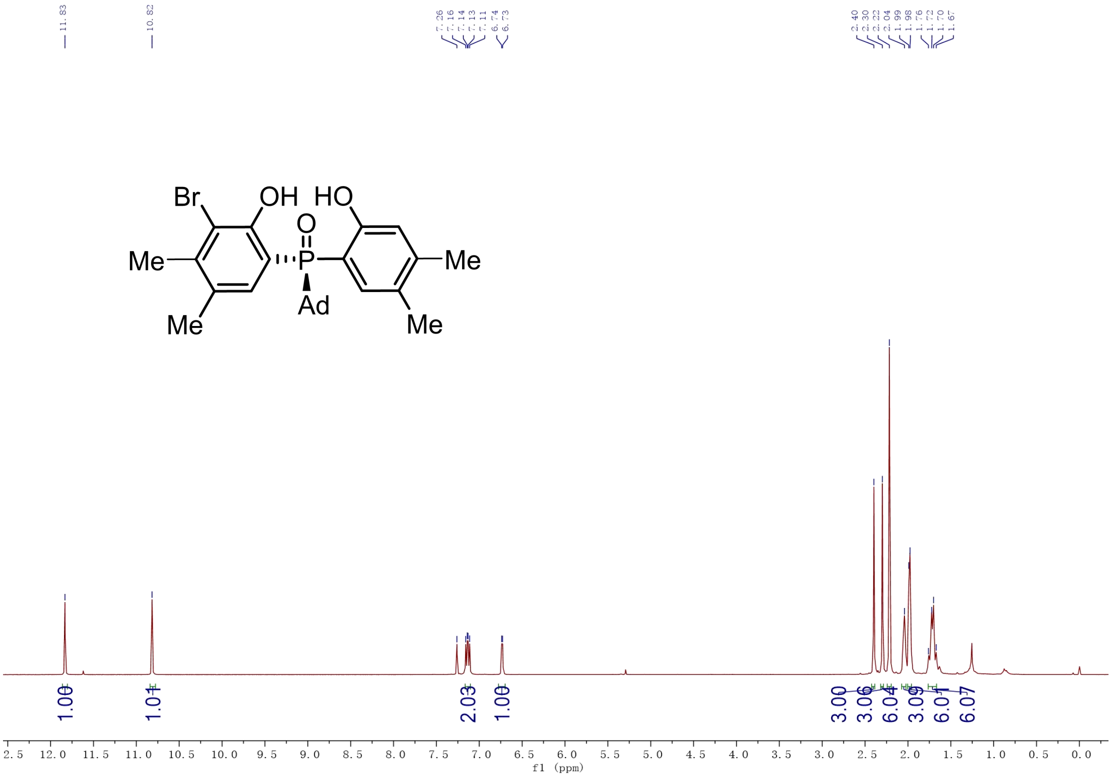

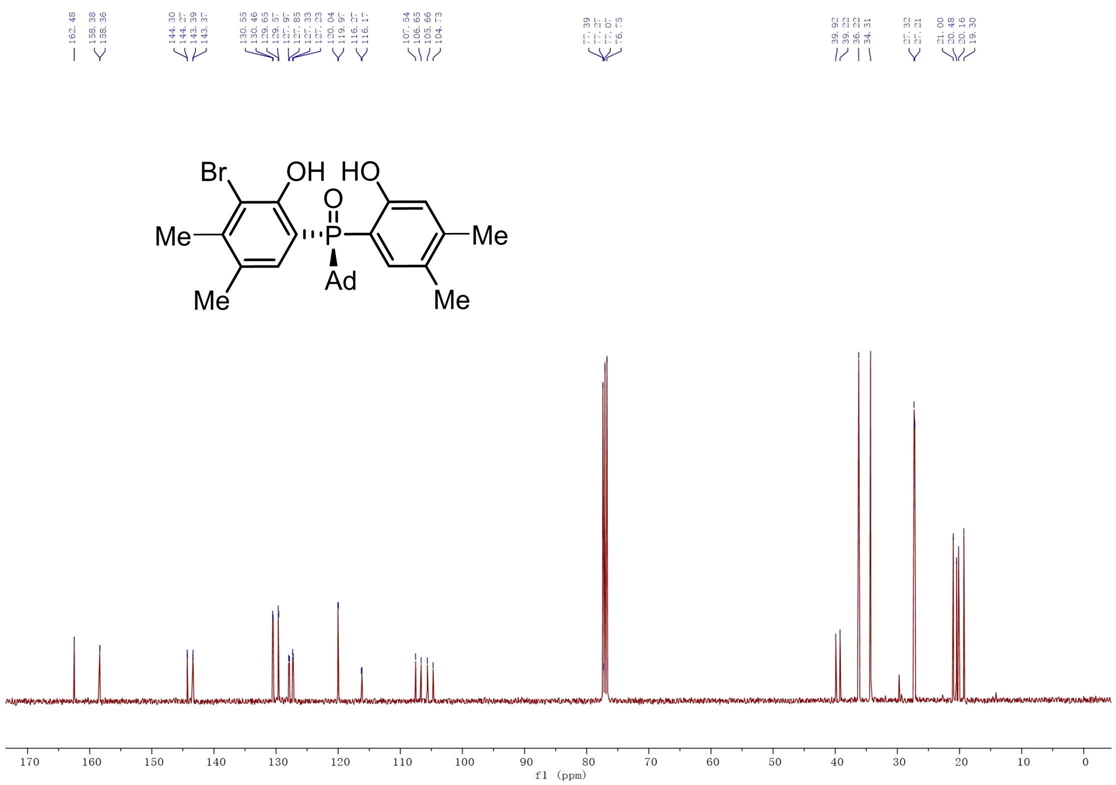
Br
OH
HO
O
P
Me
Me
Ad
Me
Me
f1 (ppm)

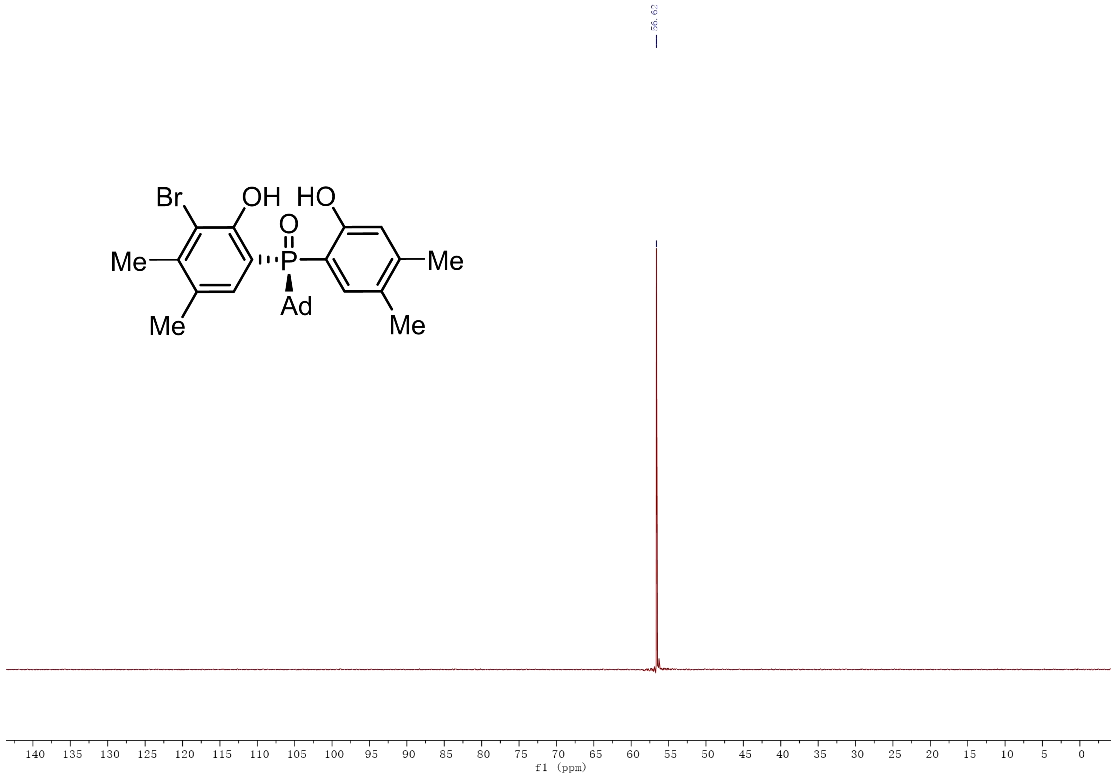
56.62
Br
OH
HO
O
P
Me
Me
Ad
Me
Me
f1 (ppm)

^{1}H NMR/^{13}C NMR/^{31}P NMR of product 5

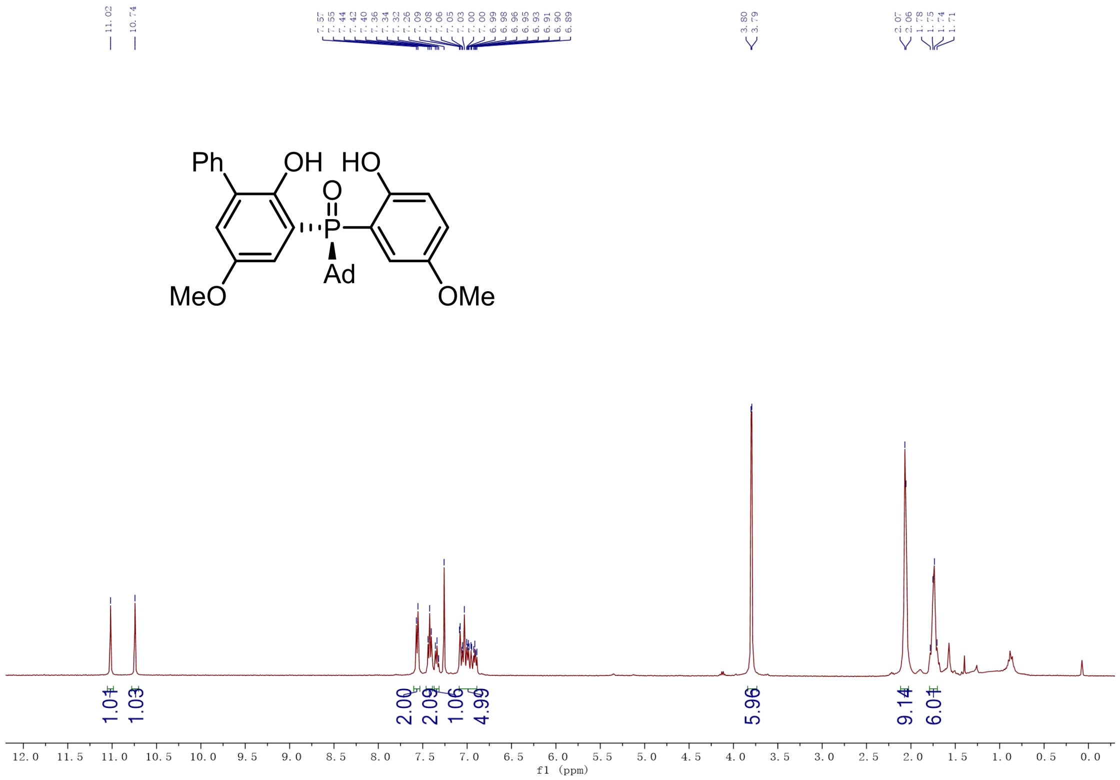

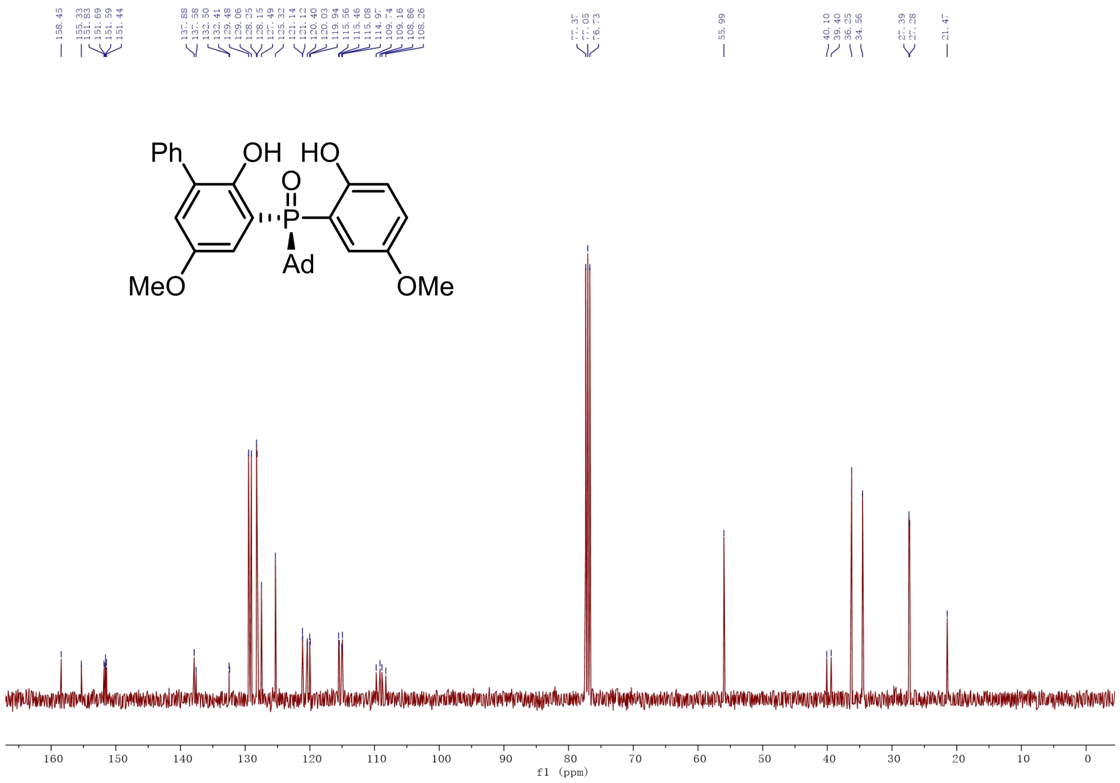

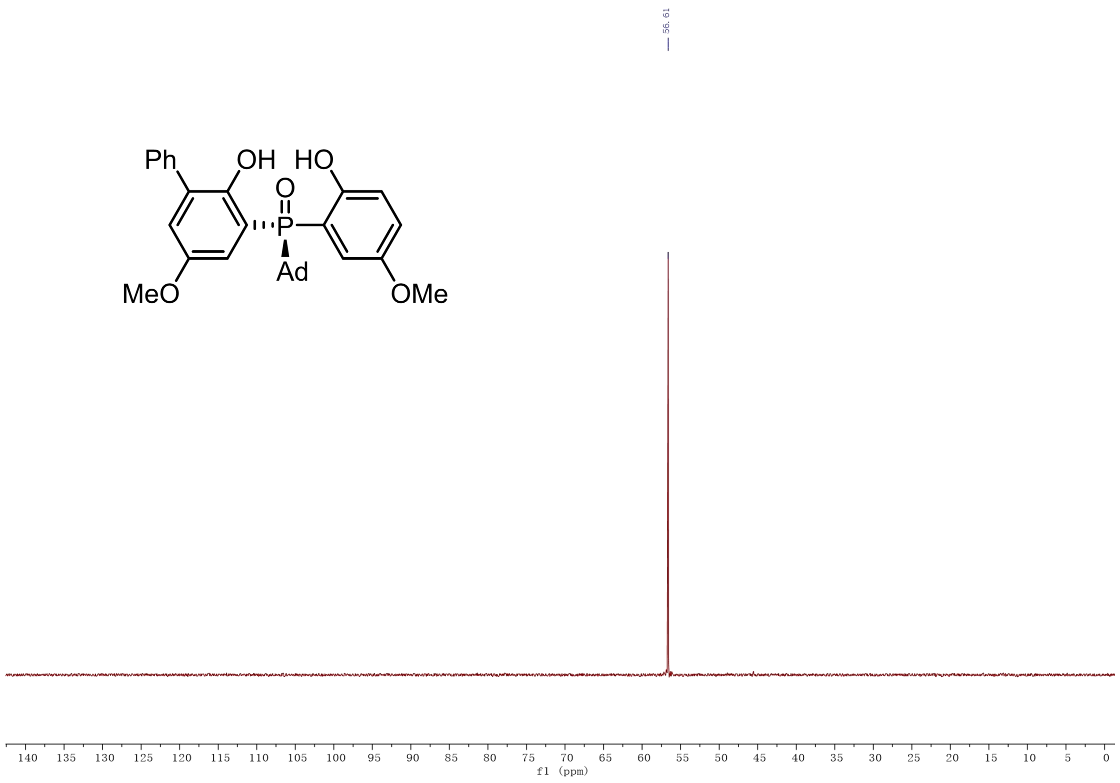

56.61
Ph
OH
HO
O
P
Ad
MeO
OMe
140 135 130 125 120 115 110 105 100 95 90 85 80 75 70 65 60 55 50 45 40 35 30 25 20 15 10 5 0
f1 (ppm)

^{1}H NMR/^{13}C NMR/^{31}P NMR of product 6

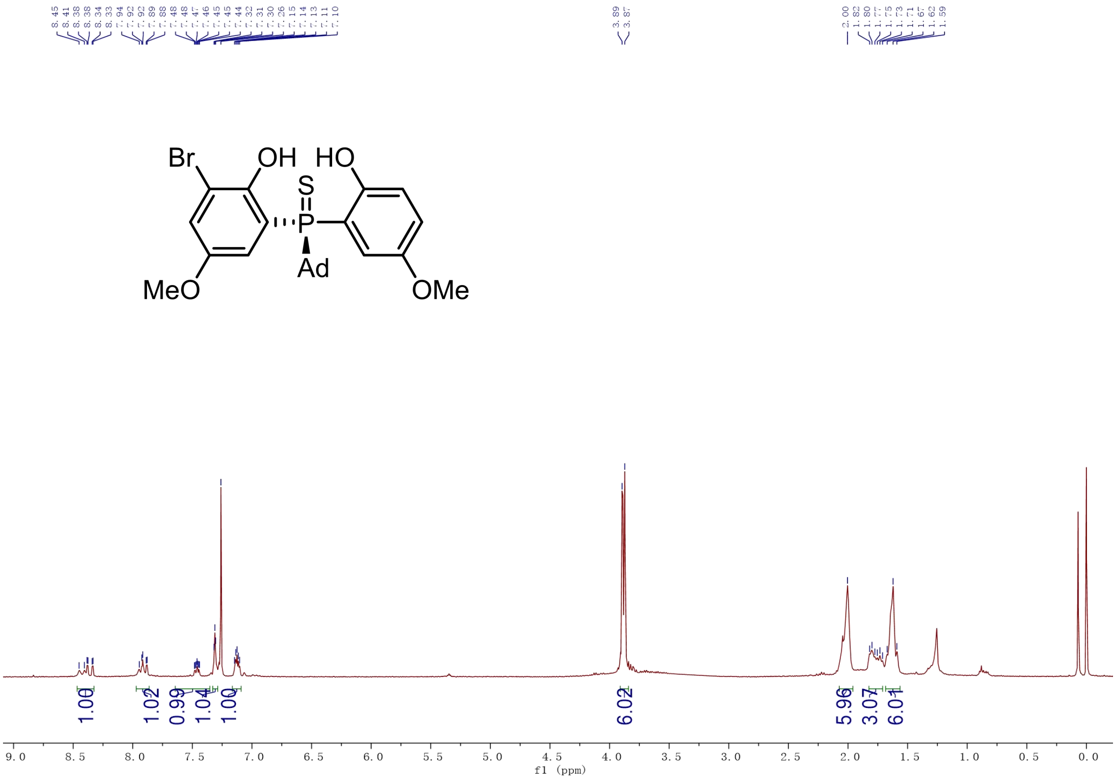

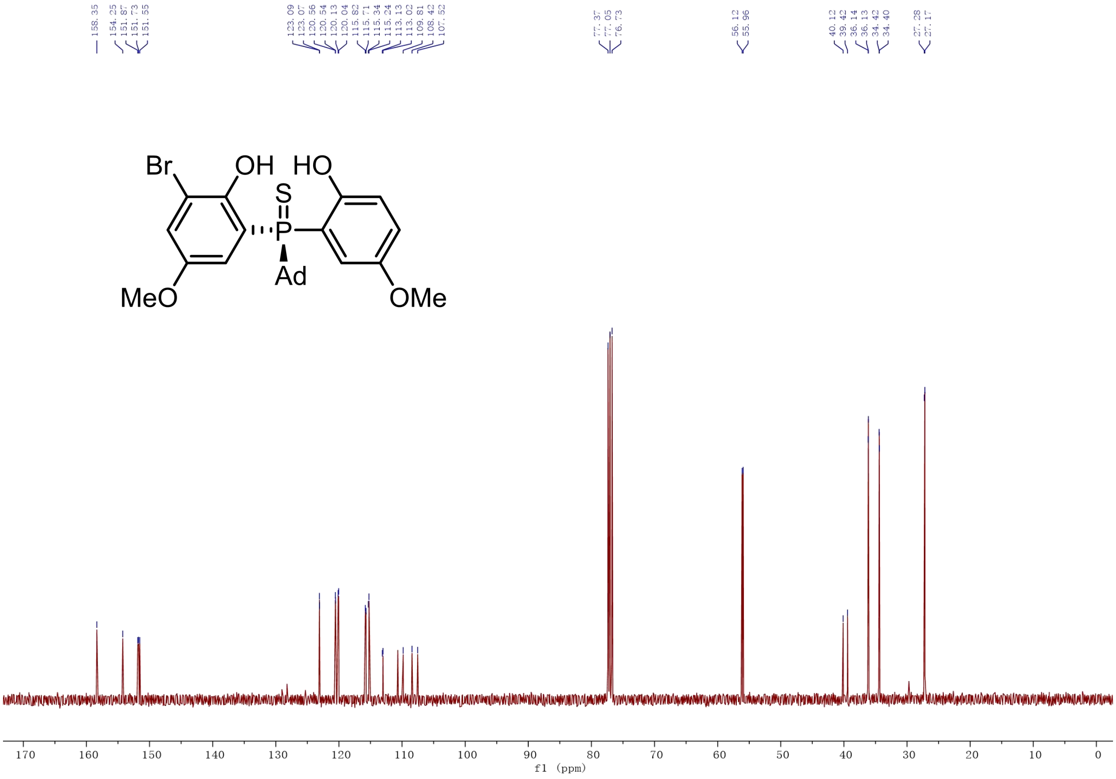

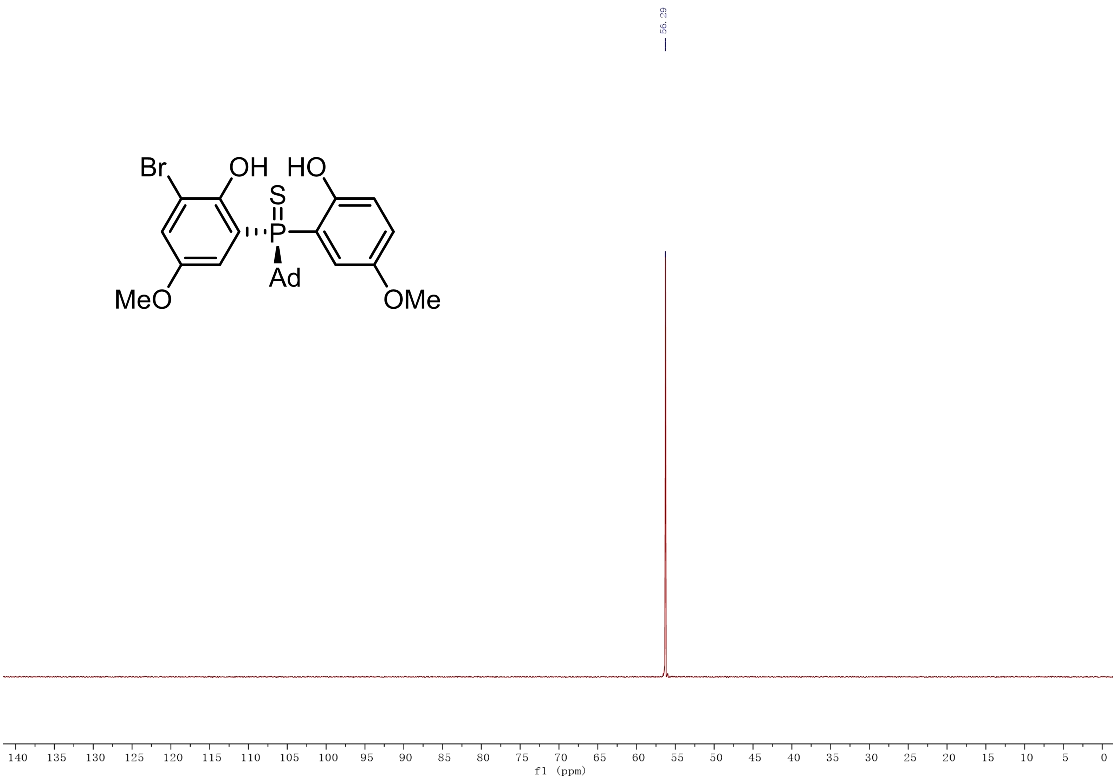
56.29
Br
OH
HO
S
P
Ad
MeO
OMe
140 135 130 125 120 115 110 105 100 95 90 85 80 75 70 65 60 55 50 45 40 35 30 25 20 15 10 5 0
f1 (ppm)

^{1}H NMR/^{13}C NMR/^{31}P NMR of product 7

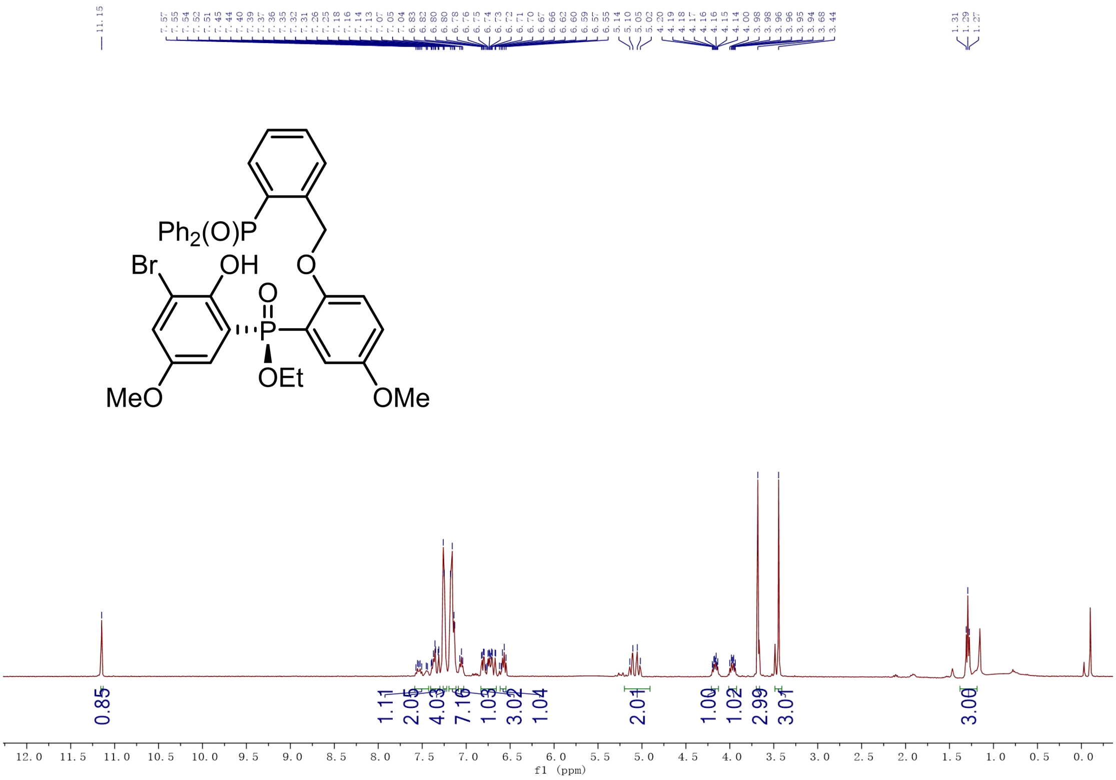

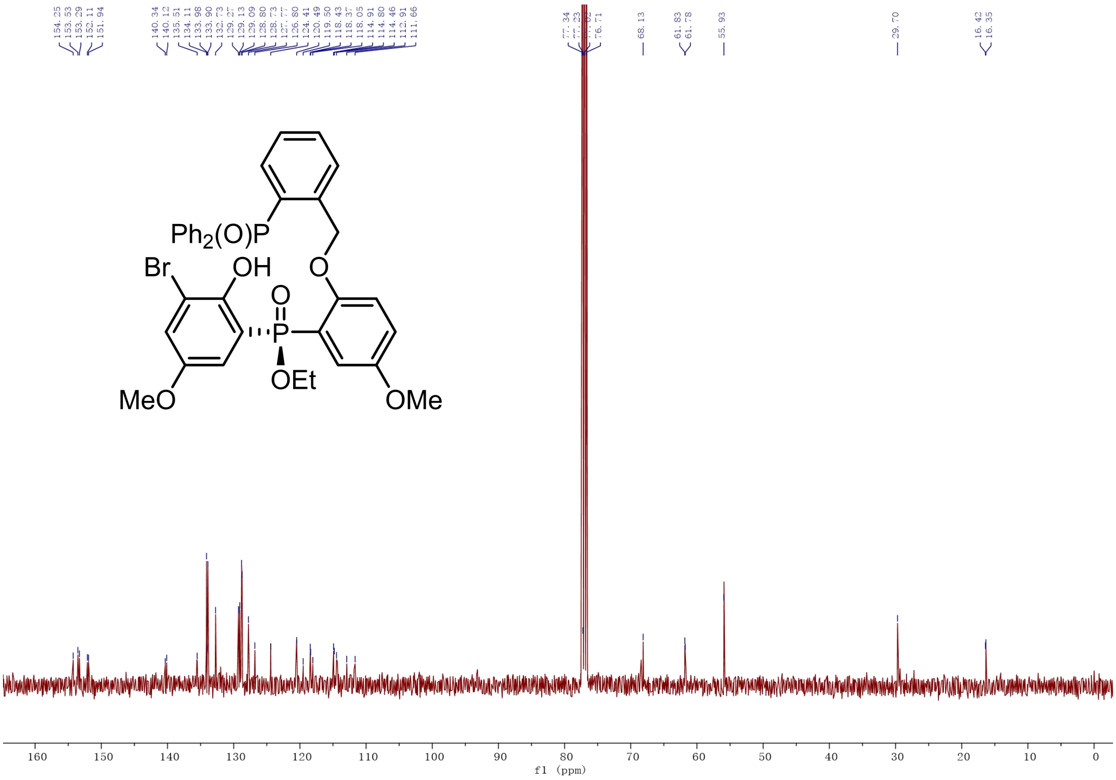

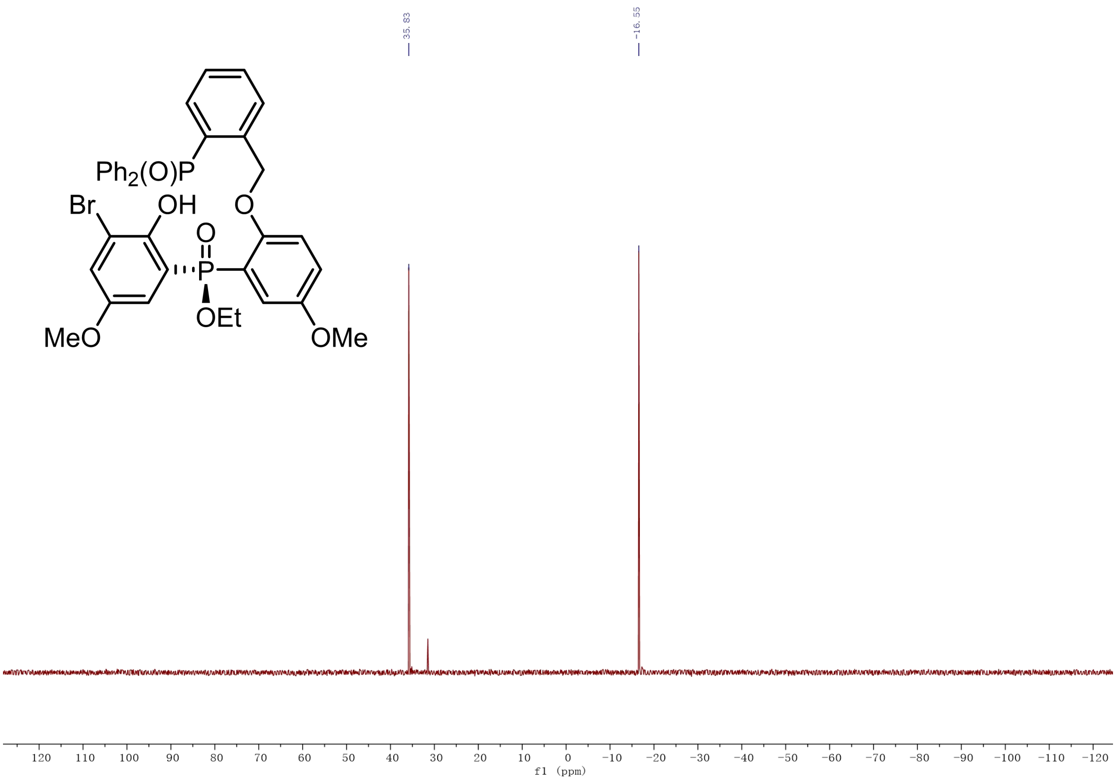

^{1}H NMR/^{13}C NMR/^{31}P NMR of product 9

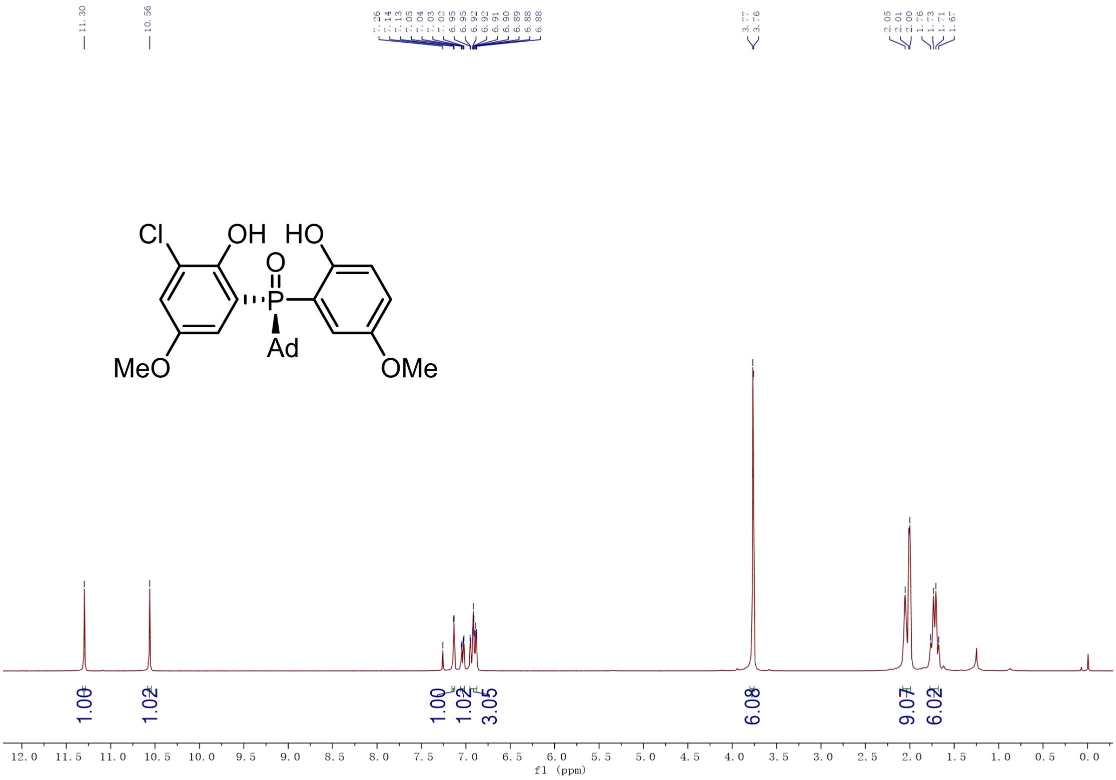

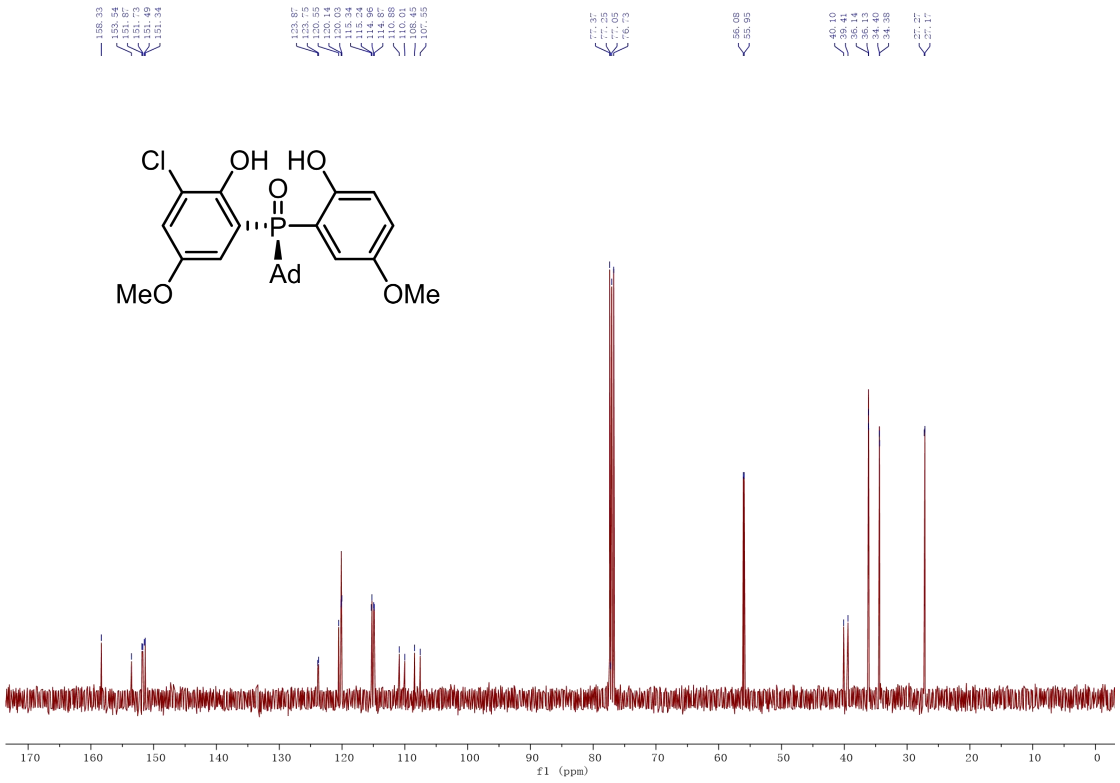
158.33
153.54
151.87
151.73
151.49
151.34
123.87
123.75
120.55
120.14
120.03
115.34
115.24
114.96
114.87
110.88
110.01
108.45
107.55
77.37
77.25
77.05
76.73
56.08
55.95
40.10
39.41
36.14
36.13
34.40
34.38
27.27
27.17
Cl
OH
HO
O
P
Ad
MeO
OMe
f1 (ppm)

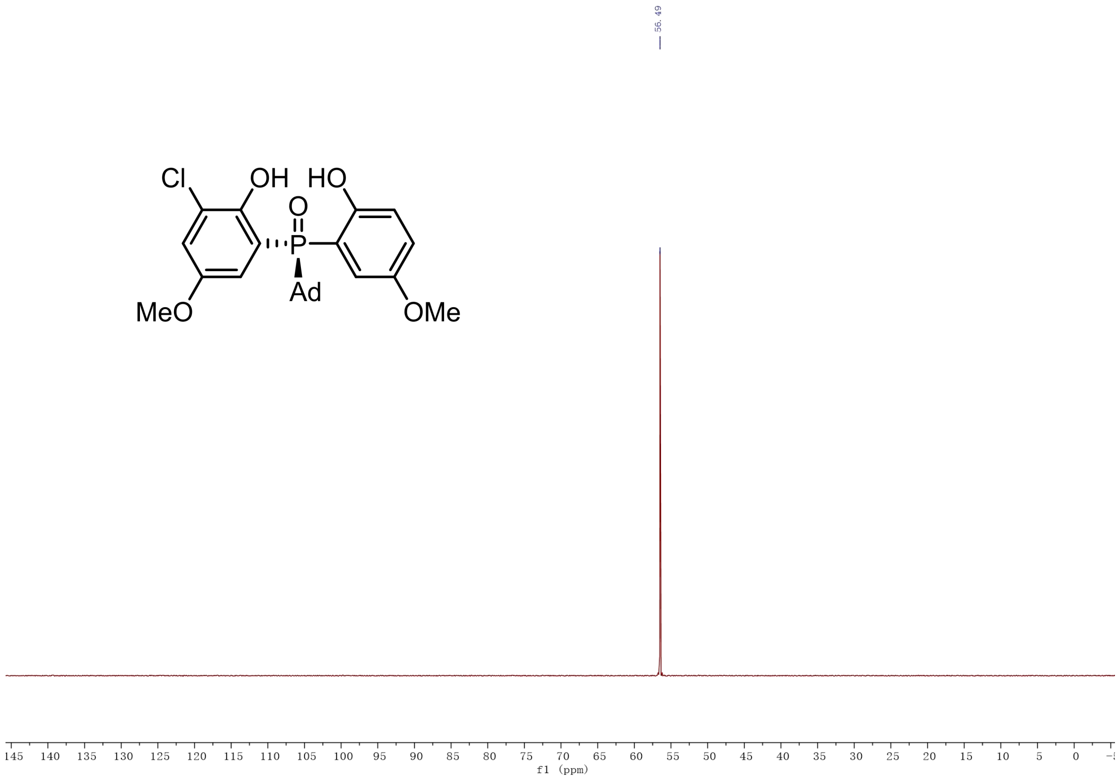
56.49
Cl
OH
HO
O
P
Ad
MeO
OMe
f1 (ppm)

^{1}H NMR/^{13}C NMR/^{31}P NMR of product 10

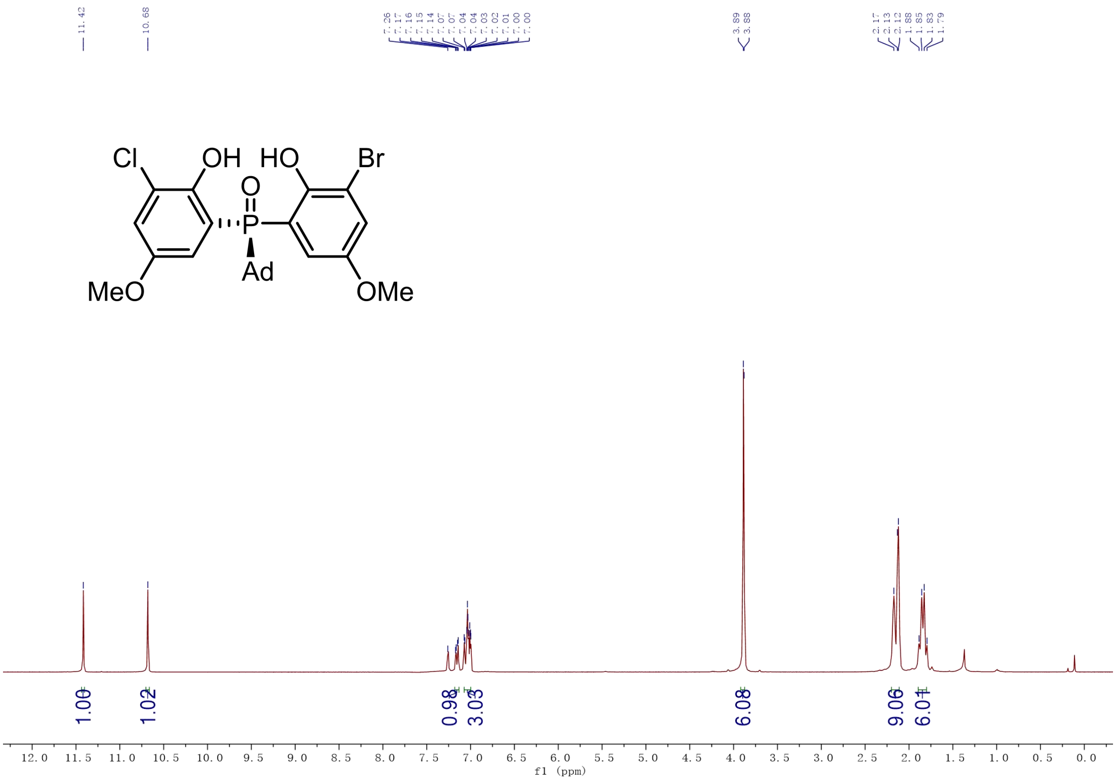

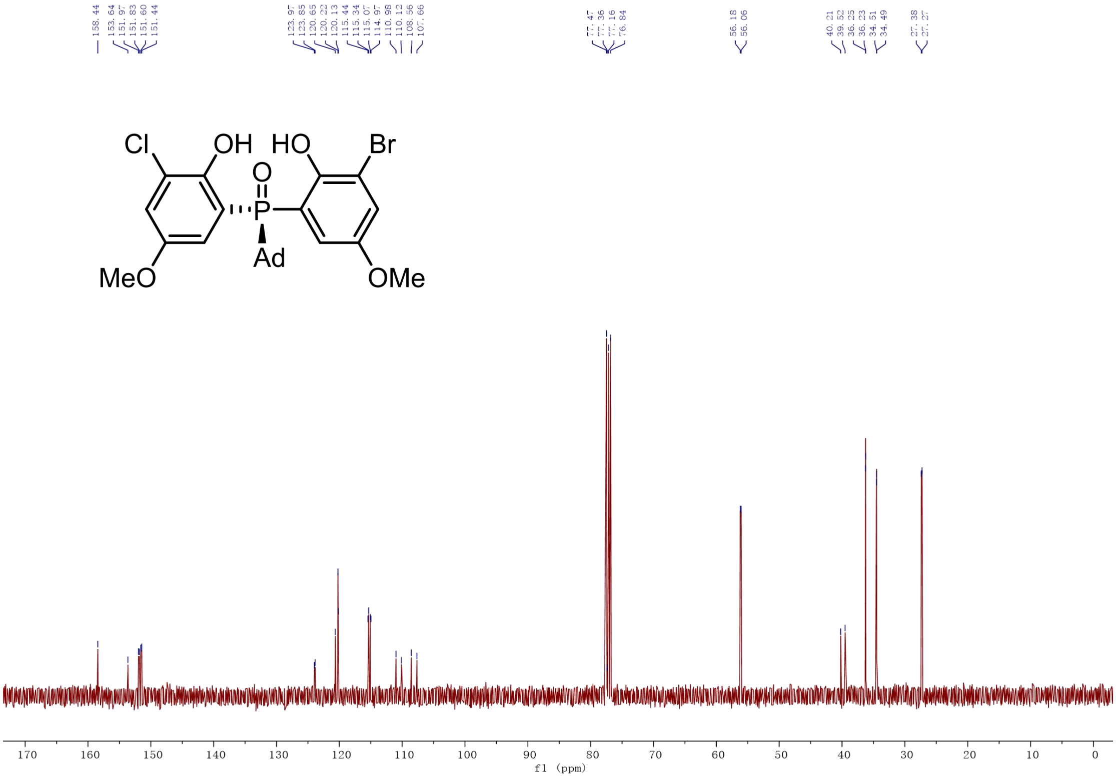

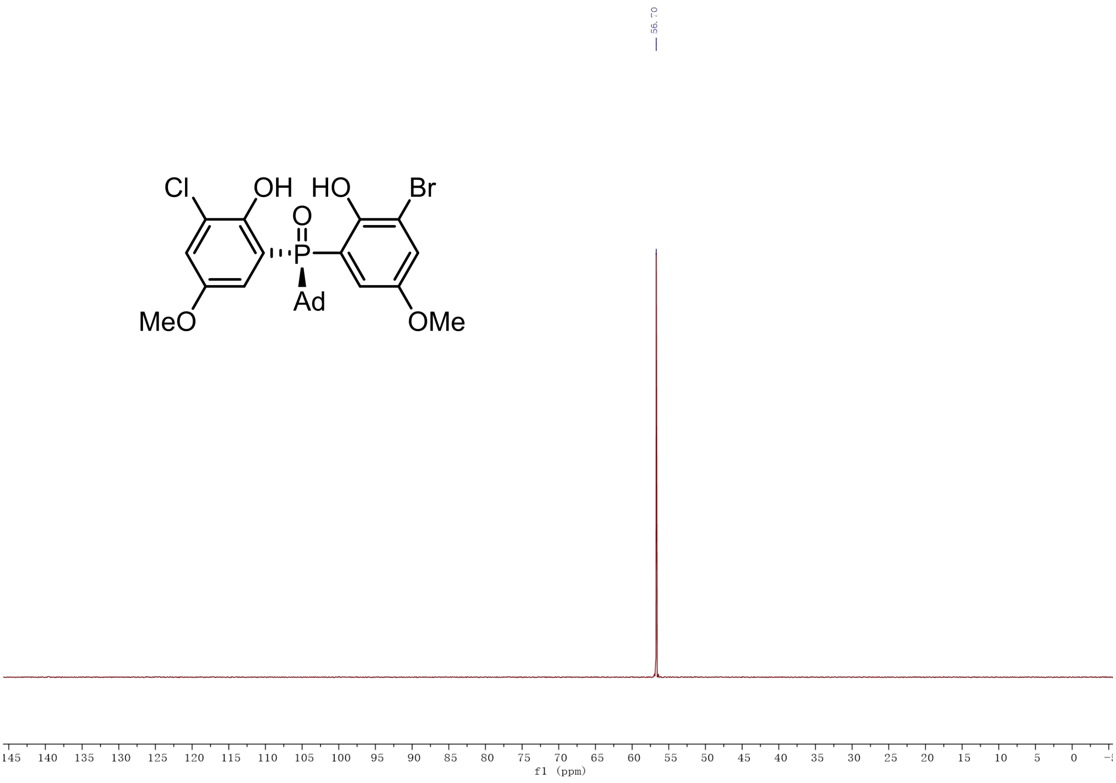

^{1}H NMR/^{13}C NMR/^{31}P NMR of product 11

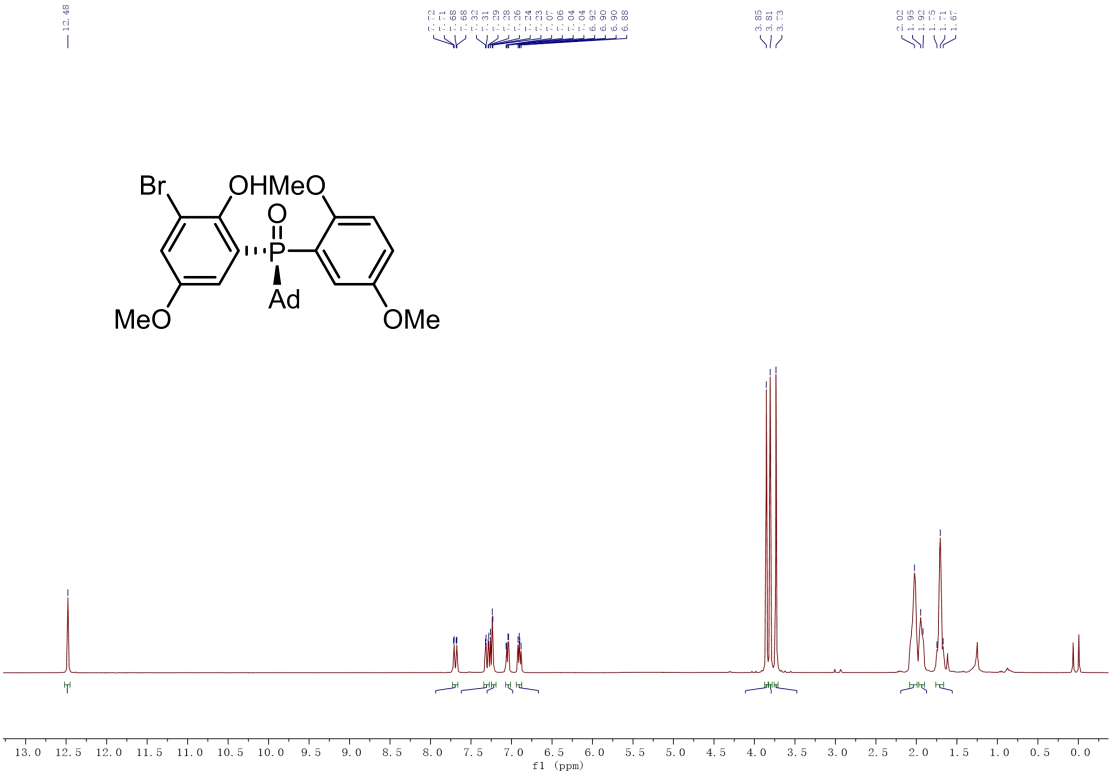

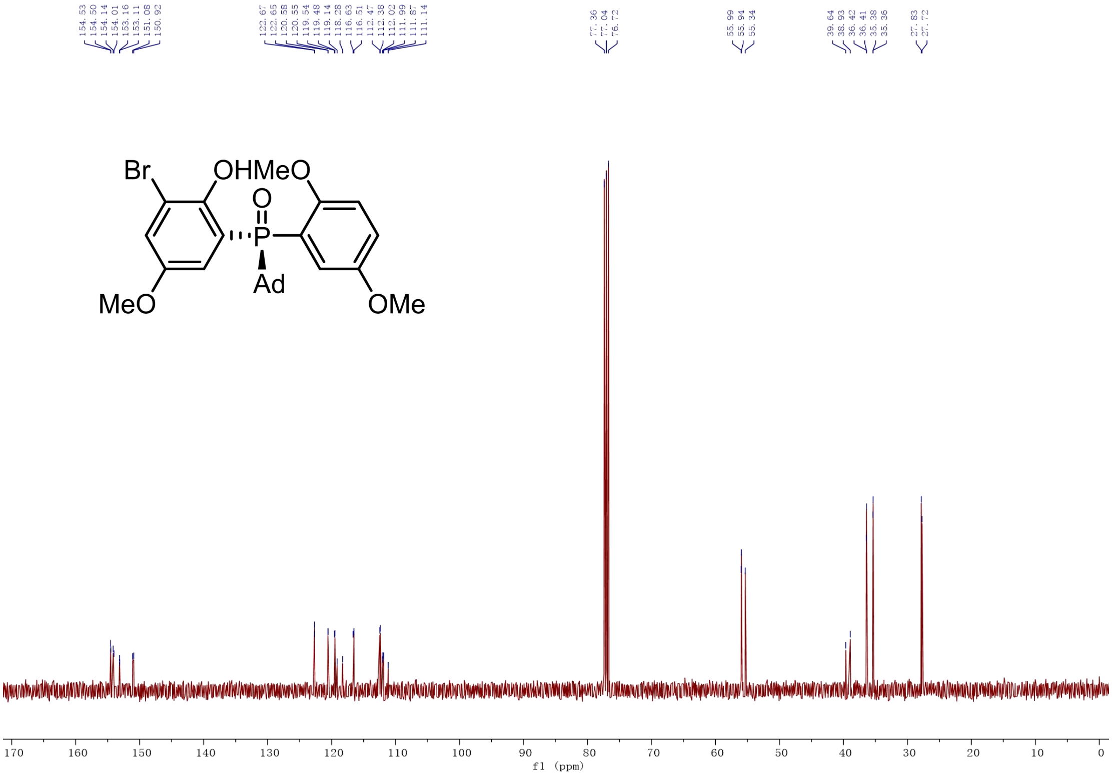
154.53
154.50
154.14
154.01
153.16
153.11
151.08
150.92
122.67
122.65
120.58
120.55
119.54
119.48
119.14
118.28
116.63
116.51
112.47
112.38
112.02
111.99
111.87
111.14
77.36
77.04
76.72
55.99
55.94
55.34
39.64
38.93
36.42
36.41
35.38
35.36
27.83
27.72
Br
OH
MeO
O
P
Ad
MeO
OMe
f1 (ppm)

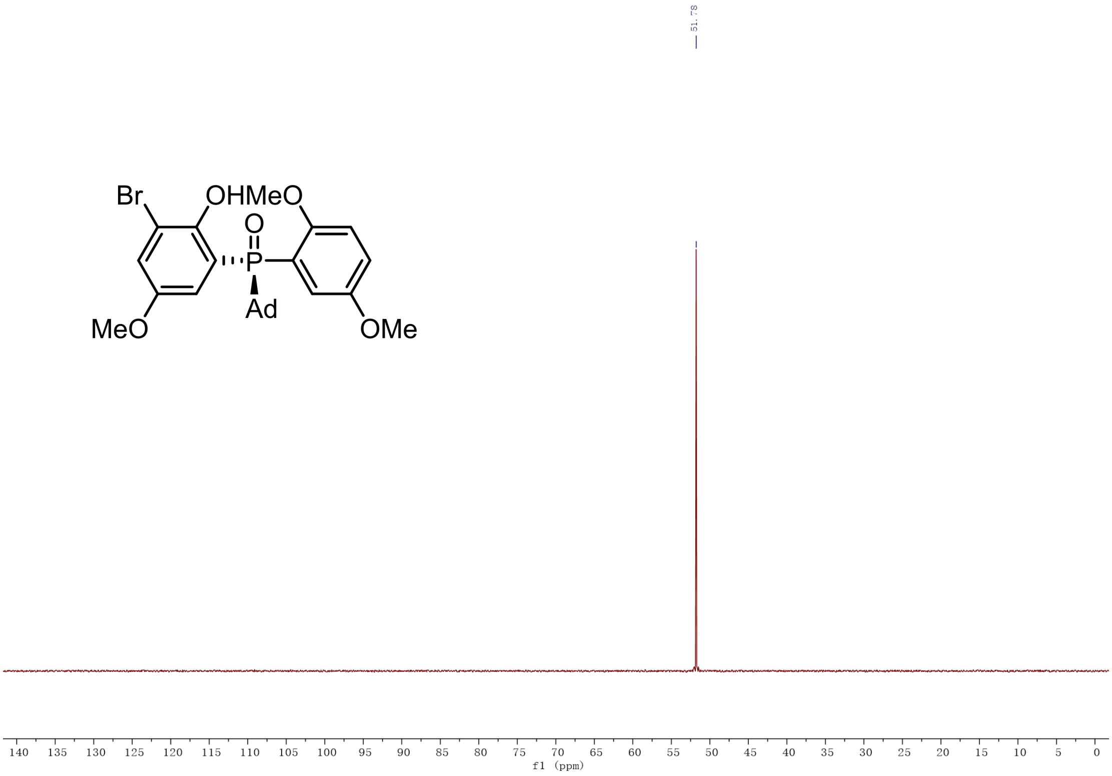
51.78
Br
OH
MeO
O
P
Ad
MeO
OMe
f1 (ppm)

HPLC spectra

HPLC spectra of product 3a

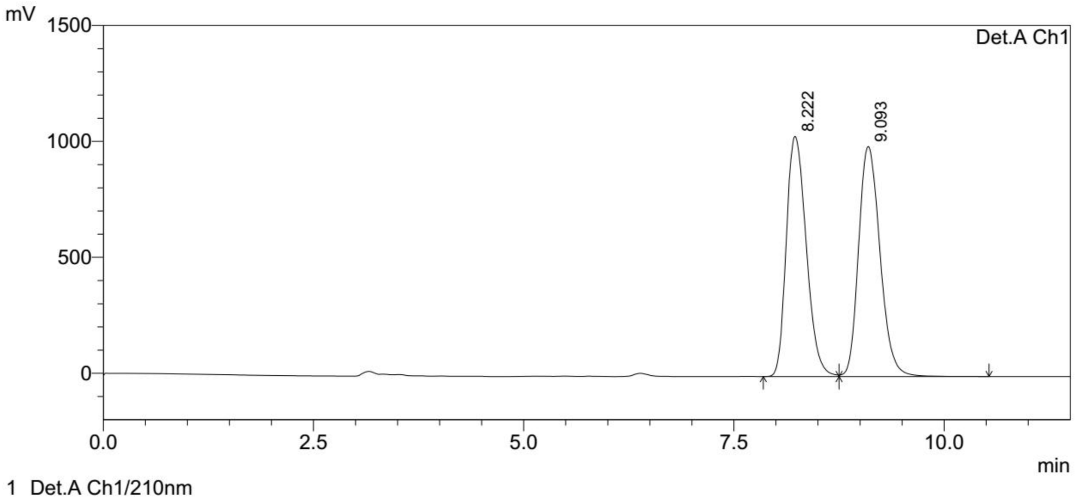

PeakTable

Detector A Ch1 210nm

Peak#	Ret. Time	Area	Height	Area %	Height %
1	8.222	17120824	1035868	49.278	51.077
2	9.093	17622585	992190	50.722	48.923
Total		34743410	2028058	100.000	100.000

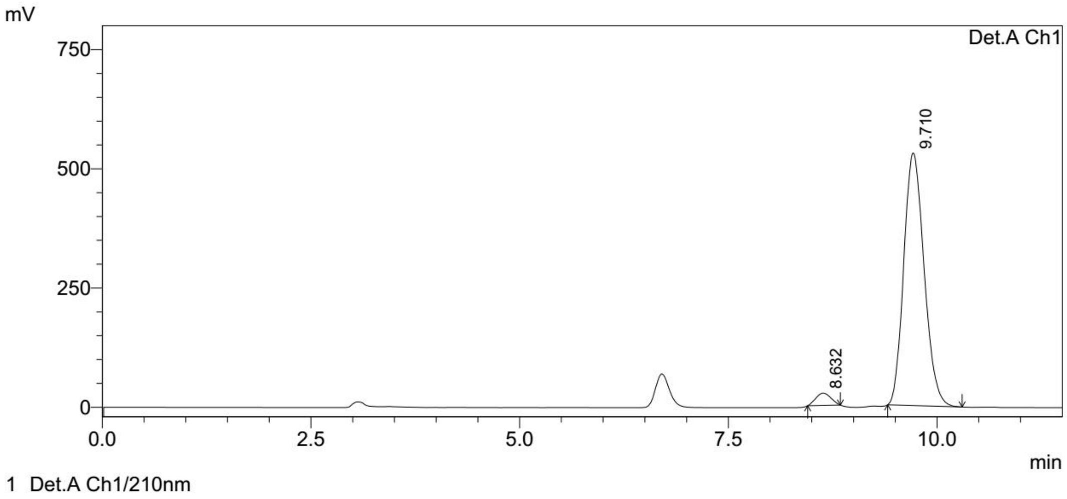

PeakTable

Detector A Ch1 210nm

Peak#	Ret. Time	Area	Height	Area %	Height %
1	8.632	323665	25748	3.476	4.635
2	9.710	8987610	529724	96.524	95.365
Total		9311275	555473	100.000	100.000

HPLC spectra of product 3b

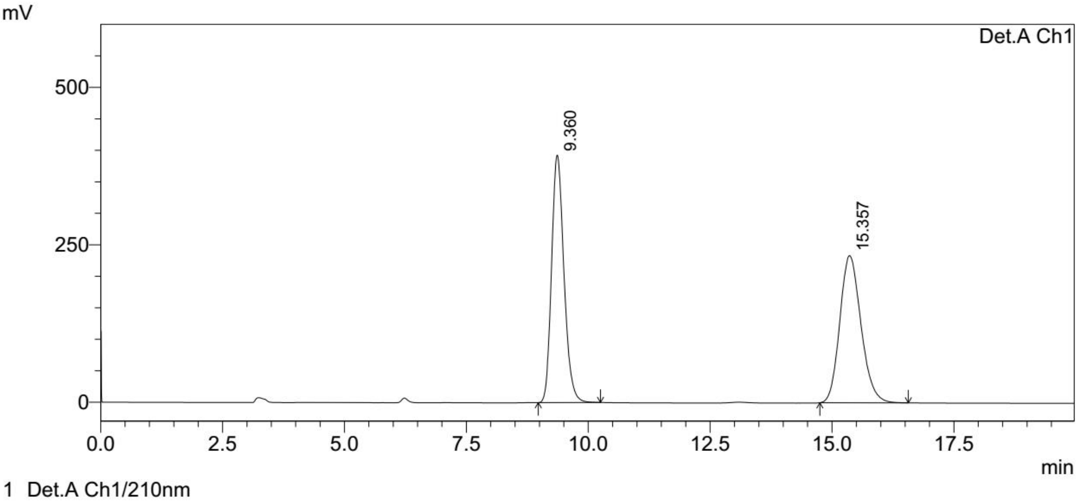

1 Det.A Ch1/210nm

PeakTable

Detector A Ch1 210nm

Peak#	Ret. Time	Area	Height	Area %	Height %
1	9.360	6959307	392844	49.649	62.736
2	15.357	7057702	233347	50.351	37.264
Total		14017009	626191	100.000	100.000

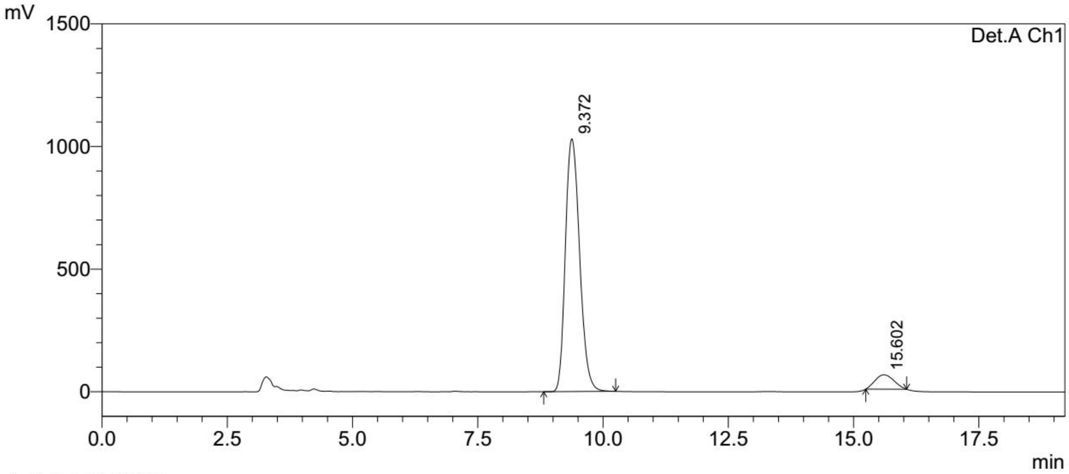

1 Det.A Ch1/210nm

PeakTable

Detector A Ch1 210nm

Peak#	Ret. Time	Area	Height	Area %	Height %
1	9.372	20353362	1030821	92.994	94.592
2	15.602	1533455	58934	7.006	5.408
Total		21886817	1089755	100.000	100.000

HPLC spectra of product 3c

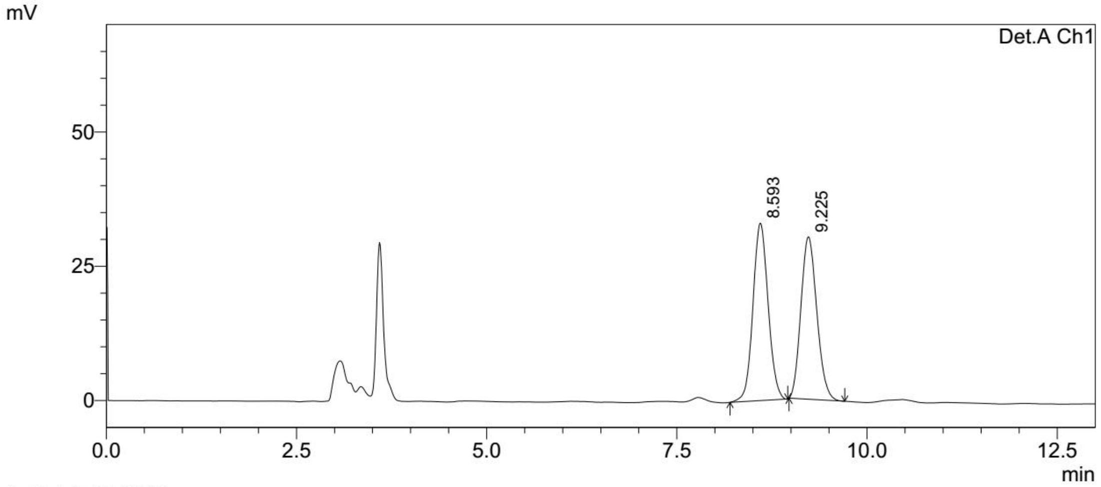

1 Det.A Ch1/210nm

PeakTable

Detector A Ch1 210nm

Peak#	Ret. Time	Area	Height	Area %	Height %
1	8.593	457561	33028	50.969	52.227
2	9.225	440165	30211	49.031	47.773
Total		897726	63240	100.000	100.000

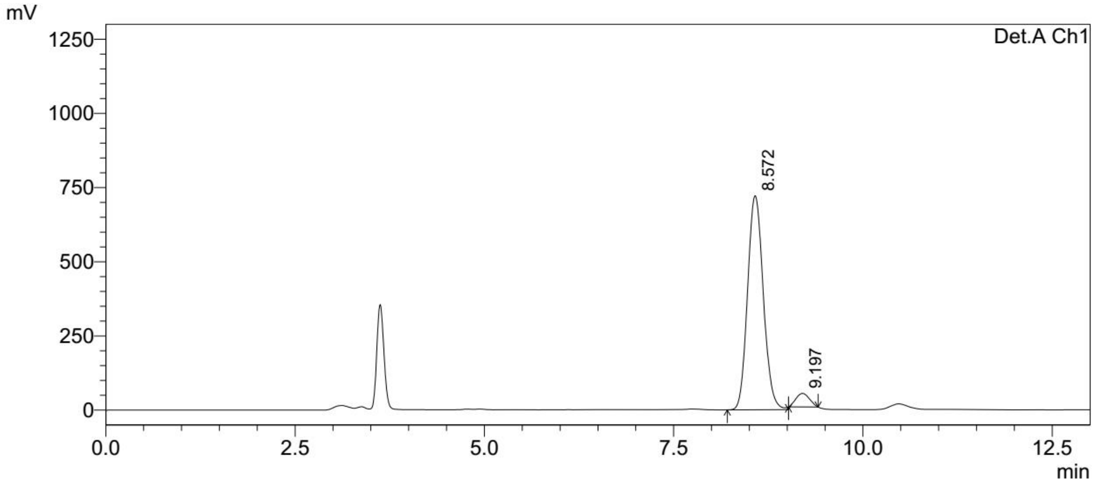

1 Det.A Ch1/210nm

PeakTable

Detector A Ch1 210nm

Peak#	Ret. Time	Area	Height	Area %	Height %
1	8.572	10434955	721972	94.844	94.058
2	9.197	567242	45608	5.156	5.942
Total		11002197	767580	100.000	100.000

HPLC spectra of product 3d

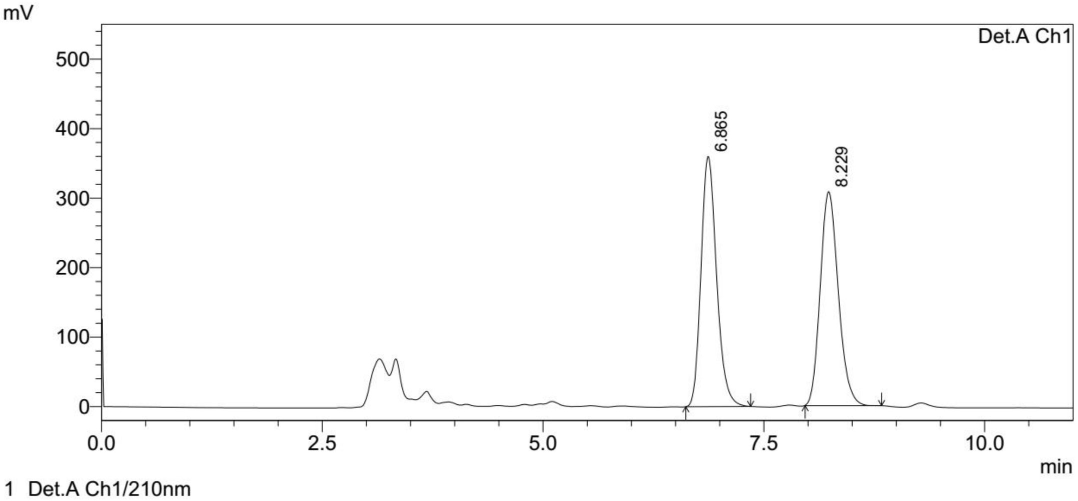

1 Det.A Ch1/210nm

PeakTable

Detector A Ch1 210nm

Peak#	Ret. Time	Area	Height	Area %	Height %
1	6.865	4417586	358402	50.224	53.839
2	8.229	4378243	307289	49.776	46.161
Total		8795830	665691	100.000	100.000

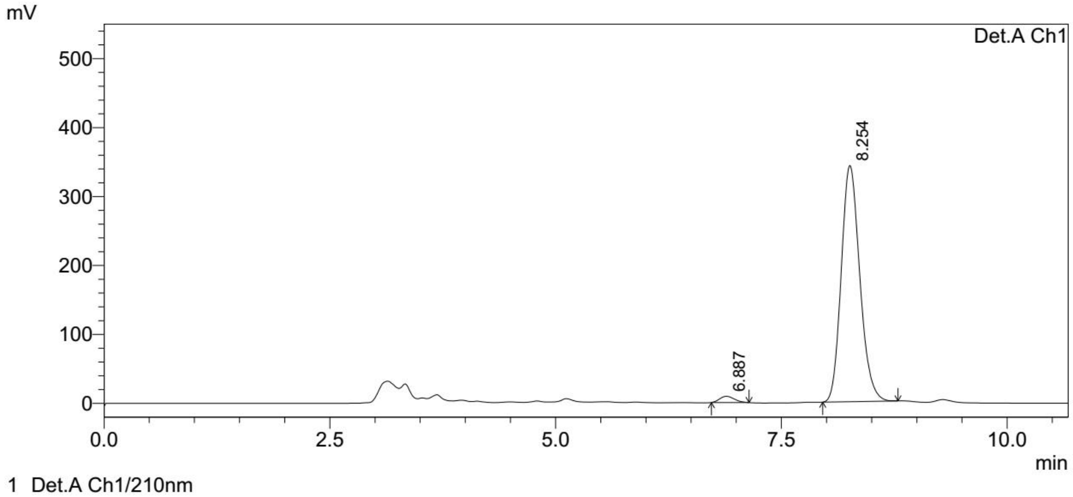

1 Det.A Ch1/210nm

PeakTable

Detector A Ch1 210nm

Peak#	Ret. Time	Area	Height	Area %	Height %
1	6.887	102931	9124	2.062	2.598
2	8.254	4889059	342103	97.938	97.402
Total		4991990	351226	100.000	100.000

HPLC spectra of product 3e

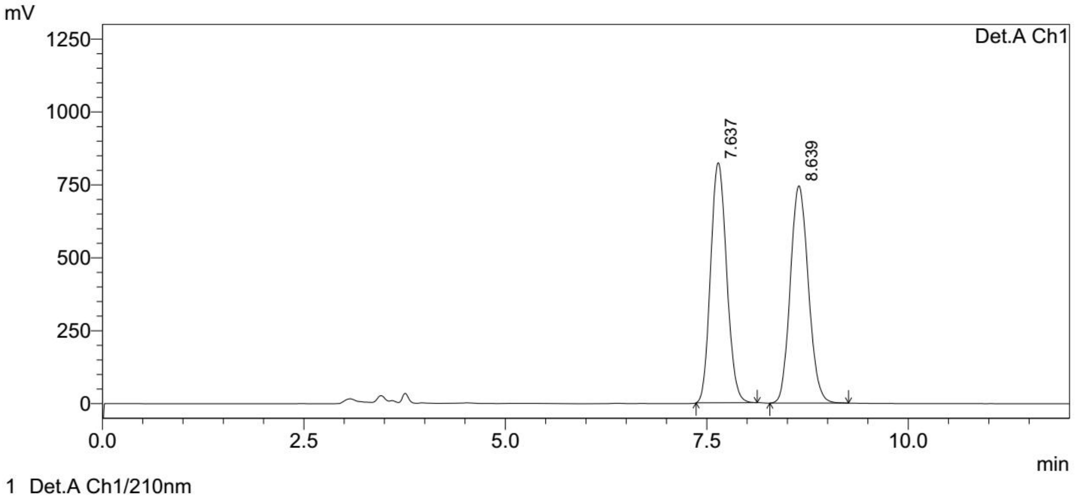

PeakTable

Detector A Ch1 210nm

Peak#	Ret. Time	Area	Height	Area %	Height %
1	7.637	11413356	823249	49.574	52.491
2	8.639	11609421	745106	50.426	47.509
Total		23022777	1568355	100.000	100.000

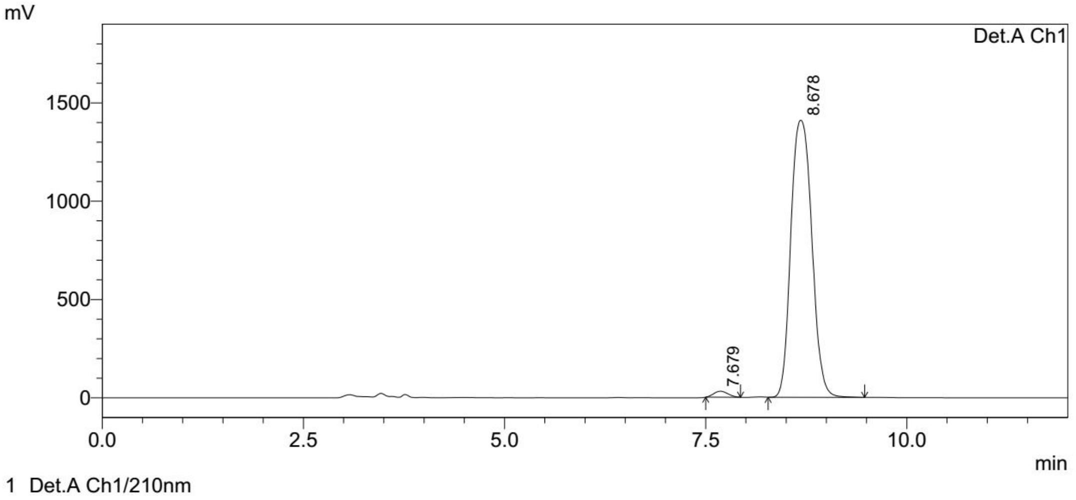

PeakTable

Detector A Ch1 210nm

Peak#	Ret. Time	Area	Height	Area %	Height %
1	7.679	378457	30635	1.463	2.127
2	8.678	25490300	1409390	98.537	97.873
Total		25868757	1440025	100.000	100.000

HPLC spectra of product 3f

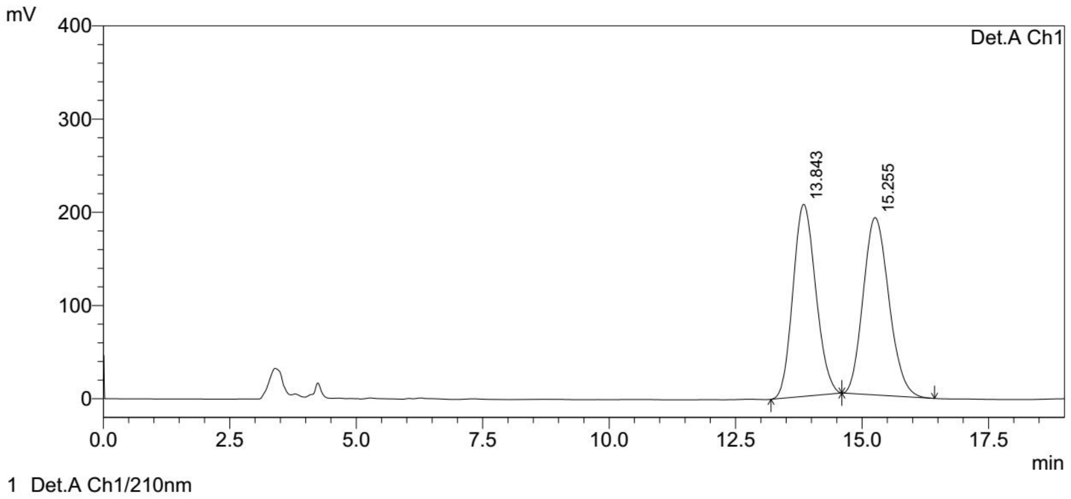

PeakTable

Detector A Ch1 210nm

Peak#	Ret. Time	Area	Height	Area %	Height %
1	13.843	6467121	206083	48.955	51.983
2	15.255	6743133	190364	51.045	48.017
Total		13210254	396446	100.000	100.000

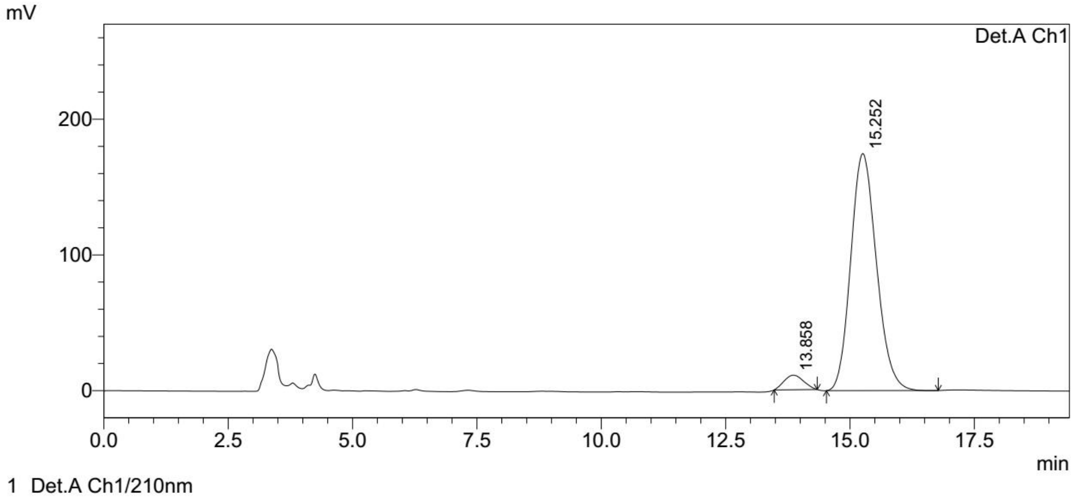

PeakTable

Detector A Ch1 210nm

Peak#	Ret. Time	Area	Height	Area %	Height %
1	13.858	291919	10724	4.441	5.784
2	15.252	6281875	174670	95.559	94.216
Total		6573794	185394	100.000	100.000

HPLC spectra of product 3g

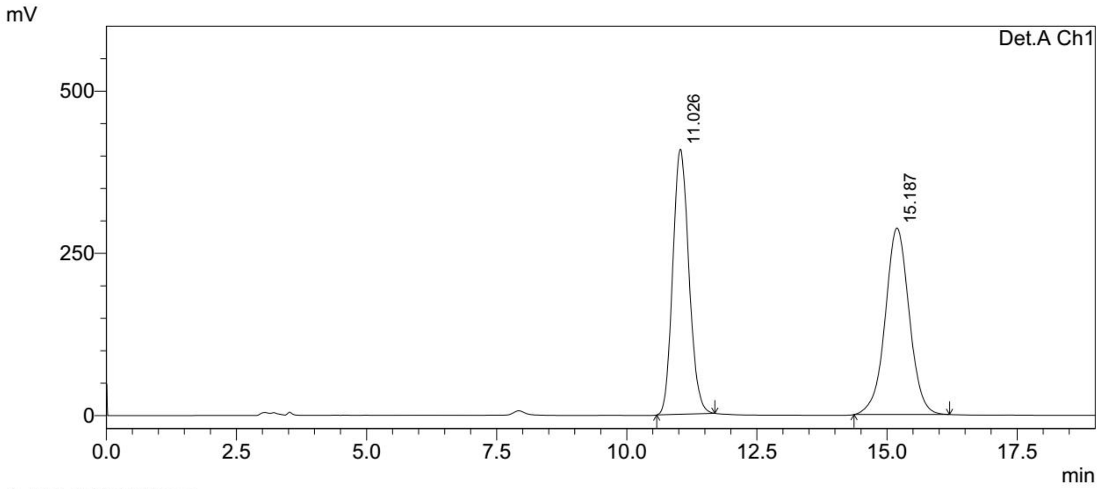

1 Det.A Ch1/210nm

PeakTable

Detector A Ch1 210nm

Peak#	Ret. Time	Area	Height	Area %	Height %
1	11.026	8829620	408686	48.938	58.703
2	15.187	9212859	287509	51.062	41.297
Total		18042479	696195	100.000	100.000

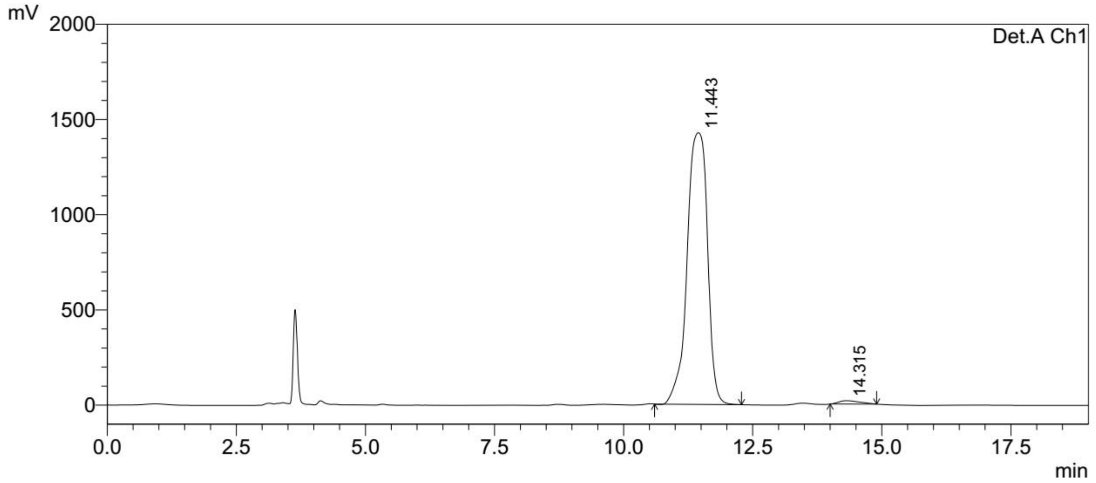

1 Det.A Ch1/210nm

PeakTable

Detector A Ch1 210nm

Peak#	Ret. Time	Area	Height	Area %	Height %
1	11.443	39138303	1427030	98.690	98.744
2	14.315	519404	18155	1.310	1.256
Total		39657707	1445186	100.000	100.000

HPLC spectra of product 3h

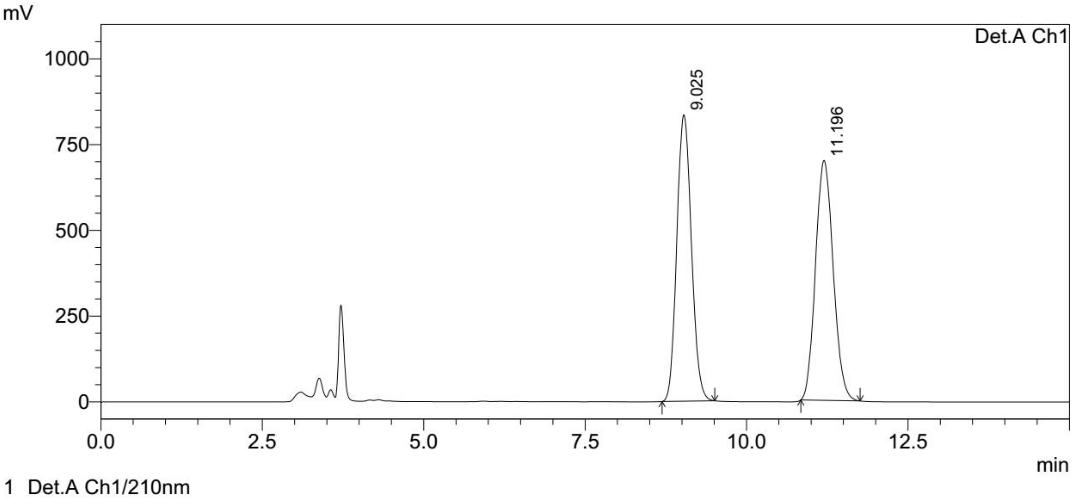

PeakTable

Detector A Ch1 210nm

Peak#	Ret. Time	Area	Height	Area %	Height %
1	9.025	13178642	832506	49.661	54.358
2	11.196	13358785	699011	50.339	45.642
Total		26537428	1531517	100.000	100.000

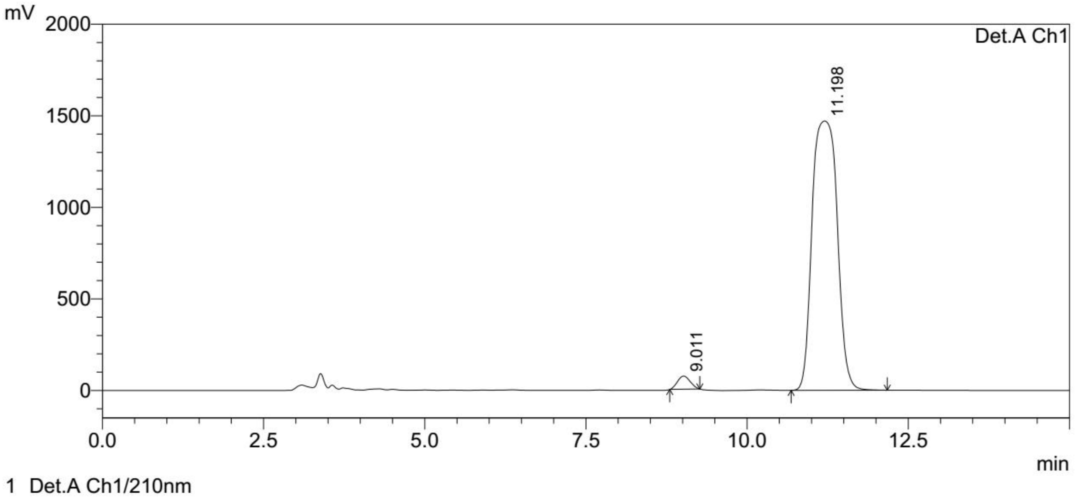

PeakTable

Detector A Ch1 210nm

Peak#	Ret. Time	Area	Height	Area %	Height %
1	9.011	990697	72385	2.424	4.692
2	11.198	39887503	1470501	97.576	95.308
Total		40878200	1542886	100.000	100.000

HPLC spectra of product 3i

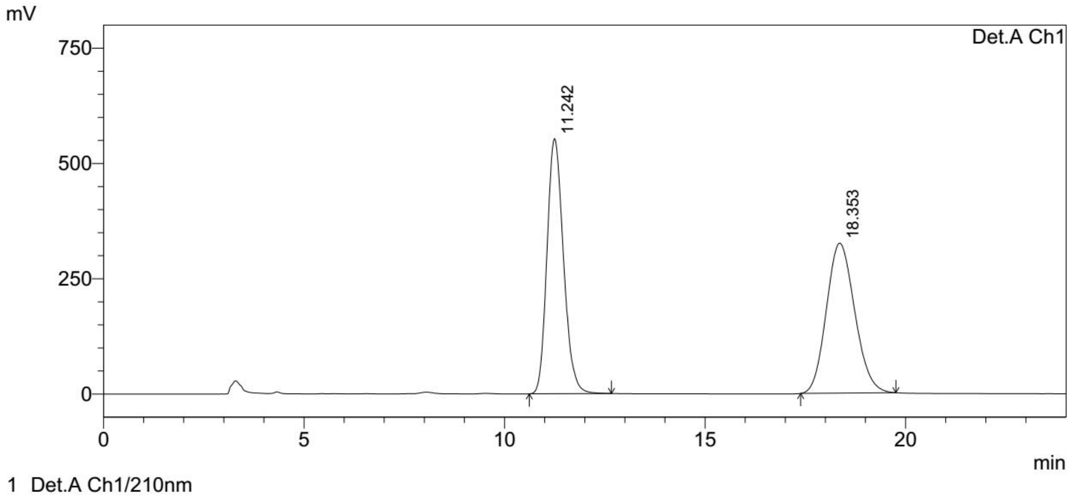

PeakTable

Detector A Ch1 210nm

Peak#	Ret. Time	Area	Height	Area %	Height %
1	11.242	15821170	553178	49.760	62.986
2	18.353	15974022	325083	50.240	37.014
Total		31795193	878261	100.000	100.000

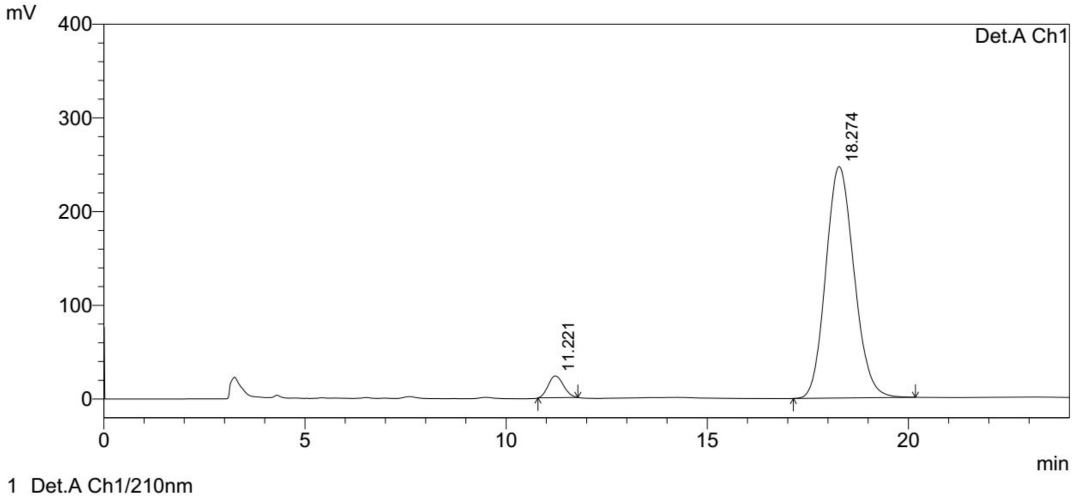

PeakTable

Detector A Ch1 210nm

Peak#	Ret. Time	Area	Height	Area %	Height %
1	11.221	614971	23351	4.841	8.636
2	18.274	12089245	247054	95.159	91.364
Total		12704216	270406	100.000	100.000

HPLC spectra of product 3j

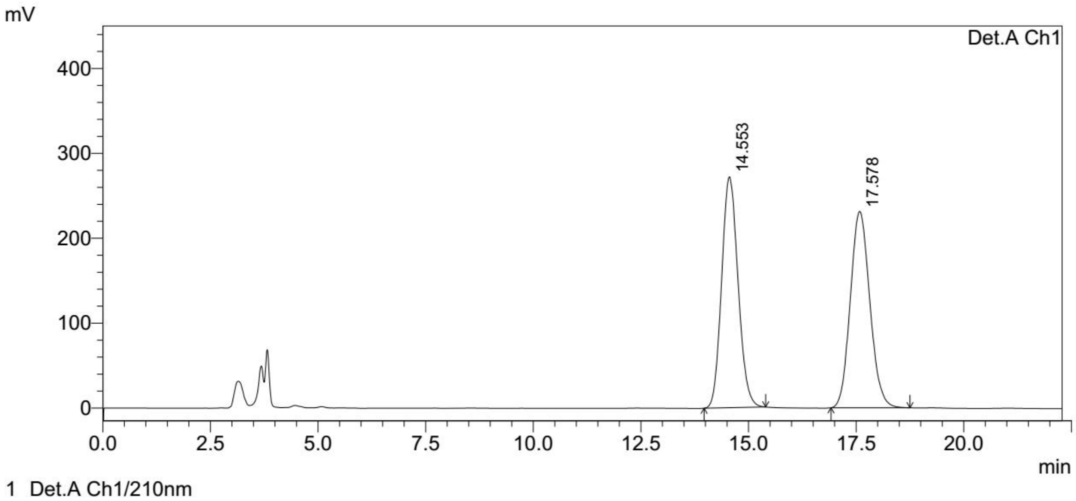

PeakTable

Detector A Ch1 210nm

Peak#	Ret. Time	Area	Height	Area %	Height %
1	14.553	7234558	271848	49.824	54.032
2	17.578	7285744	231272	50.176	45.968
Total		14520302	503120	100.000	100.000

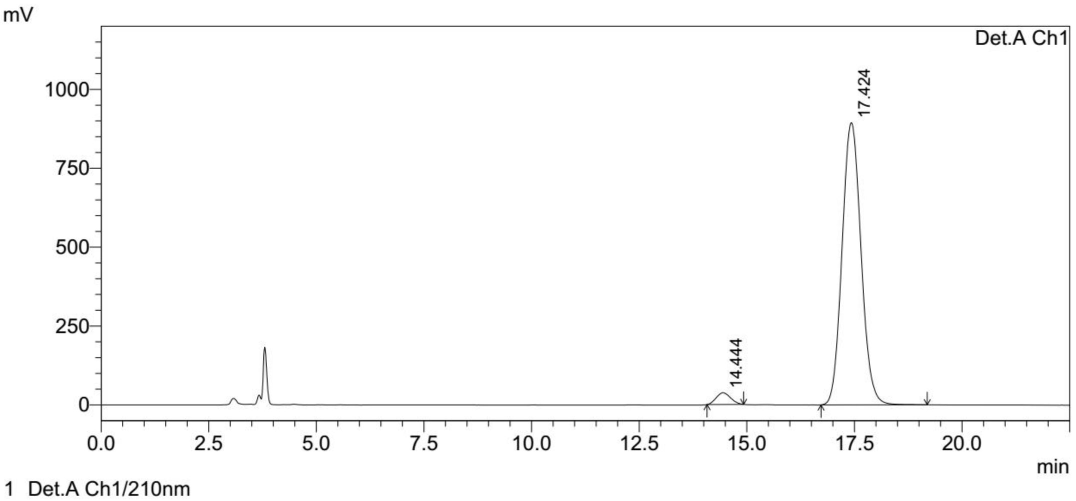

PeakTable

Detector A Ch1 210nm

Peak#	Ret. Time	Area	Height	Area %	Height %
1	14.444	857500	37044	3.032	3.981
2	17.424	27421702	893437	96.968	96.019
Total		28279202	930481	100.000	100.000

HPLC spectra of product 3k

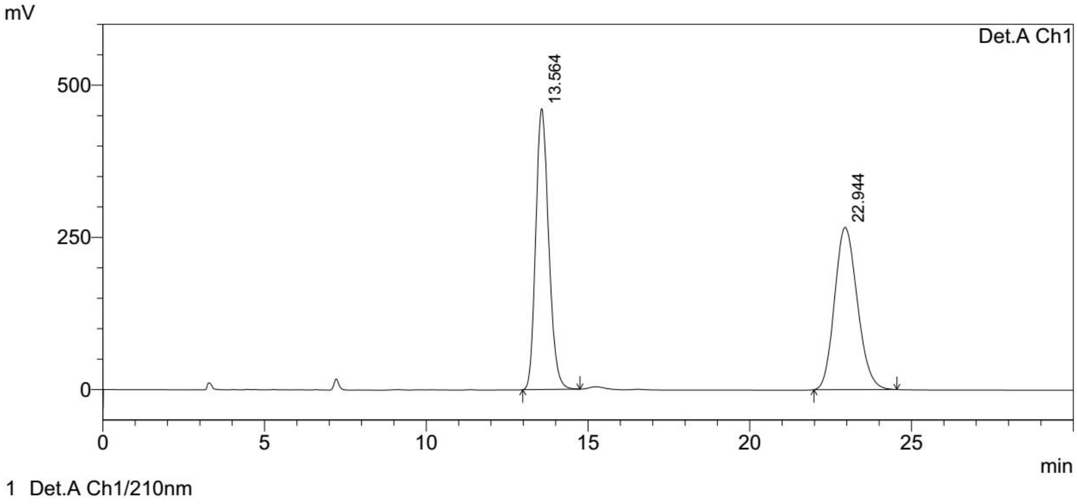

PeakTable

Detector A Ch1 210nm

Peak#	Ret. Time	Area	Height	Area %	Height %
1	13.564	12862741	460846	49.642	63.482
2	22.944	13048229	265105	50.358	36.518
Total		25910970	725951	100.000	100.000

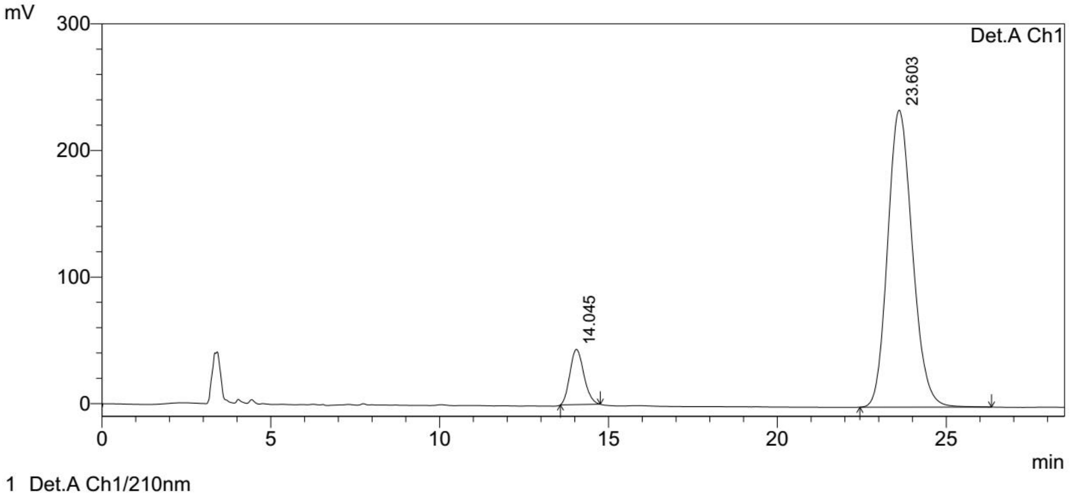

PeakTable

Detector A Ch1 210nm

Peak#	Ret. Time	Area	Height	Area %	Height %
1	14.045	1253527	43666	9.490	15.692
2	23.603	11956014	234602	90.510	84.308
Total		13209541	278267	100.000	100.000

HPLC spectra of product 3l

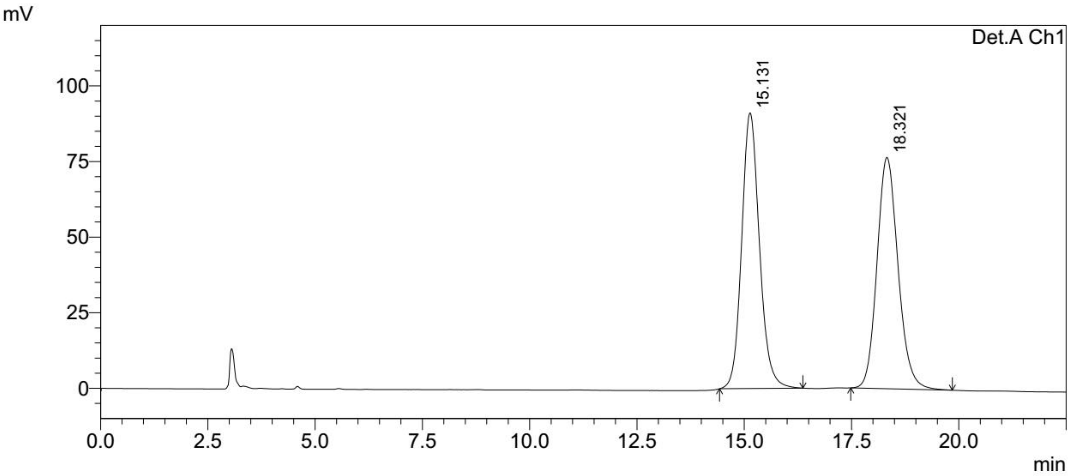

1 Det.A Ch1/210nm

PeakTable

Detector A Ch1 210nm

Peak#	Ret. Time	Area	Height	Area %	Height %
1	15.131	2625088	91167	50.130	54.368
2	18.321	2611500	76517	49.870	45.632
Total		5236588	167684	100.000	100.000

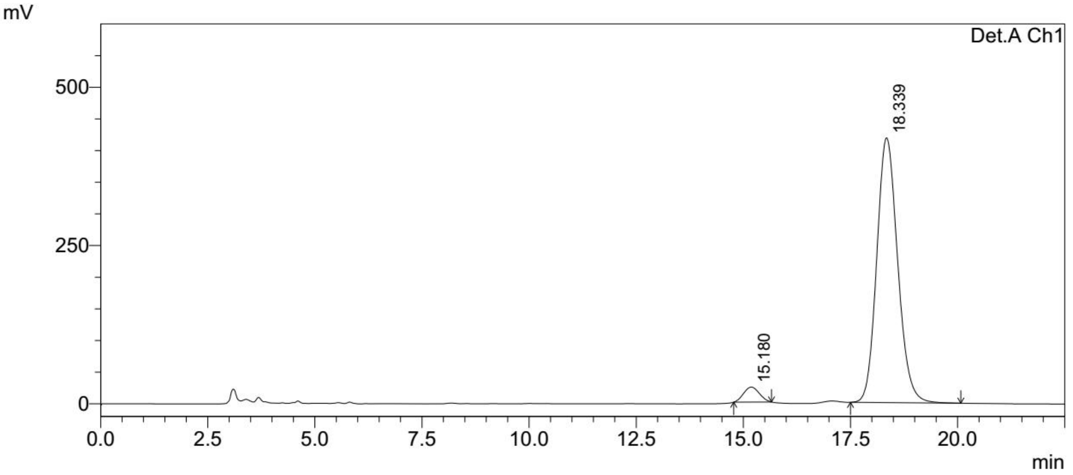

1 Det.A Ch1/210nm

PeakTable

Detector A Ch1 210nm

Peak#	Ret. Time	Area	Height	Area %	Height %
1	15.180	606563	23789	4.093	5.381
2	18.339	14211525	418312	95.907	94.619
Total		14818088	442101	100.000	100.000

HPLC spectra of product 3m

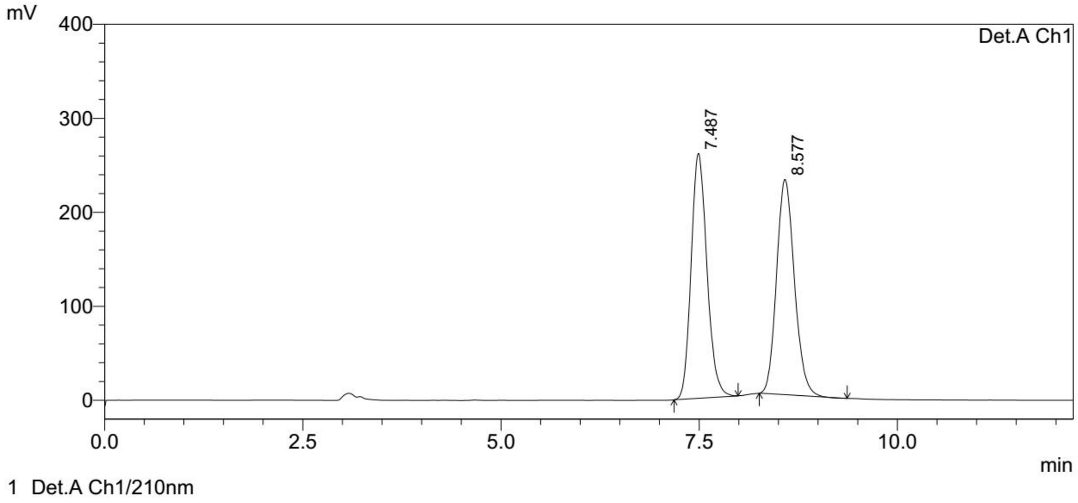

PeakTable

Detector A Ch1 210nm

Peak#	Ret. Time	Area	Height	Area %	Height %
1	7.487	3620623	260704	50.207	53.227
2	8.577	3590714	229092	49.793	46.773
Total		7211337	489796	100.000	100.000

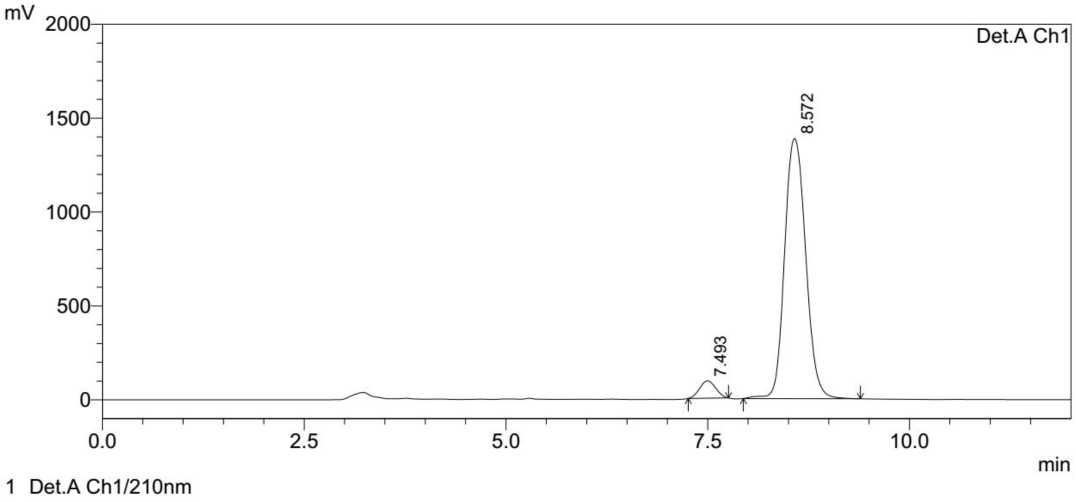

PeakTable

Detector A Ch1 210nm

Peak#	Ret. Time	Area	Height	Area %	Height %
1	7.493	1289888	92874	4.859	6.289
2	8.572	25256978	1383962	95.141	93.711
Total		26546866	1476837	100.000	100.000

HPLC spectra of product 3n

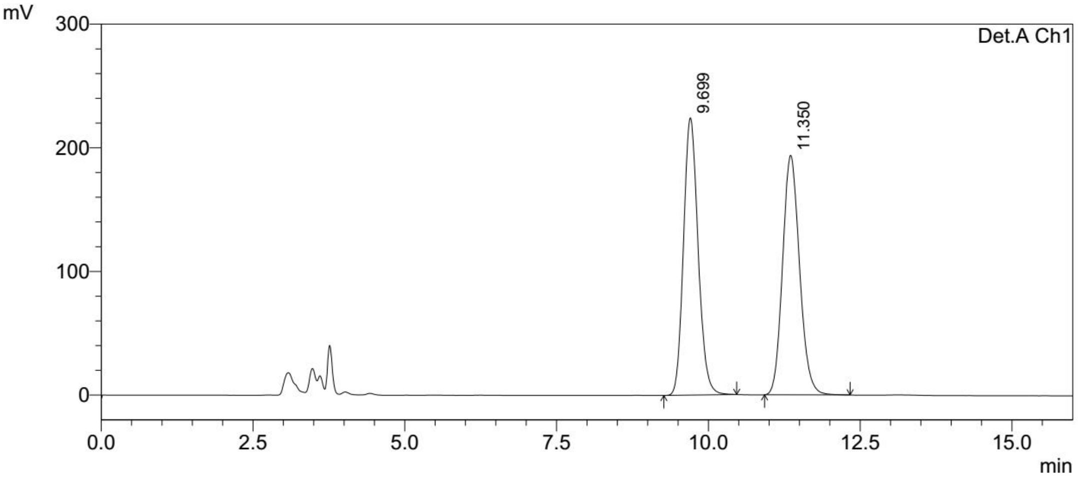

1 Det.A Ch1/210nm

PeakTable

Detector A Ch1 210nm

Peak#	Ret. Time	Area	Height	Area %	Height %
1	9.699	3749539	224252	49.871	53.665
2	11.350	3768946	193625	50.129	46.335
Total		7518486	417877	100.000	100.000

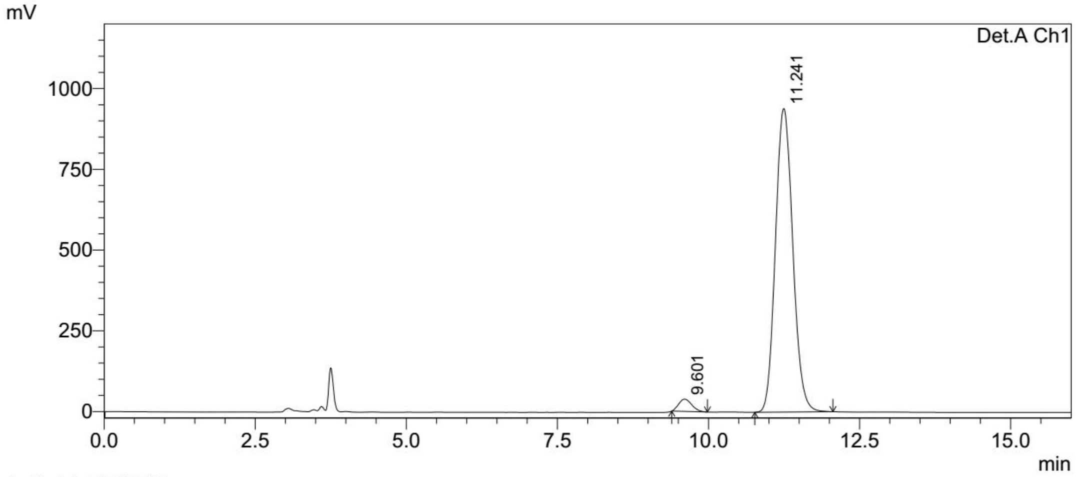

1 Det.A Ch1/210nm

PeakTable

Detector A Ch1 210nm

Peak#	Ret. Time	Area	Height	Area %	Height %
1	9.601	575904	38165	2.969	3.903
2	11.241	18818109	939768	97.031	96.097
Total		19394013	977933	100.000	100.000

HPLC spectra of product 3o

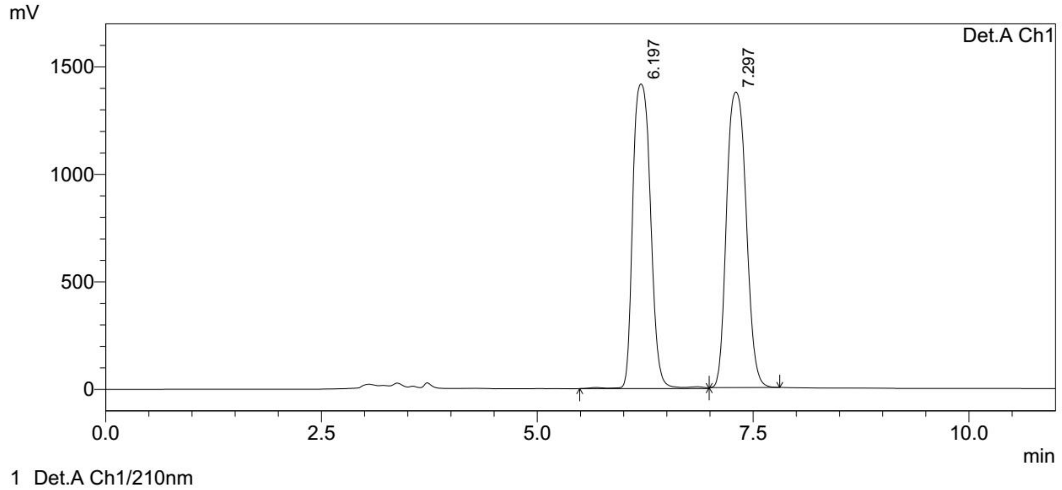

1 Det.A Ch1/210nm

PeakTable

Detector A Ch1 210nm

Peak#	Ret. Time	Area	Height	Area %	Height %
1	6.197	20296743	1415680	48.394	50.744
2	7.297	21644113	1374153	51.606	49.256
Total		41940856	2789833	100.000	100.000

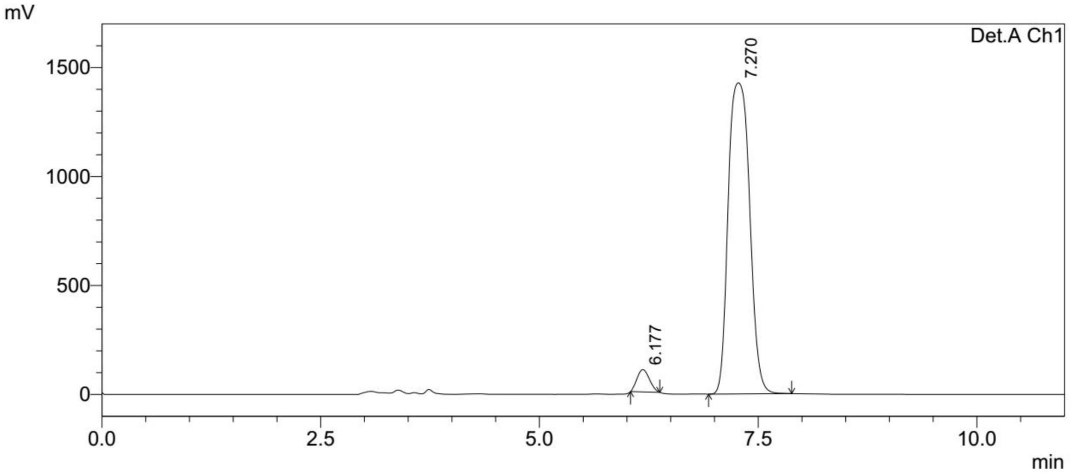

1 Det.A Ch1/210nm

PeakTable

Detector A Ch1 210nm

Peak#	Ret. Time	Area	Height	Area %	Height %
1	6.177	1006769	102113	3.981	6.675
2	7.270	24281445	1427776	96.019	93.325
Total		25288215	1529890	100.000	100.000

HPLC spectra of product 3p

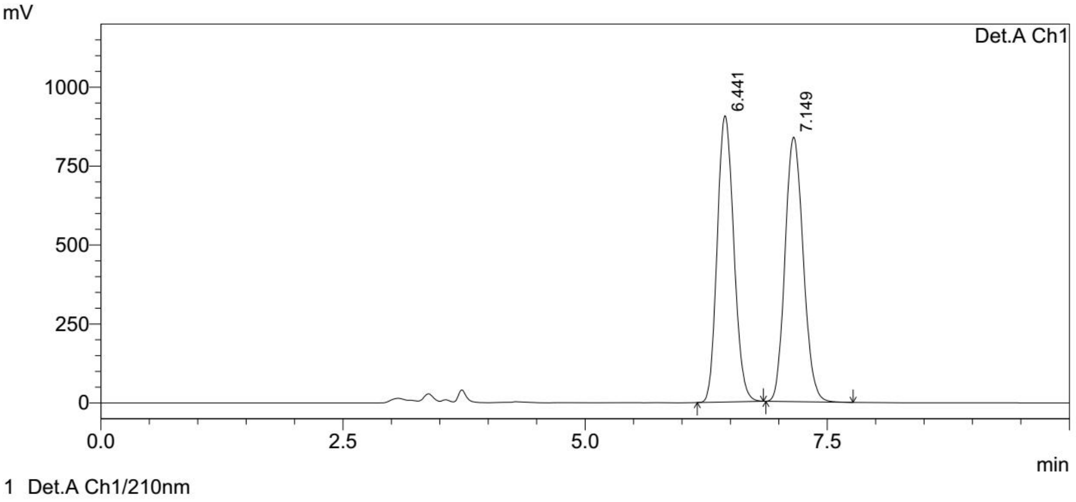

PeakTable

Detector A Ch1 210nm

Peak#	Ret. Time	Area	Height	Area %	Height %
1	6.441	10820553	906550	49.660	51.970
2	7.149	10968861	837820	50.340	48.030
Total		21789413	1744370	100.000	100.000

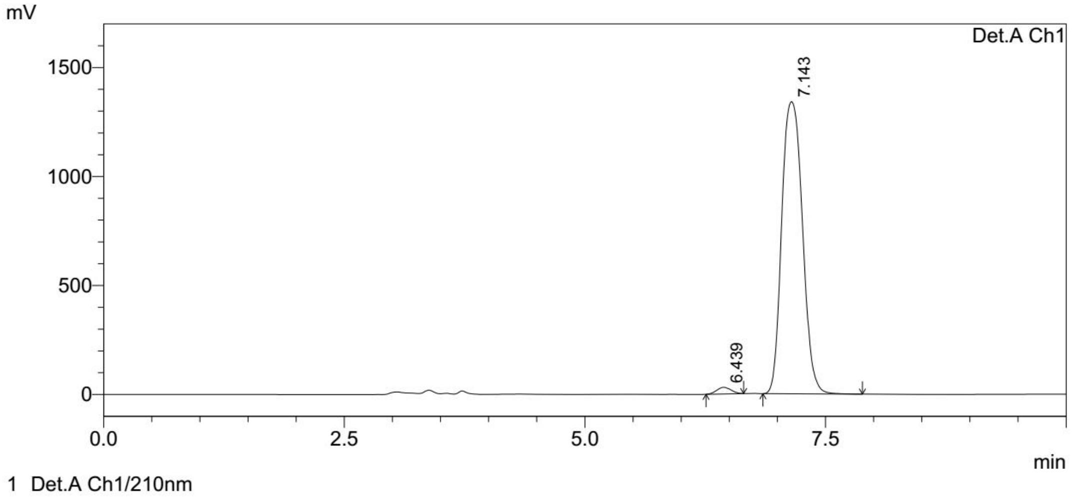

PeakTable

Detector A Ch1 210nm

Peak#	Ret. Time	Area	Height	Area %	Height %
1	6.439	317989	30513	1.559	2.226
2	7.143	20083216	1340267	98.441	97.774
Total		20401205	1370780	100.000	100.000

HPLC spectra of product 3q

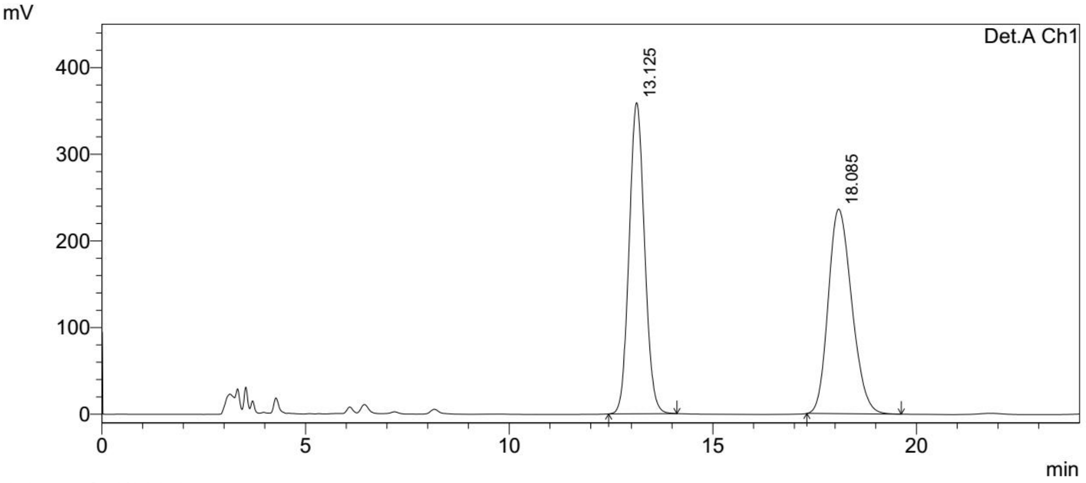

1 Det.A Ch1/210nm

PeakTable

Detector A Ch1 210nm

Peak#	Ret. Time	Area	Height	Area %	Height %
1	13.125	9459146	359003	49.921	60.324
2	18.085	9489254	236121	50.079	39.676
Total		18948400	595124	100.000	100.000

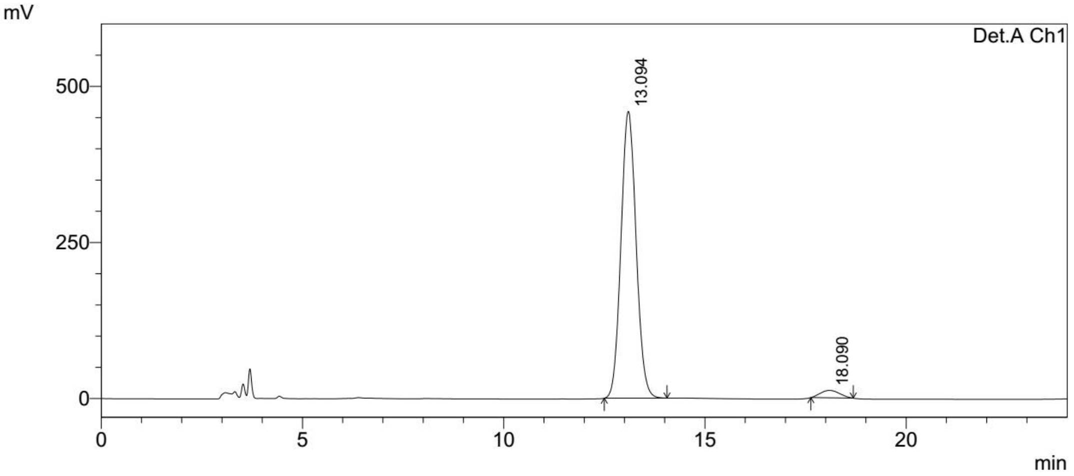

1 Det.A Ch1/210nm

PeakTable

Detector A Ch1 210nm

Peak#	Ret. Time	Area	Height	Area %	Height %
1	13.094	12022649	459456	96.812	97.465
2	18.090	395950	11950	3.188	2.535
Total		12418599	471406	100.000	100.000

HPLC spectra of product 3r

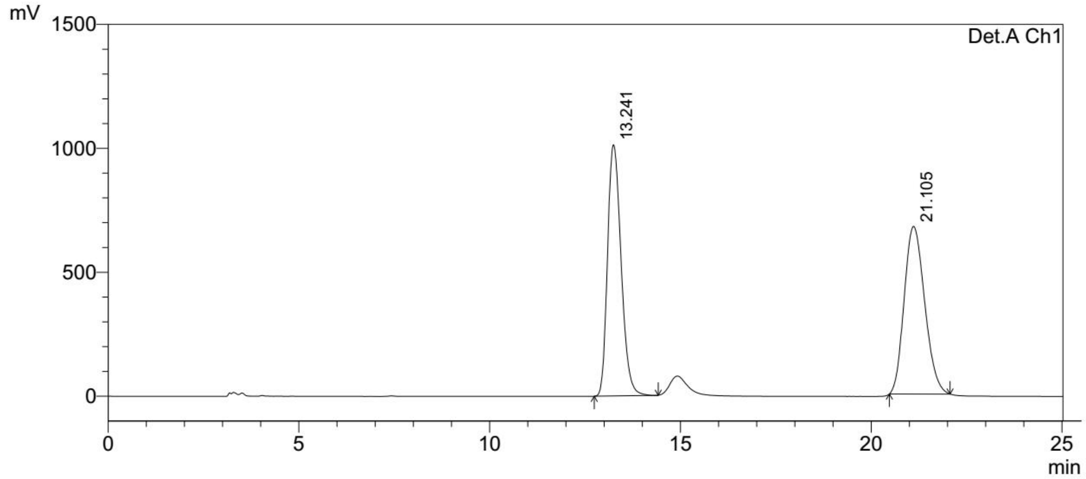

1 Det.A Ch1/210nm

PeakTable

Detector A Ch1 210nm

Peak#	Ret. Time	Area	Height	Area %	Height %
1	13.241	25013281	1013302	49.734	59.966
2	21.105	25280851	676498	50.266	40.034
Total		50294133	1689801	100.000	100.000

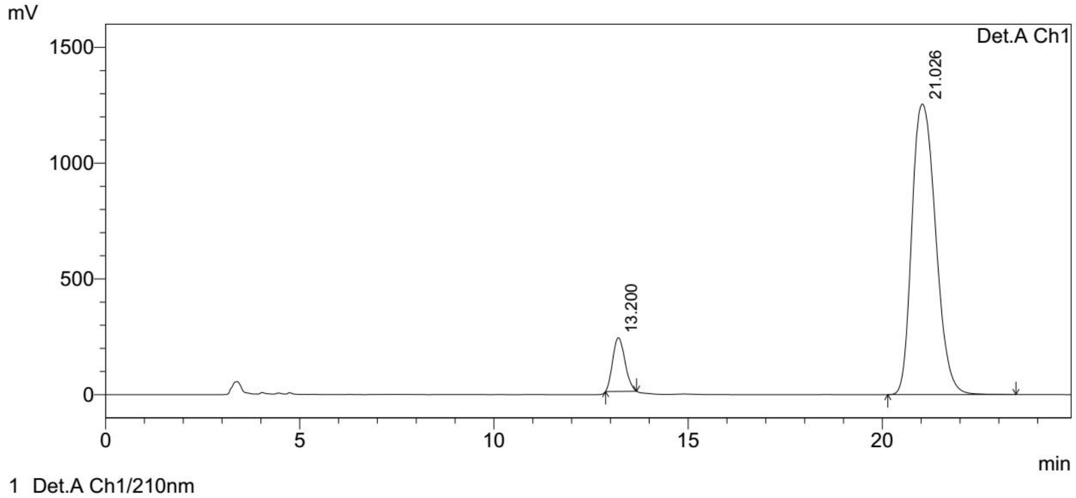

1 Det.A Ch1/210nm

PeakTable

Detector A Ch1 210nm

Peak#	Ret. Time	Area	Height	Area %	Height %
1	13.200	5090546	232218	8.678	15.617
2	21.026	53567991	1254741	91.322	84.383
Total		58658537	1486958	100.000	100.000

HPLC spectra of product 3s

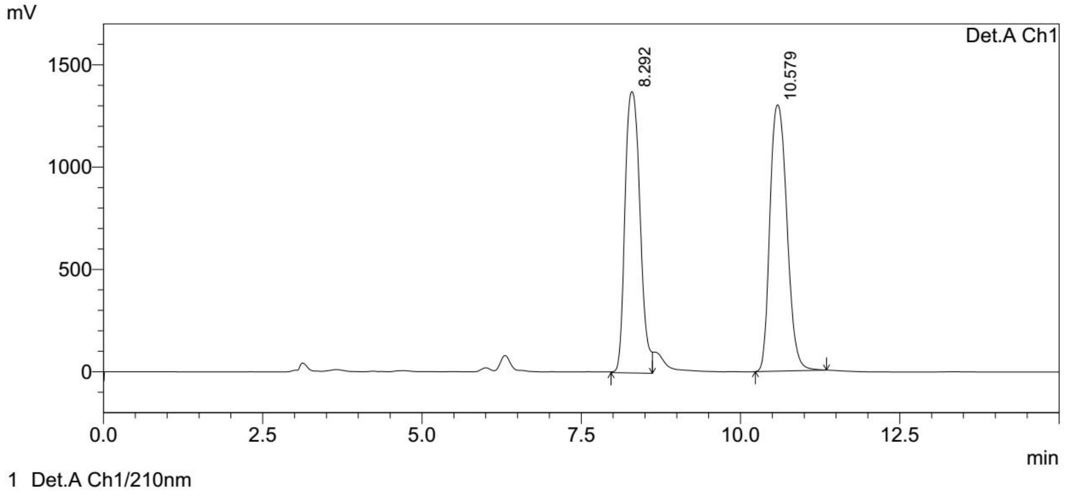

PeakTable

Detector A Ch1 210nm

Peak#	Ret. Time	Area	Height	Area %	Height %
1	8.292	22628327	1375186	48.391	51.380
2	10.579	24133155	1301303	51.609	48.620
Total		46761482	2676489	100.000	100.000

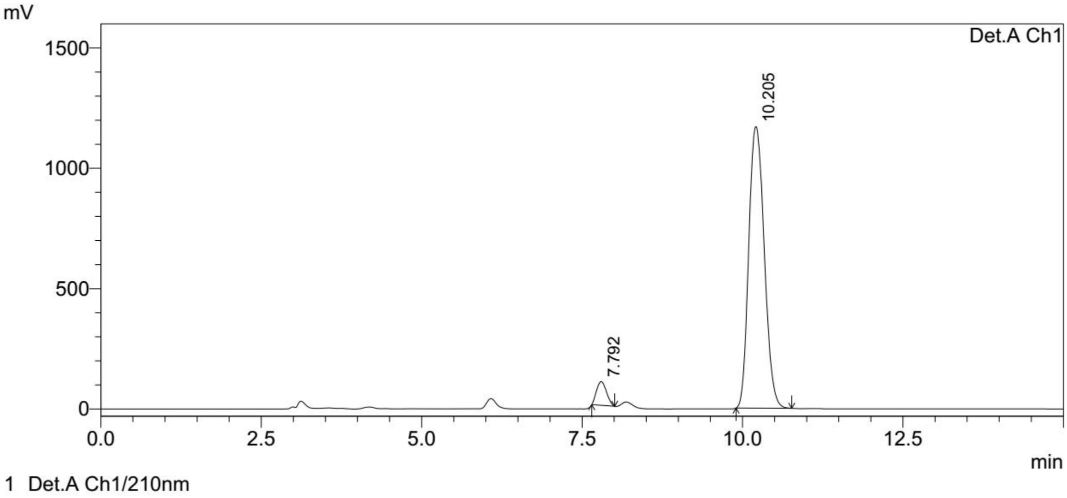

PeakTable

Detector A Ch1 210nm

Peak#	Ret. Time	Area	Height	Area %	Height %
1	7.792	1062456	98513	5.120	7.765
2	10.205	19687445	1170138	94.880	92.235
Total		20749901	1268651	100.000	100.000

HPLC spectra of product 3t

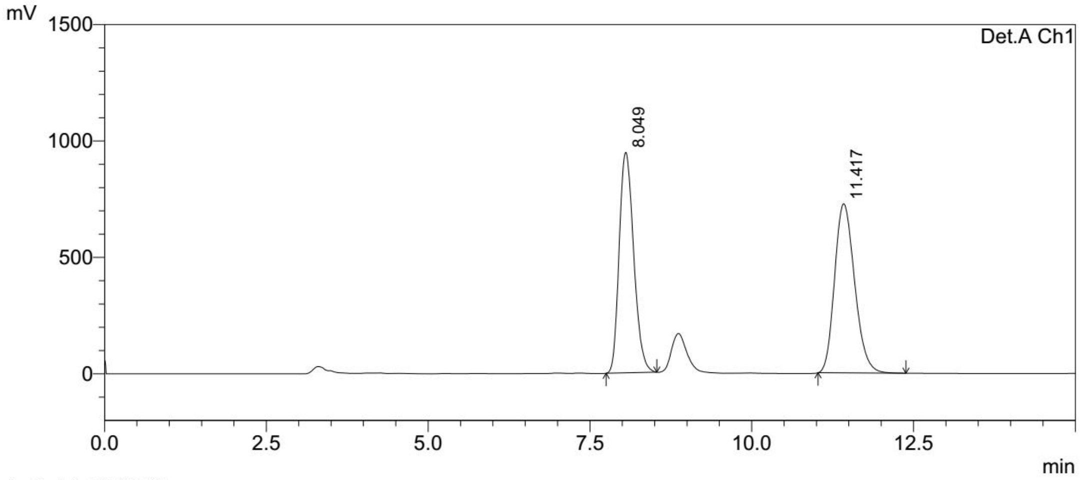

1 Det.A Ch1/210nm

PeakTable

Detector A Ch1 210nm

Peak#	Ret. Time	Area	Height	Area %	Height %
1	8.049	14769695	946758	48.538	56.596
2	11.417	15659160	726081	51.462	43.404
Total		30428854	1672838	100.000	100.000

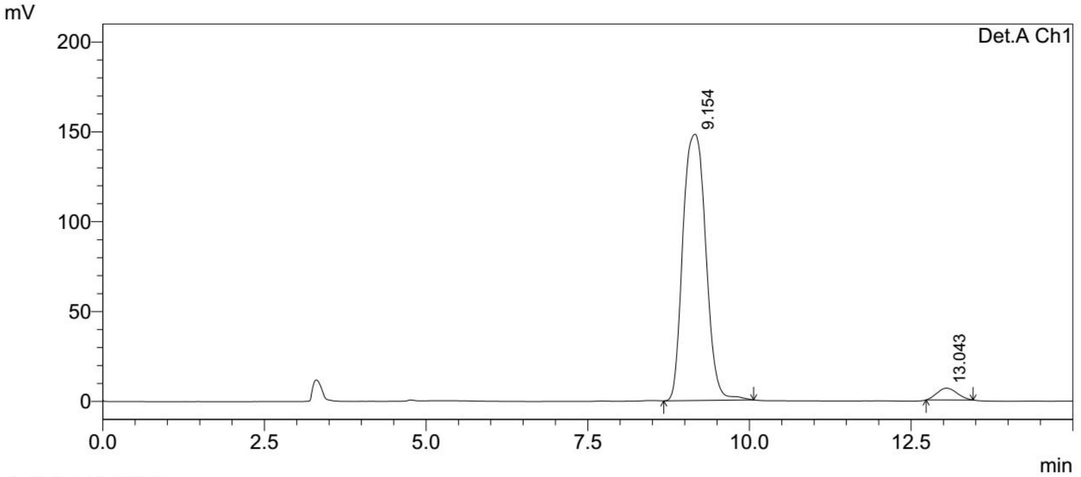

1 Det.A Ch1/210nm

PeakTable

Detector A Ch1 210nm

Peak#	Ret. Time	Area	Height	Area %	Height %
1	9.154	3661975	148219	96.291	95.744
2	13.043	141054	6588	3.709	4.256
Total		3803029	154808	100.000	100.000

HPLC spectra of product 3u

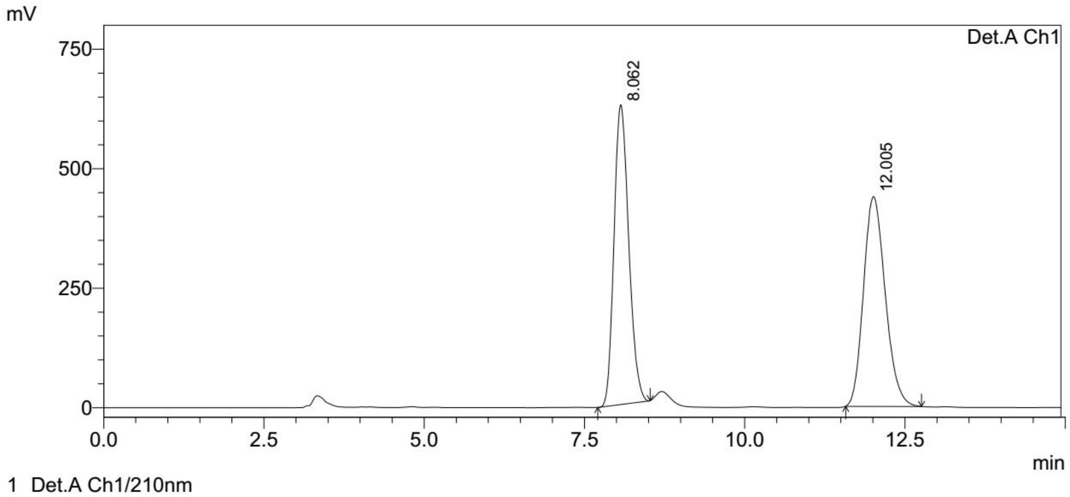

1 Det.A Ch1/210nm

PeakTable

Detector A Ch1 210nm

Peak#	Ret. Time	Area	Height	Area %	Height %
1	8.062	10012950	626271	49.144	58.799
2	12.005	10361854	438826	50.856	41.201
Total		20374804	1065097	100.000	100.000

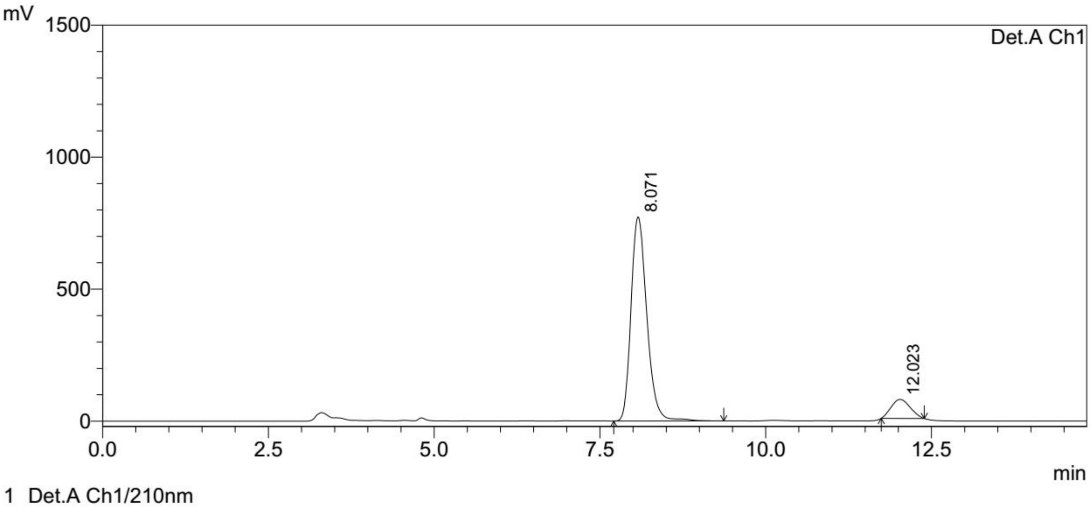

1 Det.A Ch1/210nm

PeakTable

Detector A Ch1 210nm

Peak#	Ret. Time	Area	Height	Area %	Height %
1	8.071	12806607	772914	89.934	91.490
2	12.023	1433337	71889	10.066	8.510
Total		14239944	844803	100.000	100.000

HPLC spectra of product 3v

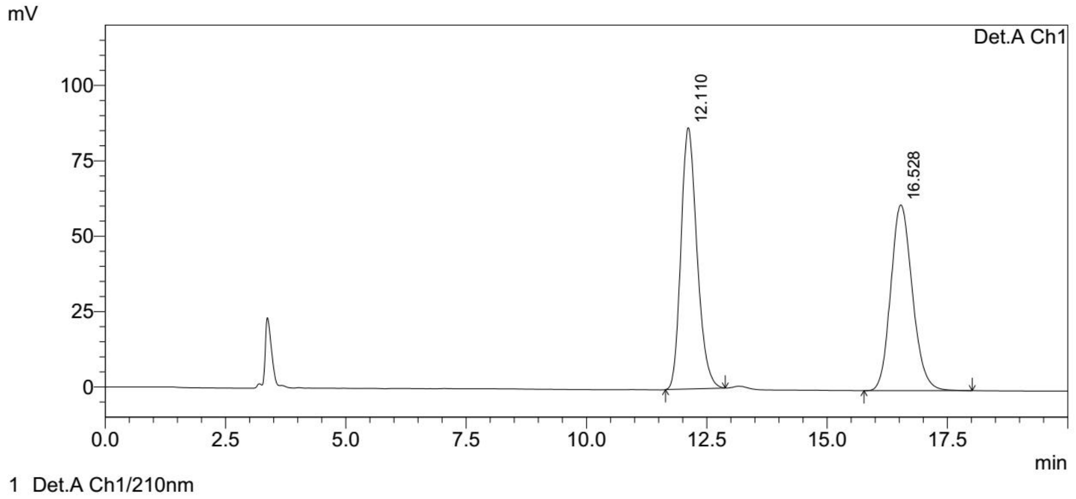

PeakTable

Detector A Ch1 210nm

Peak#	Ret. Time	Area	Height	Area %	Height %
1	12.110	2013778	86669	51.116	58.442
2	16.528	1925834	61630	48.884	41.558
Total		3939612	148299	100.000	100.000

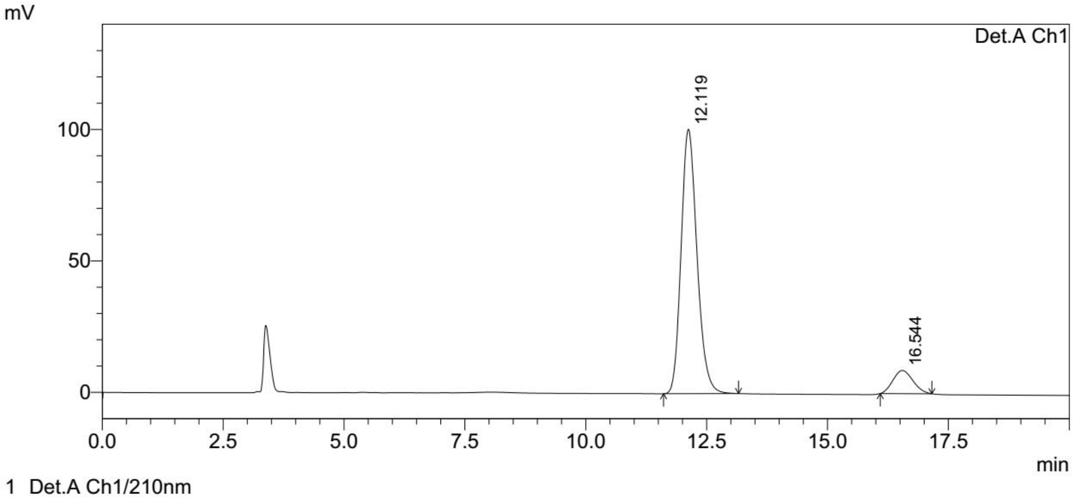

PeakTable

Detector A Ch1 210nm

Peak#	Ret. Time	Area	Height	Area %	Height %
1	12.119	2299860	100584	90.071	91.954
2	16.544	253520	8801	9.929	8.046
Total		2553380	109386	100.000	100.000

HPLC spectra of product 3w

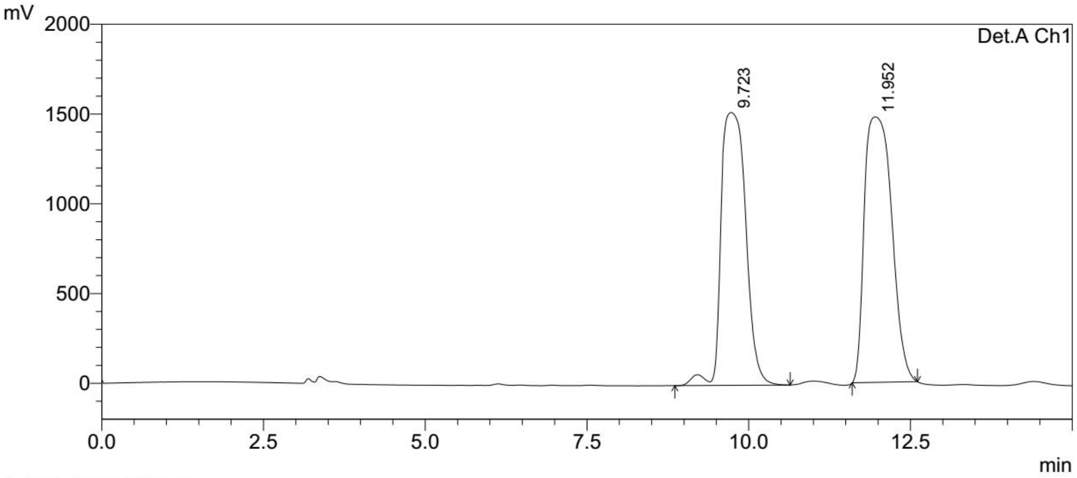

1 Det.A Ch1/210nm

PeakTable

Detector A Ch1 210nm

Peak#	Ret. Time	Area	Height	Area %	Height %
1	9.723	40620918	1519896	48.278	50.676
2	11.952	43519193	1479358	51.722	49.324
Total		84140110	2999254	100.000	100.000

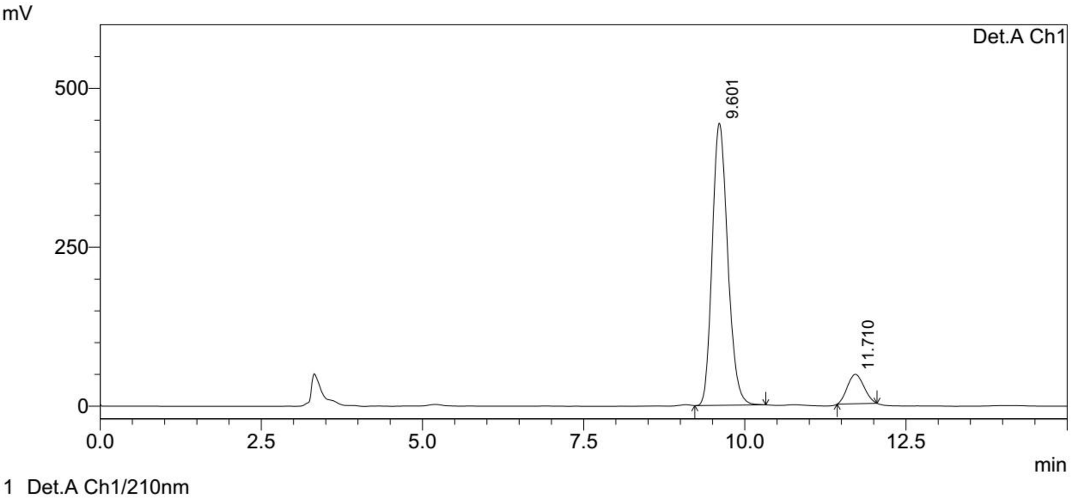

1 Det.A Ch1/210nm

PeakTable

Detector A Ch1 210nm

Peak#	Ret. Time	Area	Height	Area %	Height %
1	9.601	7436988	443917	90.007	90.571
2	11.710	825734	46213	9.993	9.429
Total		8262722	490130	100.000	100.000

HPLC spectra of product 3x

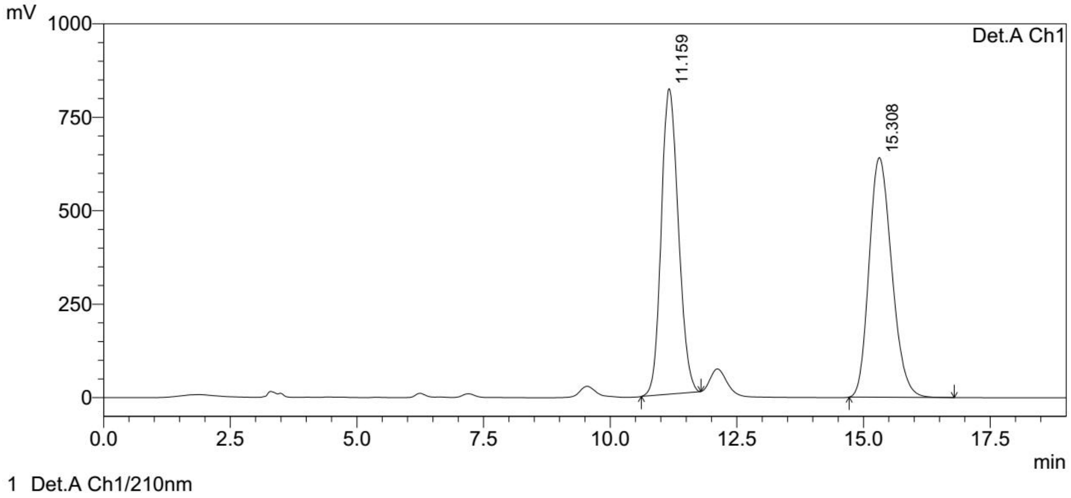

PeakTable

Detector A Ch1 210nm

Peak#	Ret. Time	Area	Height	Area %	Height %
1	11.159	19316245	817213	49.208	56.044
2	15.308	19937653	640953	50.792	43.956
Total		39253898	1458166	100.000	100.000

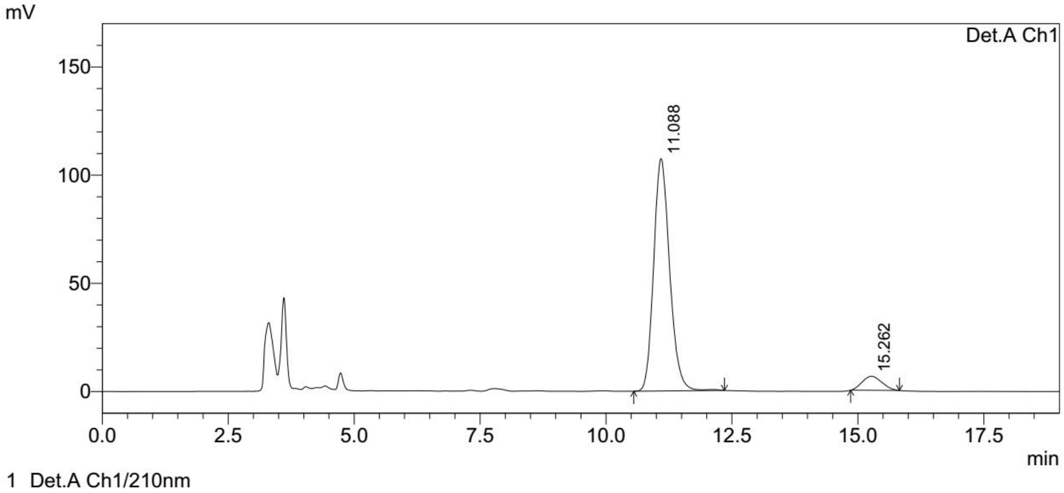

PeakTable

Detector A Ch1 210nm

Peak#	Ret. Time	Area	Height	Area %	Height %
1	11.088	2375782	107389	93.086	94.305
2	15.262	176475	6486	6.914	5.695
Total		2552258	113875	100.000	100.000

HPLC spectra of product 3y

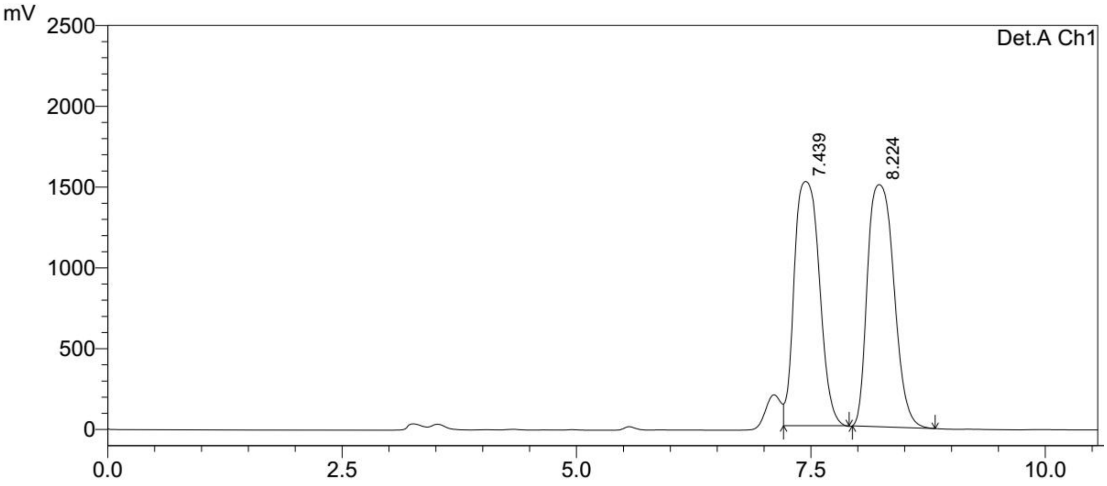

1 Det.A Ch1/210nm

PeakTable

Detector A Ch1 210nm

Peak#	Ret. Time	Area	Height	Area %	Height %
1	7.439	28165177	1511285	48.874	50.228
2	8.224	29462861	1497553	51.126	49.772
Total		57628038	3008837	100.000	100.000

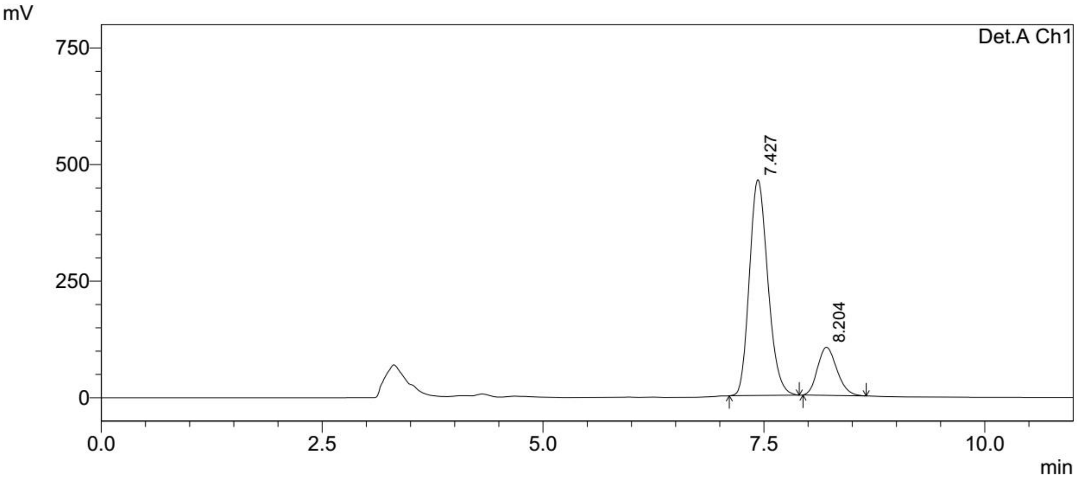

1 Det.A Ch1/210nm

PeakTable

Detector A Ch1 210nm

Peak#	Ret. Time	Area	Height	Area %	Height %
1	7.427	6711439	462757	81.051	81.791
2	8.204	1569041	103023	18.949	18.209
Total		8280481	565779	100.000	100.000

HPLC spectra of product 3z

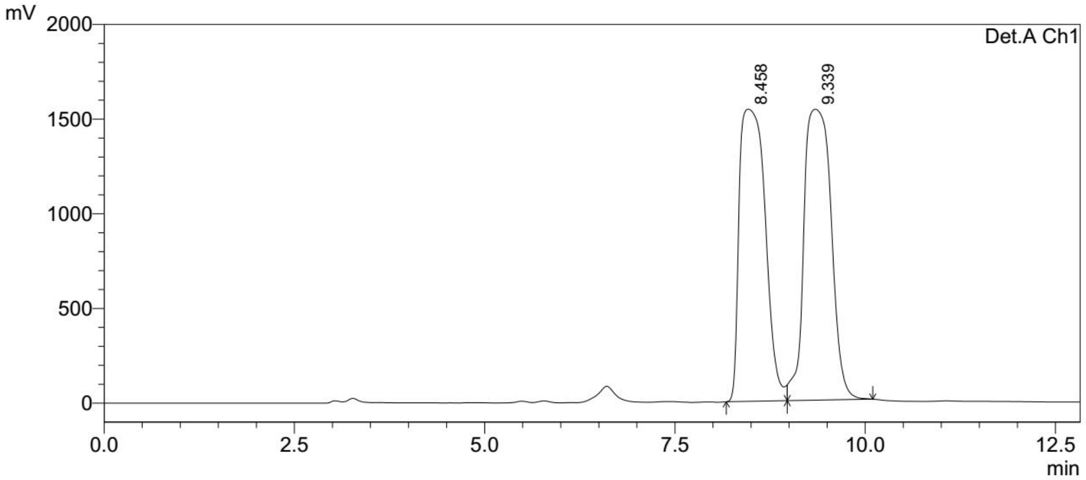

1 Det.A Ch1/210nm

PeakTable

Detector A Ch1 210nm

Peak#	Ret. Time	Area	Height	Area %	Height %
1	8.458	36760772	1543524	48.970	50.109
2	9.339	38307500	1536794	51.030	49.891
Total		75068273	3080318	100.000	100.000

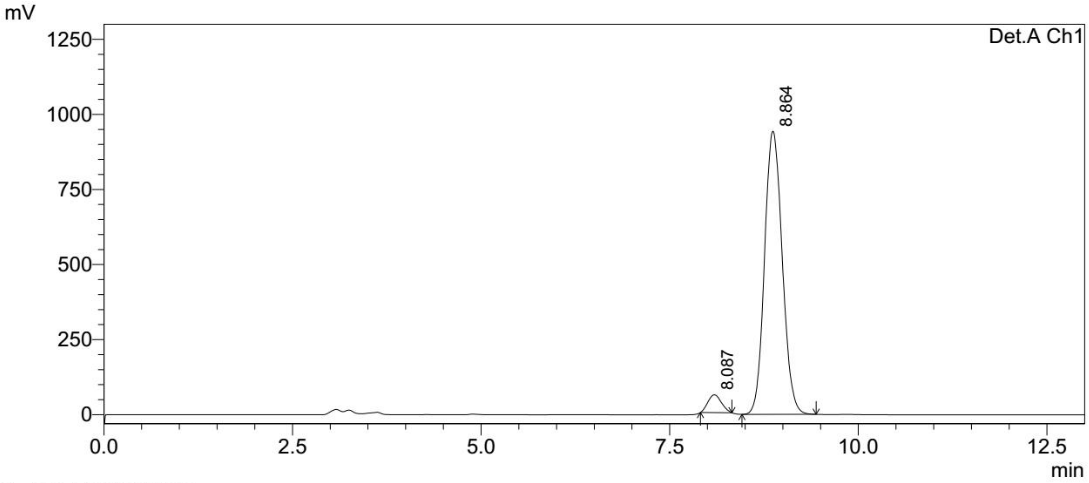

1 Det.A Ch1/210nm

PeakTable

Detector A Ch1 210nm

Peak#	Ret. Time	Area	Height	Area %	Height %
1	8.087	738547	59352	4.567	5.921
2	8.864	15431271	943119	95.433	94.079
Total		16169818	1002471	100.000	100.000

HPLC spectra of product 5

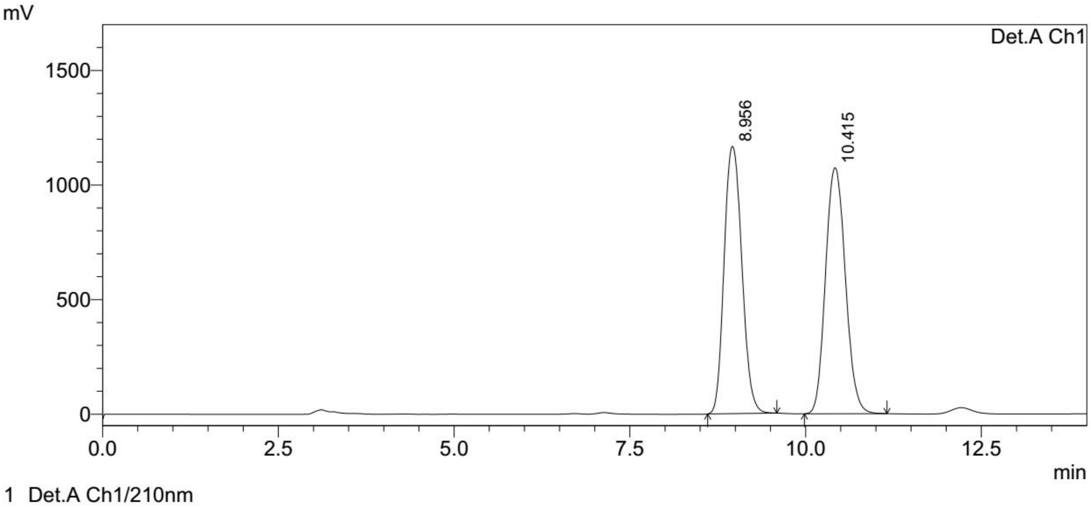

1 Det.A Ch1/210nm

PeakTable

Detector A Ch1 210nm

Peak#	Ret. Time	Area	Height	Area %	Height %
1	8.956	20483241	1166507	49.194	52.062
2	10.415	21154699	1074083	50.806	47.938
Total		41637940	2240590	100.000	100.000

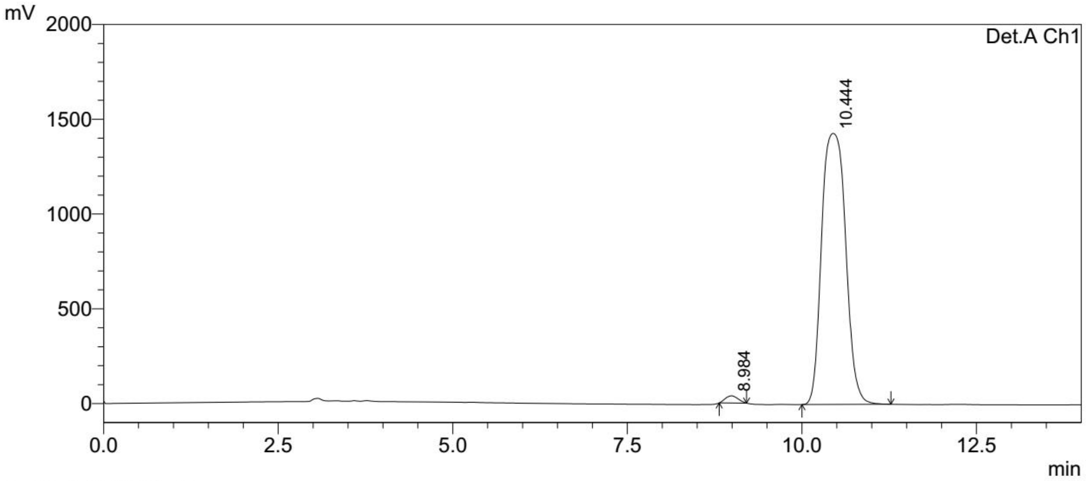

1 Det.A Ch1/210nm

PeakTable

Detector A Ch1 210nm

Peak#	Ret. Time	Area	Height	Area %	Height %
1	8.984	477113	37851	1.374	2.579
2	10.444	34236164	1430057	98.626	97.421
Total		34713277	1467908	100.000	100.000

HPLC spectra of product 6

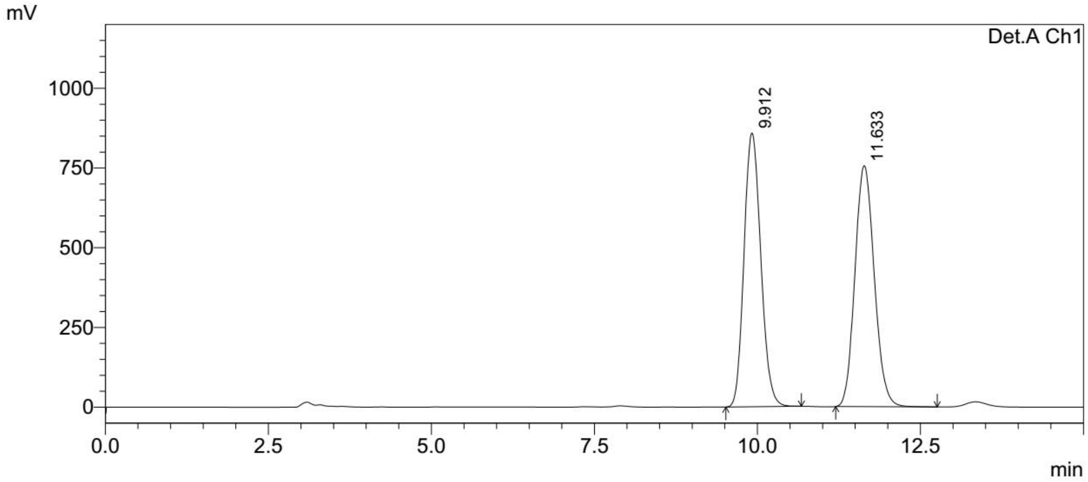

1 Det.A Ch1/210nm

PeakTable

Detector A Ch1 210nm

Peak#	Ret. Time	Area	Height	Area %	Height %
1	9.912	15414734	858914	49.684	53.211
2	11.633	15610657	755245	50.316	46.789
Total		31025391	1614159	100.000	100.000

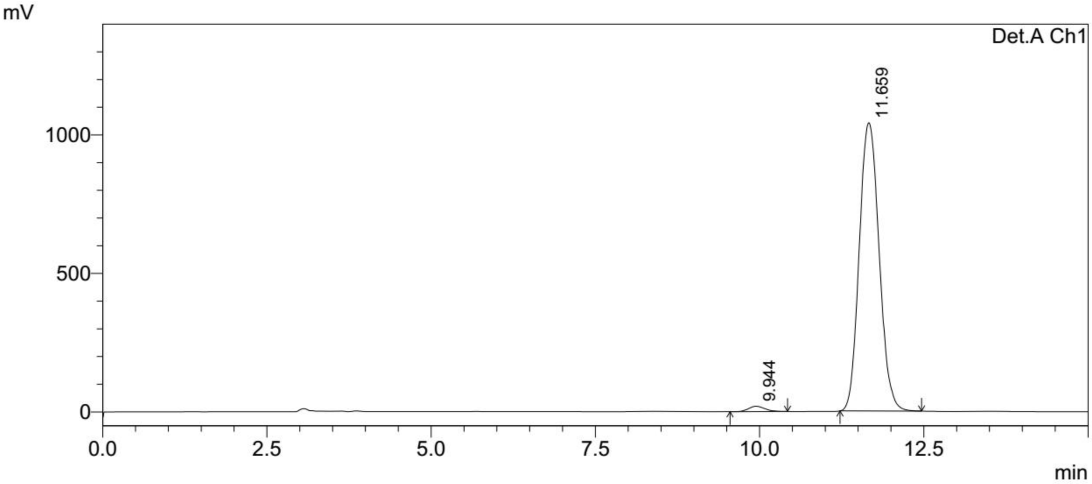

1 Det.A Ch1/210nm

PeakTable

Detector A Ch1 210nm

Peak#	Ret. Time	Area	Height	Area %	Height %
1	9.944	327810	19326	1.474	1.823
2	11.659	21905174	1040647	98.526	98.177
Total		22232984	1059973	100.000	100.000

HPLC spectra of product 7

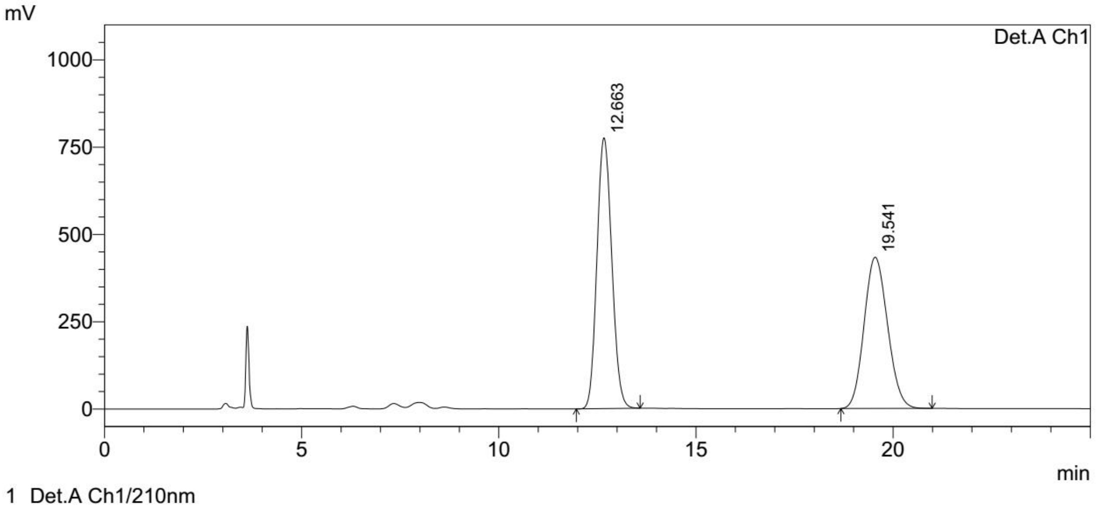

PeakTable

Detector A Ch1 210nm

Peak#	Ret. Time	Area	Height	Area %	Height %
1	12.663	20303795	775585	53.112	64.160
2	19.541	17924748	433244	46.888	35.840
Total		38228543	1208830	100.000	100.000

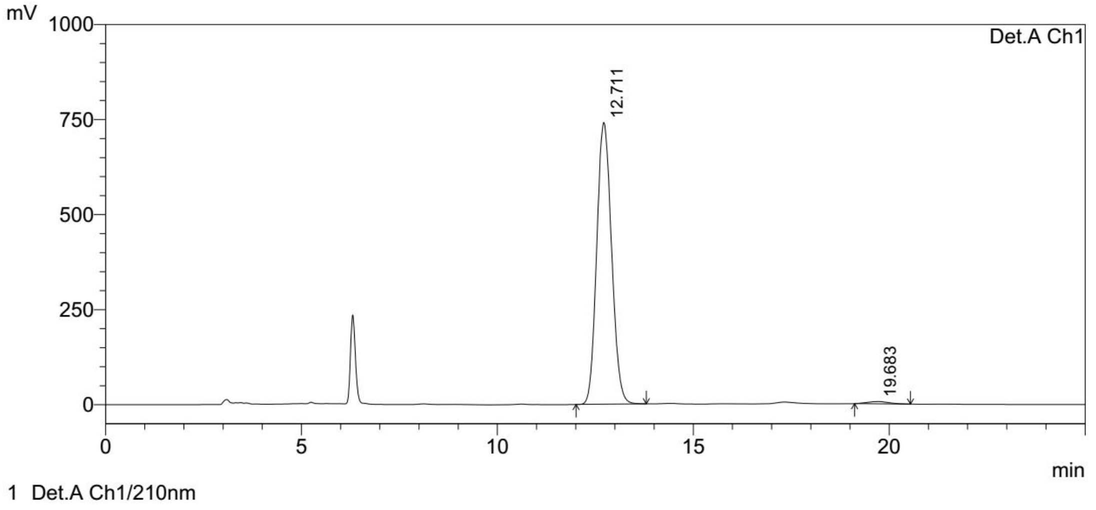

PeakTable

Detector A Ch1 210nm

Peak#	Ret. Time	Area	Height	Area %	Height %
1	12.711	19531153	741212	98.843	99.202
2	19.683	228568	5959	1.157	0.798
Total		19759721	747171	100.000	100.000

HPLC spectra of product 8

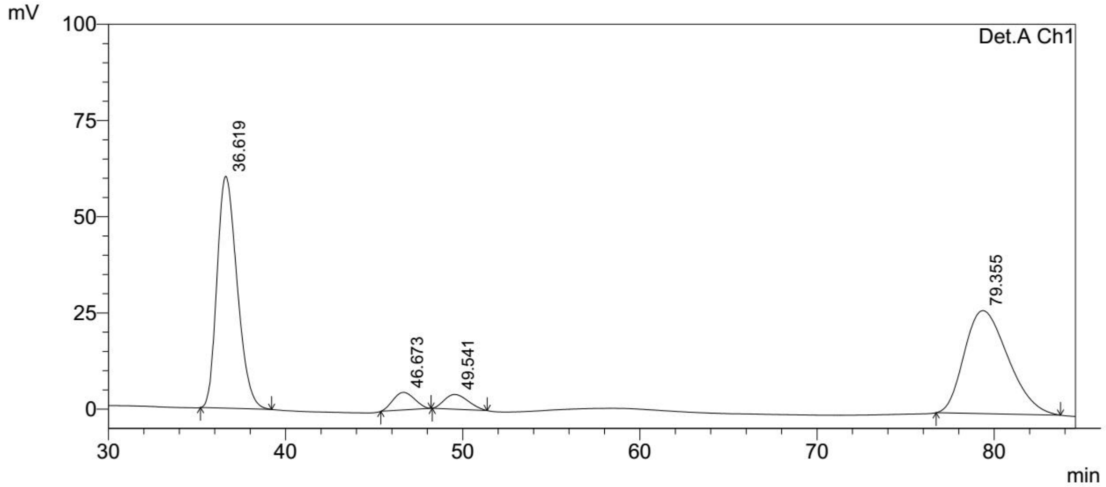

PeakTable

Detector A Ch1 210nm

Peak#	Ret. Time	Area	Height	Area %	Height %
1	36.619	4757725	60261	47.076	63.152
2	46.673	385677	4565	3.816	4.784
3	49.541	358781	3827	3.550	4.010
4	79.355	4604351	26769	45.558	28.054
Total		10106535	95421	100.000	100.000

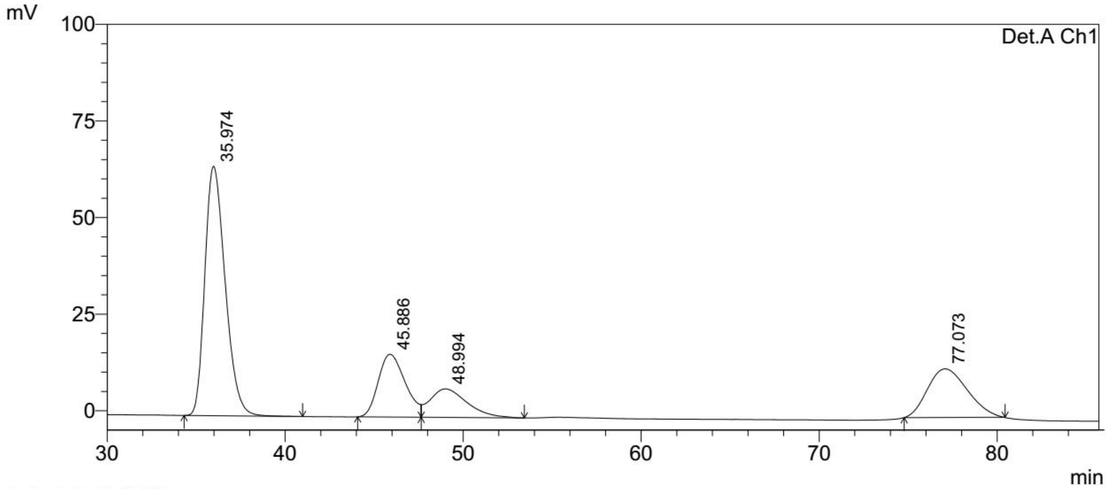

PeakTable

Detector A Ch1 210nm

Peak#	Ret. Time	Area	Height	Area %	Height %
1	35.974	5130028	64528	51.951	64.055
2	45.886	1706434	16229	17.281	16.110
3	48.994	1074504	7390	10.881	7.335
4	77.073	1963739	12592	19.887	12.500
Total		9874704	100738	100.000	100.000

HPLC spectra of product 9

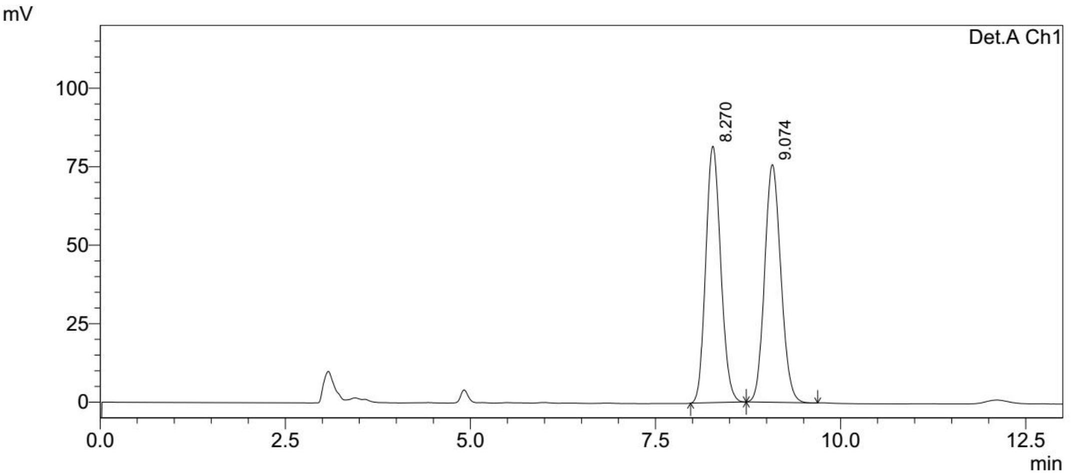

1 Det.A Ch1/210nm

PeakTable

Detector A Ch1 210nm

Peak#	Ret. Time	Area	Height	Area %	Height %
1	8.270	1147835	81554	49.779	51.934
2	9.074	1158026	75479	50.221	48.066
Total		2305860	157034	100.000	100.000

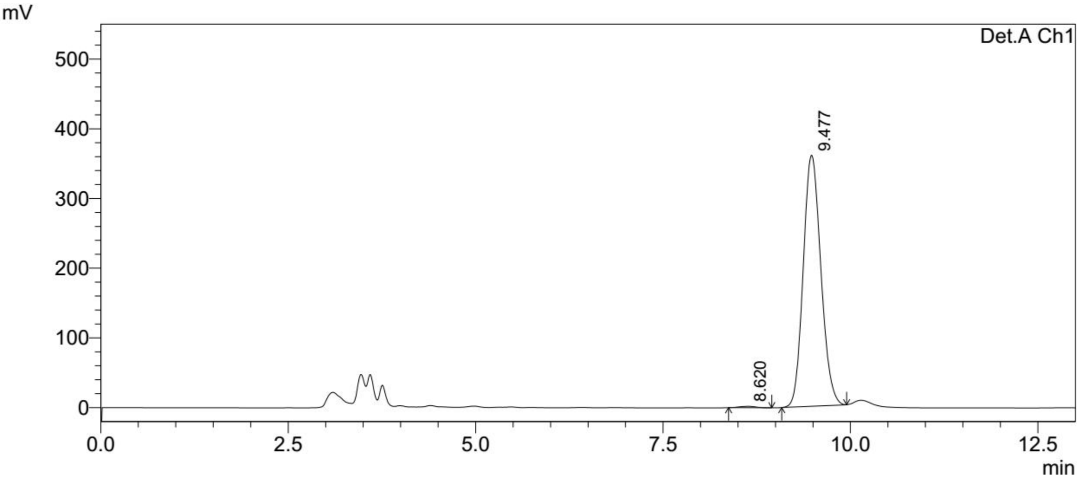

1 Det.A Ch1/210nm

PeakTable

Detector A Ch1 210nm

Peak#	Ret. Time	Area	Height	Area %	Height %
1	8.620	31340	2185	0.528	0.603
2	9.477	5901439	360186	99.472	99.397
Total		5932779	362371	100.000	100.000

HPLC spectra of product 10

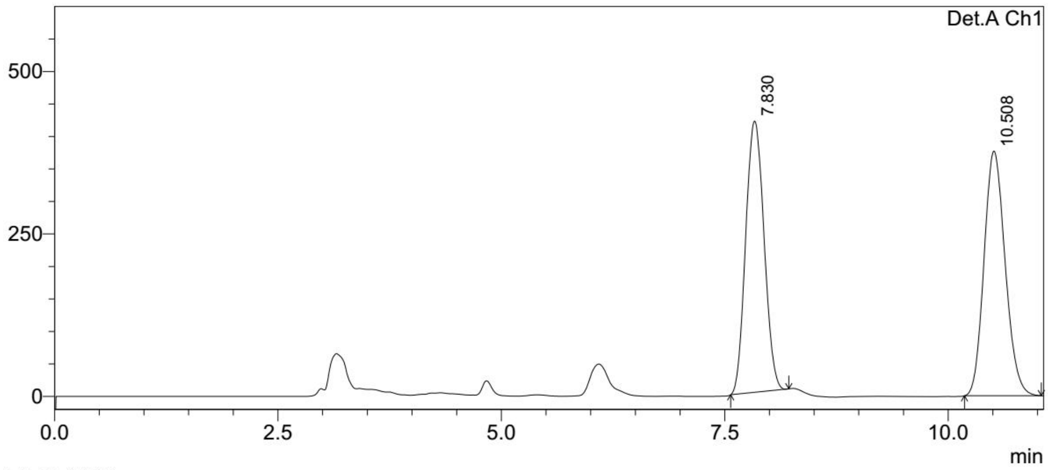

1 Det.A Ch1/210nm

PeakTable

Detector A Ch1 210nm

Peak#	Ret. Time	Area	Height	Area %	Height %
1	7.830	5984493	417634	49.122	52.597
2	10.508	6198338	376386	50.878	47.403
Total		12182830	794020	100.000	100.000

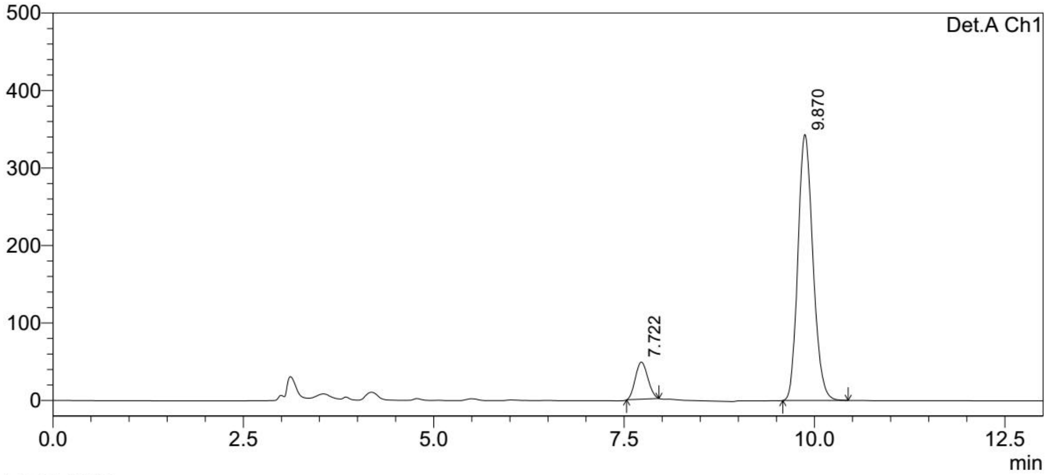

1 Det.A Ch1/210nm

PeakTable

Detector A Ch1 210nm

Peak#	Ret. Time	Area	Height	Area %	Height %
1	7.722	545695	48013	10.386	12.271
2	9.870	4708648	343273	89.614	87.729
Total		5254343	391286	100.000	100.000

HPLC spectra of product 11

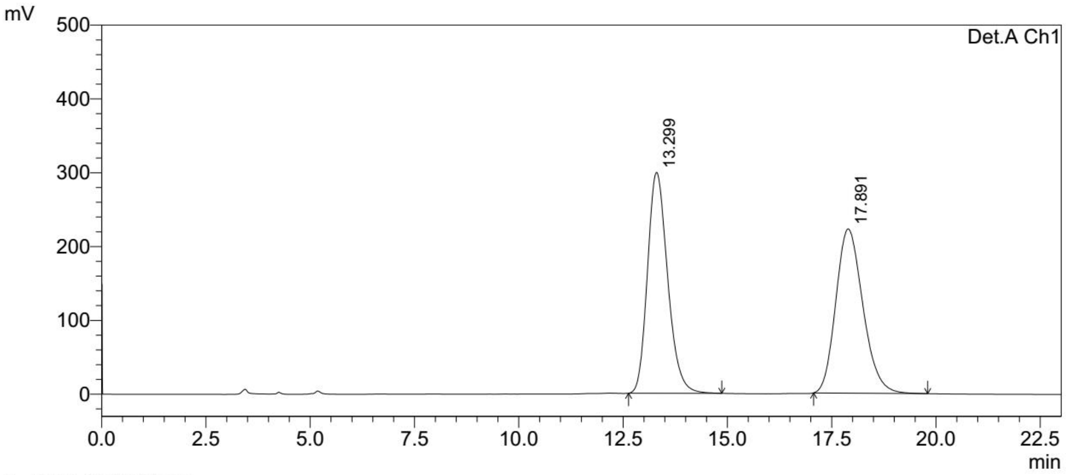

1 Det.A Ch1/210nm

PeakTable

Detector A Ch1 210nm

Peak#	Ret. Time	Area	Height	Area %	Height %
1	13.299	10049531	298177	49.985	57.287
2	17.891	10055464	222324	50.015	42.713
Total		20104995	520501	100.000	100.000

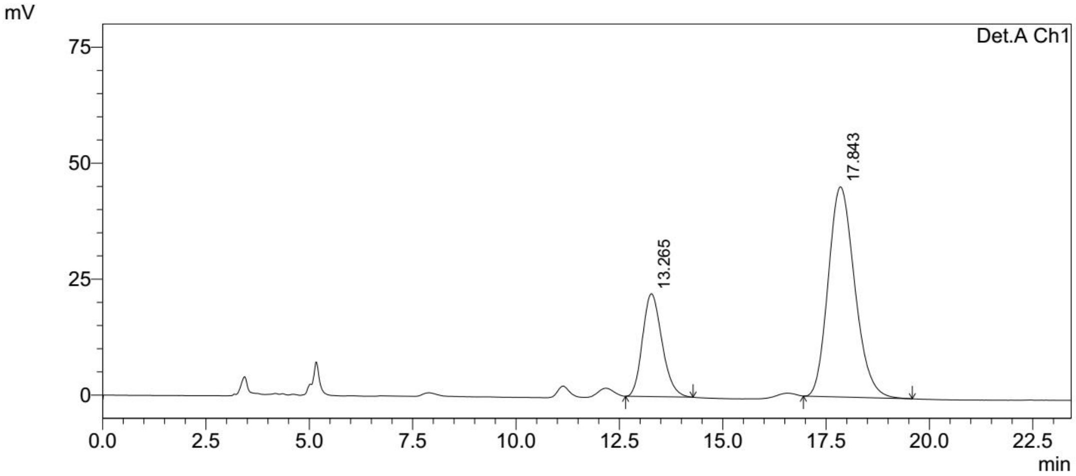

1 Det.A Ch1/210nm

PeakTable

Detector A Ch1 210nm

Peak#	Ret. Time	Area	Height	Area %	Height %
1	13.265	729061	22166	26.518	32.872
2	17.843	2020282	45265	73.482	67.128
Total		2749343	67431	100.000	100.000

11. References

1. Z. Huang, X. Huang, B. Li, C. Mou, S. Yang, B.-A. Song and Y. R. Chi, *J. Am. Chem. Soc.,* 2016, **138**, 7524.
2. G.-H. Yang, Y. Li, X. Li and J.-P. *Cheng, Chem. Sci*., 2019, **10**, 4322.
3. (*a*) W. Yang, R. G. Parr, *Proc. Nat. Acad. Sci. USA,*1985, ***82***, 6723; (*b*) P. Geerlings, F. De Proft, *Phys. Chem. Chem. Phys.,* 2008, ***10***, 3028; (*c*) R. G. Parr, W. Yang, *J. Am. Chem. Soc.* 1984, ***106***, 4049; (*d*) R. G. Parr, W. Yang, *Density functional theory of atoms and molecules. Oxford University Press, New York,* 1989.
4. S. Liu, C. Rong, T. Lu, *J. Phys. Chem. A.*, 2014, ***118***, 3698;
5. *Gaussian 16, Revision C.01*, M. J. Frisch, G. W. Trucks, H. B. Schlegel, G. E. Scuseria, M. A. Robb, J. R. Cheeseman, G. Scalmani, V. Barone, G. A. Petersson, H. Nakatsuji, X. Li, M. Caricato, A. V. Marenich, J. Bloino, B. G. Janesko, R. Gomperts, B. Mennucci, H. P. Hratchian, J. V. Ortiz, A. F. Izmaylov, J. L. Sonnenberg, D. Williams-Young, F. Ding, F. Lipparini, F. Egidi, J. Goings, B. Peng, A. Petrone, T. Henderson, D. Ranasinghe, V. G. Zakrzewski, J. Gao, N. Rega, G. Zheng, W. Liang, M. Hada, M. Ehara, K. Toyota, R. Fukuda, J. Hasegawa, M. Ishida, T. Nakajima, Y. Honda, O. Kitao, H. Nakai, T. Vreven, K. Throssell, J. A. Montgomery, Jr., J. E. Peralta, F. Ogliaro, M. J. Bearpark, J. J. Heyd, E. N. Brothers, K. N. Kudin, V. N. Staroverov, T. A. Keith, R. Kobayashi, J. Normand, K. Raghavachari, A. P. Rendell, J. C. Burant, S. S. Iyengar, J. Tomasi, M. Cossi, J. M. Millam, M. Klene, C. Adamo, R. Cammi, J. W. Ochterski, R. L. Martin, K. Morokuma, O. Farkas, J. B. Foresman, and D. J. Fox, *Gaussian, Inc., Wallingford CT*, 2019.
6. T. Lu, F. J. Chen, *Comput. Chem.* 2012, ***33***, 580.
7. CCDC: 2041102, see Electronic Supplementary Information for more details.
8. (*a*) M. Sugiura, N. Sato, Y. Sonoda, S. Kotani and M. Nakajima, *Chem.-Asian J.,* 2010, **5**, 478; (*b*) M. Sugiura, N. Sato, S. Kotani and M. Nakajima, *Chem. Commun*, 2008, **44**, 4309.

9. M. Sugiura, N. Sato, Y. Sonoda, S. Kotani and M. Nakajima, *Chem.-Asian J.,* 2010, **5**, 478.
